# Structure and Biosynthesis of Perochalasins A–C, Open-Chain Merocytochalasans Produced by the Marine-Derived Fungus *Peroneutypa* sp. M16

**DOI:** 10.1021/acs.jnatprod.4c00516

**Published:** 2024-08-16

**Authors:** Marcelo R. de Amorim, Sydney M. Schoellhorn, Camila de S. Barbosa, Giovana R. Mendes, Kamila de L. Macedo, Antonio G. Ferreira, Tiago Venâncio, Rafael V. C. Guido, Andrea N. L. Batista, João M. Batista, Elizabeth Skellam, Roberto G. S. Berlinck

**Affiliations:** † Instituto de Química de São Carlos, Universidade de São Paulo, CP 780, CEP 13560-970, São Carlos, SP, Brazil; ‡ Department of Chemistry and BioDiscovery Institute, University of North Texas, 1155 Union Circle, Denton, Texas 76203, United States; § Instituto de Física de São Carlos, Universidade de São Paulo, CEP 13563-120, São Carlos, SP Brazil; ⊥ Departamento de Química, Universidade Federal de São Carlos, CEP 13565-905, São Carlos, SP, Brazil; ∥ Universidade Federal Fluminense, Instituto de Química, Outeiro de São João Batista s/n, Niterói, RJ, 24020-141, Brazil; ¶ Universidade Federal de São Paulo. Instituto de Ciência e Tecnologia, R. Talim 330, São José dos Campos, SP 12231-280, Brazil

## Abstract

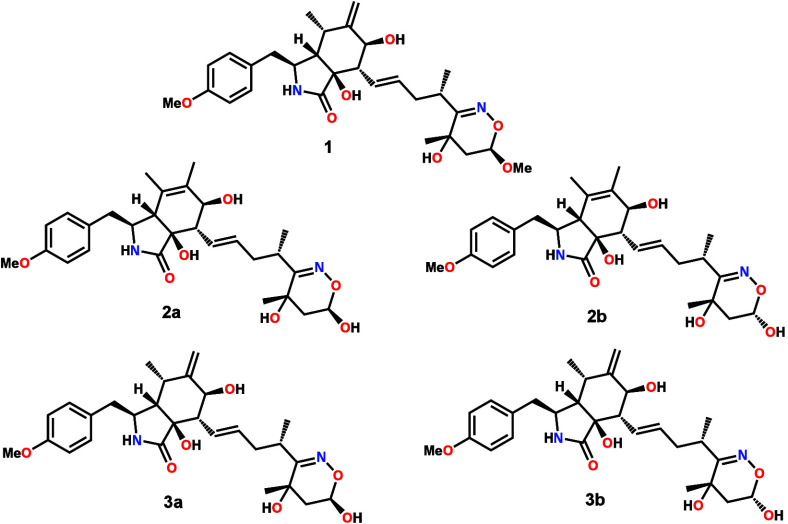

Novel open-chain merocytochalasans, perochalasins A–C (**1**–**3**), containing an unusual N–O six-membered heterocyclic moiety, were isolated from cultures of the marine-derived *Peroneutypa* sp. M16 fungus, along with cytochalasin Z_27_ (**4**), cytochalasin Z_28_ (**5**), [12]-cytochalasin (**6**), and phenochalasin B (**7**). The structures of compounds **1**–**3** were established by analysis of the spectroscopic data. Full genome sequencing of *Peroneutypa* sp. M16 enabled the identification of a cytochalasan biosynthetic gene cluster and a proposal for the biosynthetic assembly of perochalasins. The proposal is supported by the nonenzymatic conversion of phenochalasin B (**7**) into **1**–**3**, based on isotope-labeled hydroxylamine (^15^NH_2_OH and ND_2_OD) feeding studies *in vivo* and *in vitro*. In contrast to other merocytochalasans, these are the first cytochalasans confirmed to arise via nucleophilic addition and at a distinct location from the reactive macrocycle olefin, potentially expanding further the range of merocytochalasans to be discovered or engineered. Cytochalasin Z_27_ (**4**) exhibited antiplasmodial activities in the low micromolar range against the chloroquine-sensitive *Plasmodium falciparum* 3D7 strain as well as against resistant strains of the parasite (Dd2, TM90C6B, and 3D7r_MMV848).

Cytochalasans constitute a large family of fungal metabolites with diverse biological properties including antiproliferative, cytotoxic, antiplasmodial, antibacterial, and antiviral activities.^1−5^ Only 100 cytochalasans were reported between 1996 and 2009. By 2020, this number had grown to 500 representative metabolites.^6,7^ Cytochalasans present a tricyclic framework composed of a macrocycle fused to a perhydroisoindole moiety, which is constructed by four core enzymes: a polyketide synthase/nonribosomal peptide synthetase (PKS-NRPS), a *trans*-acting enoylreductase (*trans*-ER), an α,β-hydrolase (HYD), and a Diels–Alderase (DA) that promotes the formation of a 4,5-dimethyl-2,3,3a,6,7,7a-hexahydro-1*H*-isoindol-1-one moiety.^8−13^

Subsequent functional group additions and conversions result from reactions with a variety of tailoring enzymes, including cytochrome P450 monooxygenases (P450s/CYP),^14,15^ oxidoreductases (OXR),^15,16^ Baeyer–Villiger monooxygenases (BVMO),^17^ and acetyltransferases (OAcT).^18^ Such functionalizations contribute to the chemical diversity of cytochalasans. Further complex motifs presenting tetracyclic or polycyclic carbon frameworks, as well as the merocytochalasan subfamily of metabolites, derive from intermolecular condensations between one or more non-cytochalasan metabolites and a cytochalasan-derived natural scaffold.^19−22^ The formation of polycyclic and merocytochalasans has recently been shown to be a nonenzymatic process.^16,23^

Our previous investigations on cultures of the fungus *Peroneutypa* sp. M16 indicated that fractions of the organic extract exhibited antiplasmodial activities and contained a variety of PKS and terpenoid-derived metabolites.^24^ Herein we report that further investigation on *Peroneutypa* sp. M16 metabolites led to the isolation and identification of the yet undescribed cytochalasans perochalasin A (**1**), perochalasin B/*epi*-perochalasin B (**2a**+**2b**), and perochalasin C/*epi*-perochalasin C (**3a**+**3b**); **2a**+**2b** and **3a**+**3b** were isolated as pairs of inseparable epimeric compounds. Known cytochalasin Z_27_ (**4**),^25^ cytochalasin Z_28_ (**5**),^25^ [12]-cytochalasin (**6**),^26^ and phenochalasin B (**7**)^27^ were also isolated and identified. The new cytochalasan derivatives (**1**, **2a**+**2b**, and **3a**+**3b**) represent an unusual open macrocyclic skeleton, with an as yet unreported 5,6-dihydro-4*H*-1,2-oxazine moiety. We also describe the identification of the biosynthetic gene cluster (BGC) within the *Peroneutypa* sp. M16 genome and the use of isotope-labeled feeding studies to understand the biosynthetic origin of the novel 5,6-dihydro-4*H*-1,2-oxazine cyclic functionality.

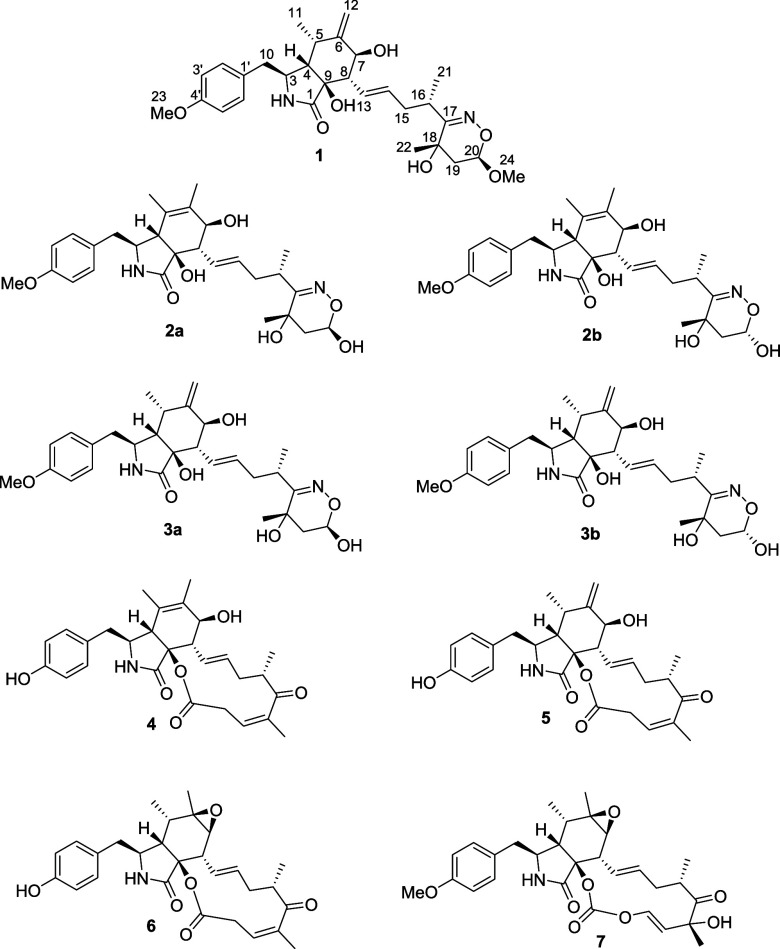



## Results and Discussion

*Peroneutypa* sp. M16 was cultivated under both still and shaking conditions in potato dextrose broth (PDB) medium in artificial seawater. Fractions previously obtained from still cultures of *Peroneutypa* sp. M16^24^ were fractionated by C18 solid phase extraction (SPE) and semipreparative HPLC-UV (see Experimental Section for details), resulting in the isolation of compounds **1**, **2a+2b**, **3a+3b**, **4**, and **5**. Under shaking conditions, compounds **6** and **7** were isolated by similar steps of purification.

**Figure 1:**
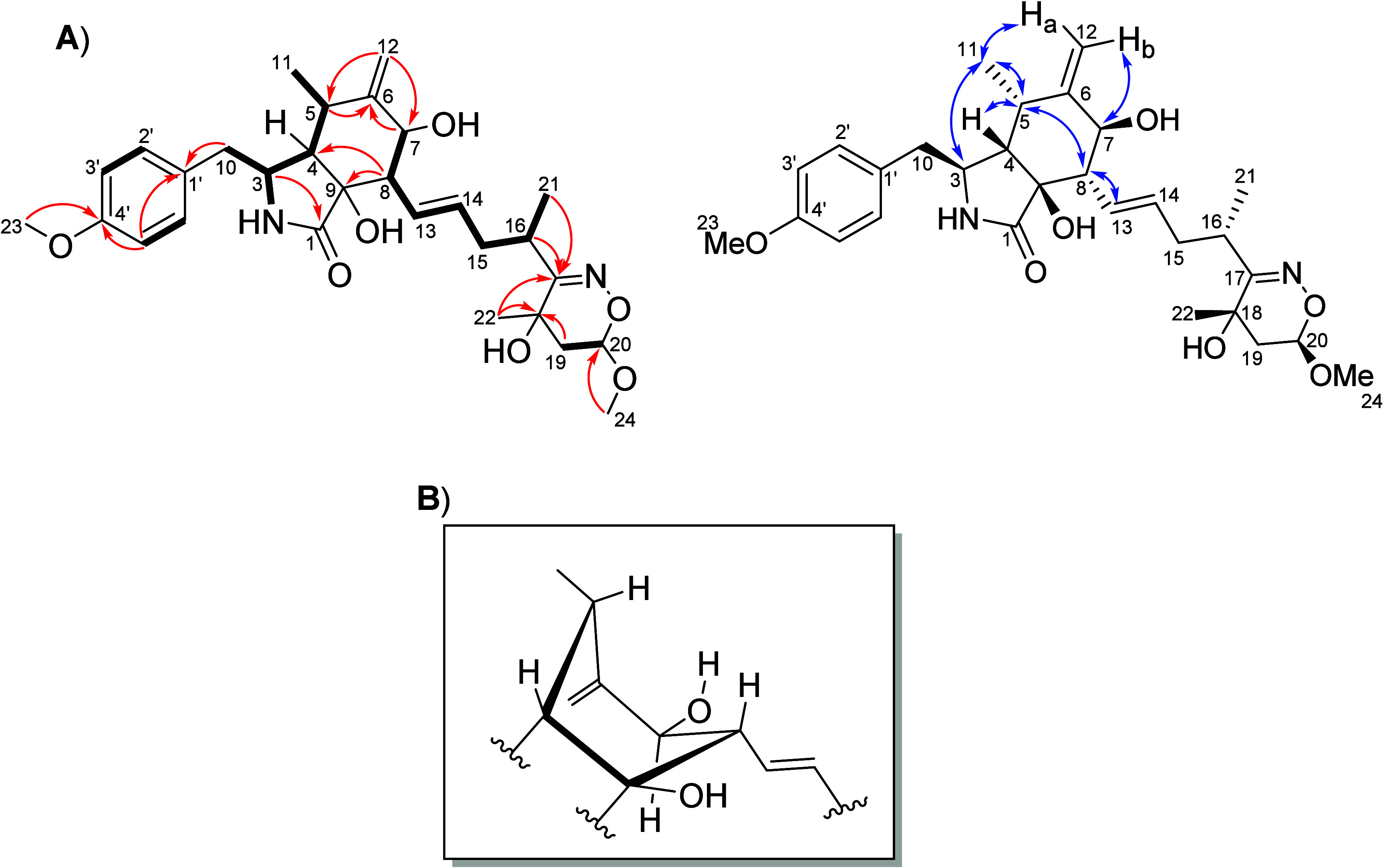
(A) Key HMBC (red arrow) and COSY (bold bonds) correlations and NOESY (blue arrows) interactions of **1**. (B) Representation of the boat conformation for the six-membered ring of perochalasin A (**1**).

Compound **1** was obtained as a colorless amorphous solid, with [α]_D_^23^ +77.0 (*c* 0.06; MeOH). Analysis by HRESIMS indicated the molecular formula C_29_H_40_N_2_O_7_, corresponding to 11 double-bond equivalents (DBE). Analysis of 1D and 2D NMR data of **1** identified the presence of the perhydroisoindole and *O*-methyl phenyl residue moieties, with ^1^H and ^13^C chemical shifts typically observed for a cytochalasan skeleton (Table 1).^27,28^ Its UV spectrum exhibited absorption maxima at λ_max_ 223, 247, and 271 nm. Analysis of the COSY spectrum indicated the presence of the spin systems -(CH-7)-(CH-8)-(CH-13)-(CH-14)-(CH-15)-(CH-16)-(CH-21)- and -(CH_2_-19)-(CH-20)- (Figure 1). HMBC correlations of H_3_-21 and H-16 with C-17 (δ_C_ 151.7) and correlations of H_3_-22 with C-17, C-18 (δ_C_ 77.0) and C-19 (δ_C_ 42.6), along with the COSY correlations, demonstrated that these spin systems were connected through the nonprotonated carbons C-17/C-18. The HMBC correlation between the methoxy group (δ_H_ 3.60; OCH_3_-24) and C-20 (δ_C_ 99.2) defined its position. The chemical shifts of CH-20, C-17, and C-18 were typical of electronegative atom substituted carbons. Such data, along with the molecular formula, enabled us to place a hydroxy group at C-18, a nitrogen atom at C-17, and an oxygen at C-20. The nitrogen and oxygen were connected to support the molecular formula. Additional support for the structure hypothesis was provided by ^13^C NMR chemical shift calculations (see below). Thus, the planar structure of compound **1** was defined as a novel open-chain cytochalasan with a unique 5,6-dihydro-4*H*-1,2-oxazine moiety, named perochalasin A.

**Table 1:** ^1^H (600 MHz) and ^13^C (150 MHz) NMR Data for **1** (DMSO-*d*_6_)

**No**.	***δ*****_C_, type**	***δ***_**H**_, **mult**. **(*****J*****in Hz)**
1	174.8, C	-
2-NH	-	7.70, br s
3	52.1, CH	3.11, dd (12.0, 5.4)
4	50.8, CH	2.06, t (5.2)
5	30.1, CH	2.82, m
6	151.2, C	-
7	71.4, CH	3.69, d (8.8
8	52.5, CH	2.38, t (8.8)
9	76.9, C	-
10	41.6, CH_2_	2.70, dd (13.6, 6.1)
		2.65, dd (13.6, 5.4)
11	14.4, CH_3_	0.78, d (7.0)
12a	110.7, CH_2_	4.84, br s
12b		5.05, br s
13	129.2, CH	5.53, dd (15.2, 9.2)
14	131.3, CH	5.35, dt (15.2, 7.4)
15	35.5, CH_2_	2.49, m overlapped
		2.22, dt (13.4, 7.2)
16	30.5, CH	2.81, m
17	151.7, C	-
18	77.0, C	-
19	42.6, CH_2_	2.35, dd (13.7, 7.1)
		1.89, dd (13.7, 3.2)
20	99.2, CH	4.95, dd (7.0, 3.2)
21	15.0, CH_3_	1.19, d (7.0)
22	27.4, CH_3_	1.37, (s)
1′	129.2, C	-
2′	130.8, CH	7.10, d (8.5)
3′	113.6, CH	6.85, d (8.5)
4′	157.8, C	-
5′	113.6, CH	6.85, d (8.5)
6′	130.8, CH	7.10, d (8.5)
23-OCH_3_	54.9, CH_3_	3.71, s
24-OCH_3_	59.0, CH_3_	3.60, s

To investigate the stereochemistry of **1**, analysis of the NOESY spectrum showed a correlation between H-8 (δ_H_ = 2.39) and H-5 (δ_H_ = 2.82) (Figure S13), indicating a boat conformation for the six-membered ring with these protons as β-oriented (Figure 1), a preferential conformation resulting from the equatorial orientation of the bulky acyclic moiety of **1**. NOE interactions between H_3_-11 (δ_H_ 0.78) and H-3 (δ_H_ 3.11) (Figure S14), as well as between H-4 (δ_H_ 2.06) and H-5 (Figure S15), indicated that H_3_-11 and H-3 were cofacial with an α-orientation, while the H-4, H-5, and H-8 were in a β-orientation. The *pseudo*-diaxial coupling *J*_H-7/H-8_ = 8.8 Hz by the ^1^H NMR spectrum (Figure S5), together with only a single NOE interaction with H-12b (δ_H_ 5.05) when H-7 (δ_H_ 3.69) was irradiated (Figure S16), suggested an α-orientation for H-7. The NOESY interactions observed for **1** revealed that OH-9 only had the possibility to be in a β-orientation, corroborating with the fixed relative configuration at C-9.^3^ Irradiation on H-20 (δ_H_ 4.95) did not exhibit a NOE interaction with H_3_-22 (δ_H_ 1.37) (Figure S17) as well, suggesting that H-20 and H_3_-22 were on opposite sides of the 5,6-dihydro-4*H*-1,2-oxazine.

**Figure 2:**
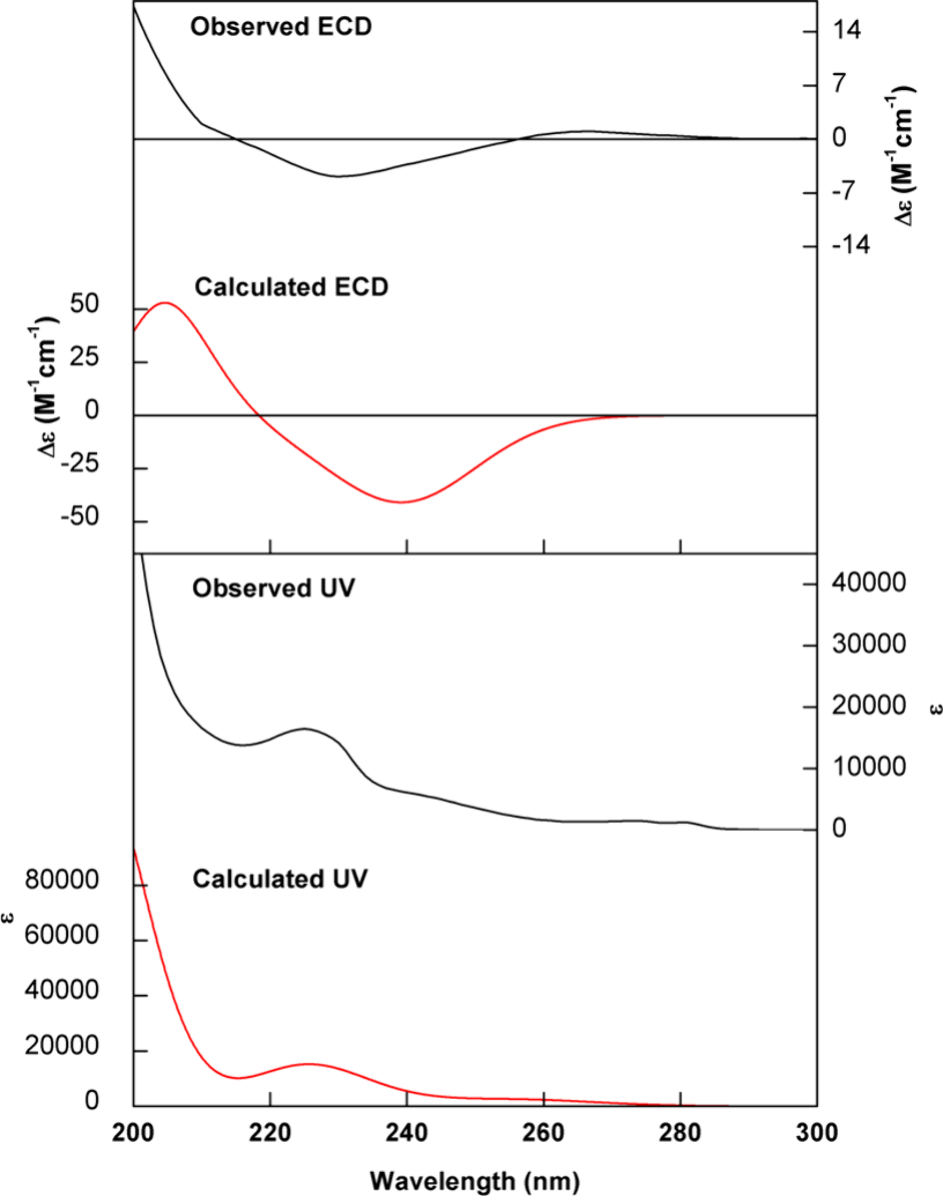
Comparison of experimental UV and ECD spectra (black trace) of (+)-**1** in MeOH with calculated data (red trace) at the CAM-B3LYP/PCM(MeOH)/TZVP level for the 3*S*,4*S*,5*S*,7*S*,8*S*,9*S*,13*E*,16*S*,18*S*,20*R* configuration.

Although the relative configurations of the perhydroisoindole and 5,6-dihydro-4*H*-1,2-oxazine moieties could be determined by analysis of the NOESY data, the relative position of the methyl group at C-16 remained unassigned due to the flexibility of the acyclic side chain moiety. Nevertheless, multiple stereoisomeric combinations were possible among the different structural moieties, since no NOE correlations were observed either between H_3_-21 and the 5,6-dihydro-4*H*-1,2-oxazine or between the latter and the perhydroisoindole moiety. Therefore, ^13^C NMR chemical shift calculations were performed for all possible stereoisomeric compositions of **1** and compared to the experimental ^13^C NMR data. Considering the three stereogenic units, namely, the perhydroisoindole moiety, the methyl group H_3_-21, and the 5,6-dihydro-4*H*-1,2-oxazine, a total of eight stereoisomers were possible, out of which four were considered for relative configuration determination. DP4+ statistical analysis^29^ (Table S5) indicated perochalasin A (**1**) to have the 3S*,4S*,5S*,7S*,8S*,9S*,16S*,18S*,20R* relative configuration (>99.9% confidence, Table S5). Following the assignment of the relative configuration of **1**, we proceeded to determine its absolute configuration by an analysis of electronic circular dichroism (ECD) spectroscopy data. The agreement between experimental and calculated UV/ECD spectra led to the assignment of (+)-**1** as 3*S*,4*S*,5*S*,7*S*,8*S*,9*S*,13*E*,16*S*,18*S*,20*R* (Figure 2).

Analysis of ^1^H and ^13^C NMR spectra of compounds **2a**+**2b** indicated data similar to those of **1**, except for the replacement of the methoxy group at C-20 in **1** by a hydroxy group in **2a**+**2b**. Also, the position of the double bond in the cyclohexane moiety changed from C-5/C-6 in **1** to C-6/C-12 in **2a**+**2b**. The mixture of epimers **2a**+**2b** was established by the analysis of NMR data (Table 2).

**Table 2:** ^1^H (600 MHz) and ^13^C (150 MHz) NMR Data for Epimer Mixture **2a**+**2b** (DMSO-*d*_6_)

	**2a**		**2b**	
**No**.	***δ***_**C**_, **type**	***δ***_**H**_, **mult**. **(*****J*****in Hz)**	***δ***_**C**_, **type**	***δ***_**H**_, **mult**. **(*****J*****in Hz)**
1	175.0, C	-	174.8, C	-
2-NH	-	7.75, d (3.8)	-	7.75, d (3.8)
3	56.8, CH	3.27, m	57.0, CH	3.27, m
4	51.6, CH	2.30, m	51.7, CH	2.30, m
5	125.0, C	-	125.1, C	-
6	131.2, C	-	131.0, C	-
7	70.8, CH	3.62, m	70.9, CH	3.62, m
8	52.3, CH	2.25, m	52.3, CH	2.30, m
9	76.2, C	-	76.1, C	-
10	41.2, CH_2_	2.80, m	41.2, CH_2_	2.80, m
		2.93, dd (13.2, 7.2)		2.93, dd (13.2, 7.2)
11	17.4, CH_3_	1.38, br s	17.5, CH_3_	1.44, br s
12	15.8, CH_3_	1.60, br s	16.1, CH_3_	1.62, br s
13	128.7, CH	5.62, dd (15.2, 9.3)	128.7, CH	5.62, dd (15.2, 9.3)
14	131.8, CH	5.35, dd (15.2, 7.7)	132.0, CH	5.32, dd (15.2, 8.3)
15	35.3, CH_2_	2.55, m	34.2, CH_2_	2.49, m overlapped
		2.20, dt (13.4, 6.8)		2.28, m
16	30.6, CH	2.78, m	30.4, CH	2.63, q (7.2)
17	150.0, C	-	149.4, C	-
18	76.7, C	-	74.1, C	-
19	44.0, CH_2_	1.84, dd (13.2, 3.9)	44.6, CH_2_	1.78, dd (12.9, 7.1)
		2.32, dd (13.2, 6.8)		2.42, dd (12.9, 6.5)
20	91.5, CH	5.09, m	89.6, CH	4.95, m
21	15.1, CH_3_	1.20, d (7.0)	14.7, CH_3_	1.17, d (7.0)
22	27.6, CH_3_	1.39, s	26.7, CH_3_	1.27, s
1′	129.4, C	-	129.4, C	-
2′	130.8, CH	7.14, d (8.6)	130.8, CH	7.14, d (8.6)
3′	113.7, CH	6.87, d (8.6)	113.7, CH	6.87, d (8.6)
4′	157.8, C	-	157.8, C	-
5′	113.7, CH	6.87, d (8.6)	113.7, CH	6.87, d (8.6)
6′	130.8, CH	7.14, d (8.6)	130.8, CH	7.14, d (8.6)
4′-OCH_3_	54.9, CH_3_	3.72, s	54.9, CH_3_	3.72, s
7-OH	-	4.61, d (7.9)	-	4.60, d (7.9)
9-OH	-	5.22, s	-	5.14, s
18-OH	-	5.26, br s	-	5.29, br s
20-OH	-	7.39, d (4.4)	-	7.19, d (4.3)

**Figure 3:**
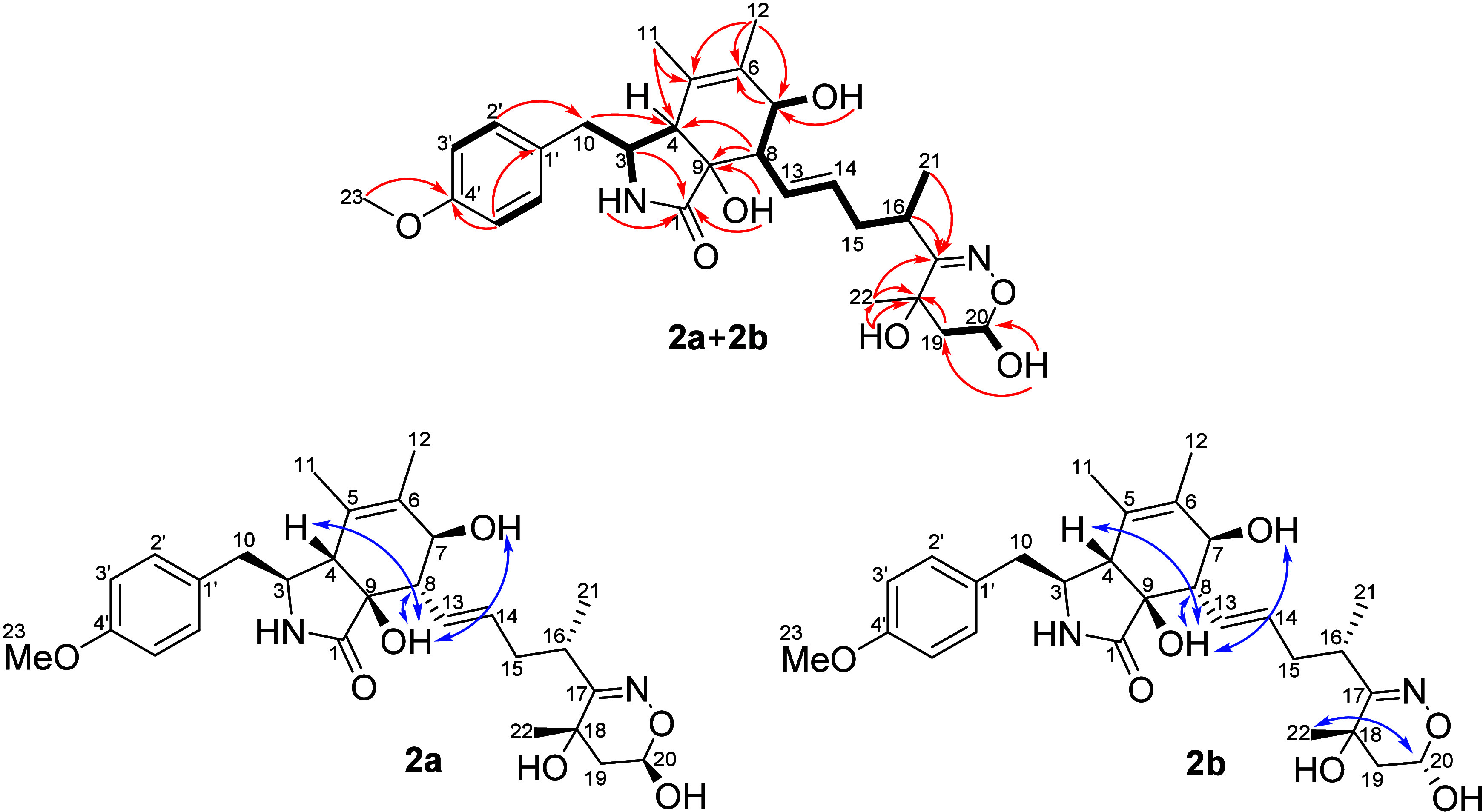
Key HMBC (red arrows) and COSY (bold bonds) correlations and NOESY (blue arrows) interactions of **2a**+**2b**.

Hydrogen integrals in a 1:1 ratio were observed for all ^1^H signals for structures **2a** and **2b**. The epimeric mixture was obtained as a white oil with [α]_D_^23^ +63.5 (*c* 0.27; MeOH). The HMBC correlations of the hydroxy signal at δ_H_ 5.22 with C-1 (δ_C_ 175.0), C-9 (δ_C_ 76.2), and C-13 (δ_C_ 175.0) (Figure 3) confirmed an open macrocyclic portion similar to that observed for **1**. The molecular formulas of **2a** and **2b** epimers were established as C_28_H_38_N_2_O_7_ by HRESIMS ([M + H]^+^ at *m*/*z* 515.2755, calculated for C_28_H_39_N_2_O_7_^+^, 515.2752) and NMR data analysis. The UV spectrum was identical to that of compound **1**, with absorption maxima at λ_max_ 223, 247, and 273 nm.

The hydroxy groups OH-9 of **2a** and **2b** (δ_H_ 5.22 for **2a** and δ_H_ 5.14 for **2b**) exhibited NOEs with H-4 (δ_H_ 2.30 for **2a** and **2b**), H-8 (δ_H_ 2.25 for **2a** and δ_H_ 2.30 for **2b**), and OH-7 (δ_H_ 4.61 for **2a** and δ_H_ 4.60 for **2b**) (Figure 3), suggesting that H-4, H-8, OH-7, and OH-9 were β-oriented (Figure S30). An additional NOE interaction between H-3 (δ_H_ 3.27 for **2a** and **2b**) and H-13 (δ_H_ 5.62) indicated an α-orientation for H-3 (Figure 3) and the pentacyclic moiety in an envelope conformation (Figure S33). A NOE correlation observed between H-20 in **2b** (δ_H_ 4.95) and H_3_-22 (δ_H_ 1.27) showed the main difference between the two epimers, because the signal for H-20 in **2a** (δ_H_ 5.09) did not show a NOE correlation with H_3_-22 (δ_H_ 1.39) (Figure S31). The relative configuration at C-16 for the epimers was determined as S* based on the nonenzymatic reactions of the precursor phenochalasin B with hydroxylamine carried out in the *in vitro* and *in vivo* experiments discussed below. Thus, the epimeric relationship between **2a** and **2b** was defined, and these compounds were named perochalasin B (**2a**) and *epi*-perochalasin B (**2b**).

The second 1:1 epimeric mixture, **3a**+**3b**, was similarly identified by analysis of NMR data (Table 3), also obtained as a colorless oil with [α]_D_^23^ +60.4 (*c* 0.1; MeOH). Similarly to perochalasin A (**1**), the **3a**+**3b** epimeric mixture indicated differences only by replacement of a methoxy group by a hydroxy group at C-20. The molecular formula was identical to that of **2a**+**2b**, established by HRESIMS and NMR data analysis.

**Table 3:** ^1^H (600 MHz) and ^13^C (150 MHz) NMR Data for Epimer Mixture **3a**+**3b** (DMSO-*d*_6_)

	**3a**		**3b**	
**No**.	***δ***_**C**_, **type**	***δ***_**H**_, **mult**. **(*****J*****in Hz)**	***δ***_**C**_, **type**	***δ***_**H**_ , **mult**. **(*****J*****in Hz)**
1	174.7	-	174.8	-
2-NH	-	7.72, d (3.3)	-	7.72, d (3.3)
3	52.1, CH	3.11, quint (5.7)	52.1, CH	3.11, quint (5.7)
4	50.8, CH	2.05, m	51.0, CH	2.06, m
5	29.9, CH	2.82, m	29.8, CH	2.82, m
6	151.0, C	-	151.1, CH	-
7	71.7, CH	3.69, d (8.4)	71.8, CH	3.70, d (8.4)
8	52.4, CH	2.40, m	52.4, CH	2.40, m
9	76.9, C	-	76.7, C	-
10	41.6, CH_2_	2.67, m	41.7, CH_2_	2.67, m
11	14.4, CH_3_	0.78, d (7.5)	14.4, CH_3_	0.79, d (7.5)
12a	110.7, CH_2_	4.84, br s	110.7, CH_2_	4.84, br s
12b		5.04, br d (1.3)		5.04, br d (1.3)
13	129.0, CH	5.50, dd (15.0, 9.3)	129.2, CH	5.55, dd (15.0, 9.1)
14	131.5, CH	5.37, dt (15.0, 7.3)	131.4, CH	5.31, ddd (15.0, 8.3, 6.6)
15	35.6, CH_2_	2.54, m	34.4, CH_2_	2.48, m
		2.20, dt (13.4, 6.7)		2.29, m
16	30.5, CH	2.82, m	30.4, CH	2.66, m
17	150.1, C	-	149.6, C	-
18	77.1, C	-	74.2, C	-
19	44.0, CH_2_	2.32, dd (13.1, 6.9)	44.7, CH_2_	2.41, m
		1.84, dd (13.1, 4.0)		1.79, dd (13.0, 7.1)
20	91.4, CH	5.12, dd (6.9, 4.0)	89.6, CH	4.95, t (6.9)
21	15.3, CH_3_	1.20, d (7.1)	14.7, CH_3_	1.18, d (7.1)
22	27.5, CH_3_	1.38, s	26.7, CH_3_	1.28, s
1′	129.1, C	-	129.1, C	-
2′	130.8, CH	7.09, d (8.7)	130.8, CH	7.11, d (8.7)
3′	113.6, CH	6.85, d (8.7)	113.6, CH	6.85, d (8.7)
4′	157.8, C	-	157.8, C	-
5′	113.6, CH	6.85, d (8.7)	113.6, CH	6.85, d (8.7)
6′	130.8, CH	7.09, d (8.7)	130.8, CH	7.11, d (8.7)
4′-OCH_3_	54.9, CH_3_	3.72, s	54.9, CH_3_	3.72, s

**Figure 4:**
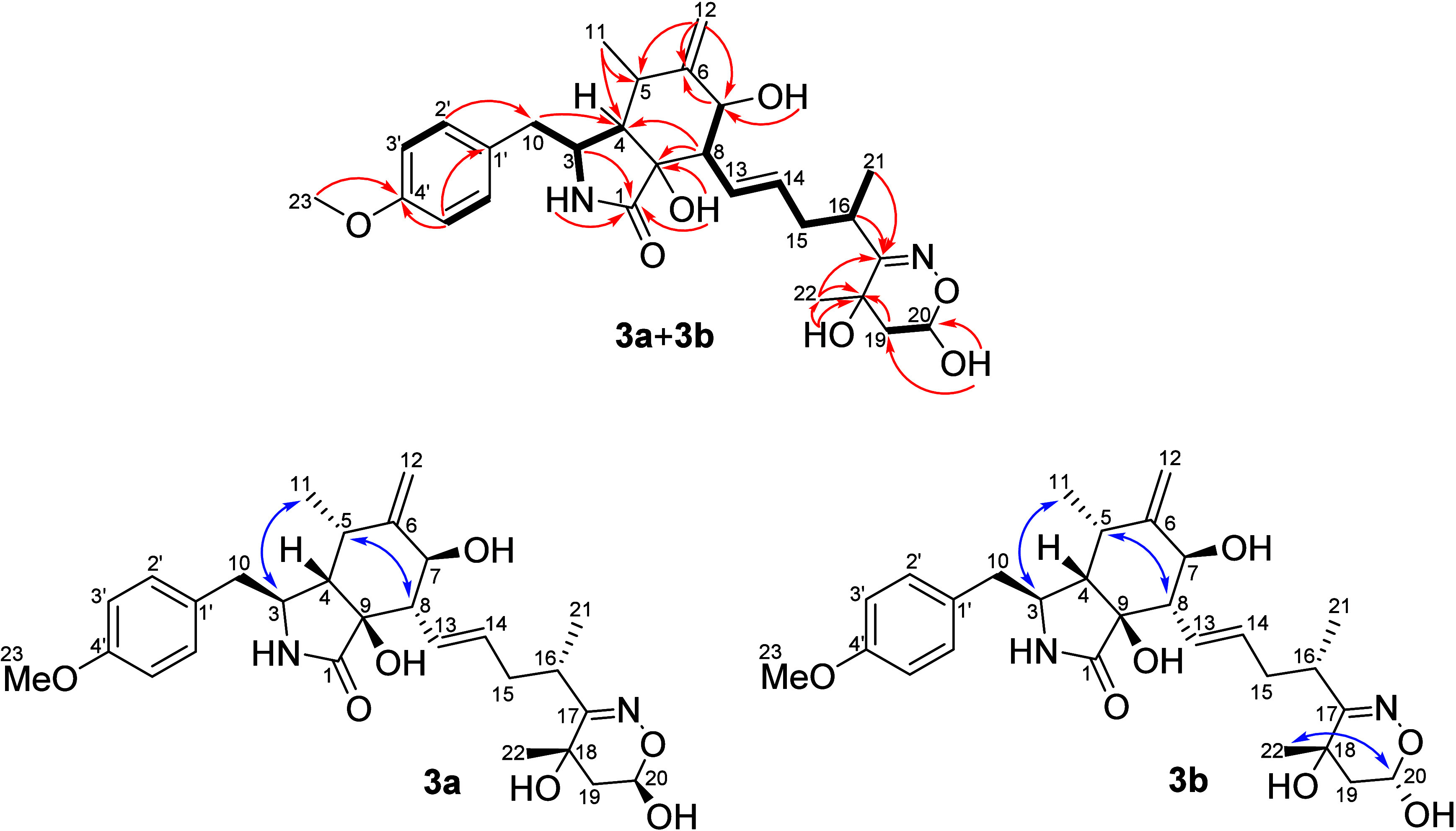
Key HMBC (red arrow) and COSY (bold bond) correlations and NOESY (blue arrow) interactions of **3a**+**3b**.

**Figure 5:**
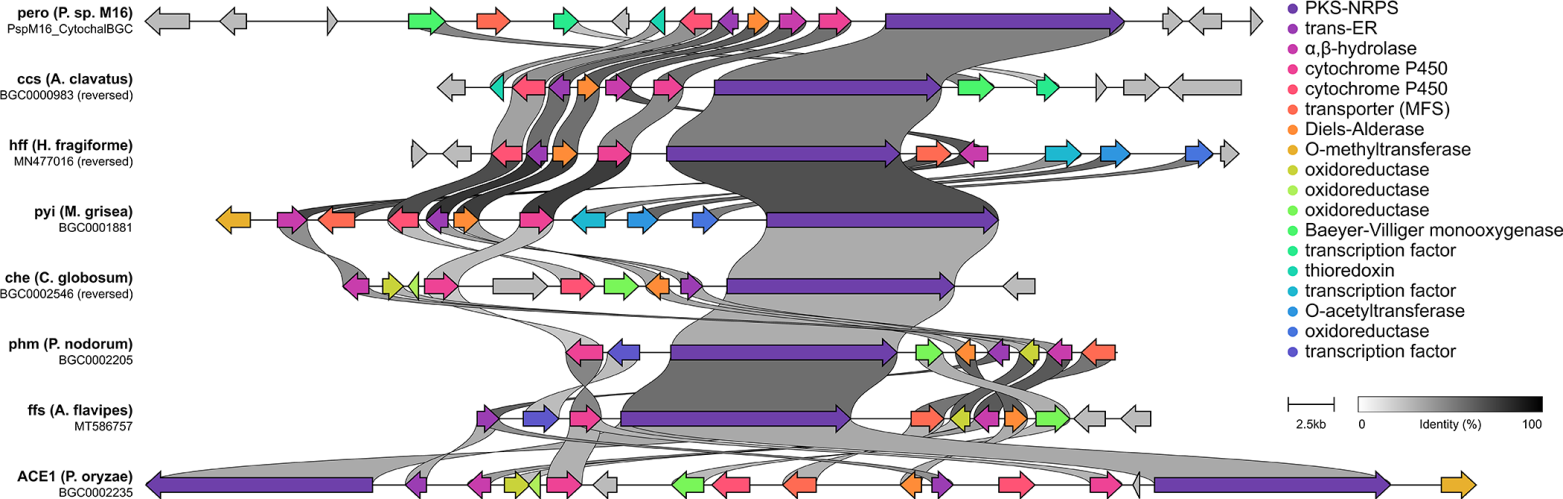
Synteny analysis between the cytochalasan BGC identified in *Peroneutypa* sp. M16 and experimentally validated cytochalasan BGCs.^8−10,15,18,32,33^ Gene color code is described in the figure key.

Once the planarity of **3a**+**3b** was defined, NOE correlations were observed between H-8 (δ_H_ 2.40 for **3a** and **3b**) and H-5 (δ_H_ 2.82 for **3a** and **3b**) (Figure S45), as well as between H-3 (δ_H_ 3.11 for **3a** and **3b**) and H_3_-11 (δ_H_ 0.78 for **3a** and δ_H_ 0.79 for **3b**) (Figure S46), data that suggested that H-3 and H_3_-11 were α-oriented, while H-4, H-5, and H-8 were β-oriented (Figure 4) as for **1**. The *pseudo*-diaxial coupling of *J*_H-7/H-8_ = 8.4 Hz defined H-7 as α-oriented, like that assigned for **1**. A NOE correlation between H-20 (δ_H_ 4.95 for **3b**) and H_3_-22 (δ_H_ 1.28) was verified only for **3b** (Figure 5), while in **3a** H-20 (δ_H_ 5.12) did not exhibit an NOE interaction with H_3_-22 (δ_H_ 1.38) (Figure S47). In the same way for **2a**+**2b**, the relative configuration at C-16 for epimers **3a**+**3b** was assigned as S* based on the phenochalasin B reaction with hydroxylamine as described below in the *in vitro* and *in vivo* experiments. These compounds were named perochalasin C (**3a**) and *epi*-perochalasin C (**3b**).

Known metabolites isolated from the cultures of *Peroneutypa* sp. M16 were identified as cytochalasin Z_27_ (**4**),^25^ cytochalasin Z_28_ (**5**),^25^ [12]-cytochalasin (**6**),^26^ and phenochalasin B (**7**),^27^ by comparison of NMR data with literature data. Phenochalasin B (**7**) was reported in 1999 with a 21,23-dioxa,17,22-dione cytochalasan moiety containing a unique *O*-methyl phenyl residue at C-10.^27^ Its relative configuration was assigned as 3S*,4S*,5S*,6R*,7S*,8S*,9S*,16S*,18R* by X-ray diffraction analysis in 2015.^30^

Several investigations into the biosynthesis of cytochalasans revealed that fungi have more than one BGC encoding cytochalasans.^10,16,18^ Based on the structural uniqueness of compounds **1**–**7**, we decided to investigate the biosynthesis of the 5,6-dihydro-4*H*-1,2-oxazine moiety. After genome sequencing of *Peroneutypa* sp. M16, analysis using fungiSMASH^31^ identified 53 BGCs. Of these, only three presented PKS-NRPS genes, and only one BGC, located on scaffold 14, contained all four core genes necessary for cytochalasan biosynthesis (Table S2).^32^

Cytochalasans **1**–**3** and **7** include a *para*-methoxy group, likely introduced by an *O*-methyltransferase (*O*MeT).^18^ Similarly, the identification of cytochalasins Z_27_ (**4**) and Z_28_ (**5**), [12]-cytochalasin (**6**), and phenochalasin B (**7**), which contain one or more oxygen atoms incorporated into the macrocyclic portion, indicates the requirement of a Baeyer–Villiger monooxygenase.^17^ The putative cytochalasan BGC on scaffold 14 encodes both *O*MeT and BVMO, indicating that it should be responsible for the biosynthesis of **1**–**7**. This BGC was named *pero* (GenBank Accession No. PQ149227). A detailed gene cluster comparison analysis was performed against other characterized cytochalasan BGCs^8−10,15,18,33,34^ using Clinker,^35^ confirming high homology between clusters (Figure 5).

Cytochalasan derivatives **1**–**3** contain an unusual 5,6-dihydro-4*H*-1,2-oxazine moiety. Oxime-related functional groups are rare, reported in microbial and marine natural products.^36−38^ Biosynthetic investigations have revealed that the oxime functional group can be introduced by oxidases;^39^ however, homologues of such enzymes were not identified in the proximity of the *pero* BGC.

In a previous investigation of *Peroneutypa* sp. M16 metabolites, the new solanapyrone S also presented an oxime group, likely derived from hydroxylamine.^24^ Therefore, we searched the genome to identify the solanapyrone BGC (Figure S2 and Table S3). Careful examination of the solanapyrone BGC (GenBank Accession No. PQ149228) identified genes encoding solanapyrone synthase (a HRPKS; *sol1*), an *O*-methyltransferase (*O*MT; *sol2*), a dehydrogenase (ADH; *sol3*), a transcription factor (TF; *sol4*), a flavin-dependent monooxygenase (Oxd; *sol5*), and a P450 (*sol6*), genes essential for solanapyrone biosynthesis.^40^ Nevertheless, the genes immediately upstream and downstream of the *sol* BGC did not appear to have a biosynthetic function, and there was no obvious oxidase or transferase identified in the solanapyrone BGC.

Therefore, we hypothesize that compounds **1**–**3** are examples of merocytochalasans and may have reacted nonenzymatically with hydroxylamine, a degradation product of ammonia.^41^ To investigate this hypothesis, we added 0.1 mg/mL [^15^N]-hydroxylamine to 4-day-old cultures of *Peroneutypa* sp. M16 and analyzed the 7-day-old cultures’ organic extracts by LC-MS. Isotopic incorporation was noted by monitoring with HPLC-MS the ions at *m*/*z* 516 [M + 1 + H]^+^ and *m*/*z* 498 [M – H_2_O + 1 + H]^+^ that have the same retention time as compounds **2a**+**2b** and **3a**+**3b** (*m*/*z* 515 [M + H]^+^ and *m*/*z* 497 [M – H_2_O + H]^+^, respectively) (Figures S50–S56). A solution of [^15^N]-glycine was added as a control to a second batch of *Peroneutypa* sp. M16 cultures at a final concentration of 0.1 mg/mL; however, the expected *m*/*z* signal was not observed, ruling out the possibility of amine oxidation as the source of the oxime functional group. We therefore concluded that hydroxylamine was incorporated into the cytochalasan macrocycle by nucleophilic attack at the C-17 ketone, followed by elimination of water and cyclization. Consistent with this hypothesis, adding deuterium-labeled hydroxylamine (ND_2_OD) did not lead to any change in mass for compounds **1**–**3**.

To further establish the nonenzymatic nature of hydroxylamine incorporation into merocytochalasans **1**–**3**, we purified 5.1 mg of **7** from cultures of *Peroneutypa* sp. M16. First we incubated **7** in MeOH/H_2_O with 1% formic acid for 1, 4, and 6 days. Although there was clearly some degradation of **7**, merocytochalasans **1**–**3** could not be detected (Figures S57–S62). We then incubated **7** (1 mg; 2 μmol) in MeOH/H_2_O + 1% formic acid + 2 μmol of ^15^*N*-hydroxylamine for 1, 4, and 6 days. This time we observed small amounts of merocytochalasans **1**–**3**, each exhibiting an increased mass of 1 mu (i.e., *m*/*z* 516 [M + 1 + H]^+^ and *m*/*z* 530 [M + 1 + H]^+^ for **2a+b**/**3a+b** and **1**, respectively), forming after just 1 day and accumulating over time (Figures S57–S62). As a control, we repeated the experiment using ND_2_OD and observed the production of merocytochalasans **1**–**3** with natural mass (i*.*e., *m*/*z* 515 [M + H]^+^ and *m*/*z* 529 [M + H]^+^ for **2a+b**/**3a+b** and **1**, respectively (Figures S57–S62)).

**Scheme 1:**
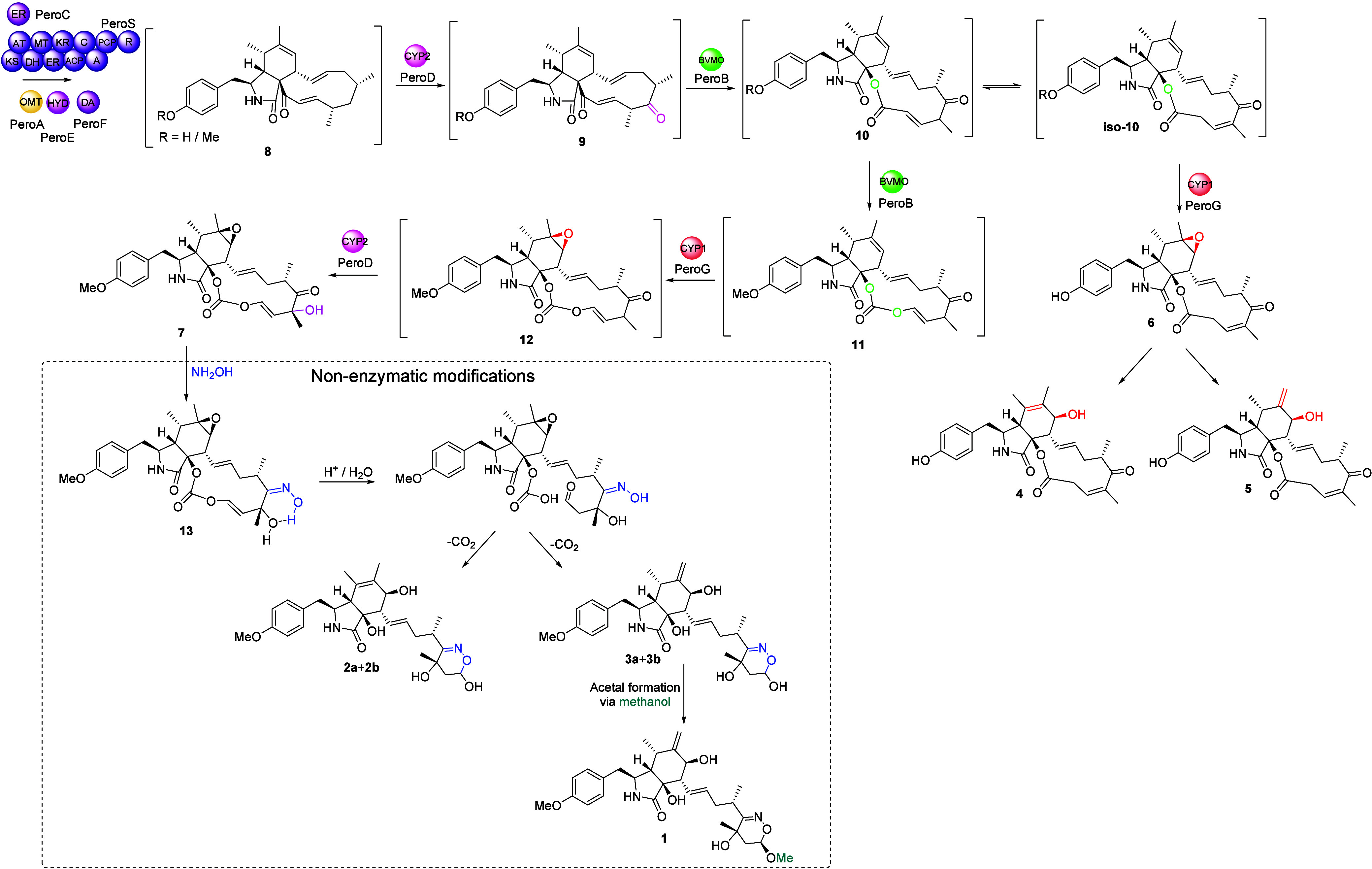
Proposed Biosynthesis of Cytochalasans **4**–**7** and Nonenzymatic Conversion of **7** into **1**–**3** Abbreviations: KS = ketosynthase; AT = acyltransferase; DH = dehydrogenase; MT = methyltransferase; ER = enoylreductase; KR = ketoreductase; ACP = acyl carrier protein; C = condensation; A = adenylation; PCP = peptidyl carrier protein; R = reductase; HYD = *α,β*-hydrolase; DA = Diels-Alderase; OMT = methyltransferase; CYP = cytochrome P450 monooxygenase; BVMO = Baeyer–Villiger monooxygenase.

Considering the results of labeled-hydroxylamine incorporation into **1**–**3**, we propose a biosynthetic pathway to explain the formation of cytochalasans **4**–**7** and nonenzymatic conversion into **1**–**3** (Scheme 1). The PKS-NRPS (PeroS), *trans*-ER (PeroC), α,β-hydrolase (PeroE), and Diels–Alderase (PeroF) collaborate to synthesize the cytochalasan core scaffold **8**. Depending on whether the A domain of the PKS-NRPS accepts tyrosine or methyltyrosine, generated by the *O*-methyltransferase (PeroA), two possible products could be formed. Next the cytochrome monooxygenase P450 (PeroD) oxidizes **8** to **9**, which possesses a keto group, necessary for the BVMO to function.^17^ The BVMO (PeroB) can complete a single oxygen insertion, generating **10**, and if the second P450 (PeroG) acts, cytochalasan **6** would be generated via epoxidation at C-6/C-7. Epoxide opening would explain the observation of cytochalasans **4** and **5**. Concurrently, PeroB introduces a second oxygen atom into the macrocycle, resulting in the carbonate-containing intermediate **11**, followed by a PeroG-mediated epoxidation/ring-opening sequence, described above, to generate **12**. Based on the observed hydroxy group at C-18 in compounds **1**–**3** and **7**, and no additional P450 candidates in the *pero* BGC, we propose that this hydroxy group is also introduced by PeroD converting **12** to **7**. It has been shown in chaetoglobosin A biosynthesis that P450s can act iteratively on the macrocycle;^15^ an iterative P450 has also been proposed in cytochalasin E/K biosynthesis,^8^ which shares many similarities to our proposed biosynthesis of **4**–**7**.

Nucleophilic addition of hydroxylamine with **7**, followed by loss of H_2_O, is a common reaction of ketones and could explain the formation of the proposed intermediate **13** (Scheme 1). Loss of CO_2_ and hemiacetal formation would explain the observation of merocytochalasans **2** and **3** (see Scheme S1 for details). The presence of MeOH during extraction and in *in vitro* reactions converts **3** to **1**, similar to other cytochalasans isolated using MeOH.^42,43^ Open-chain cytochalasans at the aldehyde oxidation state have been reported, supporting this proposed mechanism.^42,44^ Similarly, phomopsisin A, an unusual cytochalasan possessing a 2*H*-isoxazole moiety, is proposed to arise via incorporation of hydroxylamine at the C-17 ketone; however, this hypothesis was never proven.^45^

The antiplasmodial activity of **4** and epimeric mixtures (**2a+2b** and **3a+3b**) was assayed against the *P. falciparum* 3D7 strain (Table 4). Compounds **1** and **5** could not be tested due to the limited amount of material available. Since compound **4** showed good antiplasmodial inhibitory activity, it was also assayed against a human cell line (HepG2) to determine its cytotoxicity. Compound **4** showed no cytotoxic effect at a concentration as high as 100 μM (IC_50_^HepG2^ > 100 μM), and (+)-(3*S*,4*S*,7*S*,8*S*,9*S*,13*E*,16*S*,18*E*)-cytochalasin Z_27_ (**4**) presents a good selectivity toward the parasite (SI value > 23).

**Table 4:** Antiplasmodial Activity of Compound **4** against a Small Panel of *Plasmodium falciparum*-Resistant Strains (Dd2, TM90C6B, and 3D7r_MMV848)

**Sample**	**IC**_**50**_^**3D7**^**(μM)**^a^	**IC**_**50**_^**Dd2**^**(μM)**^a^	**RI**	**IC**_**50**_^**TM90C6B**^**(μM)**^a^	**RI**	**IC**_**50**_^**3D7r_MMV848**^**(μM)**^a^	**RI**
**4**	4.4 ± 0.7	7 ± 3	1.6	6 ± 2	1.4	4 ± 1	0.9
**PYR**	0.06 ± 0.01	>10	>166	NT	-	NT	-
**ATV**	0.0007 ± 0.0003	NT	-	>1	>1428	NT	-
**MMV848**	0.12 ± 0.02	NT	-	NT	-	1.5 ± 0.4	12.5
**ART**	0.006 ± 0.002	0.011 ± 0.004	2	0.006 ± 0.001	1	0.013 ± 0.004	2

a Data represent mean ± standard deviation of two independent experiments conducted in triplicate; RI = IC_50_^resistant strain^/IC_50_^3D7^; NT = not tested.

Metabolite **4** was additionally tested against a small panel of resistant *P. falciparum* strains (Table 4), including Dd2 [resistant to chloroquine (CQ), sulfadoxine (SDX), pyrimethamine (PYR), mefloquine (MQ), cycloguanil (CYC)], TM90C6B [resistant to CQ, PYR, atovaquone (ATO)], and 3D7r_MMV848 (resistant to MMV692848) strains.^46^ Compound **4** showed comparable inhibitory activities against the resistant strains assessed, providing resistant index (RI) values lower than 2. This finding indicated that metabolite **4** demonstrated no cross-resistance with the standard antimalarials used as controls for each resistant strain.

## Experimental Section

### General Experimental Procedures

Optical rotations were measured on a Jasco P-2000 polarimeter. NMR experiments were recorded on a Bruker Avance III instrument (14.1 T) with an inverse cryoprobe of 5 mm with a z-field gradient. The nondeuterated residual solvent signal was used as a reference. Electronic circular dichroism and UV spectra were measured on a JASCO J-815 spectropolarimeter in the 200–400 nm region using quartz cells (0.1 cm path length) at 25 °C, bandwidth 1 nm, scanning speed 100 nm/min, 10 accumulations, and samples in MeOH solution (0.15 mg/mL for **1** and **2a**+**2b** and 0.075 mg/mL for **3a**+**3b**). The analytical HPLC-UV-MS system was a Waters instrument (2695 Alliance separation module and 2696 photodiode array detector) coupled to a Waters Micromass ZQ 2000 mass spectrometer. Analyses were performed using a Waters XTerra RP_18_ column (250 mm × 4.6 mm, 5 μm) along with the RP_18_ protective guard column (4 × 3 mm) and eluting with H_2_O + 0.1% formic acid, MeOH + 0.1% formic acid, and MeCN + 0.1% formic acid using a gradient from 90:5:5 to 0:50:50 of H_2_O/MeOH/MeCN over 22 min, maintaining in 0:50:50 H_2_O/MeOH/MeCN for 8 min, from 0:50:50 to 90:5:5 in 1 min, and maintaining at 90:5:5 for 9 min, using a flow rate of 1.0 mL/min. The PDA detector scanned between λ 200 and 600 nm. The mass spectrometer detector was optimized using the following conditions: capillary voltage 3 kV; temperature of the source 100 °C; desolvation temperature 350 °C; ESI mode, acquisition range 100 to 1200 Da; gas flow without cone 50 L/h; and desolvation gas flow 350 L/h. Samples were diluted in MeOH at a concentration of 1 mg/mL. Semipreparative HPLC separations were performed on a Waters system, including a Waters 600 quaternary pump and a Waters 2996 controller coupled to a Waters 2487 dual absorbance detector using a Waters XTerra RP_18_ column (150.0 mm × 7.8 mm, 5 μm) and a Phenomenex PFP column (250 mm × 4.6 mm, 5 μm), both with the RP_18_ protective guard column (4 × 3 mm) and a flow rate of 2.5 mL/min and 1.0 mL/min, respectively. Another analytical HPLC-PDA-MS system used was a Shimadzu instrument (LC2030C 3D Plus Prominence) coupled to a Shimadzu LCMS-2020 mass spectrometer. Analyses were performed using a Phenomenex Kinetex RP_18_ column (100 mm × 4.6 mm i.d., 2.6 μm) along with the Security Guard RP_18_ protective guard column (4.6 mm i.d.) and eluting with H_2_O + 0.1% formic acid and MeCN + 0.1% formic acid using a gradient from 90:10 to 10:90 of H_2_O/MeCN over 15 min, maintaining in 10:90 H_2_O/MeCN for 3 min, from 10:90 to 90:10 in 1 min, and maintaining at 90:10 for 1 min, using a flow rate of 1.0 mL/min. The PDA detector scanned between λ = 190 and 700 nm. The MS was optimized using the following conditions: interface voltage, 4.5 kV; interface temperature, 350 °C; DL temperature, 250 °C; heat block, 200 °C; ESI mode, acquisition range, 100 to 1000 Da; nebulizing gas, 1.5 L/min; drying gas flow, 15 L/min. UPLC-QToF-HRMS analyses were performed on a Waters Acquity H-Class UPLC instrument coupled to a Xevo G2-XS Q-TOF instrument with an electrospray ionization (ESI) interface. The chromatographic separation was performed using a Waters Acquity UPLC BEH column (RP_18_, 2.1 × 100 mm, 1.7 μm), with a mobile phase composed of H_2_O + 0.1% formic acid (A) and MeCN + 0.1% formic acid (B). The following gradient was applied at a flow rate of 0.5 mL/min: 10% to 100% B in 8 min, maintaining the gradient condition at 100% B for 0.2 min, then setting it back to 10% B at 8.20 min, and keeping it at 10% B until *t* = 10 min. The HRESIMS data were acquired in positive and negative ion mode. HPLC-grade organic solvents and HPLC-Milli-Q-grade water were filtered prior to use. Deuterated NMR solvents (MeOH-*d*_4_ and DMSO-*d*_6_) were purchased from Cambridge Isotope Laboratories. [^15^N]-Glycine, [^15^N]-hydroxylamine, and [^2^H]-hydroxylamine were purchased from Sigma-Aldrich-Merck.

### Fungus Strain Isolation and Identification

The marine fungus *Peroneutypa* sp. M16 was isolated and identified as previously reported.^24^ Sampling permit: SisGen code (A840479).

### Fungal Genomic DNA Extraction, Sequencing, Annotation, and Analysis

*Peroneutypa* sp. M16 was cultured in liquid PDB at 28 °C with shaking at 50 rpm for 7 days and then filtered under vacuum until cells were dry. Genomic DNA was prepared following the hexadecyltrimethylammonium bromide (CTAB) protocol^47^ and checked for integrity using Tapestation. Genome sequencing was performed at UNT’s Health Science Center Genomics Core using Illumina sequencing, resulting in 5,205,570 reads with an average of 27× and maximum of 33× coverage. The genome was assembled using SPAdes, resulting in a genome size of 50 Mbp and 863 scaffolds with N50 = 113 kbp.^48^ Secondary metabolite gene clusters were preliminary identified using antiSMASH,^49^ and exon and intron positions were further refined using Softberry FGENESH and AUGUSTUS.^50,51^ Coding sequences were investigated using BLAST, CDD, Phyre2, and AlphaFold.^52−54^ Sequence data were visualized using Geneious.

### Fungus Cultivation and Growth Medium Extraction

The fungus *Peroneutypa* sp. M16 was inoculated and cultivated as previously described.^24^ When cultivated in shaking conditions, 5 plugs (ca. 0.5 cm^2^ each) of the mycelium of the strain were used to inoculate liquid growth media (100 mL of PDB+ASW in 500 mL Schotts flasks; total of 5.4 L) and incubated at 150 rpm and 25 °C. At the seventh day of growth, the glucose strips showed the absence of glucose; then the cultures were filtered and extracted with EtOAc (3×). The combined EtOAc layers were dried (anhydrous Mg_2_SO_4_) and evaporated, yielding an EtOAc extract (838 mg).

### Isolation of Metabolites **1**, **2a**+**2b**, **3a**+**3b**, **4**, **5**, **6**, and **7**

Fractions F2 and F5 from *Peroneutypa* sp. M16 cultures^24^ were subjected to further fractionation steps. Fraction F2 (380.2 mg) was solubilized in 1:1 MeOH/H_2_O (5.0 mL) with sonication and applied to a C_18_ reversed-phase silica-gel cartridge (5 g), eluted with a gradient of MeOH in H_2_O. Four fractions were obtained: F2A (54.7 mg), F2B (129.9 mg), F2C (150.3 mg), and F2D (5.7 mg). Fraction F2C was applied to a C_18_ reversed-phase silica-gel cartridge (5 g) and eluted with a gradient of MeOH in H_2_O. Twenty-three fractions were obtained and analyzed by HPLC-MS, resulting in three combined fractions, F2C-1–8 (113.4 mg), F2C-9–10 (16.4 mg), F2C-11–23 (11.4 mg). Fraction F2C-9–10 was separated by HPLC using a Waters XTerra RP_18_ column (150.0 mm × 7.8 mm, 5 μm) with an isocratic elution using 30:70 MeOH/H_2_O, during 70 min, with a flow rate of 2.5 mL/min and UV detection at λ_max_ 254 nm, resulting in the isolation of the epimeric mixture **2a**+**2b** (3.0 mg), together with subfractions C10A (1.0 mg) and C10C (1.5 mg). These subfractions were purified by HPLC using a Waters PFP reversed-phase silica-gel column (250.0 × 4.6 mm, 5 μm) using an isocratic elution of MeOH/H_2_O (48:52, v:v) during 22 min, with a flow rate of 1.0 mL/min and UV detection at λ_max_ 254 nm, to give **1** (0.3 mg) and **3a**+**3b** (0.6 mg). Fraction F5 was separated by HPLC-UV using a Waters XTerra RP_18_ column (150.0 mm × 7.8 mm, 5 μm) and a UV detector at λ_max_ 254 and 315 nm, eluting with H_2_O + 0.1% formic acid and MeOH + 0.1% formic acid using a gradient from 30:70 to 80:20 of H_2_O/MeOH over 30 min, using a flow rate of 2.5 mL/min, resulting in the isolation of **4** (0.6 mg) and **5** (0.3 mg).

An aliquot of 140 mg from the EtOAc extract obtained from a shaking culture of *Peroneutypa* sp. M16 was subjected to separation on an SPE RP_18_ column (Waters, Oasis HLB SPE). Four subfractions were obtained, of which fraction Fr4 (63 mg) showed the targeted compounds for isolating by LC-MS. An aliquot of 20 mg of Fr4 was subjected to semipreparative HPLC-UV using a Shimadzu Premier RP_18_ column (250 mm × 10 mm, 5 μm) and a UV detector at λ_max_ 254 nm, eluting with MeCN + 0.1% formic acid and H_2_O + 0.1% formic acid using a gradient from 60:40 to 0:100 of H_2_O/MeCN over 20 min, using a flow rate of 3.0 mL/min, yielding the compounds **6** (2.8 mg) and **7** (5.1 mg).

#### (+)-(3*S*,4*S*,5*S*,7*S*,8*S*,9*S*,13*E*,16*S*,18*S*,20*R*)-Perochalasin A (**1**):

colorless amorphous solid; [α]_D_^23^ +77.0 (*c* 0.06; MeOH); UV (MeOH) λ_max_ (log ε) 223 (4.2), 247 (3.7), 271 (3.1) nm; ECD (*c* 0.28 mM, MeOH) λ_max_ (Δε) 230 (−4.54) 267 (+0.96) nm; ^1^H and ^13^C NMR data, see Table 1; HREIMS *m*/*z* 529.2930 [M + H]^+^ (calcd for C_29_H_41_N_2_O_7_^+^, 529.2908).

#### (+)-Perochalasin B (**2a**) + *epi*-perochalasin B (**2b**):

colorless amorphous solid; [α]_D_^23^ +63.5 (*c* 0.27; MeOH); UV (MeOH) λ_max_ (log ε) 223 (4.0), 247 (3.5), 273 (2.9) nm; ECD (*c* 0.28 mM, MeOH) λ_max_ (Δε) 232 (+3.91) nm; ^1^H and ^13^C NMR data, see Table 2; HREIMS *m*/*z* 515.2755 [M + H]^+^ (calcd for C_28_H_39_N_2_O_7_^+^, 515.2752).

#### (+)-Perochalasin C (**3a**) + *epi*-perochalasin C (**3b**):

colorless amorphous solid; [α]_D_^23^ +60.4 (*c* 0.1; MeOH); UV (MeOH) λ_max_ (log ε) 224 (4.3), 247 (3.6), 273 (3.1) nm; ECD (*c* 0.14 mM, MeOH) λ_max_ (Δε) 230 (−1.49) nm; ^1^H and ^13^C NMR data, see Table 3; HREIMS *m*/*z* 515.2759 [M + H]^+^ (calcd for C_28_H_39_N_2_O_7_^+^, 515.2752).

### *In Vitro* Antiplasmodial Assay with *Plasmodium falciparum*

Samples **4**, **2a**+**2b**, and **3a**+**3b** were assayed against the sensitive *P. falciparum* 3D7 strain. In addition, compound **4** was assayed against resistant strains of the parasite (Dd2, TM90C6B, and 3D7r_MMV848). The procedure was previously described.^24^

### The Hepatocarcinoma Cell Culture and Cytotoxic Evaluation

Compound **4** with IC_50_^*Pf*^ < 10 μM had its cytotoxicity evaluated against a human immortalized cell line of hepatocarcinoma cells (HepG2). The procedure was previously described as well.^24^

### *In Vivo* Isotopic Incorporation Experiments

The *Peroneutypa* sp. M16 strain was cultured at 150 rpm and 25 °C using 500 mL Schott flasks containing 100 mL of PDB+ASW medium. [^15^N]-Hydroxylamine and [^15^N]-glycine were dissolved in H_2_O at 50 mg/mL and sterilized by filtration (Nylon 0.45 μm, Fisher Scientific), then added to the growth medium on the fourth day of cultivation at a final concentration of 0.1 mg/mL. Cultures were filtered on the seventh day of growth and extracted with EtOAc, as described above. The incorporation experiments were performed in duplicate. A sample without labeled precursors was grown as a control. The extracts were analyzed by LC-MS (5 μL injected; 5 mg/mL). The isotopic incorporation was determined by MS analysis of the samples by comparison of the values of the ion peaks at *m*/*z* [M + H]^+^, *m*/*z* [M – H_2_O + H]^+^, *m*/*z* [M + 1 + H]^+^, *m*/*z* [M – H_2_O + 1 + H]^+^, and retention time.

### *In Vitro* Isotopic Incorporation Experiments

Phenochalasin B (**7**) isolated from the culture of *Peroneutypa* sp. M16 was solubilized in MeOH/H_2_O (7:3, v/v) and 1% formic acid. This solution was split into three samples: to the first one was added [^15^N]-hydroxylamine; to the second one was added [^2^H]-hydroxylamine, and to the last one, nothing was added and considered as a control. The final concentration of phenochalasin B in the samples as well as for [^15^N]-hydroxylamine and [^2^H]-hydroxylamine was 2 μmol. The samples were analyzed by the LCMS (5 μL injected) after 1 and 6 days of reaction. The isotopic incorporation and the absence of incorporation were determined by MS analysis of the samples by comparison of the values of the ion peaks at *m*/*z* [M + H]^+^, *m*/*z* [M – H_2_O + H]^+^, *m*/*z* [M + 1 + H]^+^, *m*/*z* [M – H_2_O + 1 + H]^+^, and retention time of the standards **1**, **2a**+**2b**, and **3a**+**3b**.

### ^13^C NMR Chemical Shifts and ECD Calculations

The conformational searches of **1** were carried out at the molecular mechanics level of theory by employing either the MM+ or MMFF force fields incorporated in HyperChem 8.0.10 and Spartan 08 software packages, respectively. The DFT and TDDFT calculations were carried out at 298 K in either gas phase or MeOH or DMSO solutions using the polarizable continuum model (PCM) in its integral equation formalism version (IEFPCM), incorporated in Gaussian 09 software.^55^ The configurations 3*S*,4*S*,5*S*,7*S*,8*S*,9*S*,13*E*,16*S*,18*S*,20*R*, 3*S*,4*S*,5*S*,7*S*,8*S*,9*S*,13*E*,16*R*,18*S*,20*R*, 3*S*,4*S*,5*S*,7*S*,8*S*,9*S*,13*E*,16*S*,18*R*,20*S*, and 3*S*,4*S*,5*S*,7*S*,8*S*,9*S*,13*E*,16*R*,18*R*,20*S* were arbitrarily chosen for the calculations. The chiroptical properties of their enantiomers were obtained by multiplying the calculated data by −1. Initially, all 100 conformers identified within a 10 kcal/mol energy window for each configuration were kept, and geometry was optimized at the B3LYP/6-31G(d) level. The lowest-energy conformers with relative energy (rel *E*) ≤ 2.0 kcal/mol were then selected for ^13^C NMR and UV/ECD calculations. The ^13^C NMR shielding constants (*s*) of the stereoisomers of **1** were calculated using a gauge-including atomic orbital–hybrid density functional theory (GIAO–HDFT) calculation procedure at the mPW1PW91/PCM(DMSO)/6-31+G(d,p) level of theory. ^13^C NMR chemical shifts were calculated as δ_calc_ = σ_ref_ – σ, where σ_ref_ is the shielding constant of TMS predicted at the same level of theory and plotted as Boltzmann averages of the lowest-energy conformers identified (B3LYP/631G(d) zero-point-corrected electronic energies). DP4+ analyses were obtained as described.^29^ UV and ECD spectra were calculated at the CAM-B3LYP/PCM(MeOH)/TZVP level. Vibrational analysis at the level used for geometry optimizations resulted in no imaginary frequencies for all conformers, confirming them as real minima. TDDFT was employed to calculate the excitation energy (in nm) and rotatory strength *R* in the dipole velocity (*R*_vel_ in cgs units: 10^–40^ esu^2^ cm^2^) form. The calculated rotatory strengths from the first 30 singlet → singlet electronic transitions were simulated into an ECD curve using Gaussian bands with an HWHM of σ 0.25 eV. The predicted wavelength transitions were multiplied with a scaling factor of 1.05, determined by the best agreement between the experimental and calculated UV spectra, and the composite spectra were plotted as Boltzmann averages (electronic energies).

## Data Availability

Raw NMR data has been provided within the NP-MRD platform. NP-MRD Deposit data for the new compounds: perochalasin A NP-Card ID: NP0333714; perochalasin B + *epi*-perochalasin B NP-Card ID: NP0333715; perochalasin C + *epi*-perochalasin C NP-Card ID: NP0333716.

## Supporting Information Available

Spectroscopic and spectrometric data of **1**, **2a+2b**, and **3a+3b**; atomic coordinates for the stereoisomers of **1**; procedures of fungal gDNA extraction and purification; procedures of whole genome sequencing preparation and analysis; bioinformatic data analyses of the biosynthetic gene clusters (PDF)

## References

[ref1] Garcia K. Y. M.; Quimque M. T. J.; Lambert C.; Schmidt K.; Primahana G.; Stradal T. E. B.; Ratzenböck A.; Dahse H. M.; Phukhamsakda C.; Stadler M.; Surup F.; Macabeo A. P. G. J. Fungi 2022, 8, 560 10.3390/jof8060560. PMC9225350 35736043

[ref2] Kumarihamy M.; Ferreira D.; Croom E. M.; Sahu R.; Tekwani B. L.; Duke S. O.; Khan S.; Techen N.; Dhammika Nanayakkara N. P. Molecules 2019, 24, 777 10.3390/molecules24040777. 30795572 PMC6413121

[ref3] Hu X. Y.; Wang C. Y.; Li X. M.; Yang S. Q.; Li X.; Wang B. G.; Si S. Y.; Meng L. H. J. Nat. Prod. 2021, 84, 3122–3130. 10.1021/acs.jnatprod.1c00907. 34846891

[ref4] Chen T. S.; Doss G. A.; Hsu A.; Hsu A.; Lingham R. B.; White R. F.; Monaghan R. L. J. Nat. Prod. 1993, 56, 755–761. 10.1021/np50095a013. 8326323

[ref5] Wang J. F.; Huang R.; Liu S. S.; Wu S. H. Chem. Nat. Compd. 2021, 57, 1169–1174. 10.1007/s10600-021-03579-5.

[ref6] Skellam E. Nat. Prod. Rep. 2017, 34, 1252–1263. 10.1039/C7NP00036G. 28849835

[ref7] Zhu H.; Chen C.; Tong Q.; Zhou Y.; Ye Y.; Gu L.; Zhang Y. In Progress in the Chemistry of Organic Natural Products; Progress in the Chemistry of Cytochalasans; Kinghorn A. D.; Falk H.; Gibbons S.; Kobayashi J.; Asakawa Y.; Liu J.-K., Eds.; Springer Nature Switzerland AG: Cham, Switzerland, 2021; pp 1–134. 10.1007/978-3-030-59444-2_1 33792860

[ref8] Qiao K.; Chooi Y. H.; Tang Y. Metab. Eng. 2011, 13, 723–732. 10.1016/j.ymben.2011.09.008. 21983160 PMC3254600

[ref9] Fujii R.; Minami A.; Gomi K.; Oikawa H. Tetrahedron Lett. 2013, 54, 2999–3002. 10.1016/j.tetlet.2013.03.120.

[ref10] Song Z.; Bakeer W.; Marshall J. W.; Yakasai A. A.; Khalid R. M.; Collemare J.; Skellam E.; Tharreau D.; Lebrun M. H.; Lazarus C. M.; Bailey A. M.; Simpson T. J.; Cox R. J. Chem. Sci. 2015, 6, 4837–4845. 10.1039/C4SC03707C. 29142718 PMC5667575

[ref11] Nielsen M. L.; Isbrandt T.; Petersen L. M.; Mortensen U. H.; Andersen M. R.; Hoof J. B.; Larsen T. O. PLoS One 2016, 11, 1–18. 10.1371/journal.pone.0161199. PMC4994942 27551732

[ref12] Zhang H.; Hantke V.; Bruhnke P.; Skellam E. J.; Cox R. J. Chem. - A Eur. J. 2021, 27, 3106–3113. 10.1002/chem.202004444. PMC7898483 33146923

[ref13] Hantke V.; Skellam E. J.; Cox R. J. Chem. Commun. 2020, 56, 2925–2928. 10.1039/C9CC09590J. 32039410

[ref14] Wang C.; Becker K.; Pfütze S.; Kuhnert E.; Stadler M.; Cox R. J.; Skellam E. Org. Lett. 2019, 21, 8756–8760. 10.1021/acs.orglett.9b03372. 31644300

[ref15] Ishiuchi K.; Nakazawa T.; Yagishita F.; Mino T.; Noguchi H.; Hotta K.; Watanabe K. J. Am. Chem. Soc. 2013, 135, 7371–7377. 10.1021/ja402828w. 23611317

[ref16] Zhang J. M.; Liu X.; Wei Q.; Ma C.; Li D.; Zou Y. Nat. Commun. 2022, 13, 225 10.1038/s41467-021-27931-z. 35017571 PMC8752850

[ref17] Hu Y.; Dietrich D.; Xu W.; Patel A.; Thuss J. A. J.; Wang J.; Yin W. B.; Qiao K.; Houk K. N.; Vederas J. C.; Tang Y. Nat. Chem. Biol. 2014, 10, 552–554. 10.1038/nchembio.1527. 24838010 PMC4062580

[ref18] Wang C.; Hantke V.; Cox R. J.; Skellam E. Org. Lett. 2019, 21, 4163–4167. 10.1021/acs.orglett.9b01344. 31099577

[ref19] Zhu H.; Chen C.; Tong Q.; Yang J.; Wei G.; Xue Y.; Wang J.; Luo Z.; Zhang Y. Angew. Chem. 2017, 56, 5242–5246. 10.1002/anie.201701125. 28378450

[ref20] Wu Z.; Zhang X.; Chen C.; Zhou P.; Zhang M.; Gu L.; Luo Z.; Wang J.; Tong Q.; Zhu H.; Zhang Y. Org. Lett. 2020, 22, 2162–2166. 10.1021/acs.orglett.0c00141. 32129633

[ref21] Wang W.; Zeng F.; Bie Q.; Dai C.; Chen C.; Tong Q.; Liu J.; Wang J.; Zhou Y.; Zhu H.; Zhang Y. Org. Lett. 2018, 20, 6817–6821. 10.1021/acs.orglett.8b02942. 30350674

[ref22] Chen C.; Zhu H.; Li X. N.; Yang J.; Wang J.; Li G.; Li Y.; Tong Q.; Yao G.; Luo Z.; Xue Y.; Zhang Y. Org. Lett. 2015, 17, 644–647. 10.1021/ol503666b. 25615686

[ref23] Kemkuignou B. M.; Lambert C.; Schmidt K.; Schweizer L.; Anoumedem E. G. M.; Kouam S. F.; Stadler M.; Stradal T.; Marin-Felix Y. Fitoterapia 2023, 166, 105434 10.1016/j.fitote.2023.105434. 36681097

[ref24] Amorim M. R.; Barbosa C. S.; Paz T. A.; Laura P. I.; Nicacio K. J.; Oliveira L. F. P.; Goulart M. O.; Paulino J. M.; Cruz M. O.; Ferreira A. G.; Furlan M.; Lira S. P.; Santos R. A.; Rodrigues A.; Guido R. V. C.; Berlinck R. G. S. J. Nat. Prod. 2023, 86, 1476–1486. 10.1021/acs.jnatprod.3c00175. 37289832

[ref25] Zhang Q.; Xiao J.; Sun Q.-Q.; Qin J.-C.; Pescitelli G.; Gao J.-M. J. Agric. Food Chem. 2014, 62, 10962–10969. 10.1021/jf503846z. 25350301

[ref26] Liu R.; Lin Z.; Zhu T.; Fang Y.; Gu Q.; Zhu W. J. Nat. Prod. 2008, 71, 1127–1132. 10.1021/np070539b. 18507474

[ref27] Tomoda H.; Namatame I.; Tabata N.; Kawaguchi K.; Si S.; Omura S. J. Antibiot. 1999, 52, 857–861. 10.7164/antibiotics.52.857. 10604754

[ref28] Huang L. L.; Xu Z. L.; Liang M.; Lv L. X.; Li B. C.; Qin X. Y.; Huang X. S.; Li J.; Xu W. F.; Yang R. Y. Chem. Nat. Compd. 2023, 59, 197–200. 10.1007/s10600-023-03953-5.

[ref29] Zanardi M. M.; Sarotti A. M. J. Org. Chem. 2021, 86, 8544–8548. 10.1021/acs.joc.1c00987. 34101443

[ref30] Kongprapan T.; Rukachaisirikul V.; Saithong S.; Phongpaichit S.; Poonsuwan W.; Sakayaroj J. Phytochem. Lett. 2015, 13, 171–176. 10.1016/j.phytol.2015.06.010.

[ref31] Blin K.; Shaw S.; Steinke K.; Villebro R.; Ziemert N.; Lee S. Y.; Medema M. H.; Weber T. Nucleic Acids Res. 2019, 47, W81–W87. 10.1093/nar/gkz310. 31032519 PMC6602434

[ref32] Moore G. G.; Collemare J.; Lebrun M. H.; Bradshaw R. E. In Natural Products; Evolutionary Mechanisms Involved in Development of Fungal Secondary Metabolite Gene Clusters; Osbourn A.; Goss R. J.; Carter G. T., Eds.; John Wiley & Sons, Inc.: Hoboken, NJ, 2014; pp 341–356.

[ref33] Heard S. C.; Wu G.; Winter J. M. J. Antibiot. 2020, 73, 803–807. 10.1038/s41429-020-00368-0. 32913332

[ref34] Li H.; Wei H.; Hu J.; Lacey E.; Sobolev A. N.; Stubbs K. A.; Solomon P. S.; Chooi Y. H. ACS Chem. Biol. 2020, 15, 226–233. 10.1021/acschembio.9b00791. 31815421

[ref35] Gilchrist C. L. M.; Chooi Y. H. Bioinformatics 2021, 37, 2473–2475. 10.1093/bioinformatics/btab007. 33459763

[ref36] Kelly W. L.; Townsend C. A. J. Am. Chem. Soc. 2002, 124, 8186–8187. 10.1021/ja025926g. 12105888

[ref37] Townsend C. A.; Salituro G. M. J. Chem. Soc., Chem. Commun. 1984, 1631–1632. 10.1039/c39840001631.

[ref38] Chen H.-P.; Zhao Z.-Z.; Li Z.-H.; Dong Z.-J.; Wei K.; Bai X.; Zhang L.; Wen C.-N.; Feng T.; Liu J.-K. ChemistryOpen 2016, 5, 142–149. 10.1002/open.201500198. 27308232 PMC4906468

[ref39] Patteson J. B.; Putz A. T.; Tao L.; Simke W. C.; Bryant L. H.; Britt R. D.; Li B. Science 2021, 374, 1005–1009. 10.1126/science.abj6749. 34793213 PMC8939262

[ref40] Kasahara K.; Miyamoto T.; Fujimoto T.; Oguri H.; Tokiwano T.; Oikawa H.; Ebizuka Y.; Fujii I. ChemBioChem. 2010, 11, 1245–1252. 10.1002/cbic.201000173. 20486243

[ref41] Pajares S.; Ramos R. Front. Mar. Sci. 2019, 6, 739 10.3389/fmars.2019.00739.

[ref42] Gao W.; Jiang R.; Zeng H.; Cao J.; Hu Z.; Zhang Y. Nat. Prod. Res. 2024, 38, 1599–1605. 10.1080/14786419.2022.2150846. 36441184

[ref43] Chen C.; Wang J.; Liu J.; Zhu H.; Sun B.; Wang J.; Zhang J.; Luo Z.; Yao G.; Xue Y.; Zhang Y. J. Nat. Prod. 2015, 78, 1193–1201. 10.1021/np500626x. 26068802

[ref44] Qi S.; Wang Y.; Zheng Z.; Qingyan X. U.; Deng X. Nat. Prod. Commun. 2015, 10, 2027–2030. 10.1177/1934578X1501001203. 26882656

[ref45] Tang J.-W.; Hu K.; Su X.-Z.; Li X.-N.; Yan B.-C.; Sun H.-D.; Puno P.-T. Tetrahedron 2020, 76, 131475 10.1016/j.tet.2020.131475.

[ref46] Irabuena C.; Scarone L.; de Souza G. E.; Aguiar A. C. C.; Mendes G. R.; Guido R. V. C.; Serra G. Med. Chem. Res. 2022, 31, 426–435. 10.1007/s00044-021-02843-1. 35106047 PMC8794615

[ref47] Conlon B. H.; Schmidt S.; Poulsen M.; Shik J. Z. STAR Protoc. 2022, 3, 101126 10.1016/j.xpro.2022.101126. 35112085 PMC8790498

[ref48] Prjibelski A.; Antipov D.; Meleshko D.; Lapidus A.; Korobeynikov A. Curr. Protoc. Bioinformatics 2020, 70, e102 10.1002/cpbi.102. 32559359

[ref49] Blin K.; Shaw S.; Kloosterman A. M.; Charlop-Powers Z.; Van Wezel G. P.; Medema M. H.; Weber T. Nucleic Acids Res. 2021, 49, W29–W35. 10.1093/nar/gkab335. 33978755 PMC8262755

[ref50] Solovyev V.; Kosarev P.; Seledsov I.; Vorobyev D. Genome Biol. 2006, 7, S10 10.1186/gb-2006-7-s1-s10. PMC1810547 16925832

[ref51] Keller O.; Kollmar M.; Stanke M.; Waack S. Bioinformatics 2011, 27, 757–763. 10.1093/bioinformatics/btr010. 21216780

[ref52] Madeira F.; Pearce M.; Tivey A. R. N.; Basutkar P.; Lee J.; Edbali O.; Madhusoodanan N.; Kolesnikov A.; Lopez R. Nucleic Acids Res. 2022, 50, W276–W279. 10.1093/nar/gkac240. 35412617 PMC9252731

[ref53] Kelley L. A.; Mezulis S.; Yates C. M.; Wass M. N.; Sternberg M. J. E. Nat. Protoc. 2015, 10, 845–858. 10.1038/nprot.2015.053. 25950237 PMC5298202

[ref54] Marchler-Bauer A.; Anderson J. B.; Chitsaz F.; Derbyshire M. K.; DeWeese-Scott C.; Fong J. H.; Geer L. Y.; Geer R. C.; Gonzales N. R.; Gwadz M.; He S.; Hurwitz D. I.; Jackson J. D.; Ke Z.; Lanczycki C. J.; Liebert C. A.; Liu C.; Lu F.; Lu S.; Marchler G. H.; Mullokandov M.; Song J. S.; Tasneem A.; Thanki N.; Yamashita R. A.; Zhang D.; Zhang N.; Bryant S. H. Nucleic Acids Res. 2009, 37, 205–210. 10.1093/nar/gkn845. PMC2686570 18984618

[ref55] Frisch M. J.; Trucks G. W.; Schlegel H. B.; Scuseria G. E.; Robb M. A.; Cheeseman J. R.; Scalmani G.; Barone V.; Mennucci B.; Petersson G. A.; Nakatsuji H.; Caricato M.; Li X.; Hratchian H. P.; Izmaylov A. F.; Bloino J.; Zheng G.; Sonnenberg J. L.; Hada M.; Ehara M.; Toyota K.; Fukuda R.; Hasegawa J.; Ishida M.; Nakajima T.; Honda Y.; Kitao O.; Nakai H.; Vreven T.; Montgomery J. A.; Peralta J. E.; Ogliaro F.; Bearpark M.; Heyd J. J.; Brothers E.; Kudin K. N.; Staroverov V. N.; Kobayashi R.; Normand J.; Raghavachari K.; Rendell A.; Burant J. C.; Iyengar S. S.; Tomasi J.; Cossi M.; Rega N.; Millam J. M.; Klene M.; Knox J. E.; Cross J. B.; Bakken V.; Adamo C.; Jaramillo J.; Gomperts R.; Stratmann R. E.; Yazyev O.; Austin A. J.; Cammi R.; Pomelli C.; Ochterski J. W.; Martin R. L.; Morokuma K.; Zakrzewski V. G.; Voth G. A.; Salvador P.; Dannenberg J. J.; Dapprich S.; Daniels A. D.; Farkas Ö.; Foresman J. B.; Ortiz J. V.; Cioslowski J.; Fox D. J. Gaussian 09, Revision A.02; Gaussian, Inc.: Wallingford, CT, 2009.

