# Supplementary material for: Structure and Biosynthesis of Perochalasins A–C, Open-Chain Merocytochalasans Produced by the Marine-Derived Fungus Peroneutypa sp. M16
Source: J Nat Prod. 2024 Aug 16;87(9):2204–15. doi: 10.1021/acs.jnatprod.4c00516 (PMC11443529; doi:10.1021/acs.jnatprod.4c00516)
Supplement: Supplementary file 1 — np4c00516_si_001.pdf [file np4c00516_si_001.pdf]

Supplementary Information

For

Structure and Biosynthetic Investigations of Perochalasins A–C, Open-Chain Merocytochalasans Produced by the Marine-Derived Fungus *Peroneutypa* sp. M16

Marcelo R. de Amorim,[†]* Sydney M. Schoellhorn,[‡] Camila de S. Barbosa,[§] Giovana R. Mendes,[§] Kamila de L. Macedo,[†] Antonio G. Ferreira,[⊥] Tiago Venâncio,[⊥] Rafael V. C. Guido,[§] Andrea N. L. Batista,[∥] João M. Batista Jr.,[¶] Elizabeth Skellam,[‡]* and Roberto G. S. Berlinck[†]*

[†]Instituto de Química de São Carlos, Universidade de São Paulo, CP 780, CEP 13560-970, São Carlos, SP, Brazil.

[‡]Department of Chemistry and BioDiscovery Institute, University of North Texas, 1155 Union Circle, Denton, TX 76203, USA.

[§]Instituto de Física de São Carlos, Universidade de São Paulo, CEP 13563-120, São Carlos, SP, Brazil.

[⊥]Departamento de Química, Universidade Federal de São Carlos, CEP 13565-905, São Carlos, SP, Brazil.

[∥]Universidade Federal Fluminense, Instituto de Química, Outeiro de São João Batista s/n, Niterói, RJ 24020-141, Brazil.

[¶]Universidade Federal de São Paulo. Instituto de Ciência e Tecnologia, R. Talim 330, São José dos Campos, SP 12231-280, Brazil.

Table of Contents

Experimental

Fungal gDNA extraction and purification S7

Whole Genome Sequencing preparation and analysis S8

List of Supplementary Tables

Table S1. Assembly Statistics………………………………………………………….. S9

Table S2. Assembly Statistics. ………………………………………………………... S9

Table S3. Annotation and comparison of the *sol* biosynthetic gene cluster………………………. S10

Table S4. ^{1}H (500 MHz) and ^{13}C (125 MHz) data of compound **7** in $CDCl_3$ and comparison with phenochalasin B NMR data.….…………………………………………………………S69

Table S5. Results of DP4+ analysis for the correlation between experimental and calculated NMR data of **1**. Isomer 1: 3*S*,4*S*,5*S*,7*S*,8*S*,9*S*,13*E*,16*S*,18*S*,20*R*; Isomer 2: 3*S*,4*S*,5*S*,7*S*,8*S*,9*S*, 13*E*,16*R*,18*S*,20*R*; Isomer 3: 3*S*,4*S*,5*S*,7*S*,8*S*,9*S*,13*E*,16*S*,18*R*,20*S*; Isomer 4: 3*S*,4*S*,5*S*,7*S*,8*S*,9*S*,13*E*,16*R*,18*R*,20*S*..S75

Table S6. Atomic coordinates of the lowest energy conformer of (3*S*,4*S*,5*S*,7*S*,8*S*,9*S*,13*E*,16*S*,18*S*,20*R*)-**1**………………………..………………………………S76

Table S7. Atomic coordinates of the lowest energy conformer of (3*S*,4*S*,5*S*,7*S*,8*S*,9*S*,13*E*,16*R*,18*S*,20*R*)-**1**.……………………………………………..…………S108

Table S8. Atomic coordinates of the lowest energy conformer of (3*S*,4*S*,5*S*,7*S*,8*S*,9*S*,13*E*,16*S*,18*R*,20*S*)-**1**...………………….. …………………………..…..S129

Table S9. Atomic coordinates of the lowest energy conformer of (3*S*,4*S*,5*S*,7*S*,8*S*,9*S*,13*E*,16*R*,18*R*,20*S*)-**1**...……………………. ………………………….……..S133

List of Supplementary Figures

Figure S1. Gel image of *Peroneutypa* sp. M16 gDNA samples. S7

Figure S2. Synteny analysis between the *sol* BGC in *Peroneutypa* sp. M16 to known solanapyrone clusters…… S10

Figure S3. ^{1}H NMR spectrum of perochalasin A (**1**) (600 MHz, DMSO-d_6)...............................S11

Figure S4. Expansion of the ^{1}H NMR spectrum of perochalasin A (**1**) (600 MHz, DMSO-d_6).....S12

Figure S5. Expansion of the ^{1}H NMR spectrum of perochalasin A (**1**) (600 MHz, DMSO-d_6).....S13

Figure S6. Expansion of the ^{1}H NMR spectrum of perochalasin A (**1**) (600 MHz, DMSO-d_6).....S14

Figure S7. Expansion of the ^{1}H NMR spectrum of perochalasin A (**1**) (600 MHz, DMSO-d_6).....S15

Figure S8. ^{13}C NMR spectrum of perochalasin A (**1**) (150 MHz, DMSO-d_6)..............................S16

Figure S9. HSQC spectrum of perochalasin A (**1**) (^{1}H: 600 MHz, ^{13}C: 150 MHz; DMSO-d_6). S17

Figure S10. HMBC spectrum of perochalasin A (**1**) (^{1}H: 600 MHz, ^{13}C: 150 MHz; DMSO-d_6). .S18

Figure S11. COSY spectrum of perochalasin A (**1**) (600 MHz, DMSO-d_6).................................S19

Figure S12. **(+)**-HRESIMS spectrum of perochalasin A (**1**)..S20

Figure S13. 1D-NOESY spectrum of perochalasin A (**1**) (600 MHz, DMSO-d_6). ^{1}H NMR spectrum (**A**), and irradiation at δ_H 2.39 (**B**)...S21

Figure S14. 1D-NOESY spectrum of perochalasin A (**1**) (600 MHz, DMSO-d_6). ^{1}H NMR spectrum (**A**), and irradiation at δ_H 0.78 (**B**)...S22

Figure S15. 1D-NOESY spectrum of perochalasin A (**1**) (600 MHz, DMSO-d_6). ^{1}H NMR spectrum (**A**), and irradiation at δ_H 2.06 (**B**)...S23

Figure S16. 1D-NOESY spectrum of perochalasin A (**1**) (600 MHz, DMSO-d_6). ^{1}H NMR spectrum (**A**), and irradiation at δ_H 3.69 (**B**)...S24

Figure S17. 1D-NOESY spectrum of perochalasin A (**1**) (600 MHz, DMSO-d_6). ^{1}H NMR spectrum (**A**), and irradiation at δ_H 4.95 (**B**)...S25

Figure S18. UV spectrum (150 μg/mL, MeOH) of perochalasin A (**1**)..S26

Figure S19. ECD spectrum (150 μg/mL, MeOH) of perochalasin A (**1**)......................................S27

Figure S20. ^{1}H NMR spectrum of perochalasin B (**2a**) and *epi*-perochalasin B (**2b**) (600 MHz, DMSO-d_6). S28

Figure S21. Expansion of the ^{1}H NMR spectrum of perochalasin B (**2a**) and *epi*-perochalasin B (**2b**) (600 MHz, DMSO-d_6)...S29

Figure S22. Expansion of the ^{1}H NMR spectrum of perochalasin B (**2a**) and *epi*-perochalasin B (**2b**) (600 MHz, DMSO-d_6)...S30

Figure S23. Expansion of the ^{1}H NMR spectrum of perochalasin B (**2a**) and *epi*-perochalasin B (**2b**) (600 MHz, DMSO-d_6)...S31

Figure S24. Expansion of the ^{1}H NMR spectrum of perochalasin B (**2a**) and *epi*-perochalasin B (**2b**) (600 MHz, DMSO-d_6)...S32

Figure S25. ^{13}C NMR spectrum of perochalasin B (**2a**) and *epi*-perochalasin B (**2b**) (150 MHz, DMSO-d_6). S33

Figure S26. HSQC spectrum of perochalasin B (**2a**) and *epi*-perochalasin B (**2b**) (^{1}H: 600 MHz, ^{13}C: 150 MHz; DMSO-d_6)...S34

Figure S27. HMBC spectrum of perochalasin B (**2a**) and *epi*-perochalasin B (**2b**) (^{1}H: 600 MHz, ^{13}C: 150 MHz; DMSO-d_6)...S35

Figure S28. COSY spectrum of perochalasin B (**2a**) and *epi*-perochalasin B (**2b**) (600 MHz, DMSO-d_6).......S36

Figure S29. **(+)**-HRESIMS spectrum of perochalasin B (**2a**) and *epi*-perochalasin B (**2b**).......S37

Figure S30. 1D-NOESY spectrum of perochalasin B (**2a**) and *epi*-perochalasin B (**2b**) (600 MHz, DMSO-d_6). ^{1}H NMR spectrum (**A**), and irradiation at δ_H 5.14 (**B**) and irradiation at δ_H 5.22 (**C**). S38

Figure S31. 1D-NOESY spectrum of perochalasin B (**2a**) and *epi*-perochalasin B (**2b**) (600 MHz, DMSO-d_6). ^{1}H NMR spectrum (**A**), and irradiation at δ_H 4.95 (**B**) and irradiation at δ_H 5.09 (**C**). S39

Figure S32. 2D-NOESY spectrum of perochalasin B (**2a**) and *epi*-perochalasin B (**2b**) (600 MHz, DMSO-d_6).......S40

Figure S33. Expansion of 2D-NOESY spectrum of perochalasin B (**2a**) and *epi*-perochalasin B (**2b**) (600 MHz, DMSO-d_6). NOE interaction of H-3 (δ_H 3.27) with H-13 (δ_H 5.62).......S41

Figure S34. UV spectrum (150 µg/mL, MeOH) of perochalasin B (**2a**) and *epi*-perochalasin B (**2b**)…......S42

Figure S35. ECD spectrum (150 µg/mL, MeOH) of perochalasin B (**2a**) and *epi*-perochalasin B (**2b**)…......S43

Figure S36. ^{1}H NMR spectrum of perochalasin C (**3a**) and *epi*-perochalasin C (**3b**) (600 MHz, DMSO-d_6).......S44

Figure S37. Expansion of the ^{1}H NMR spectrum of perochalasin C (**3a**) and *epi*-perochalasin C (**3b**) (600 MHz, DMSO-d_6).......45

Figure S38. Expansion of the ^{1}H NMR spectrum of perochalasin C (**3a**) and *epi*-perochalasin C (**3b**) (600 MHz, DMSO-d_6).......S46

Figure S39. Expansion of the ^{1}H NMR spectrum of perochalasin C (**3a**) and *epi*-perochalasin C (**3b**) (600 MHz, DMSO-d_6).......S47

Figure S40. ^{13}C NMR spectrum of perochalasin C (**3a**) and *epi*-perochalasin C (**3b**) (150 MHz, DMSO-d_6).......S48

Figure S41. HSQC spectrum of perochalasin C (**3a**) and *epi*-perochalasin C (**3b**) (^{1}H: 600 MHz, ^{13}C: 150 MHz; DMSO-d_6).......S49

Figure S42. HMBC spectrum of perochalasin C (**3a**) and *epi*-perochalasin C (3**b**) (^{1}H: 600 MHz, ^{13}C: 150 MHz; DMSO-d_6).......S50

Figure S43. COSY spectrum of perochalasin C (**3a**) and *epi*-perochalasin C (**3b**) (600 MHz, DMSO-d_6).......S51

Figure S44. **(+)**-HRESIMS spectrum of perochalasin C (**3a**) and *epi*-perochalasin C (**3b**).......S52

Figure S45. 1D-NOESY spectrum of perochalasin C (**3a**) and *epi*-perochalasin C (**3b**) (600 MHz, DMSO-d_6). ^{1}H NMR spectrum (**A**), and irradiation at δ_H 2.40 (**B**).......S53

Figure S46. 1D-NOESY spectrum of perochalasin C (**3a**) and *epi*-perochalasin C (**3b**) (600 MHz, DMSO-d_6). ^{1}H NMR spectrum (**A**), and irradiation at δ_H 3.11 (**B**). S54

Figure S47. 1D-NOESY spectrum of perochalasin C (**3a**) and *epi*-perochalasin C (**3b**) (600 MHz, DMSO-d_6). ^{1}H NMR spectrum (**A**), and irradiation at δ_H 5.12 (**B**) and irradiation at δ_H 4.95 (**C**). S55

Figure S48. UV spectrum (75 µg/mL, MeOH) of perochalasin C (**3a**) and *epi*-perochalasin C (**3b**)…..S56

Figure S49. ECD spectrum (75 µg/mL, MeOH) of perochalasin C (**3a**) and *epi*-perochalasin C (**3b**)…..S57

Figure S50. HPLC-MS analyses of samples from feeding studies of *Peroneutypa* sp. using [^{15}N]-hydroxylamine, as well as the standards **2a+2b** and **3a+3b** for comparison.................................S58

Figure S51. MS spectra of *Peroneutypa* sp. feeding studies using: [^{15}N]-glycine (**B**) and [^{15}N]-hydroxylamine (**C**), as well as the control (**A**). MS spectra from the extracts with the retention time of 7.1 min related to the standard **2a+2b**. S59

Figure S52. MS spectra of *Peroneutypa* sp. feeding studies using: ^{15}N-glycine (**B**) and ^{15}N-hydroxylamine (**C**), as well as the control (**A**). MS spectra from the extracts with the retention time of 6.8 min related to the standard **3a+3b**. S60

Figure S53. MS spectrum of the standard **1** (R_t = 7.2 min) by LC-MS. Positive ionization ESI mode…..61

Figure S54. MS spectrum of the standard **2a+2b** (R_t = 7.1 min) by LC-MS. Positive ionization ESI mode...S61

Figure S55. MS spectrum of the standard **3a+3b** (R_t = 6.8 min) by LC-MS. Positive ionization ESI mode...S62

Figure S56. Chromatogram of the standards **1** (R_t = 7.2 min), **2a+2b** (R_t = 7.1 min) and **3a+3b** (R_t = 6.8 min) by HPLC-PDA monitored at 190 nm. S62

Figure S57. MS spectra of the investigation of phenochalasin B in the presence of [^{15}N]-hydroxylamine and [^{2}H]-hydroxylamine evaluated at 1 and 6 days. Comparison with the standard **3a+3b**. S63

Figure S58. EIC of the investigation of phenochalasin B in the presence of [^{15}N]-hydroxylamine and [^{2}H]-hydroxylamine experiments at 1 and 6 days. MS spectra related of retention time of standard **3a+3b**. S64

Figure S59. MS spectra of the investigation of phenochalasin B in the presence of [^{15}N]-hydroxylamine and [^{2}H]-hydroxylamine evaluated at 1 and 6 days. Comparison with the standard **2a+2b**. S65

Figure S60. EIC of the investigation of phenochalasin B in the presence of [^{15}N]-hydroxylamine and [^{2}H]-hydroxylamine experiments at 1 and 6 days. MS spectra related of retention time of standard **2a+2b**. S66

Figure S61. MS spectra of the investigation of phenochalasin B in the presence of [^{15}N]-hydroxylamine and [^{2}H]-hydroxylamine evaluated at 1 and 6 days. Comparison with the standard **1**…....S67

Figure S62. EIC of the investigation of phenochalasin B in the presence of [^{15}N]-hydroxylamine and [^{2}H]-hydroxylamine experiments at 1 and 6 days. MS spectra related of retention time of standard **1**…....S68

Figure S63. ^{1}H NMR spectrum (500 MHz, $CDCl_3$) of phenochalasin B (**7**). S70

Figure S64. Expansion of ^{1}H NMR spectrum (500 MHz, $CDCl_3$) of phenochalasin B (**7**)....S71

Figure S65. Expansion of ^{1}H NMR spectrum (500 MHz, $CDCl_3$) of phenochalasin B (**7**)....S72

Figure S66. ^{13}C NMR spectrum (125 MHz, $CDCl_3$) of phenochalasin B (**7**). S73

Scheme S1. Proposed mechanism for the non-enzymatic transformations resulting in the compounds **1-3**……S74

Fungal gDNA extraction and purification

Genomic DNA (gDNA) extraction was performed *via* the hexadecyltrimethylammonium bromide (CTAB) protocol.[1] Approximately 50 – 75 mg of *Peroneutypa* sp. M16 mycelium was collected from agar in a Petri dish and homogenized with a micropestle and 200 µL of CTAB buffer. Proteinase K, RNase *α,β*-mercaptoethanol (7µL each) and 500 µL CTAB buffer were added to the sample and left to incubate at 65 ºC and 50 rpm for three hours. The samples were then centrifuged at 20,000 x *g* and 20 ºC for 5 min and the supernatant was collected in a new tube. Subsequently, 700 µL of a phenol-chloroform-isoamyl alcohol (25:24:1) solution was added, inverted 100 times, and incubated at room temperature for 5 min. This solution was then centrifuged at 20,000 x *g* and 20 ºC for 30 min and the upper phase was carefully collected in a new tube, avoiding the white interphase. Further 700 µL of a chloroform-isoamyl alcohol (24:1) solution was added, inverted 100 times, and incubated at room temperature for 5 min followed by centrifugation at 20,000 x *g* and 20 ºC for 15 min. The upper phase was carefully collected in a new tube while avoiding the white interphase. The volume of the collected supernatant was measured. An equivolume solution of 1:2 of 5 M NaCl and ice-cold isopropanol was added to the supernatant. This solution was inverted 20 times and centrifuged at 20,000 x *g* and 20 ºC for 30 min. The supernatant was carefully discarded while being cautious to not disturb the pellet. 750 µL ice-cold 70% EtOH was added followed by centrifugation at 20,000 x *g* and 20 ºC for 3 min and then repeated for a total of two EtOH washes. The supernatant was carefully discarded, again avoiding disturbing the pellet, and the pellet was left to air dry for 10 minutes at room temperature. 50 µL of TE buffer was added and the sample dissolved at 4 ˚C for 12 h, imaged (Figure S1), then stored at -20 ºC.

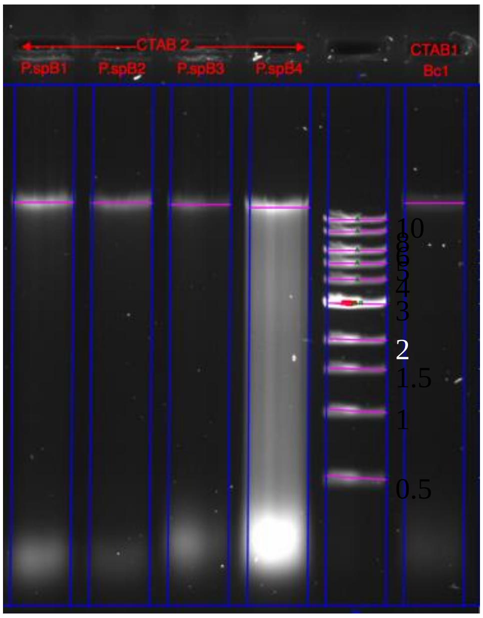

Figure S1. Gel image of *Peroneutypa* sp. M16 gDNA samples.

As some unwanted fragments were visible in the remaining lanes of the gel, fungal gDNA was purified using the Beckman AMPure XP cleanup kit by Beckman Coulter. Briefly, samples were combined with the 0.9 x blue reagent beads at a 1:1 ratio and incubated with gentle shaking. A magnet was then applied to capture the beads and discard the solution. Two washes with freshly prepared 80% EtOH were performed in the same manner before 1 mL of elution buffer (EB) was added to separate the DNA from the magnetic beads. The sample was once again magnetized to collect the DNA in EB.

(1) Conlon, B. H.; Schmidt, S.; Poulsen, M.; Shik, J. Z. *STAR Protoc.* **2022**, *3* (1), 101126.

Whole Genome Sequencing preparation and analysis

Libraries were prepared using the cleaned gDNA. Library preparation was completed according to the Illumina DNA Prep Reference Guide[2] for tagmentation, post-tagmentation cleanup, amplification of tagmented DNA, and cleaning up of libraries. This was performed on a total of six gDNA samples, resulting in the availability of six libraries for sequencing.

The whole genome sequence (WGS) run was performed on an Illumina MiSeq v3 (2x300) to produce up to 25 M paired reads at UNT's Genomics Center. Each library was run individually, resulting in six average coverages of 33.7781X, 31.5618X, 28.4983X, 25.875X, 23.387X, and 20.3844X. To produce the best possible results, all data was used for alignment regardless of coverage. Prior to alignment, data was cleaned using Geneious™ software. Default parameters for trimming reads, merging paired reads, removing duplicates, and error correction and normalization were used. Assembly was performed using SPAdes assembler 3.15.2[3] with 5,205,570 reads assembled to produce 1,094 contigs contained within 863 scaffolds. Statistics of this run are available in Table S1.

(2) Illumina. *Illumina DNA Prep Reference Guide*; Document No. 1000000025416v10; San Diego, CA, 2021.
(3) Prjibelski, A.; Antipov, D.; Meleshko, D.; Lapidus, A.; Korobeynikov, A. *Curr. Protoc. Bioinformatics* **2020**, *70*, e102.

Table S1. Assembly Statistics.

Statistics	All Contigs	Contigs ≥ 100 bp	Contigs ≥1000 bp	All Scaffolds	Scaffolds ≥100 bp	Scaffolds ≥1000 bp
**Count**	1,094	1,094	871	863	863	642
**Min Length (bp)**	128	128	1,001	128	128	1,001
**Median Length (bp)**	20,253	20,253	33,442	21,551	21,551	47,702
**Mean Length (bp)**	44,730	44,730	56,084	56,742	56,742	76,145
**Max Length (bp)**	469,099	469,099	469,099	683,096	683,096	683,096
**N50 Length (bp)**	113,003	113,003	113,003	146,156	146,156	146,156
**Number ≥ N50**	140	140	140	107	107	107
Length Sum (bp)	48,935,139	48,935,139	48,849,985	48,969,078	48,969,078	48,885,613

Table S2. Annotation and comparison of the *pero* biosynthetic gene cluster

Gene name	Protein name	Putative Function	Homolog	Identity (%)	Query Cover (%)	E-value	Score
		phenylalanine and histidine ammonia-lyase	GGS23DRAFT_615957	70.35%	99%	0	1045
		dimethylallyltransferase domain-containing protein	F4779DRAFT_578516	56.87%	99%	0	551
		transposase	FLAG1_07870	45.19%	75%	2e-28	113
peroB	PeroB	Baeyer–Villiger Monooxygenase	ACLA_078650	56.78%	98%	0	732
peroT	PeroT	Major Facilitator Superfamily Transporter	MGG_08384	39.53%	95%	7e-140	422
peroR	PeroR	Transcriptional Regulator	ACLA_078640	34.08%	93%	4e-49	170
peroA	PeroA	*O*-methyltransferase	BP5553_08684	70.45%	99%	1e-137	399
peroX	PeroX	Thioredoxin	ACLA_078720	50.43%	96%	4e-76	240
peroG	PeroG	Cytochrome P450	ACLA_078710	52.32%	98%	0	539
peroC	PeroC	Trans-enoyl reductase	ACLA_078700	68.80%	98%	2e-178	509
peroF	PeroF	Diels-Alderase	ACLA_078690	74.37%	99%	0	548
peroE	PeroE	*α,β*-hydrolase	ACLA_078680	70.29%	98%	0	647
peroD	PeroD	Cytochrome P450	ACLA_078670	67.88%	92%	0	669
peroS	PeroS	PKS-NRPS	ACLA_078660	57.59%	99%	0	4730
		unknown					
		kinase	F4775DRAFT_568542	70.65%	98%	0	825
		cyanate hydratase	F5Y19DRAFT_450473	77.47%	100%	9e-95	283

Table S3. Annotation and comparison of the *sol* biosynthetic gene cluster

Gene Name	Protein Name	Putative Function	Homolog	Identity (%)	Query Cover (%)	E-value	Score
		forkhead box protein L2	F5B20DRAFT_559662	67.31%	100%	0	970
		DUF1649-domain-containing protein	F4859DRAFT_314176	69.37%	81%	3e-102	307
sol1	Sol1	Prosolanapyrone synthase	SOL1_ALTSO	56.27%	98%	0	3007
sol2	Sol2	*O*-methyltransferase	SOL2_ALTSO	62.72%	96%	0	547
sol3	Sol3	Short chain dehydrogenase	SOL3_ALTSO	65.51%	87%	2e-122	363
sol4	Sol4	Probable transcription factor	SOL4_ALTSO	42.00%	83%	3e-120	377
sol6	Sol6	Cytochrome P450 monooxygenase	SOL6_ALTSO	49.23%	80%	3e-119	365
sol5	Sol5	FAD-dependent monooxygenase	SOL5_ALTSO	52.12%	99%	0	539
		Peroxin-3	GGR52DRAFT_6215	62.97%	99%	0	714
		snare-like protein	GGS24DRAFT_232246	65.09%	100%	1e-91	276
		Pkinase domain-containing protein	F4820DRAFT_420933	52.43%	91%	0	658

Figure S2. Synteny analysis between the *sol* BGC in *Peroneutypa* sp. M16 to known solanapyrone clusters.

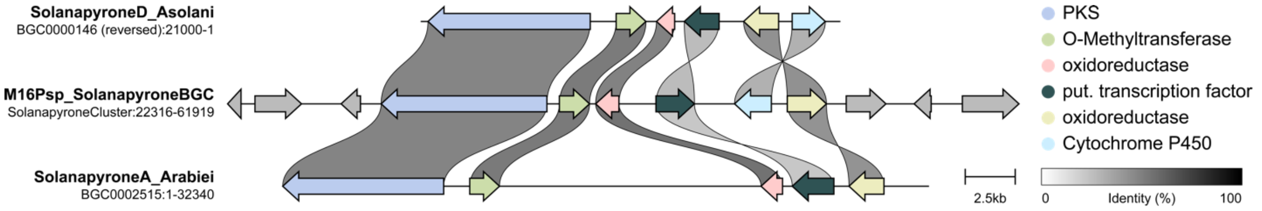

Figure S3. ^{1}H NMR spectrum of perochalasin A (**1**) (600 MHz, DMSO-d_6).

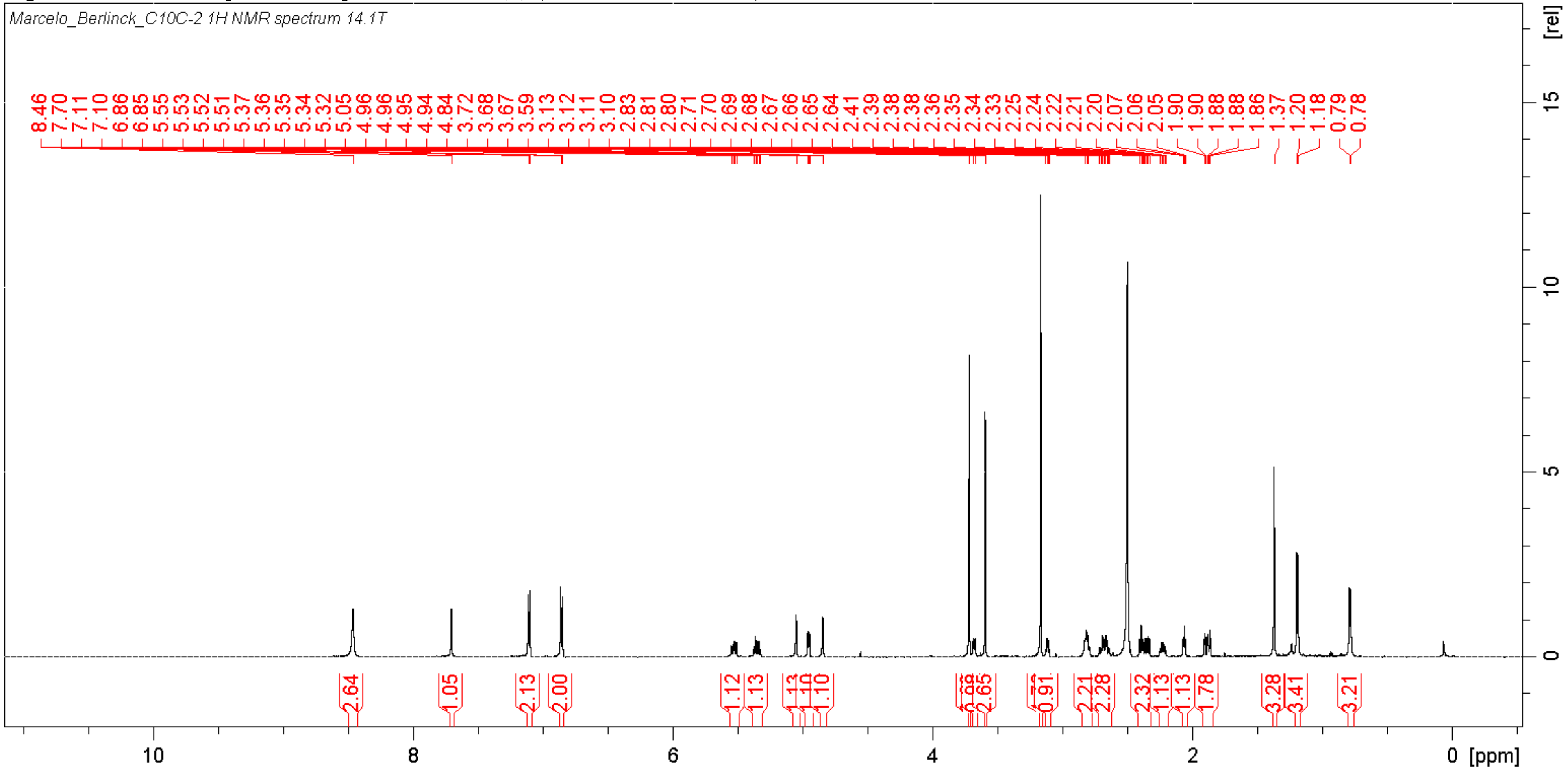

Signals at δ_H 8.46 and 3.16 are assigned to traces of formic acid and MeOH, used in HPLC purification.

Figure S4. Expansion of the ^{1}H NMR spectrum of perochalasin A (**1**) (600 MHz, DMSO-d_6).

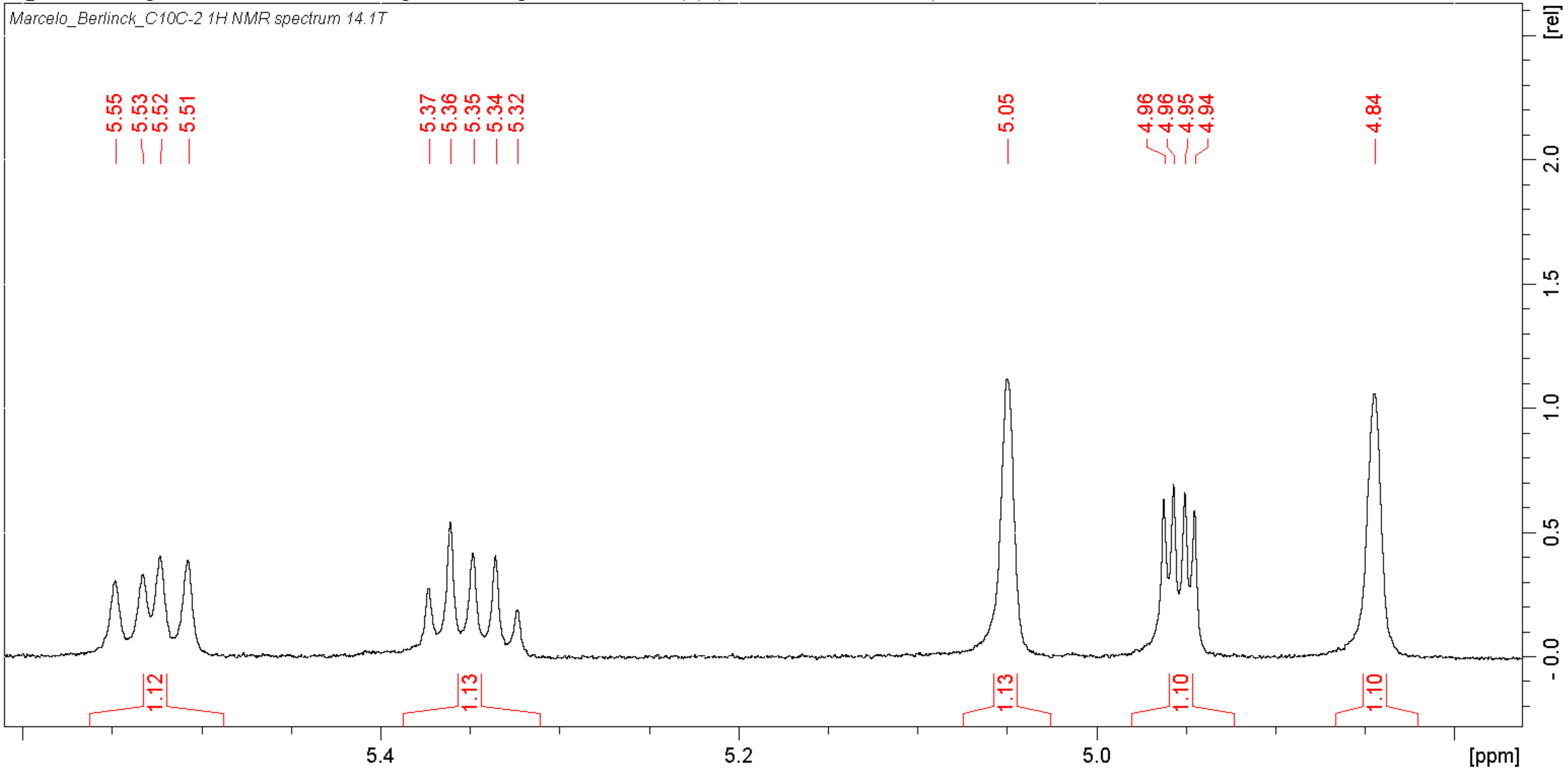

Figure S5. Expansion of the ^{1}H NMR spectrum of perochalasin A (**1**) (600 MHz, DMSO-d_6).

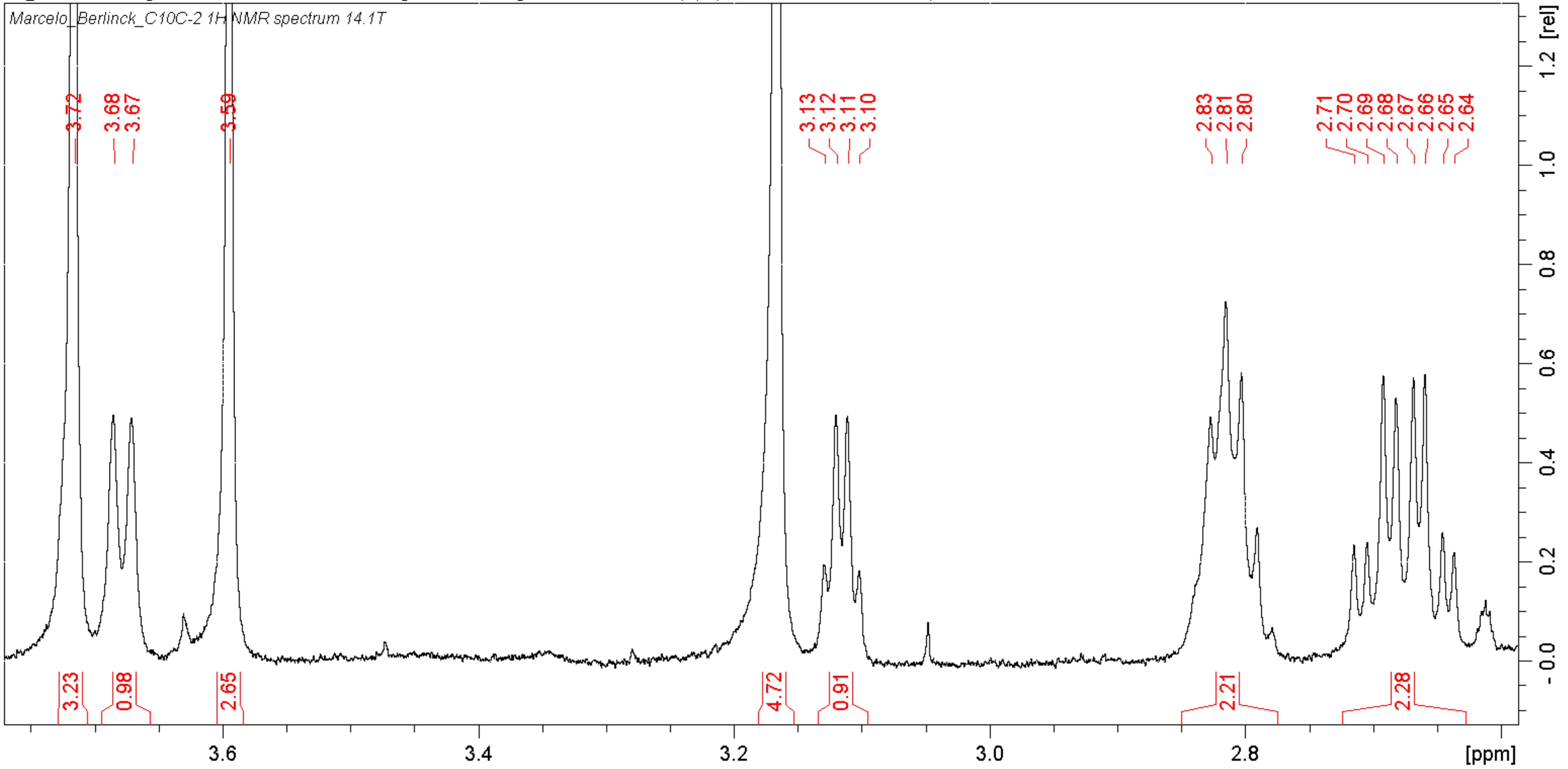

Figure S6. Expansion of the ^{1}H NMR spectrum of perochalasin A (**1**) (600 MHz, DMSO-d_6).

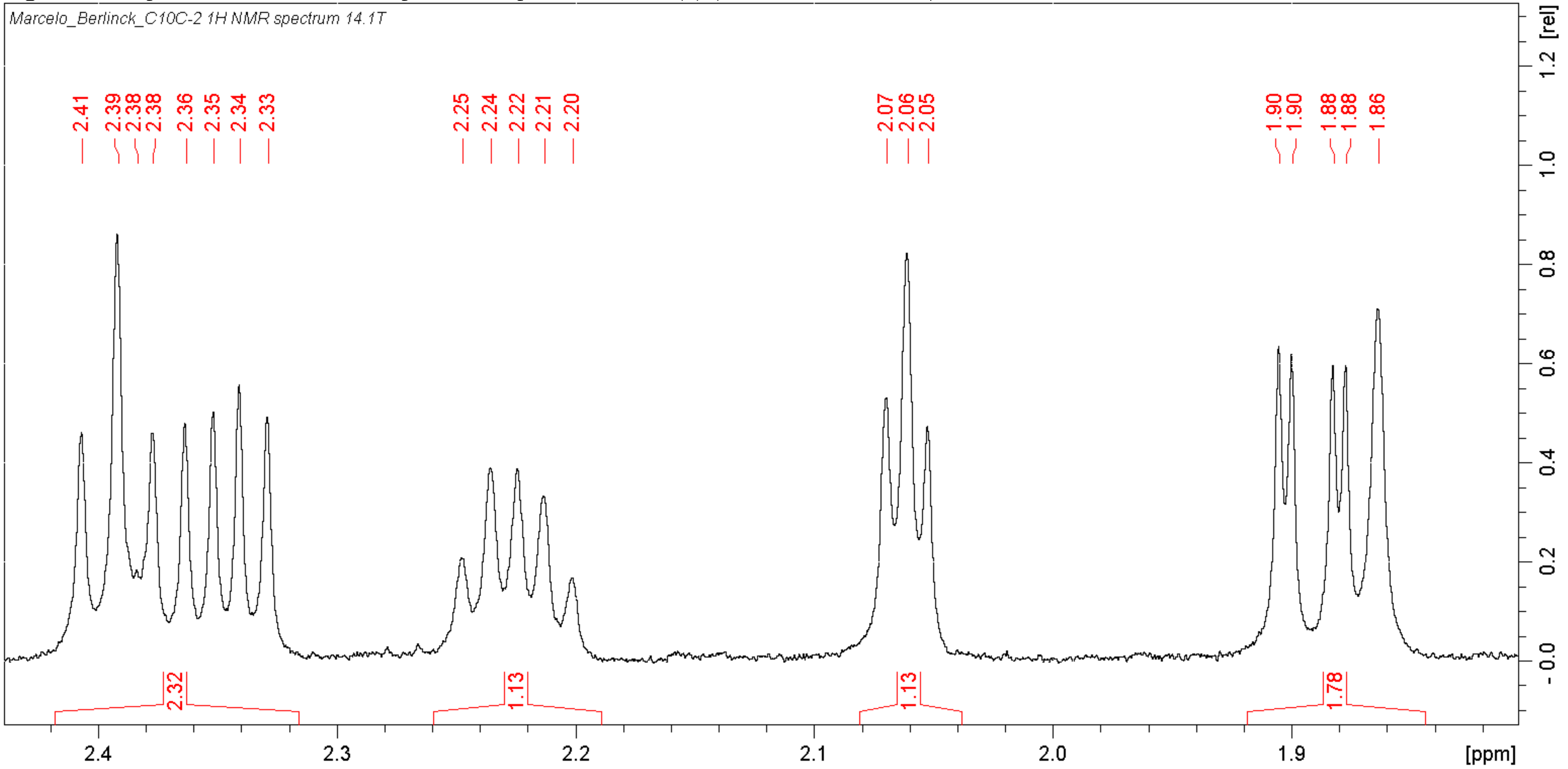

Figure S7. Expansion of the ^{1}H NMR spectrum of perochalasin A (**1**) (600 MHz, DMSO-d_6).

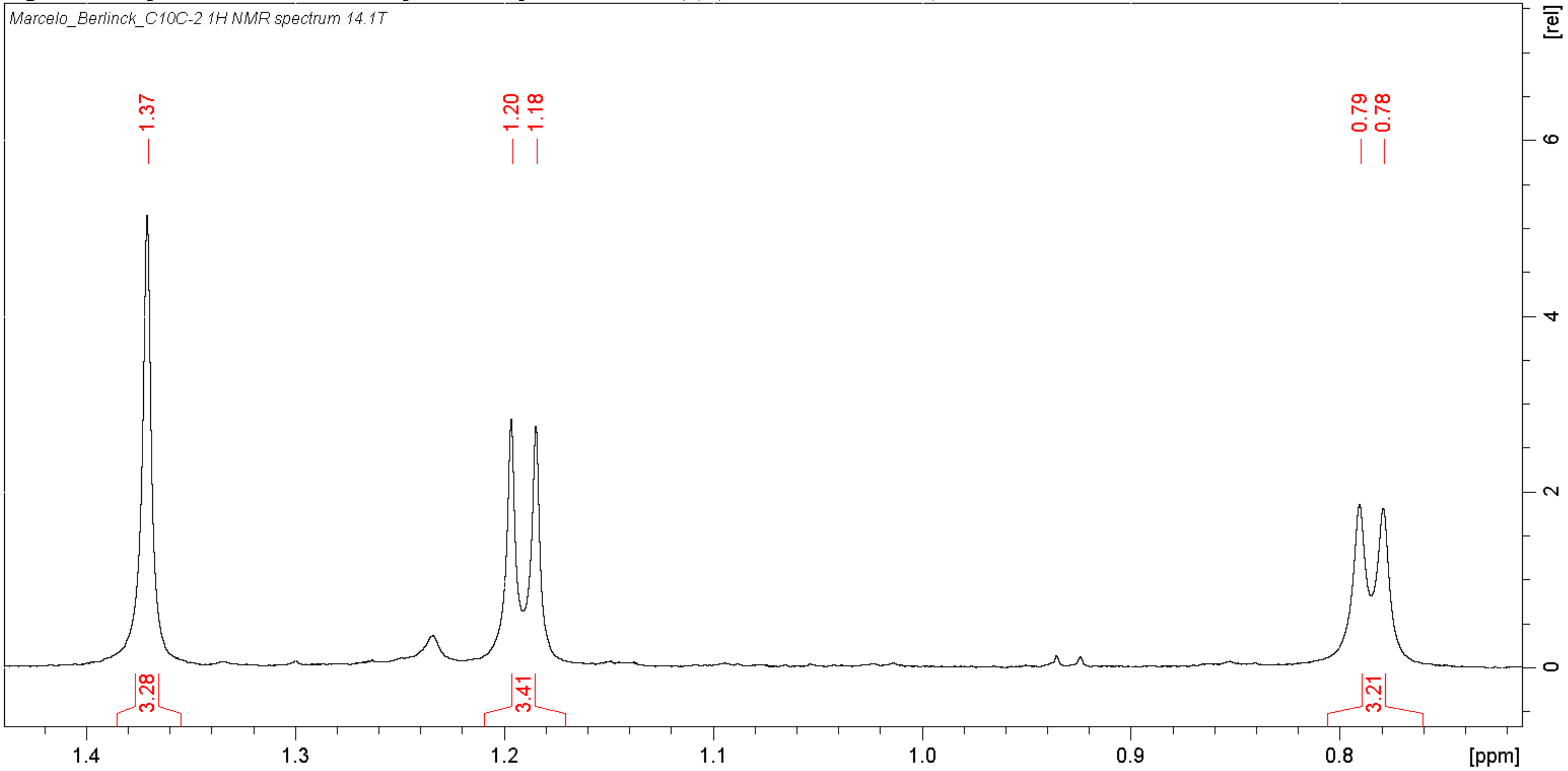

Figure S8. ^{13}C NMR spectrum of perochalasin A (**1**) (150 MHz, DMSO-d_6).

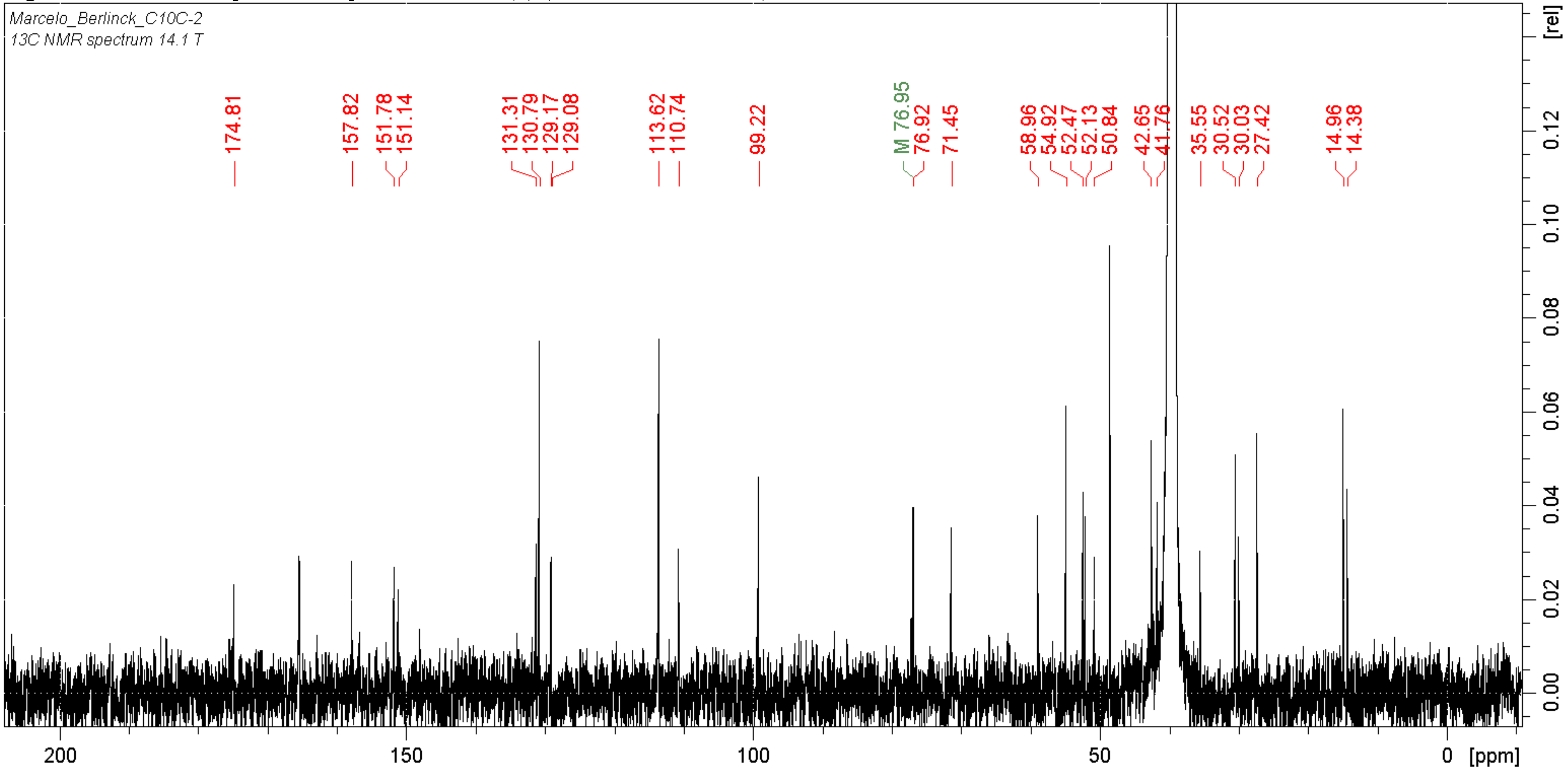

Signal at δ_C 165.4 and δ_C 48.6 are assigned to residual formic acid and MeOH, used during HPLC purification.

Figure S9. HSQC spectrum of perochalasin A (**1**) (^{1}H: 600 MHz, ^{13}C: 150 MHz; DMSO-d_6).

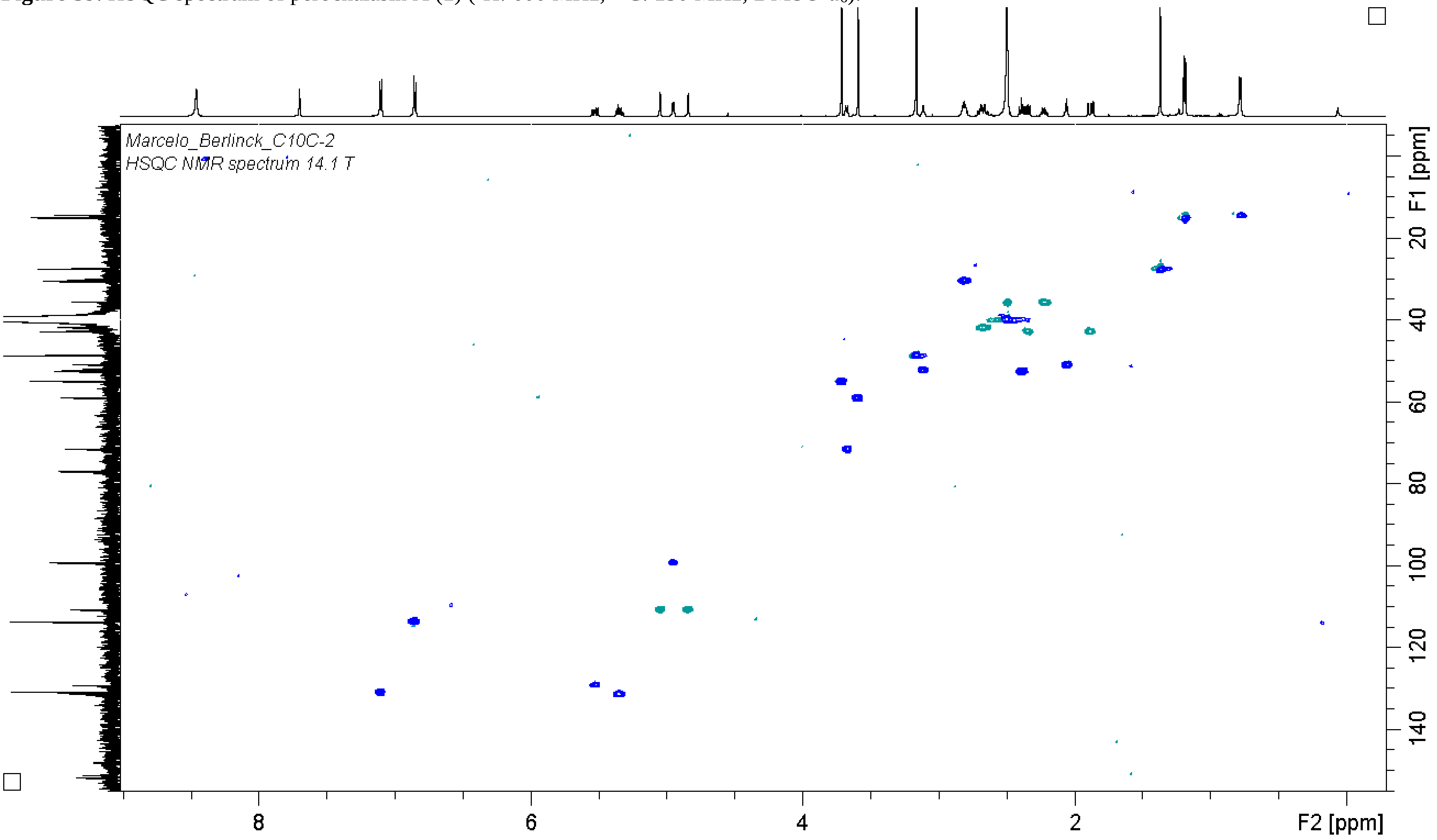

Figure S10. HMBC spectrum of perochalasin A (**1**) (^{1}H: 600 MHz, ^{13}C: 150 MHz; DMSO-d_6).

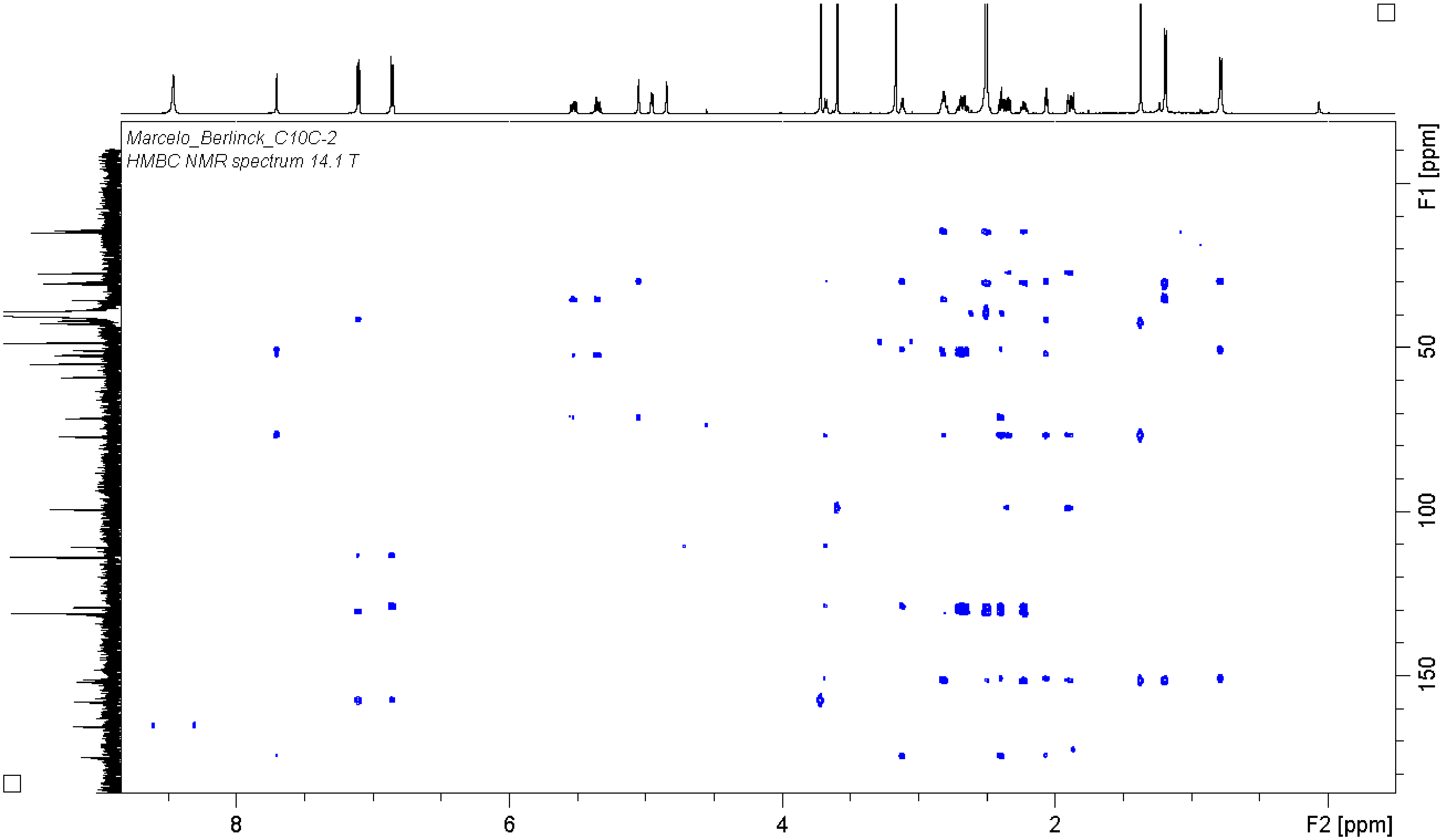

Figure S11. COSY spectrum of perochalasin A (**1**) (600 MHz, DMSO-d_6).

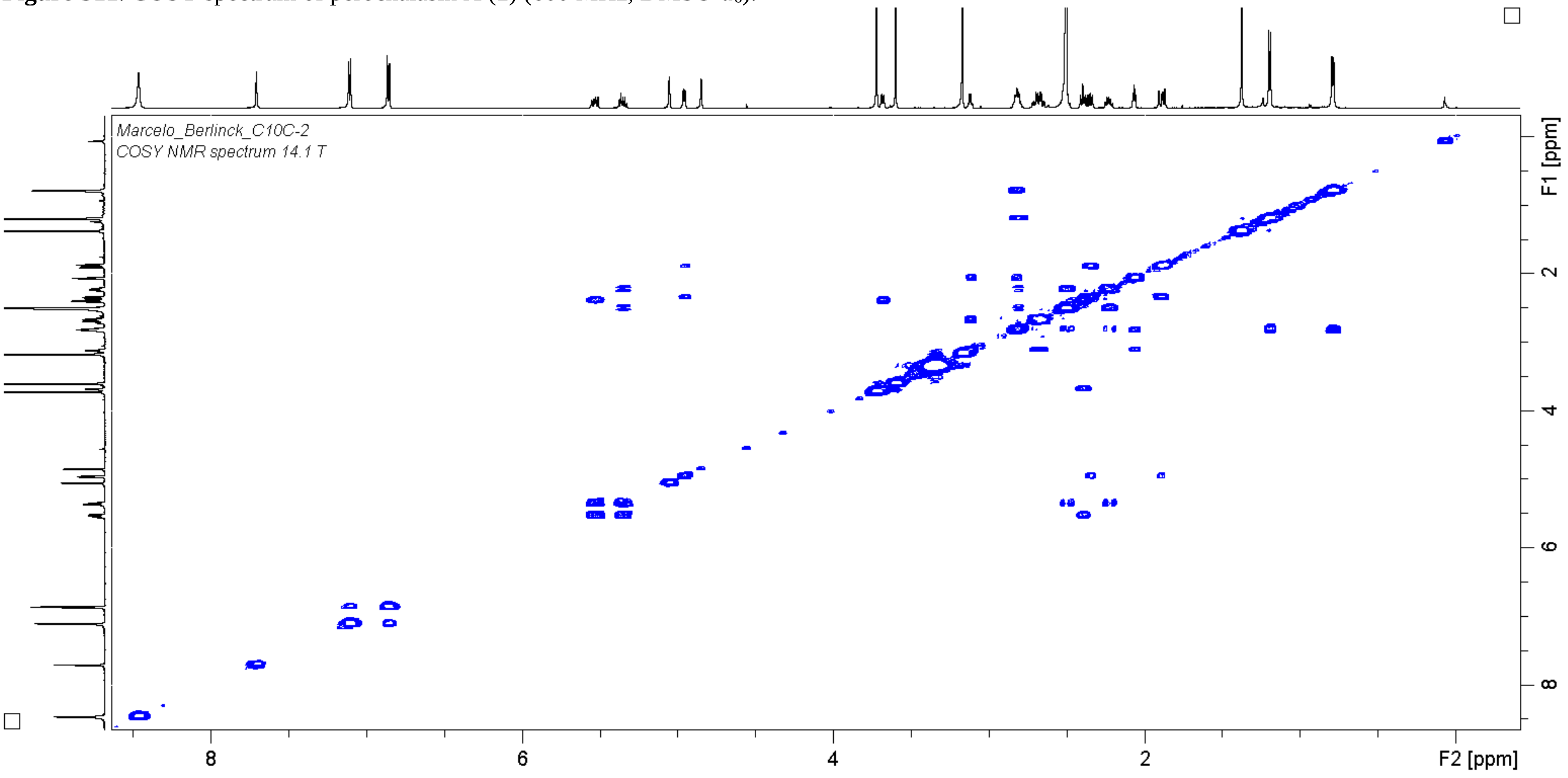

Figure S12. (+)-HRESIMS spectrum of perochalasin A (**1**).

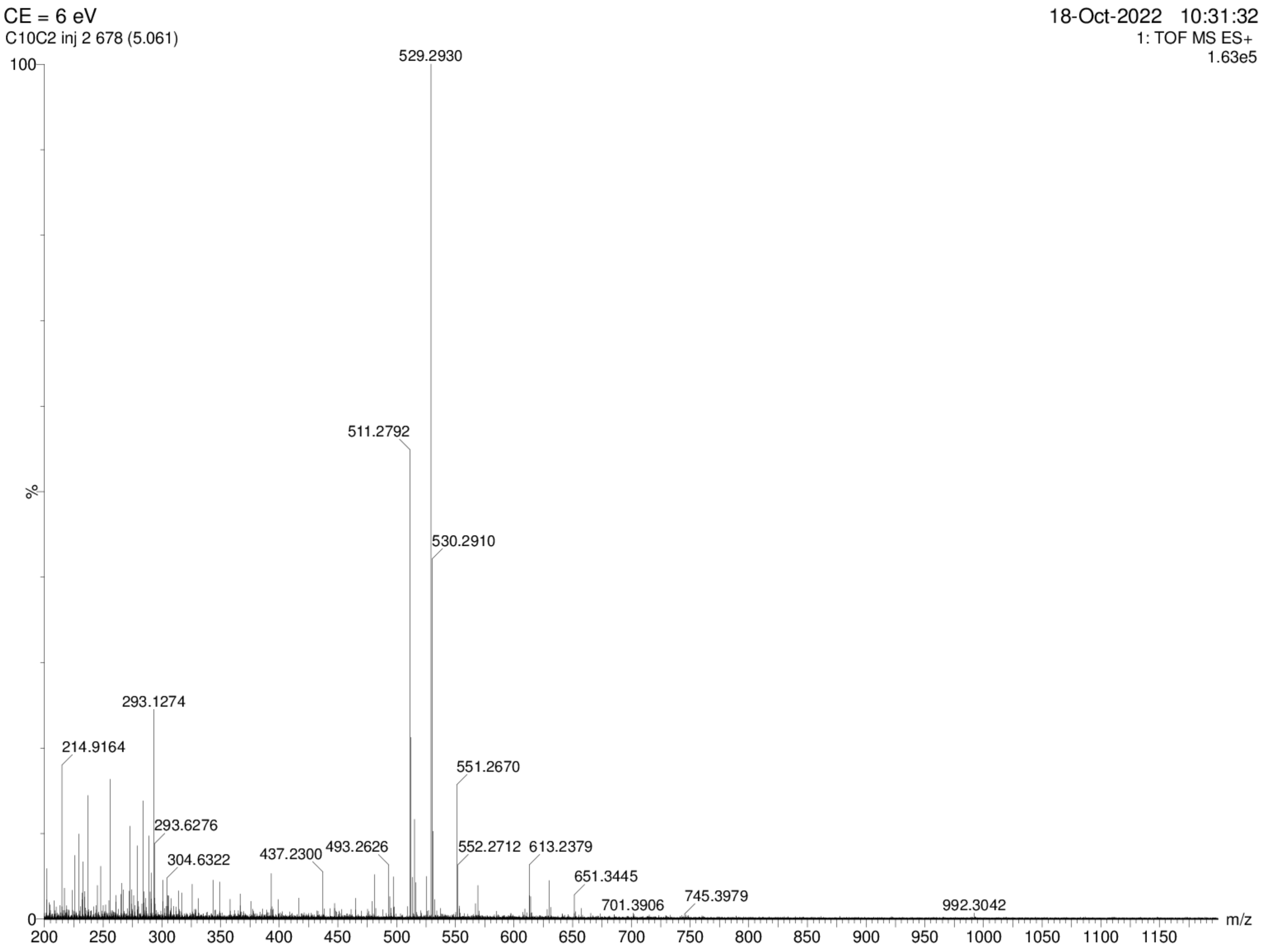

Figure S13. 1D-NOESY spectrum of perochalasin A (**1**) (600 MHz, DMSO-d_6). ^{1}H NMR spectrum (**A**), and irradiation at δ_H 2.39 (**B**).

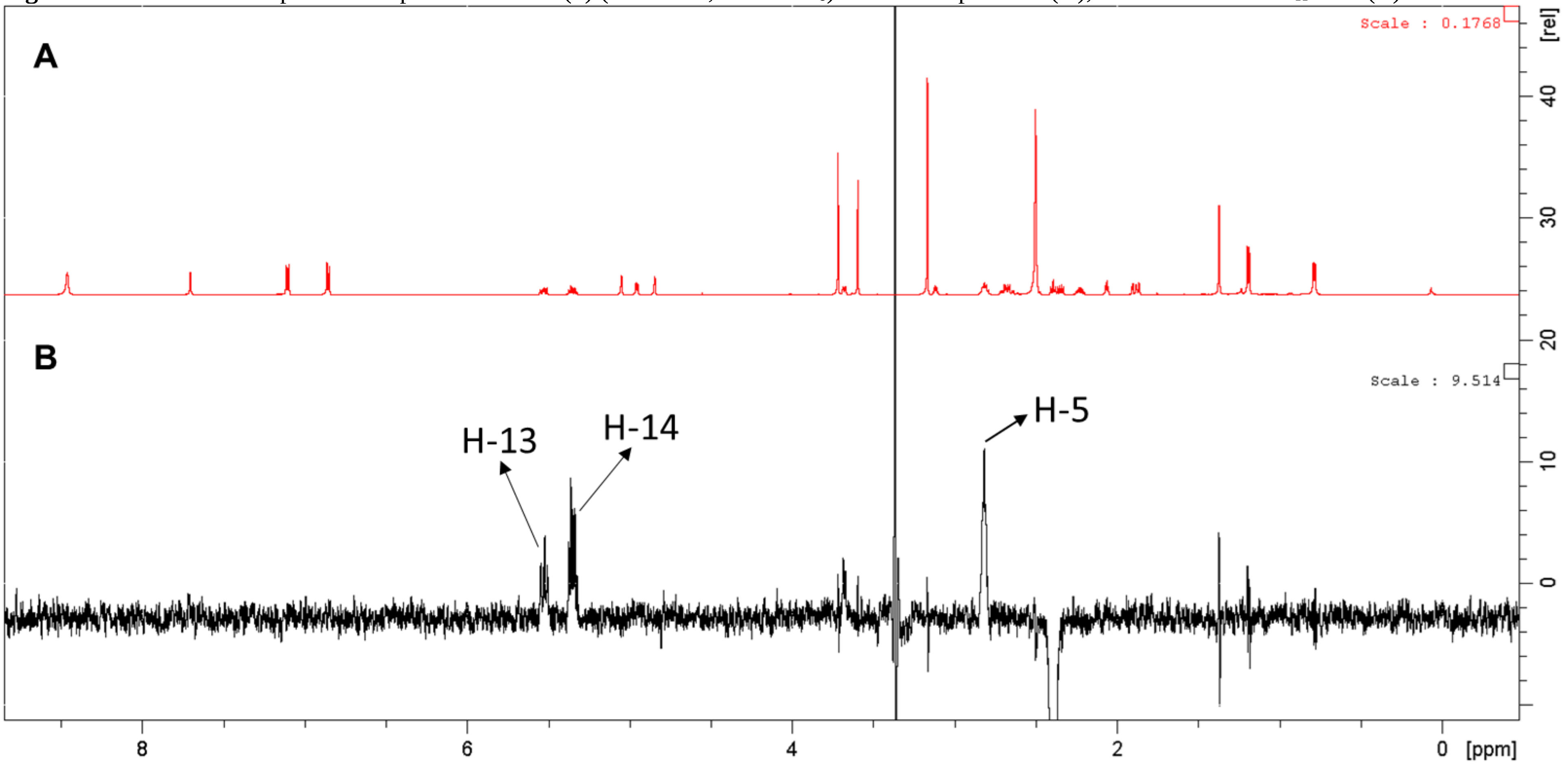

Figure S14. 1D-NOESY spectrum of perochalasin A (**1**) (600 MHz, DMSO-d_6). ^{1}H NMR spectrum (**A**), and irradiation at δ_H 0.78 (**B**).

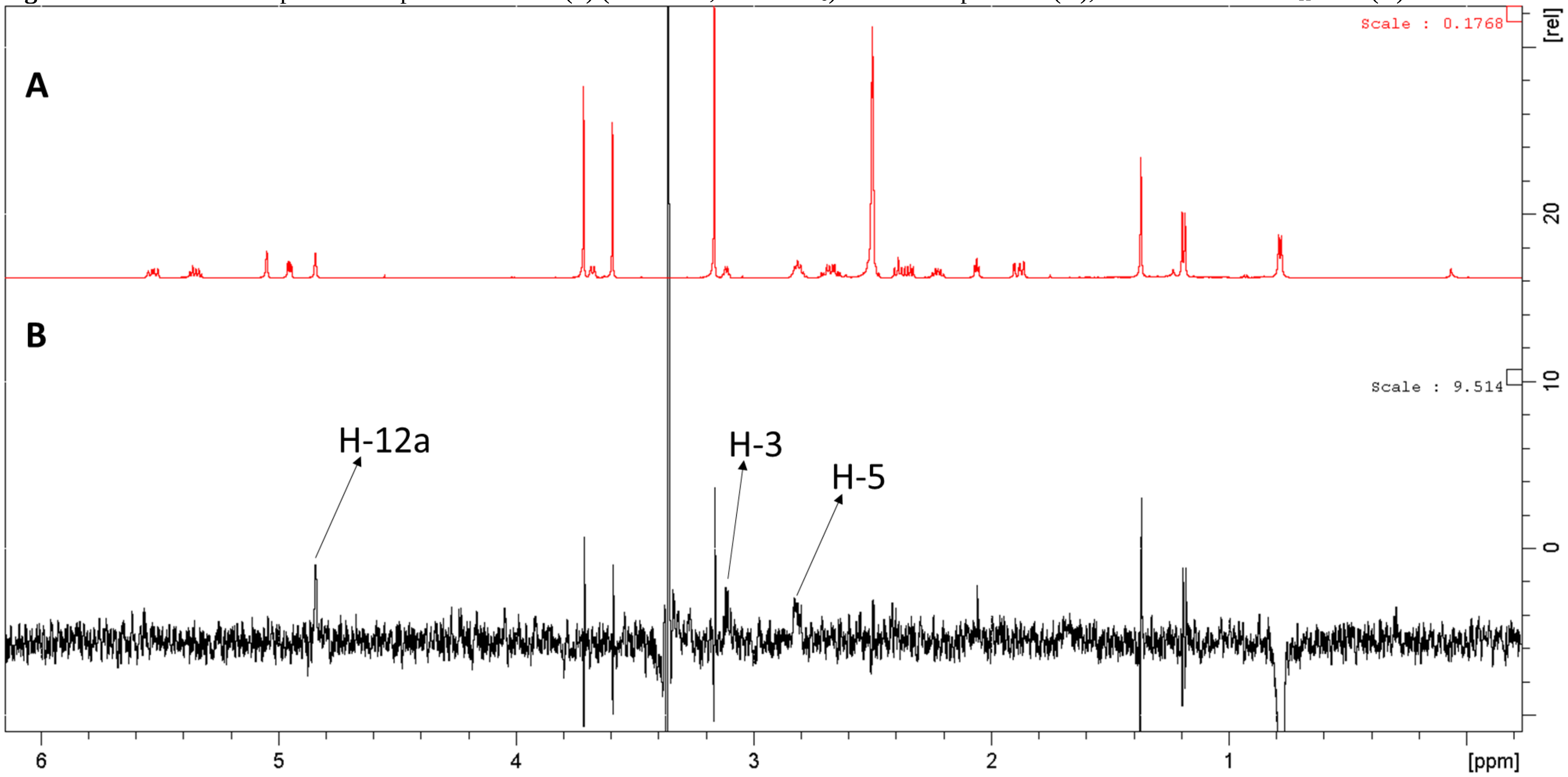

Figure S15. 1D-NOESY spectrum of perochalasin A (**1**) (600 MHz, DMSO-d_6). ^{1}H NMR spectrum (**A**), and irradiation at δ_H 2.06 (**B**).

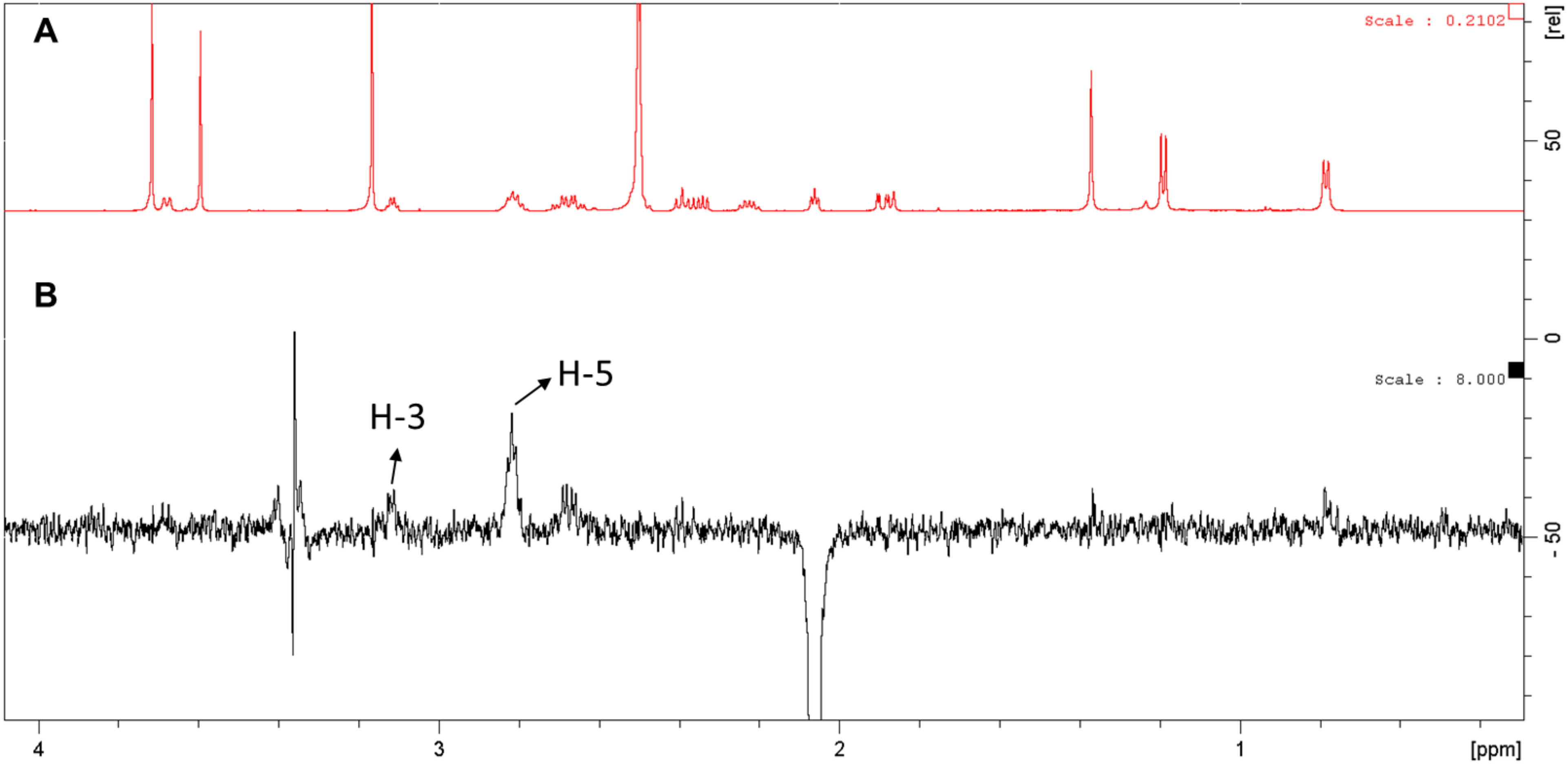

Figure S16. 1D-NOESY spectrum of perochalasin A (**1**) (600 MHz, DMSO-d_6). ^{1}H NMR spectrum (**A**), and irradiation at δ_H 3.69 (**B**).

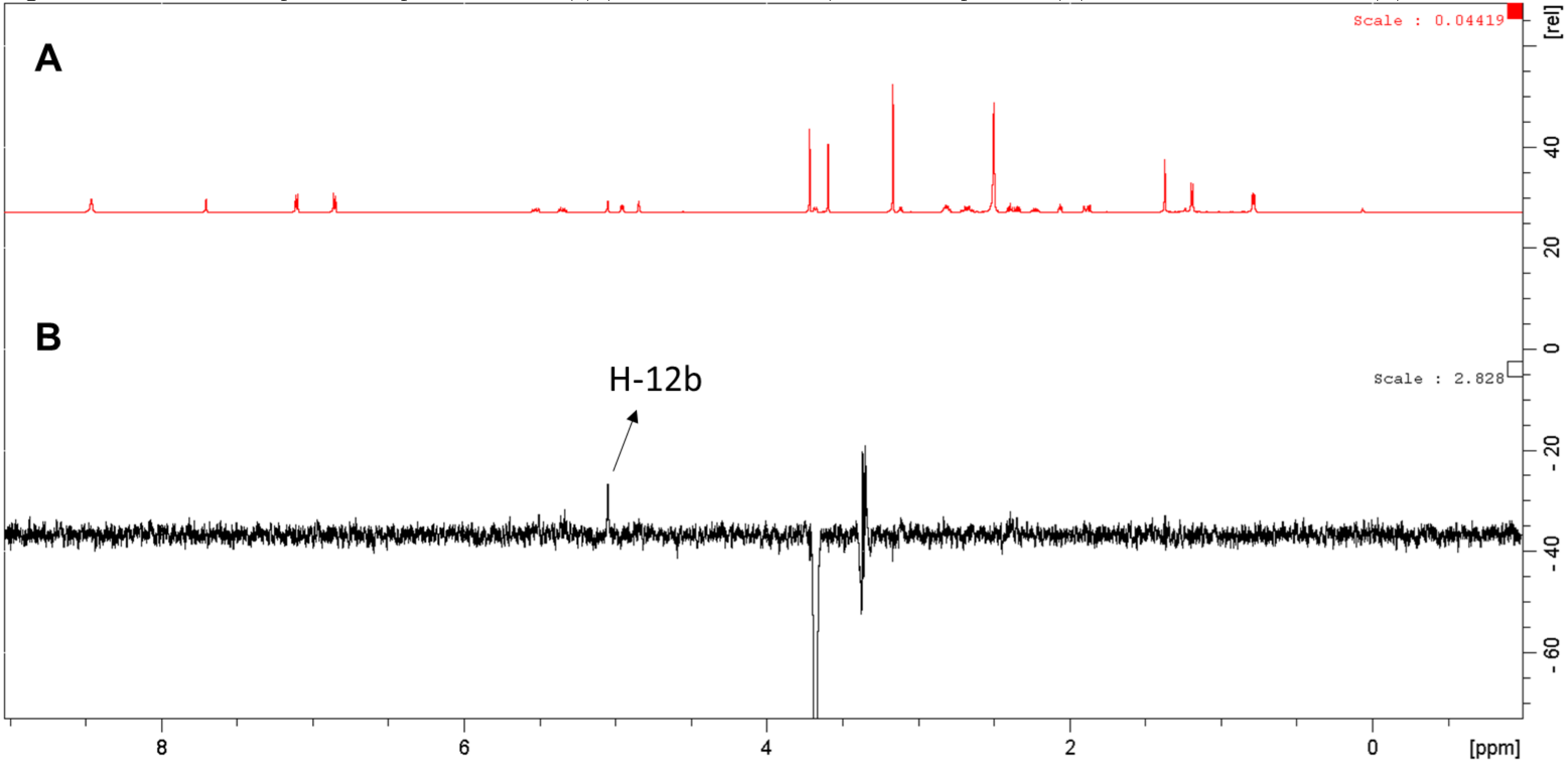

Figure S17. 1D-NOESY spectrum of perochalasin A (**1**) (600 MHz, DMSO-d_6). ^{1}H NMR spectrum (**A**), and irradiation at δ_H 4.95 (**B**).

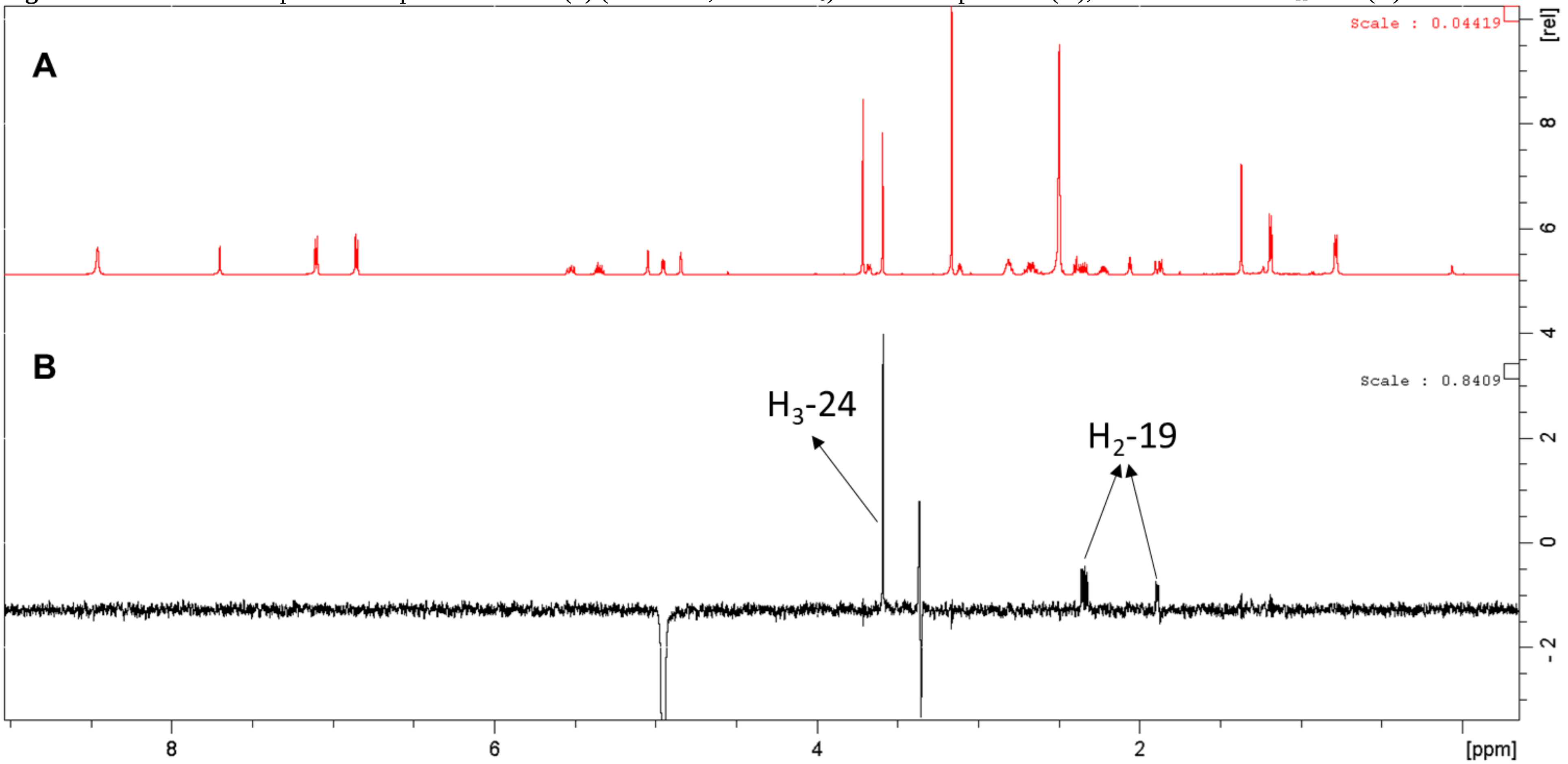

Figure S18. UV spectrum (150 µg/mL, MeOH) of perochalasin A (**1**).

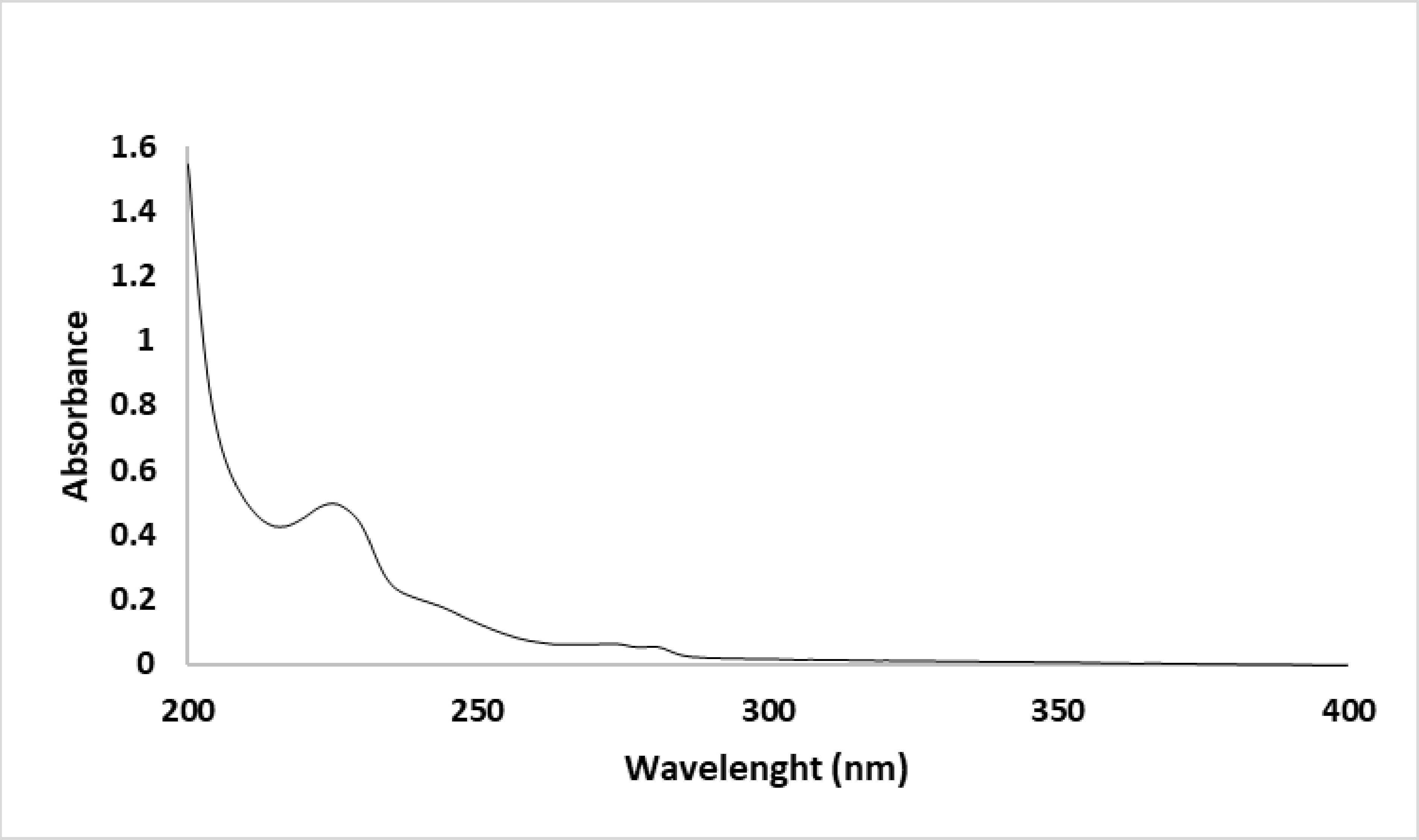

Figure S19. ECD spectrum (150 µg/mL, MeOH) of perochalasin A (**1**).

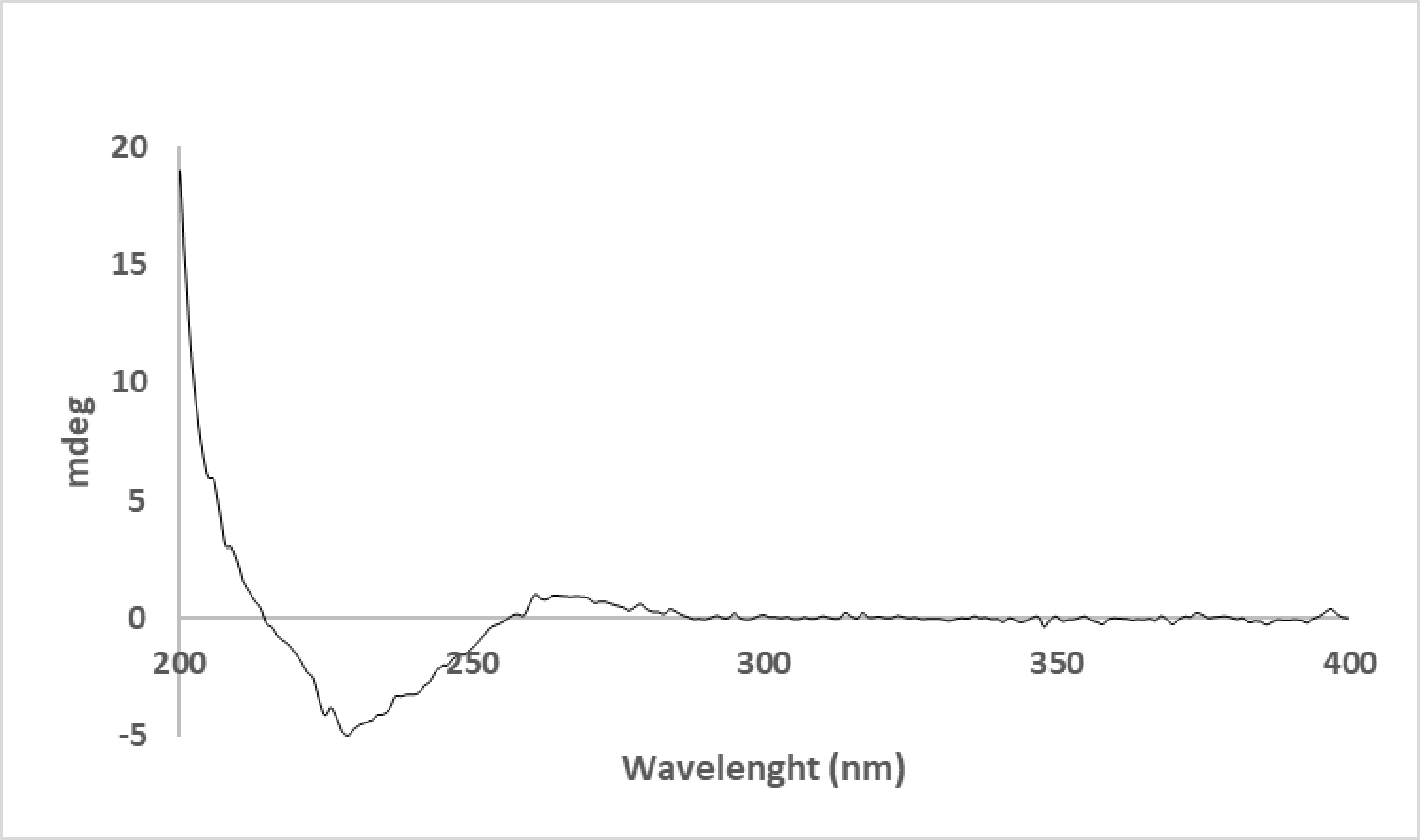

Figure S20. ^{1}H NMR spectrum of perochalasin B (**2a**) and *epi*-perochalasin B (**2b**) (600 MHz, DMSO-d_6).

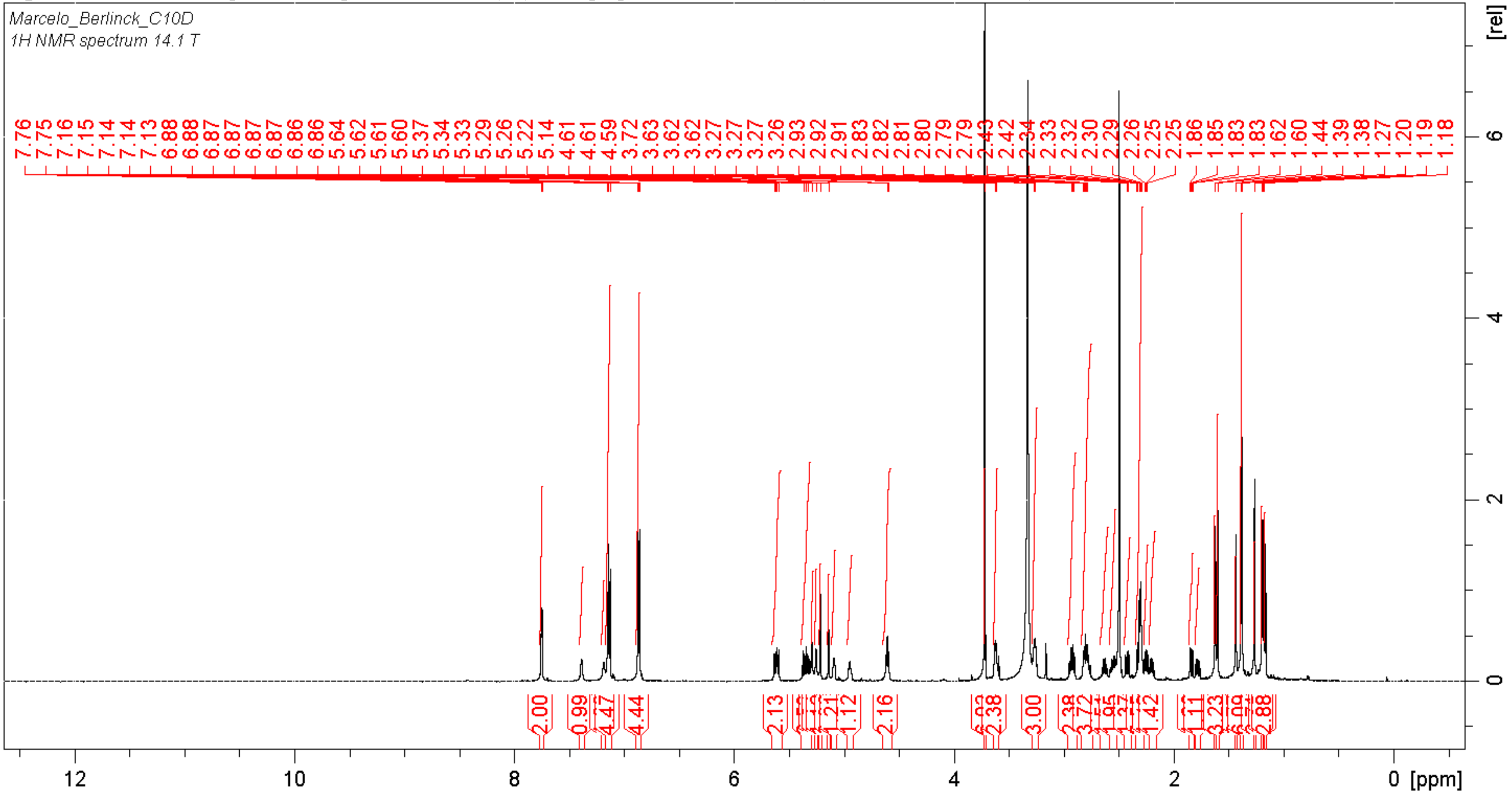

Figure S21. Expansion of the ^{1}H NMR spectrum of perochalasin B (**2a**) and *epi*-perochalasin B (**2b**) (600 MHz, DMSO-d_6).

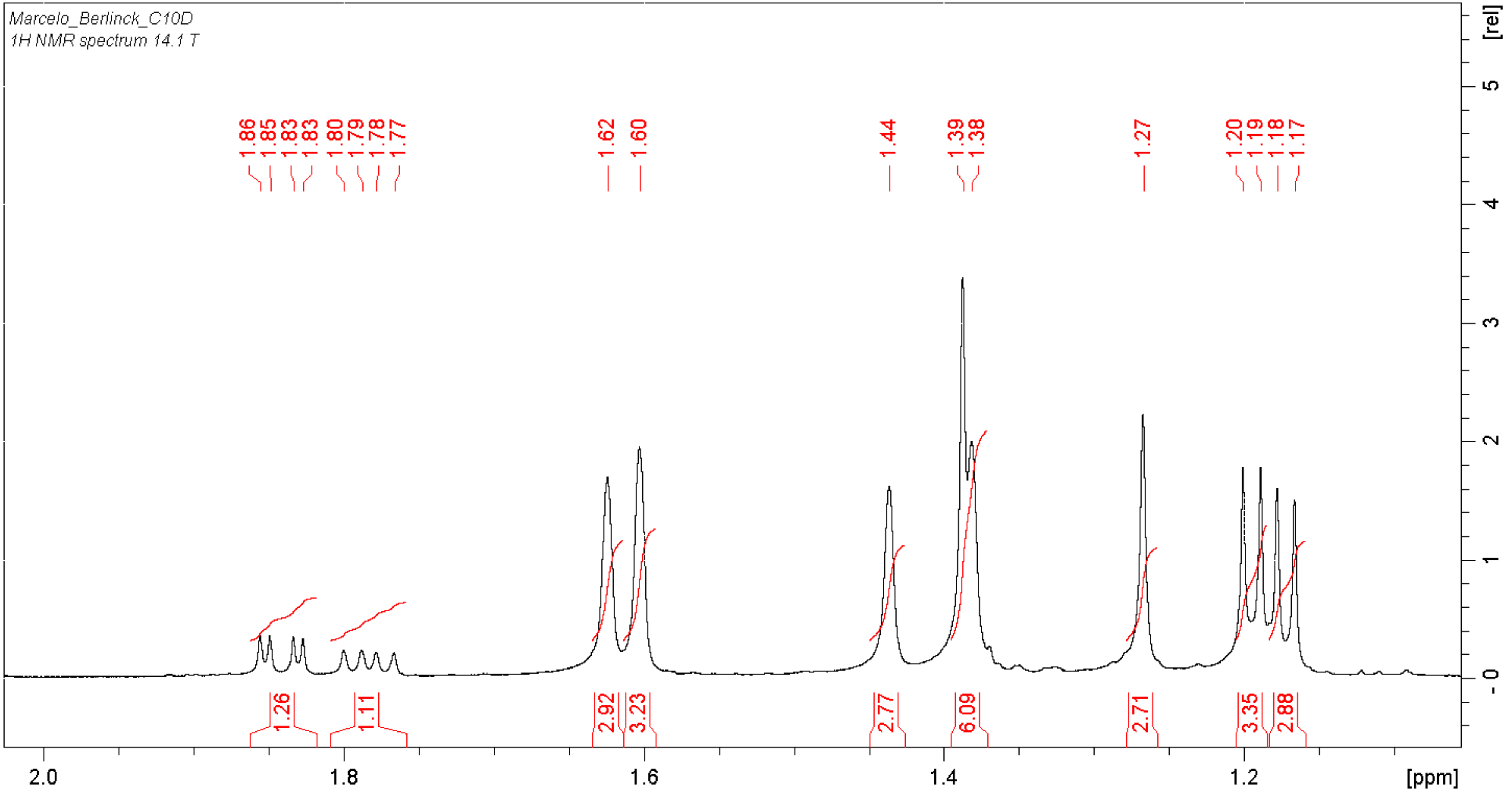

Figure S22. Expansion of the ^{1}H NMR spectrum of perochalasin B (**2a**) and *epi*-perochalasin B (**2b**) (600 MHz, DMSO-d_6).

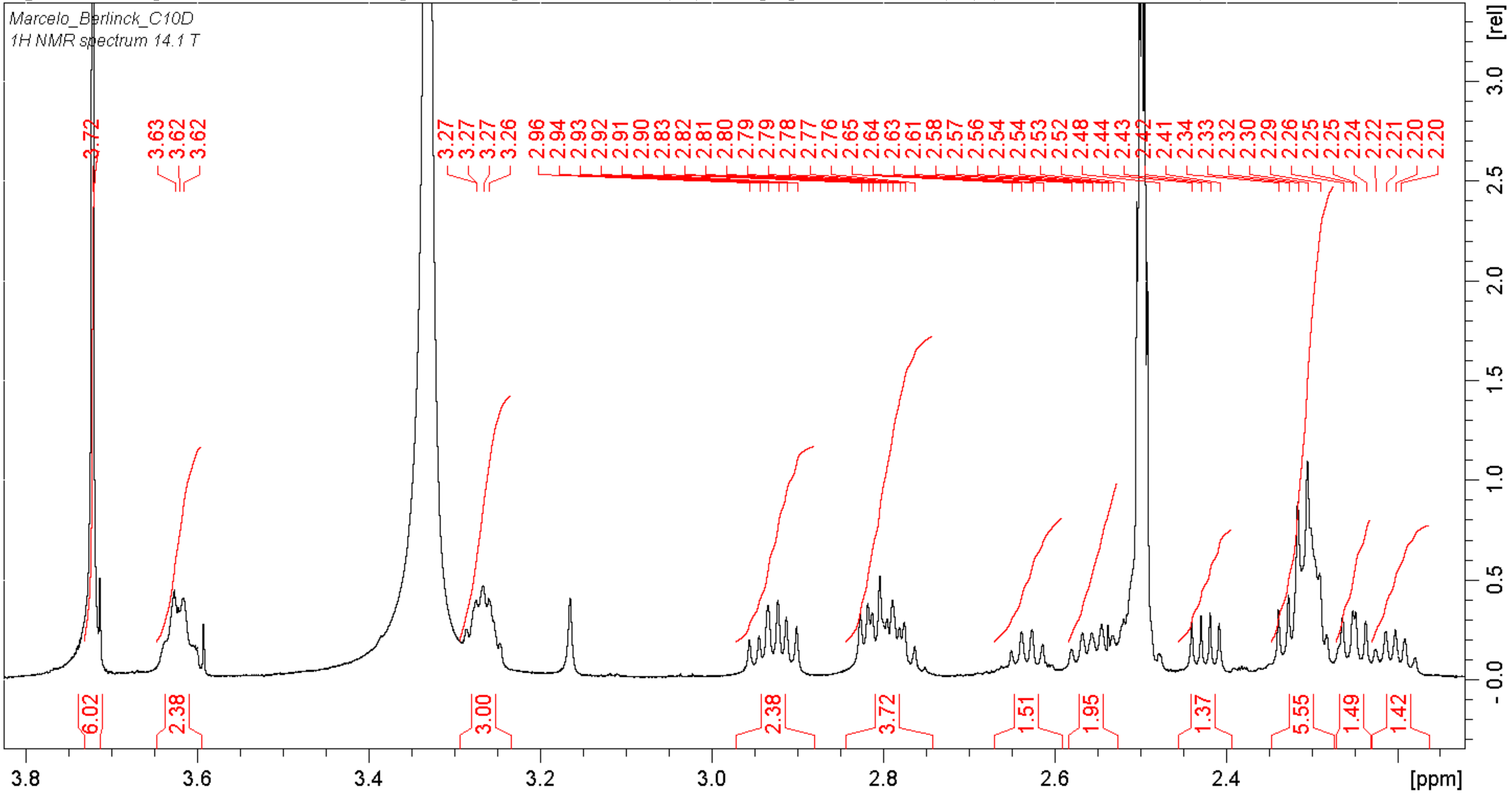

Figure S23. Expansion of the ^{1}H NMR spectrum of perochalasin B (**2a**) and *epi*-perochalasin B (**2b**) (600 MHz, DMSO-d_6).

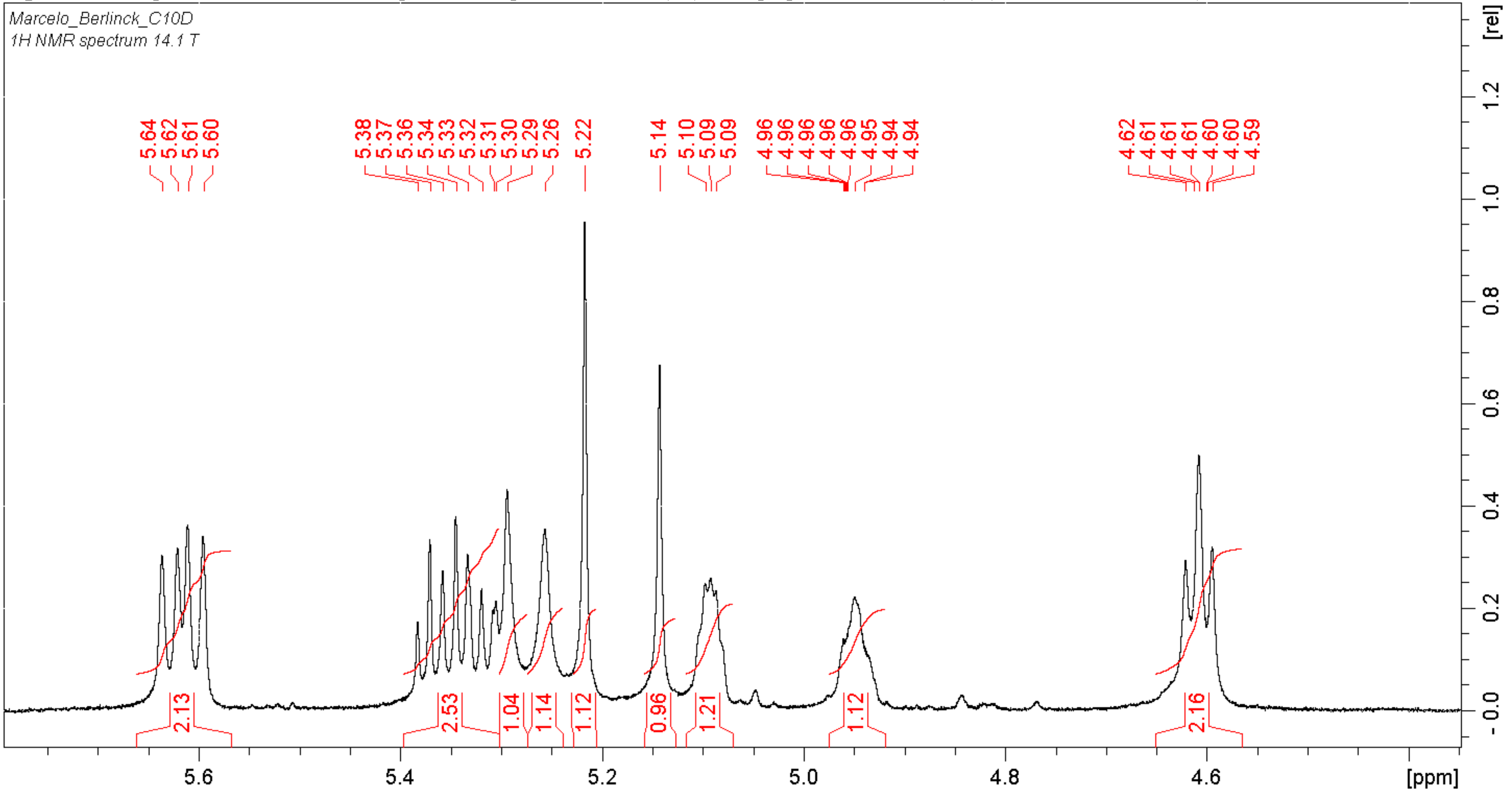

Figure S24. Expansion of the ^{1}H NMR spectrum of perochalasin B (**2a**) and *epi*-perochalasin B (**2b**) (600 MHz, DMSO-d_6).

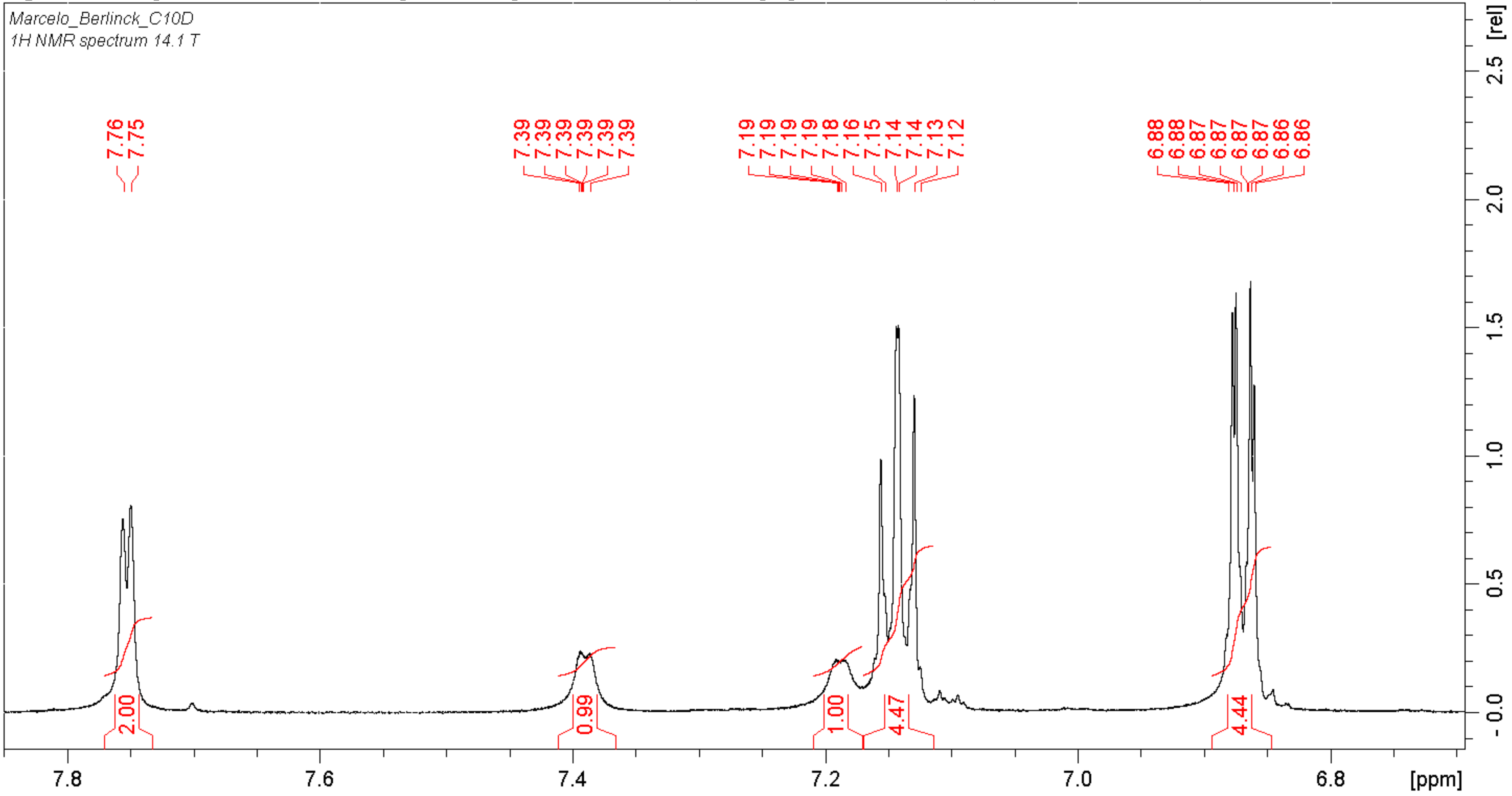

Figure S25. ^{13}C NMR spectrum of perochalasin B (**2a**) and *epi*-perochalasin B (**2b**) (150 MHz, DMSO-d_6).

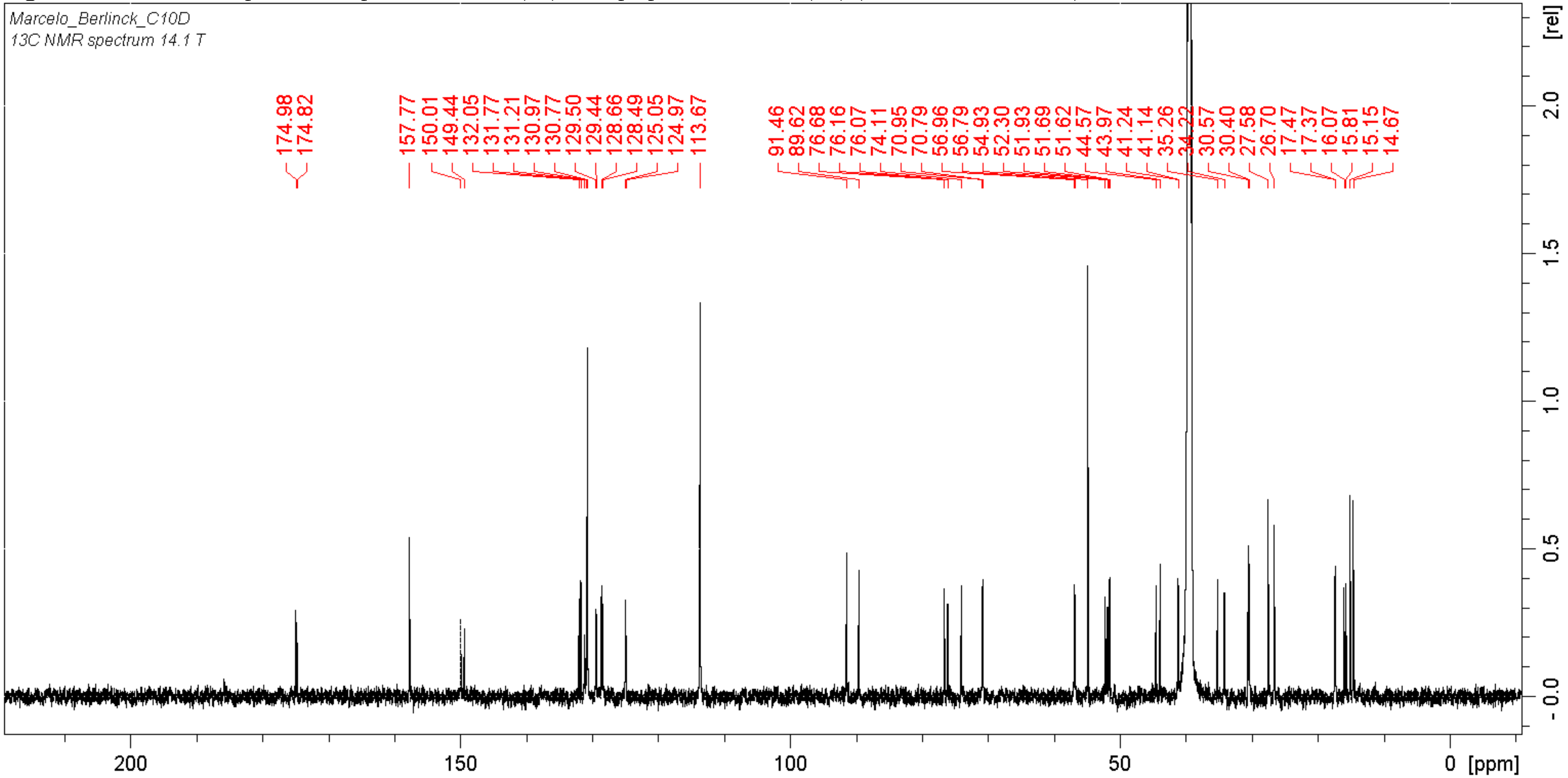

Figure S26. HSQC spectrum of perochalasin B (**2a**) and *epi*-perochalasin B (**2b**) (^{1}H: 600 MHz, ^{13}C: 150 MHz; DMSO-d_6).

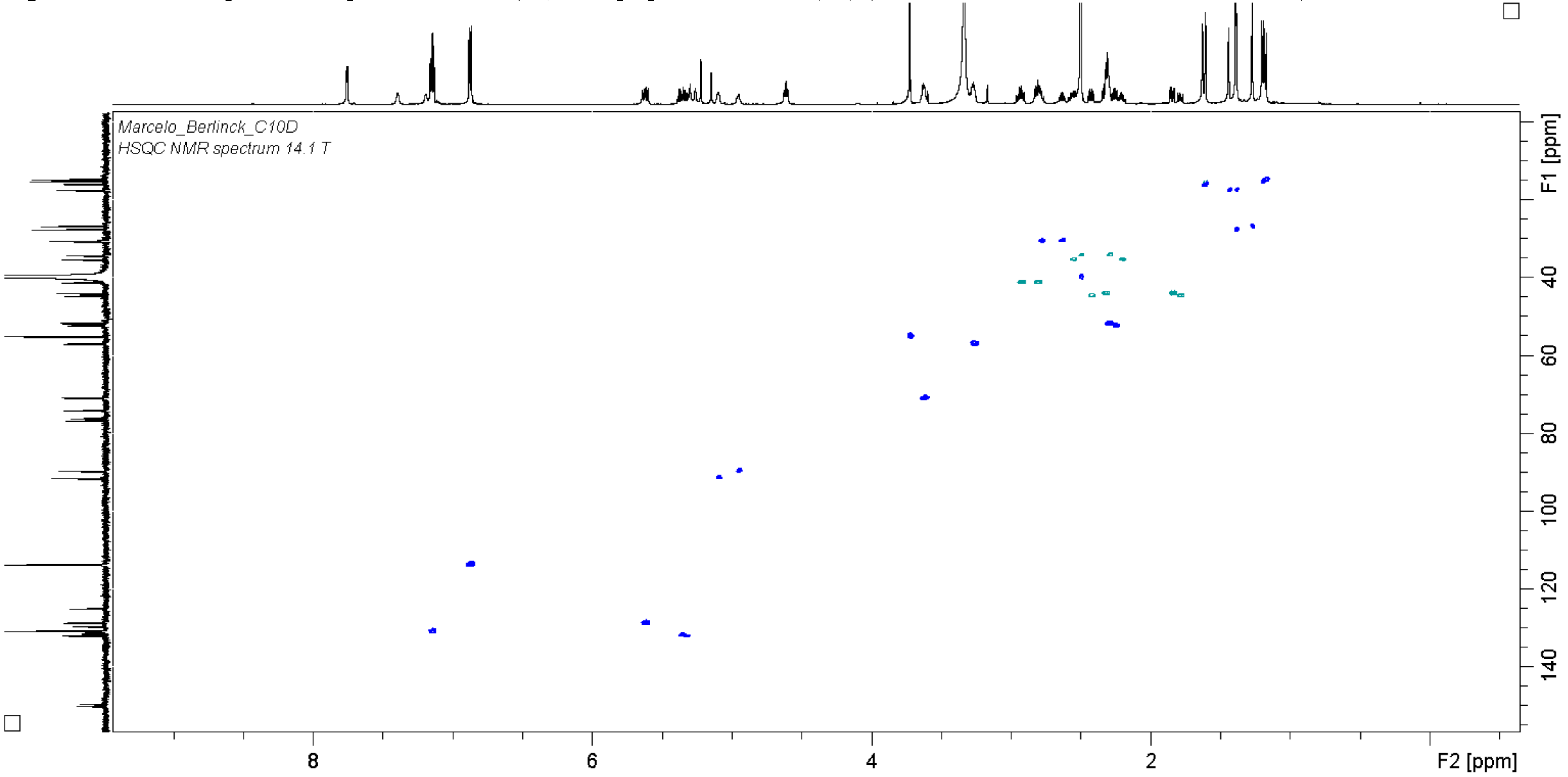

Figure S27. HMBC spectrum of perochalasin B (**2a**) and *epi*-perochalasin B (**2b**) (^{1}H: 600 MHz, ^{13}C: 150 MHz; DMSO-d_6).

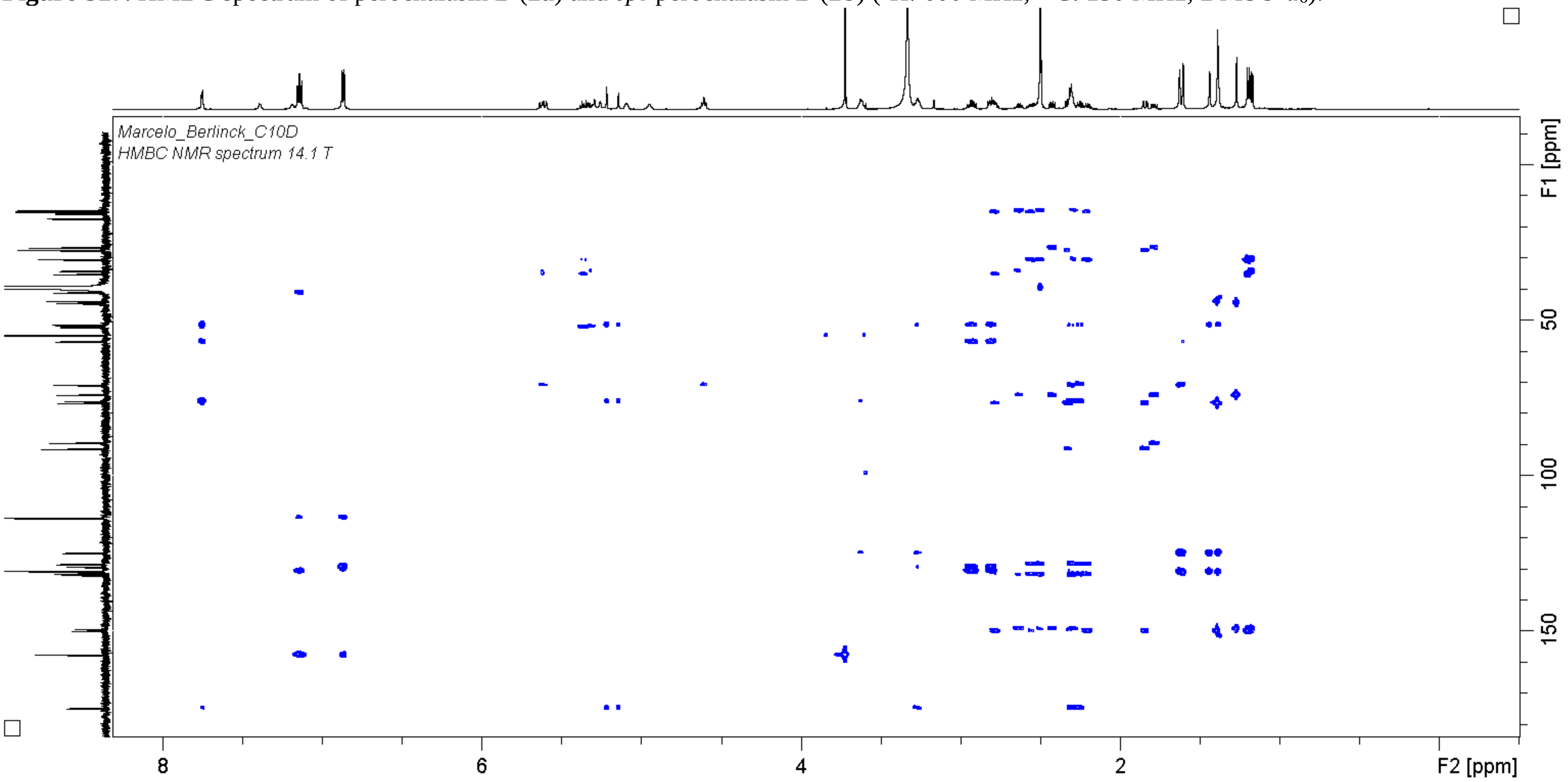

Figure S28. COSY spectrum of perochalasin B (**2a**) and *epi*-perochalasin B (**2b**) (600 MHz, DMSO-d_6).

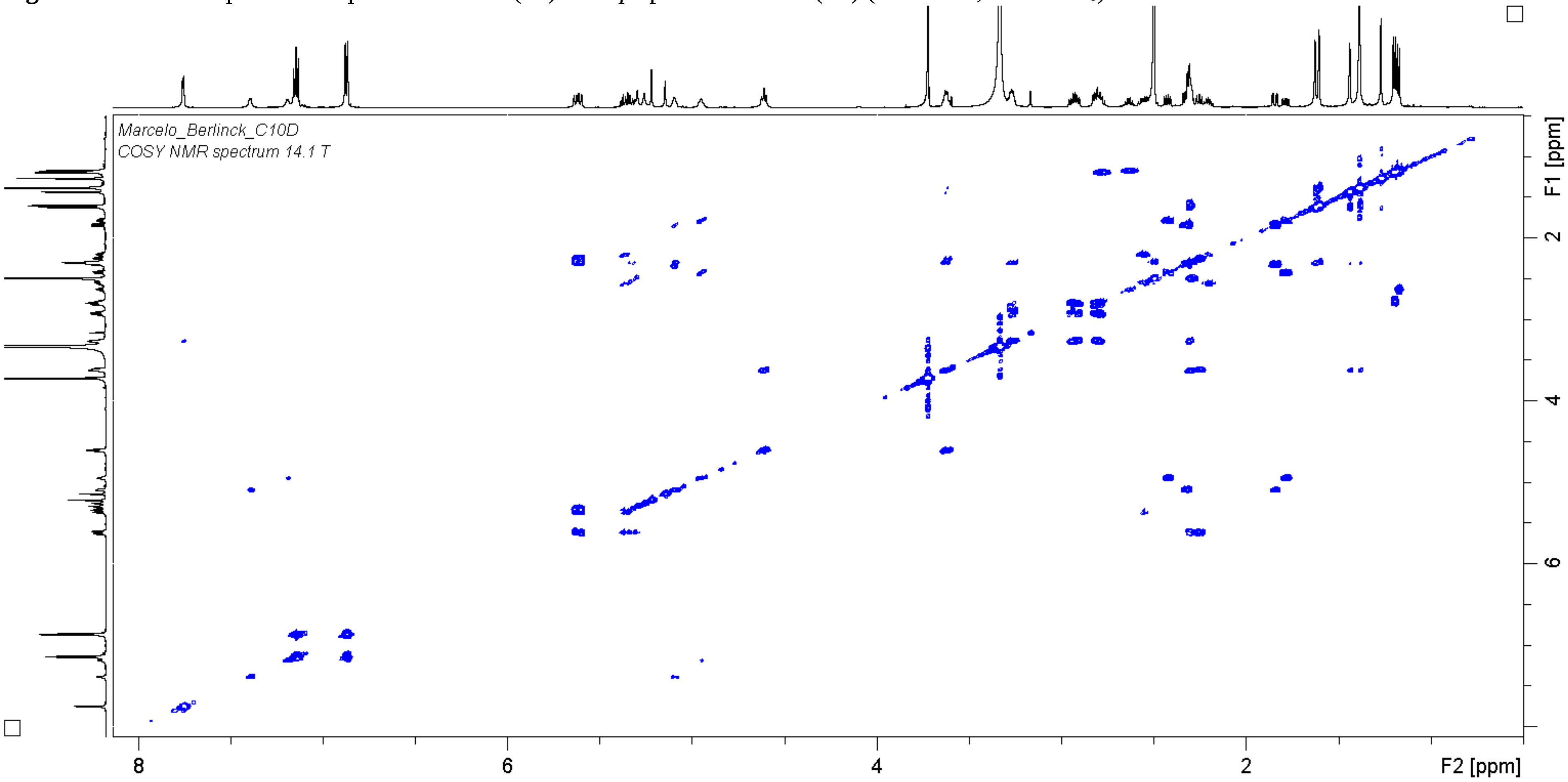

Figure S29. (+)-HRESIMS spectrum of perochalasin B (**2a**) and *epi*-perochalasin B (**2b**).

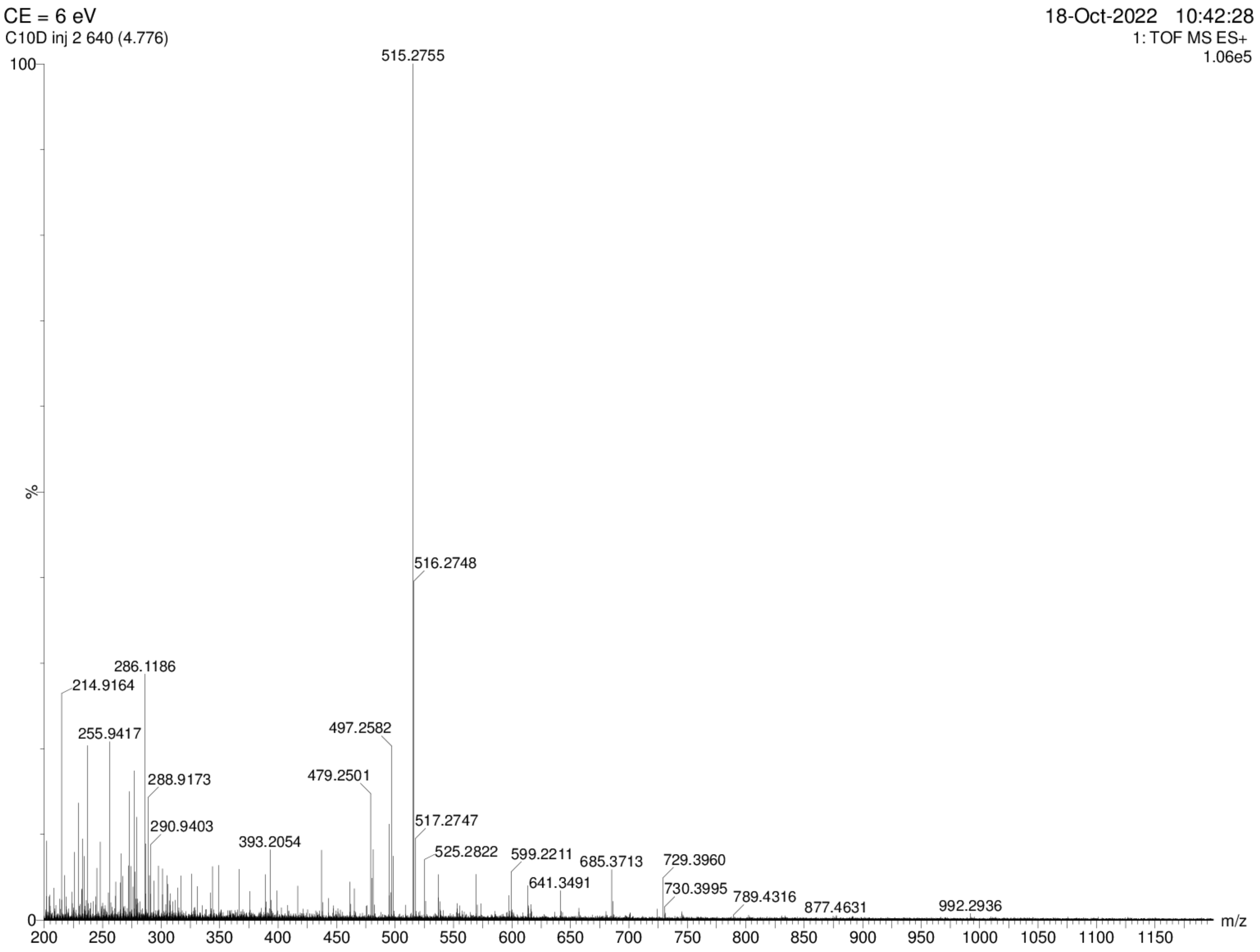

Figure S30. 1D-NOESY spectrum of perochalasin B (**2a**) and *epi*-perochalasin B (**2b**) (600 MHz, DMSO-d_6). ^{1}H NMR spectrum (**A**), and irradiation at δ_H 5.14 (**B**) and irradiation at δ_H 5.22 (**C**).

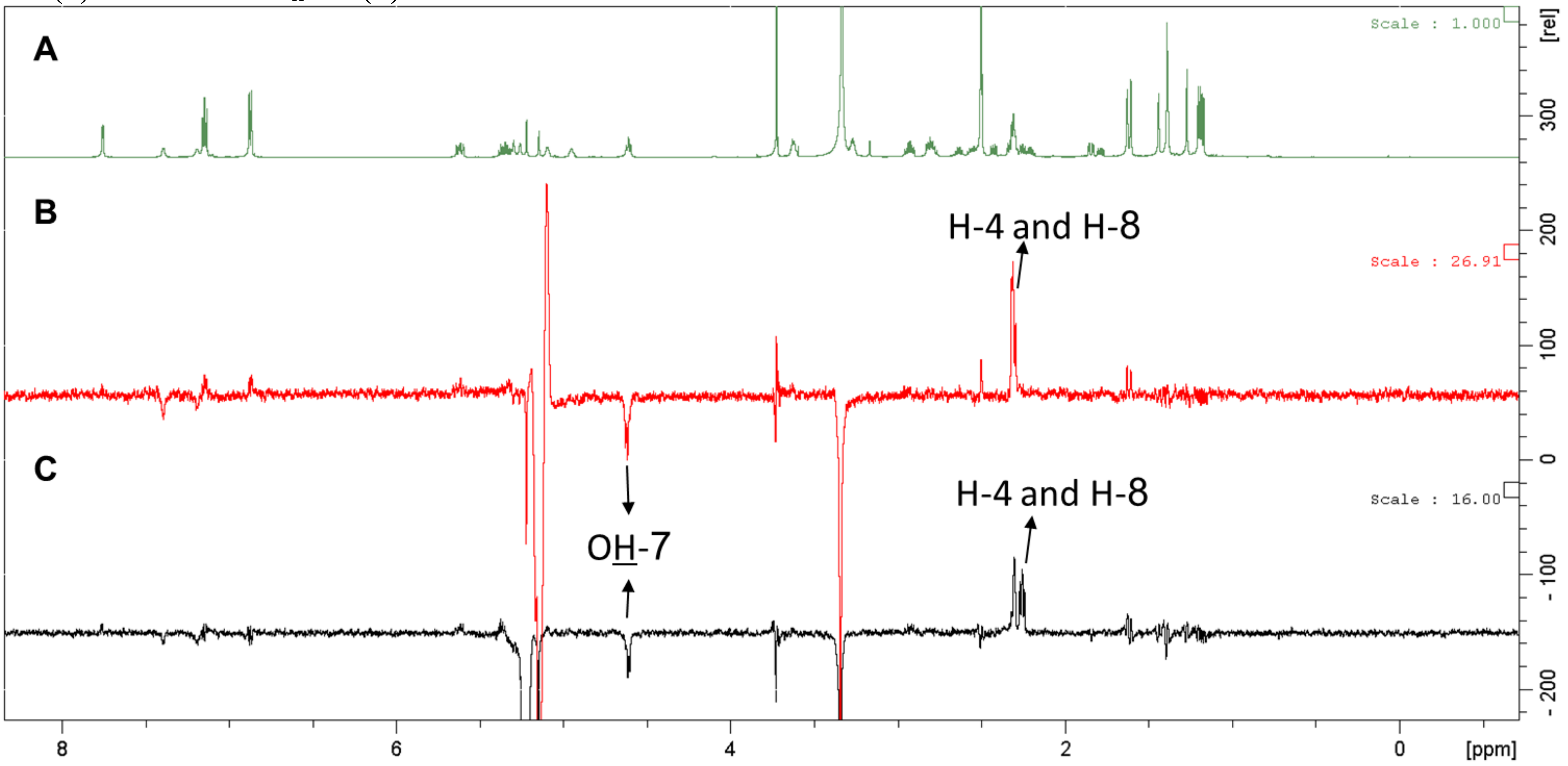

Figure S31. 1D-NOESY spectrum of perochalasin B (**2a**) and *epi*-perochalasin B (**2b**) (600 MHz, DMSO-d_6). ^{1}H NMR spectrum (**A**), and irradiation at δ_H 4.95 (**B**) and irradiation at δ_H 5.09 (**C**).

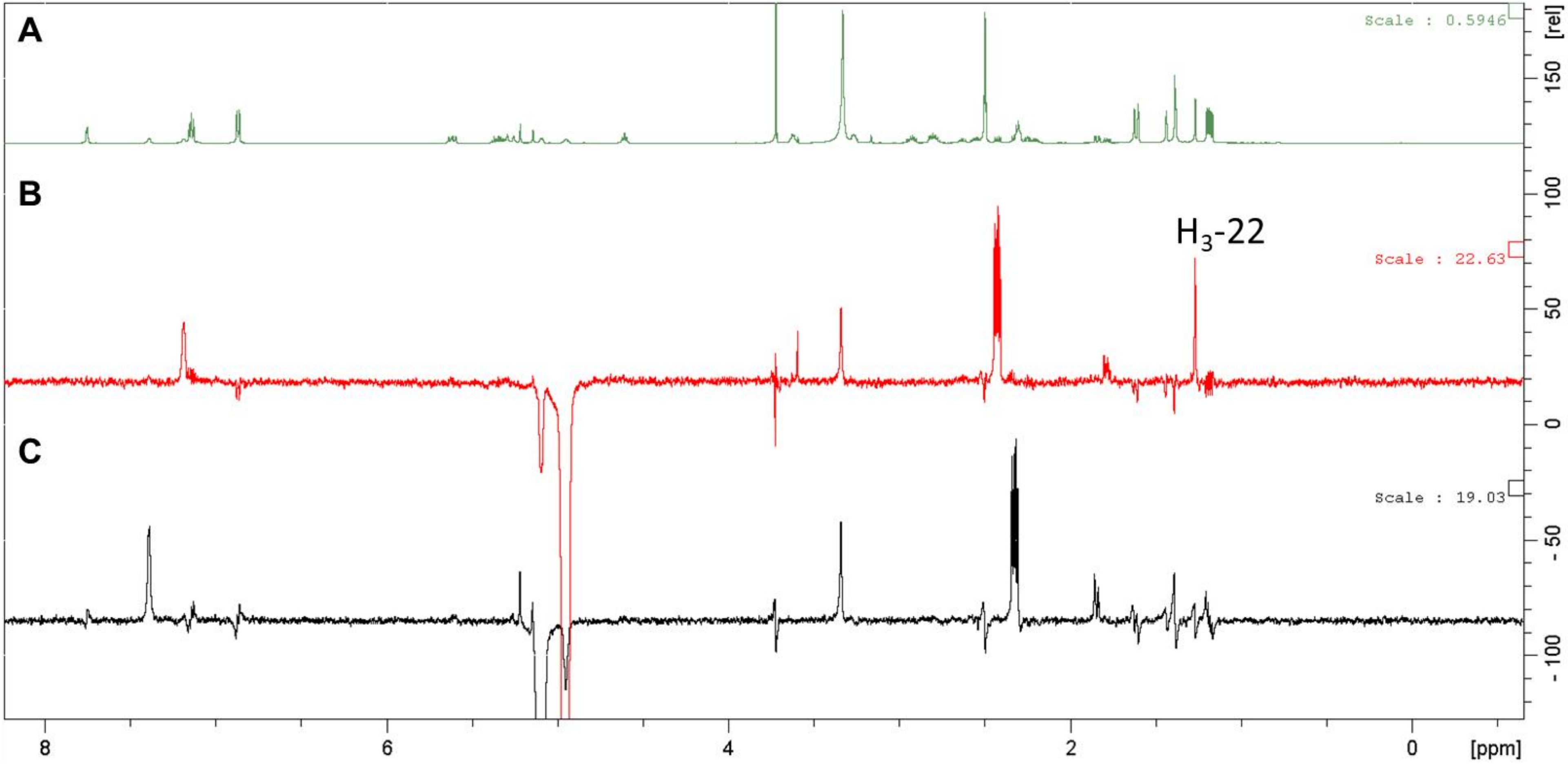

Figure S32. 2D-NOESY spectrum of perochalasin B (**2a**) and *epi*-perochalasin B (**2b**) (600 MHz, DMSO-d_6).

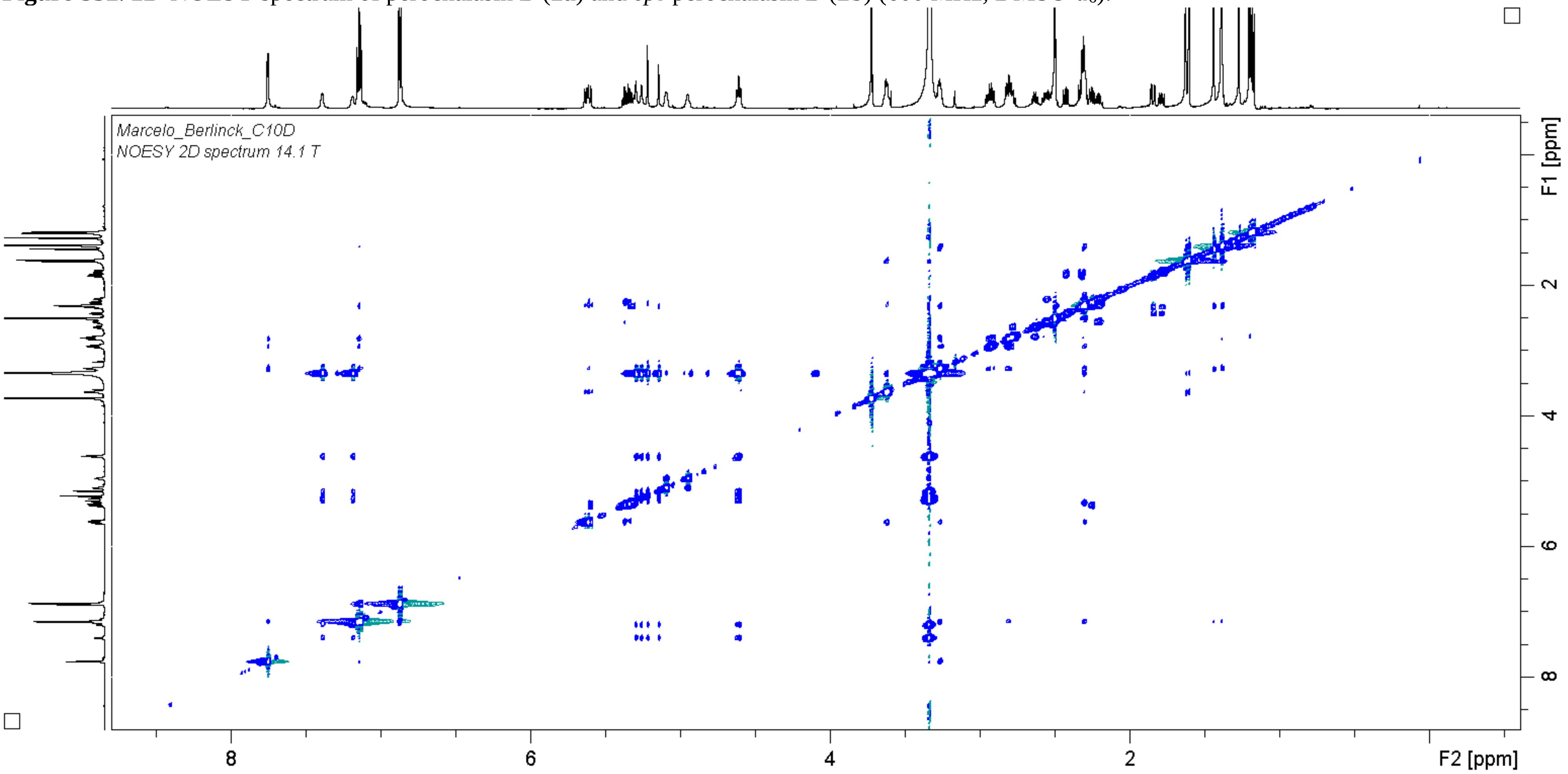

Figure S33. Expansion of 2D-NOESY spectrum of perochalasin B (**2a**) and *epi*-perochalasin B (**2b**) (600 MHz, DMSO-d_6). NOE interaction of H-3 (δ_H 3.27) with H-13 (δ_H 5.62).

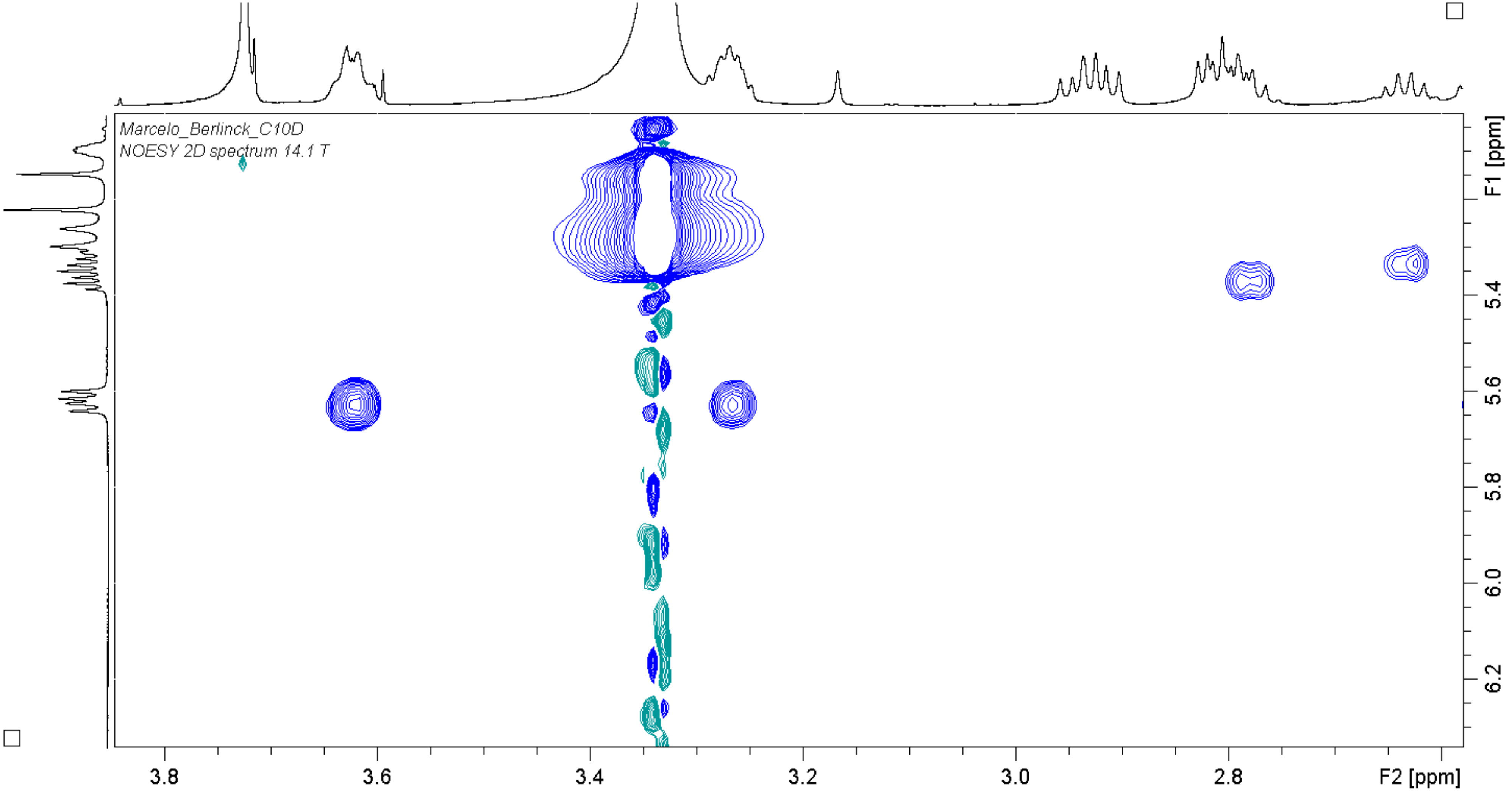

Figure S34. UV spectrum (150 µg/mL, MeOH) of perochalasin B (**2a**) and *epi*-perochalasin B (**2b**).

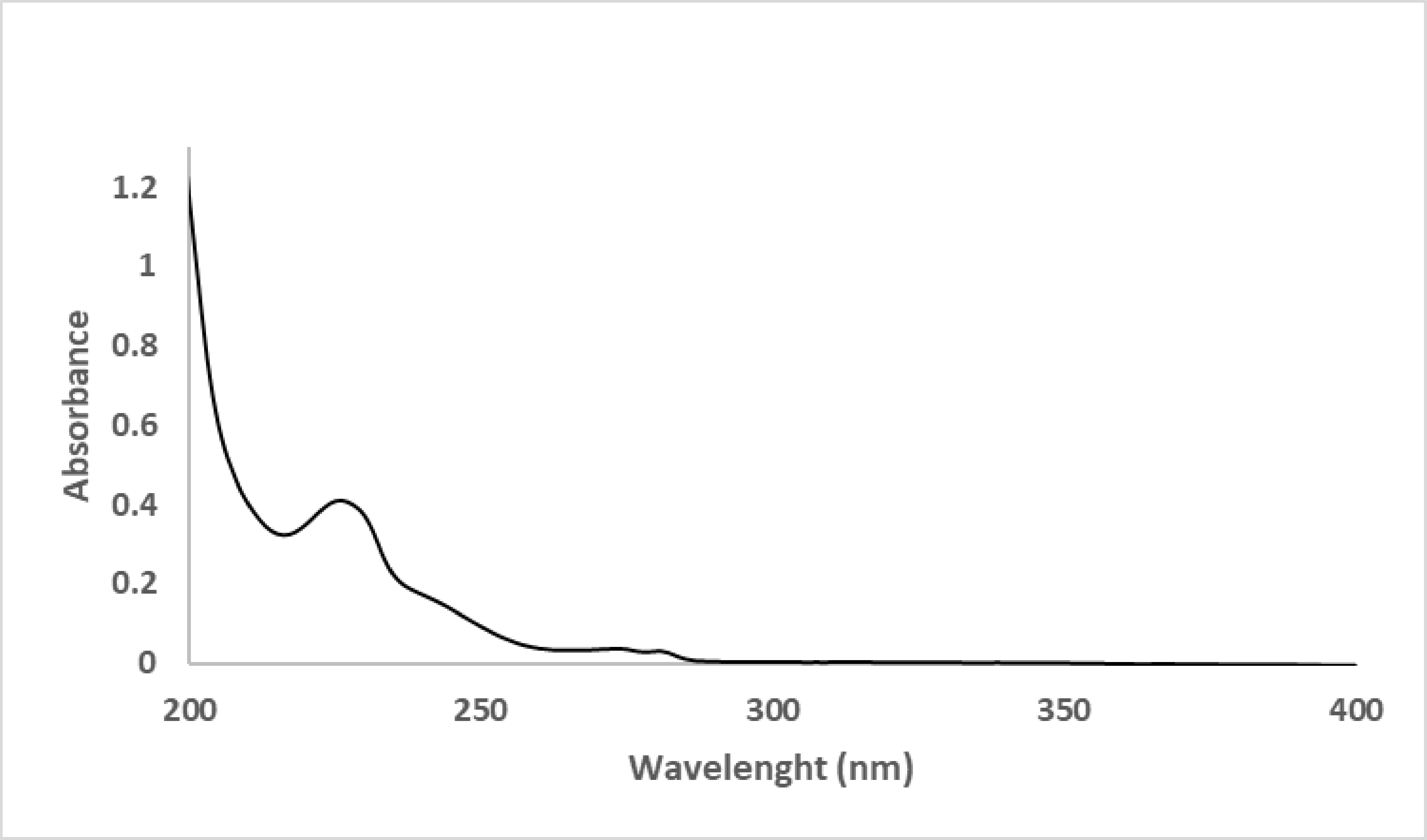

Figure S35. ECD spectrum (150 µg/mL, MeOH) of perochalasin B (**2a**) and *epi*-perochalasin B (**2b**).

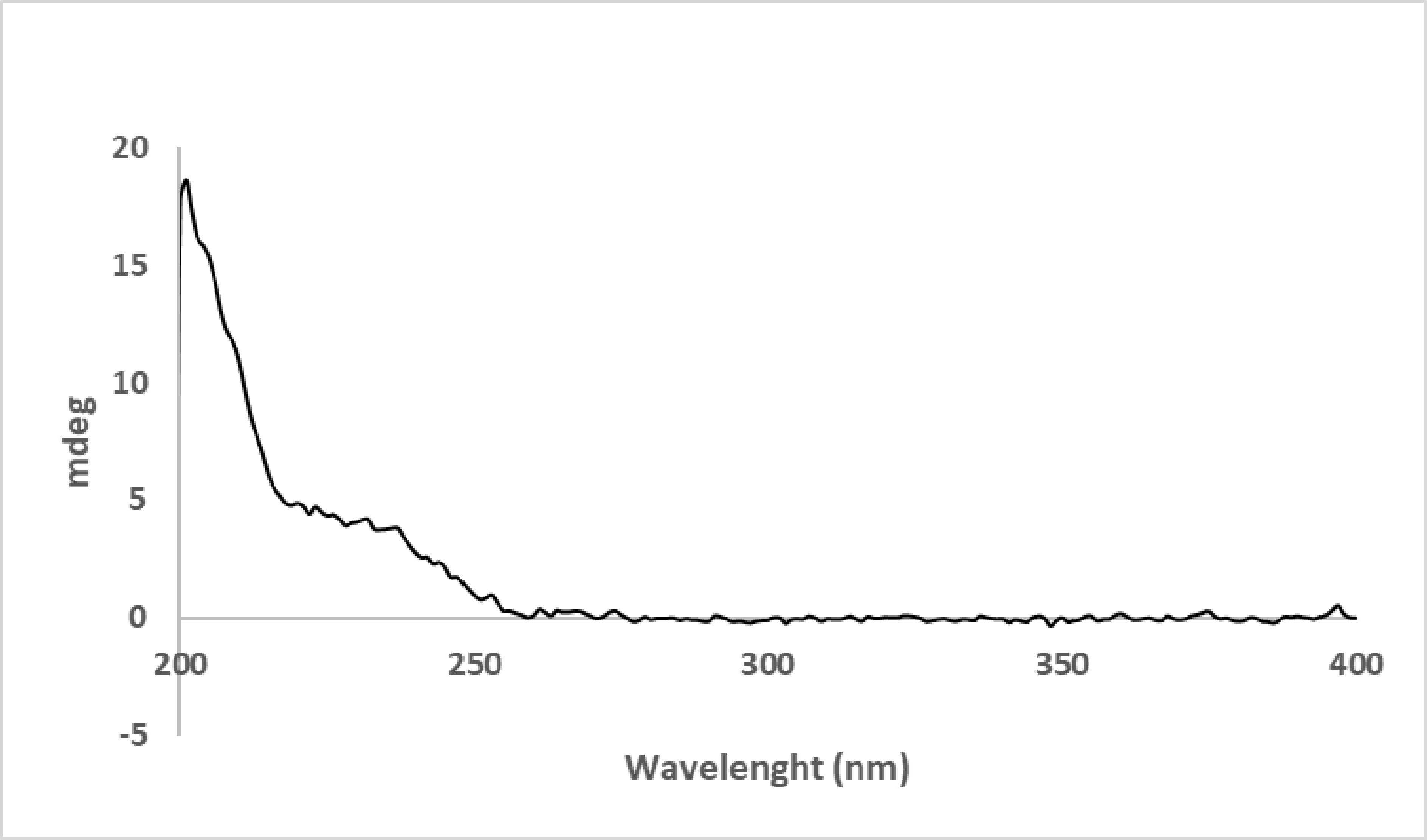

Figure S36. ^{1}H NMR spectrum of perochalasin C (**3a**) and *epi*-perochalasin C (**3b**) (600 MHz, DMSO-d_6).

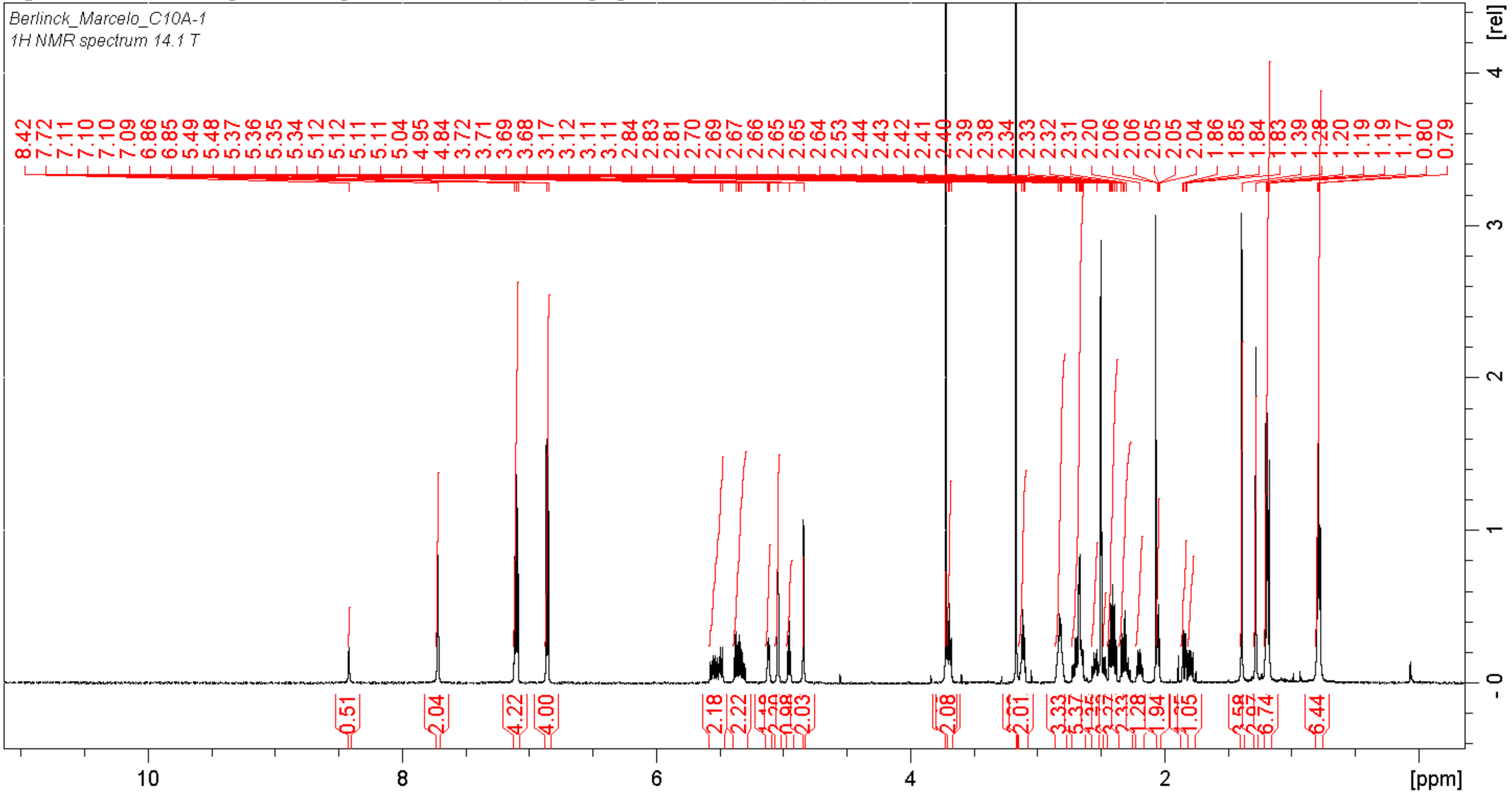

Signals at δ_H 8.46 and 3.16 are assigned to traces of formic acid and MeOH, used in HPLC purification.

Figure S37. Expansion of the ^{1}H NMR spectrum of perochalasin C (**3a**) and *epi*-perochalasin C (**3b**) (600 MHz, DMSO-d_6).

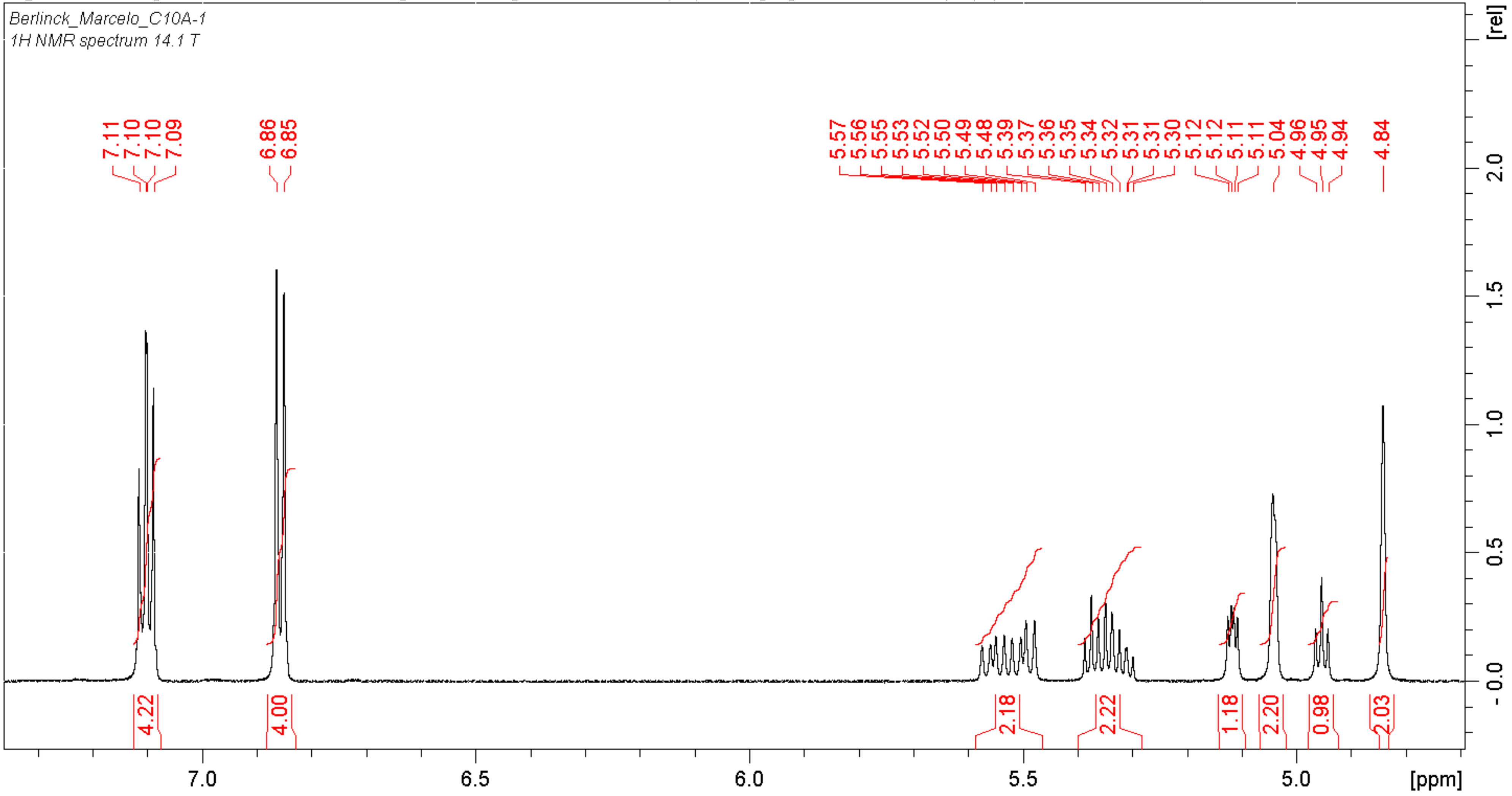

Figure S38. Expansion of the ^{1}H NMR spectrum of perochalasin C (**3a**) and *epi*-perochalasin C (**3b**) (600 MHz, DMSO-d_6).

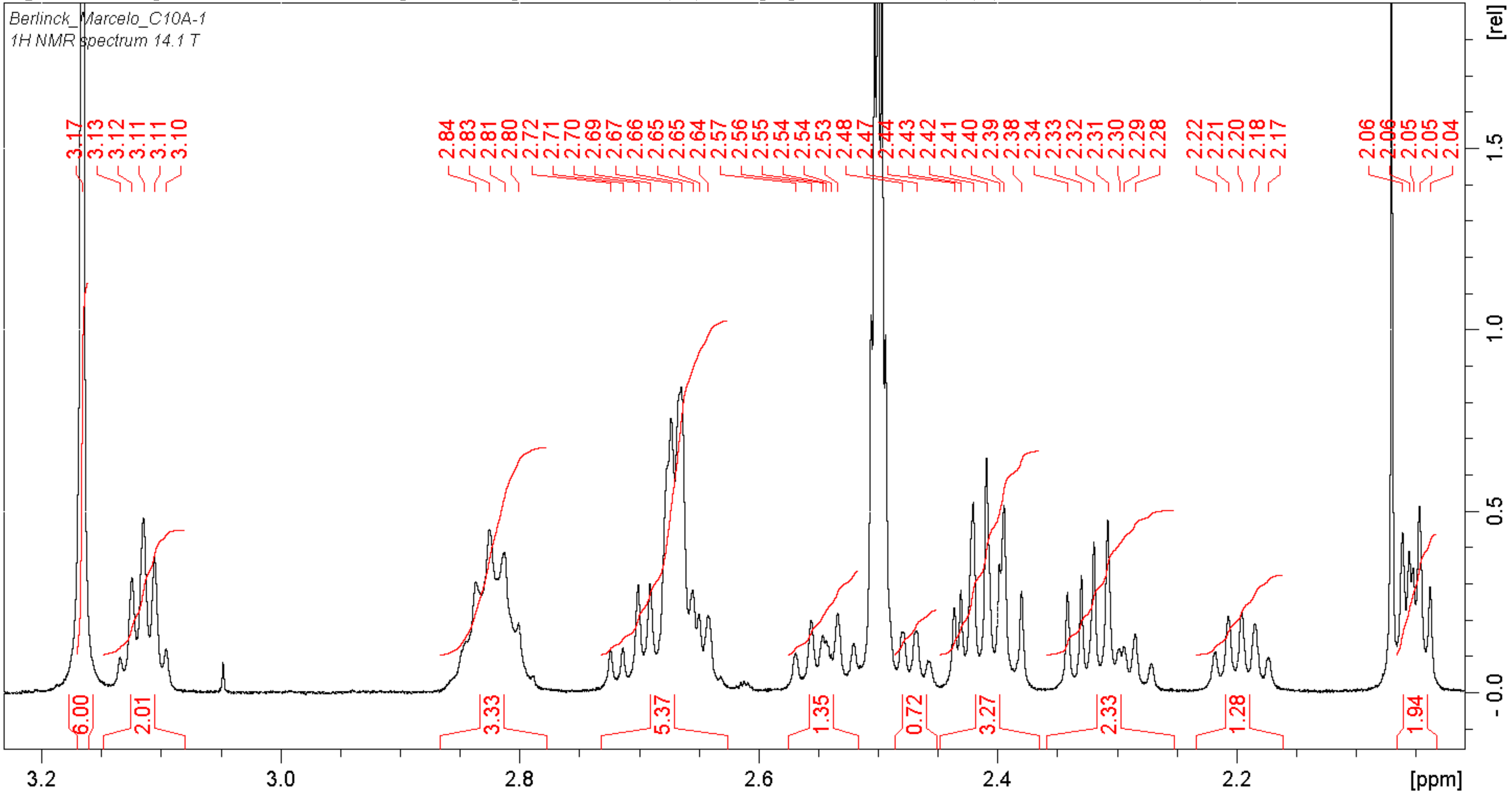

Figure S39. Expansion of the ^{1}H NMR spectrum of perochalasin C (**3a**) and *epi*-perochalasin C (**3b**) (600 MHz, DMSO-d_6).

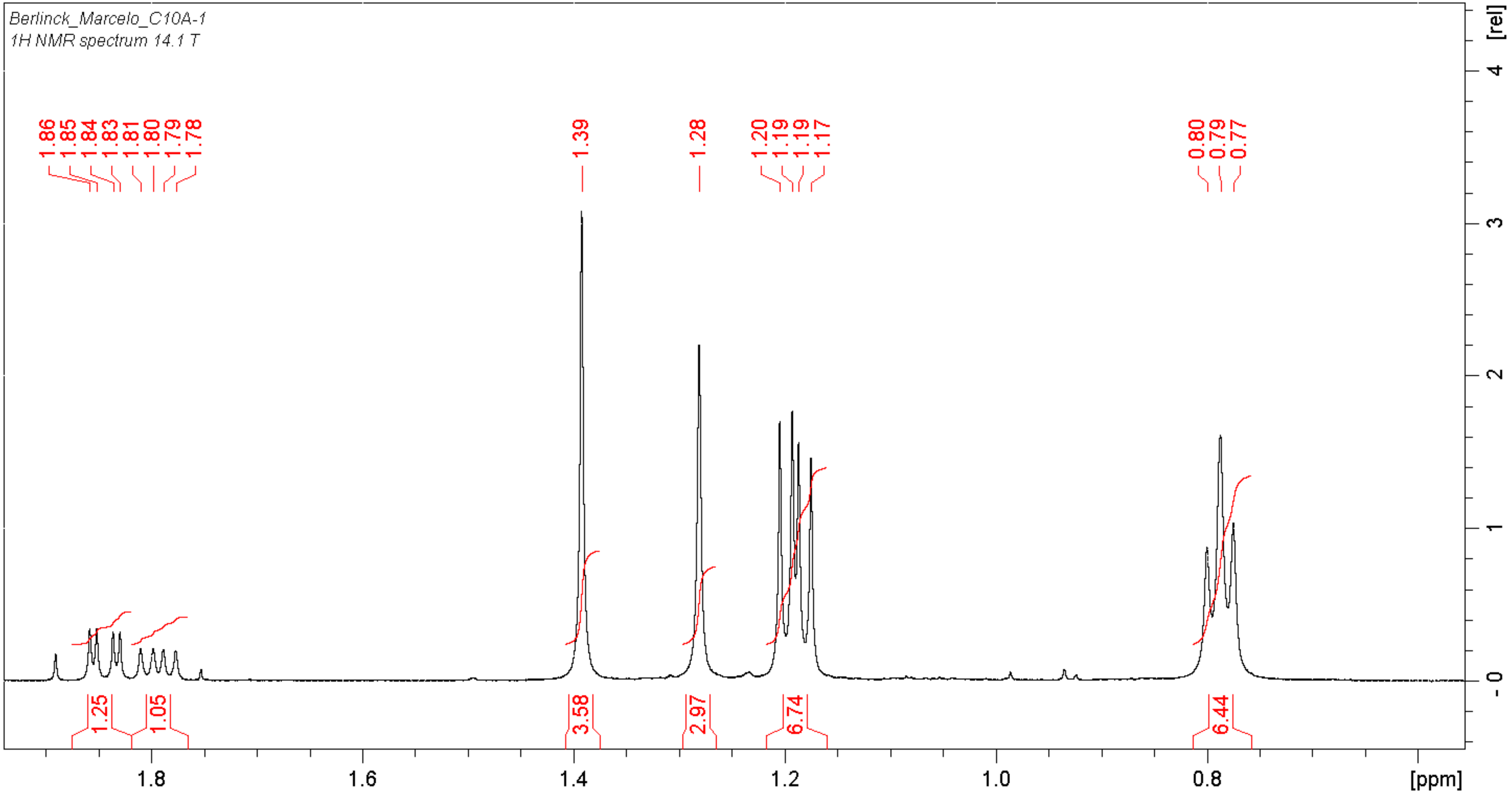

Figure S40. ¹³C NMR spectrum of perochalasin C (**3a**) and *epi*-perochalasin C (**3b**) (150 MHz, DMSO-d_6).

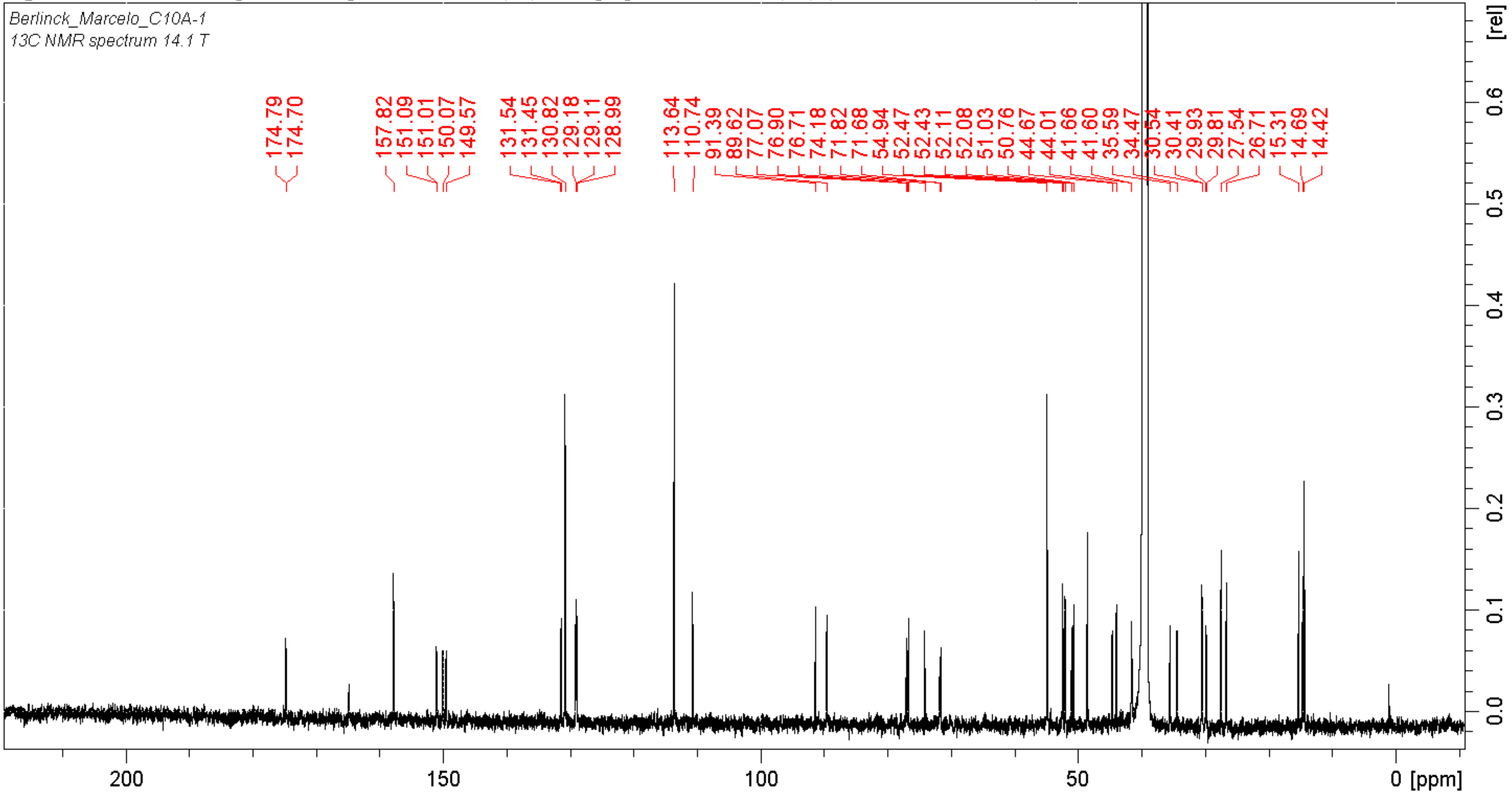

Signal at δ_C 165.4 and δ_C 48.6 are assigned to residual formic acid and MeOH, used during HPLC purification.

Figure S41. HSQC spectrum of perochalasin C (**3a**) and *epi*-perochalasin C (**3b**) (^{1}H: 600 MHz, ^{13}C: 150 MHz; DMSO-d_6).

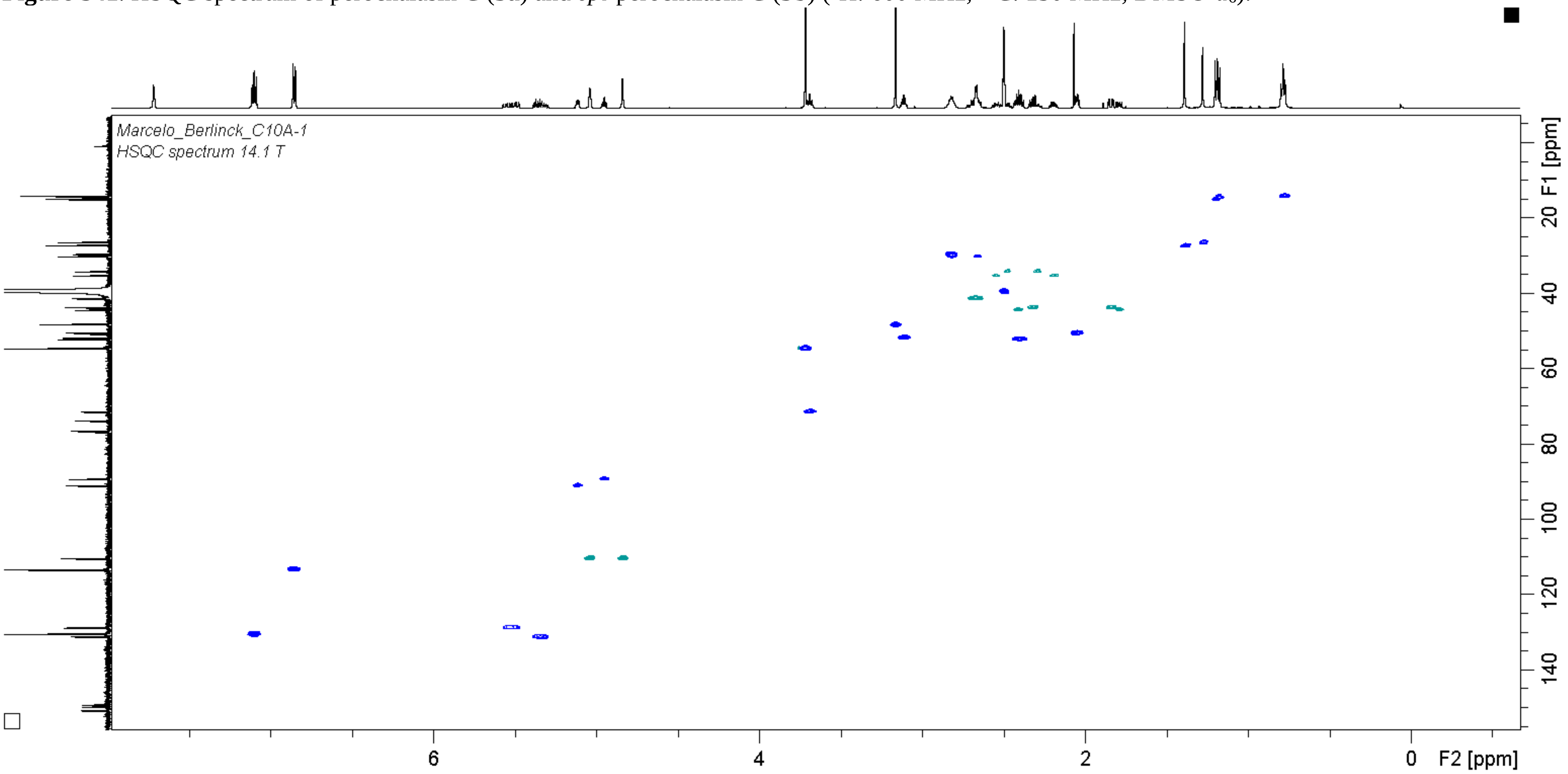

Figure S42. HMBC spectrum of perochalasin C (**3a**) and *epi*-perochalasin C (3**b**) (^{1}H: 600 MHz, ^{13}C: 150 MHz; DMSO-d_6).

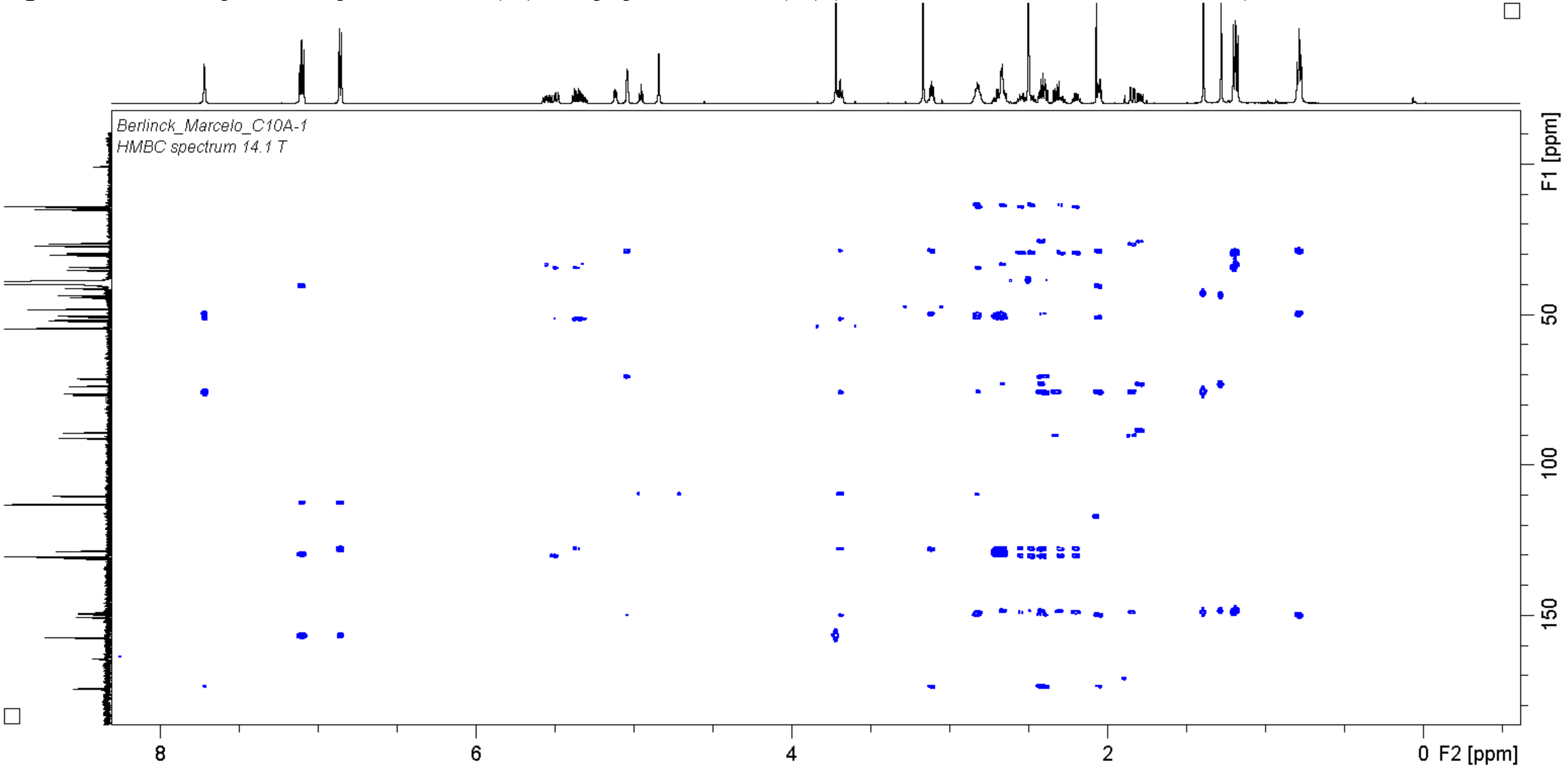

Figure S43. COSY spectrum of perochalasin C (**3a**) and *epi*-perochalasin C (**3b**) (600 MHz, DMSO-d_6).

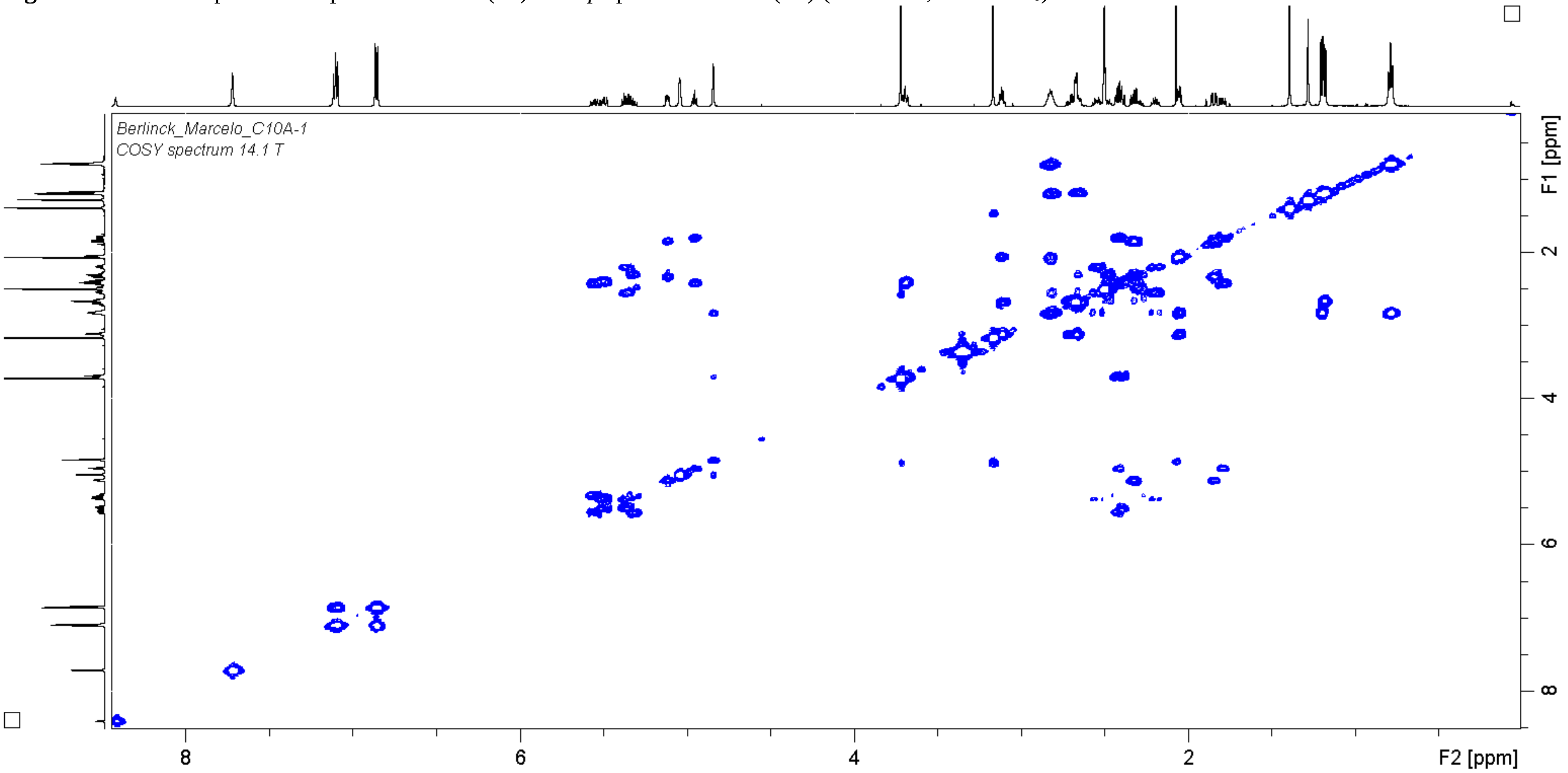

Figure S44. (+)-HRESIMS spectrum of perochalasin C (**3a**) and *epi*-perochalasin C (**3b**).

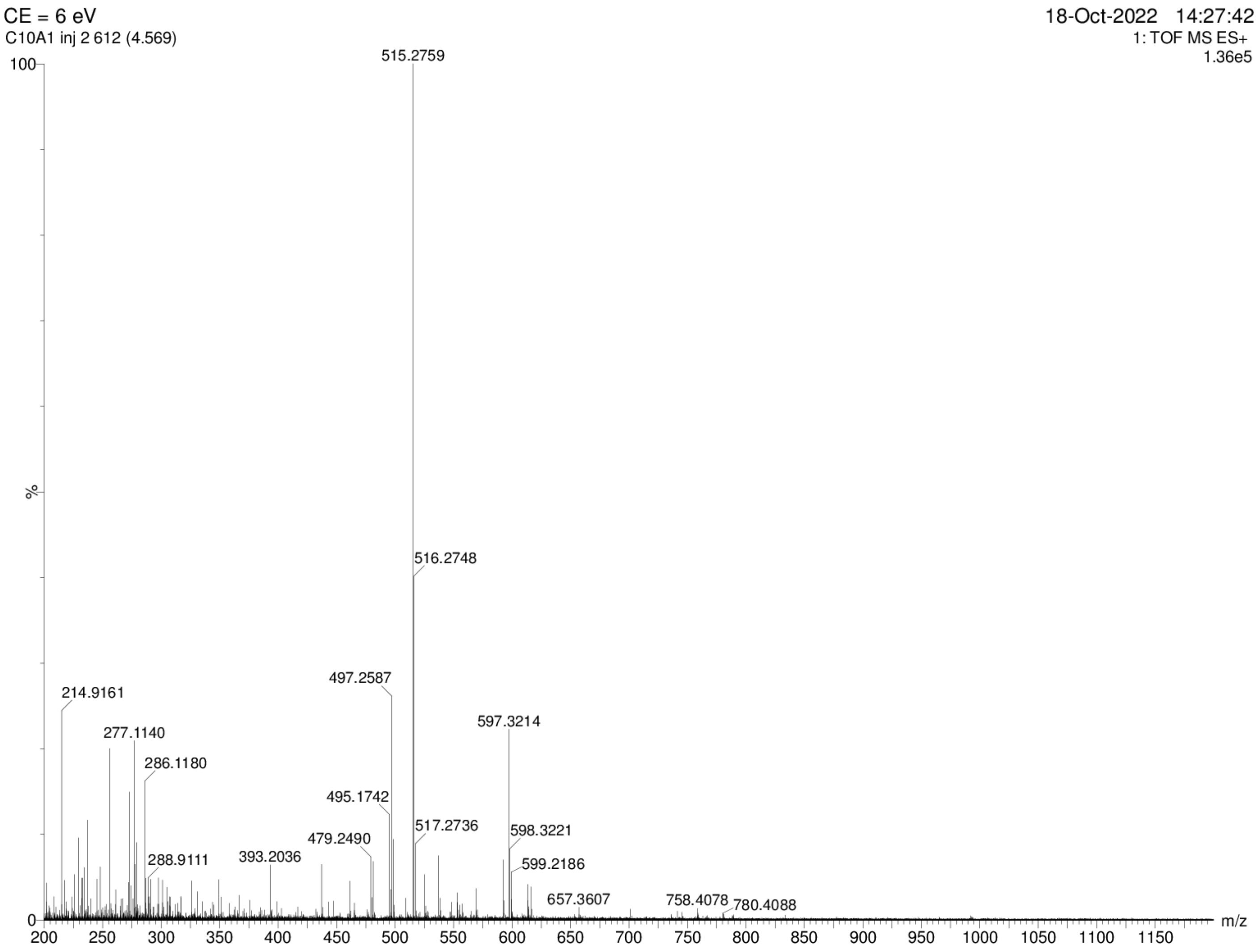

Figure S45. 1D-NOESY spectrum of perochalasin C (**3a**) and *epi*-perochalasin C (**3b**) (600 MHz, DMSO-d_6). ^{1}H NMR spectrum (**A**), and irradiation at δ_H 2.40 (**B**).

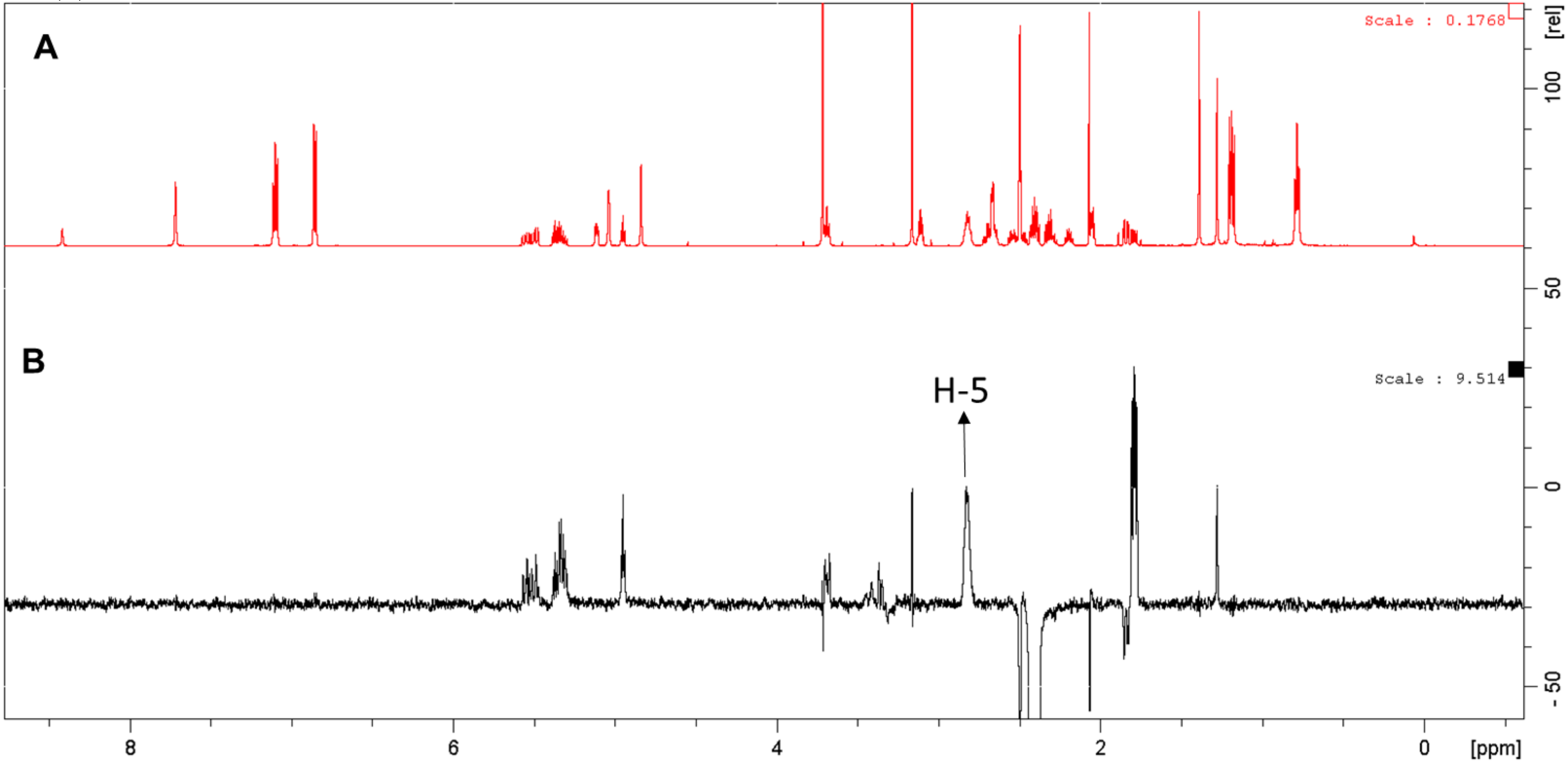

Figure S46. 1D-NOESY spectrum of perochalasin C (**3a**) and *epi*-perochalasin C (**3b**) (600 MHz, DMSO-d_6). ^{1}H NMR spectrum (**A**), and irradiation at δ_H 3.11 (**B**).

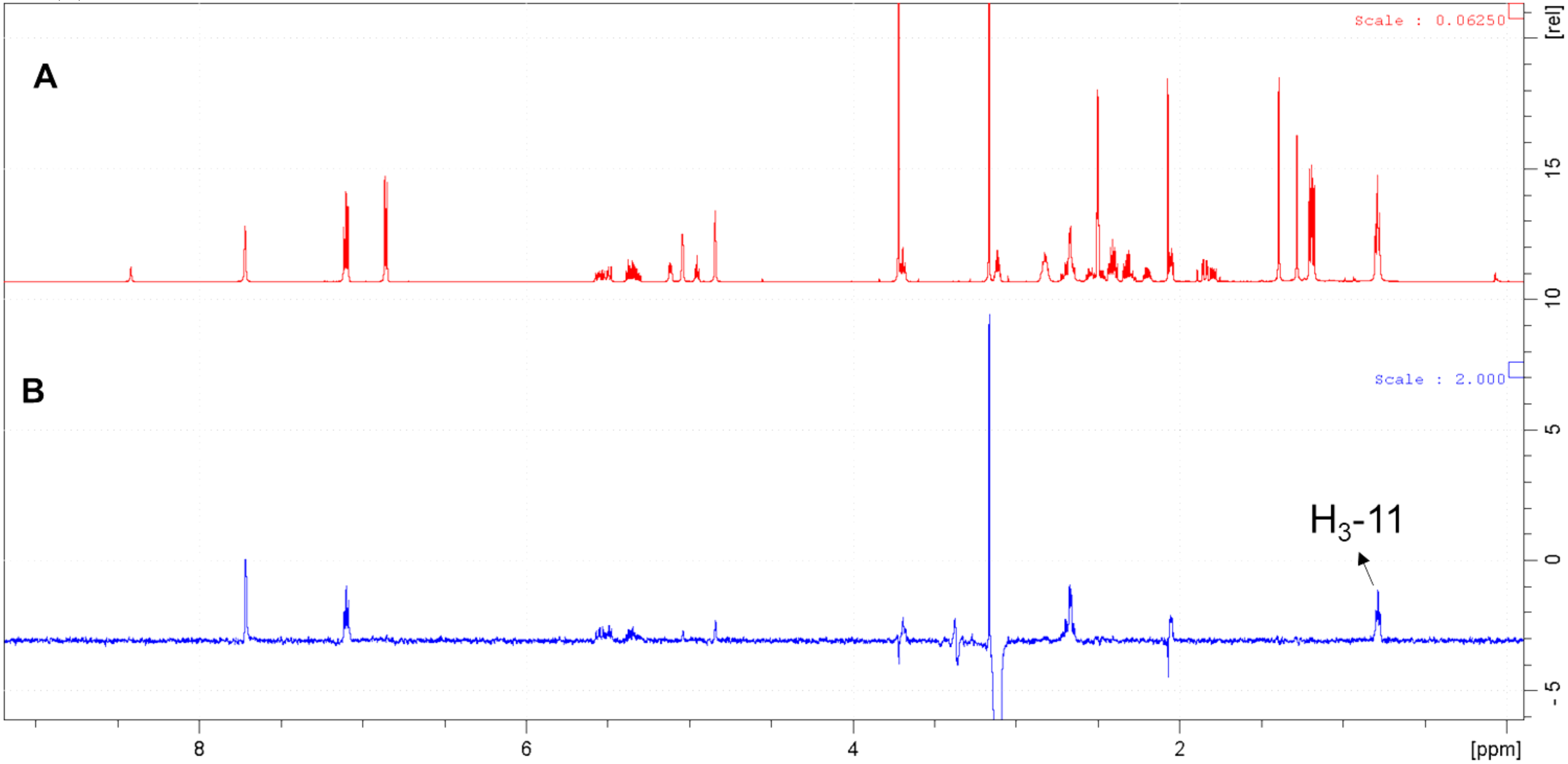

Figure S47. 1D-NOESY spectrum of perochalasin C (**3a**) and *epi*-perochalasin C (**3b**) (600 MHz, DMSO-d_6). ^1H NMR spectrum (**A**), and irradiation at δ_H 5.12 (**B**) and irradiation at δ_H 4.95 (**C**).

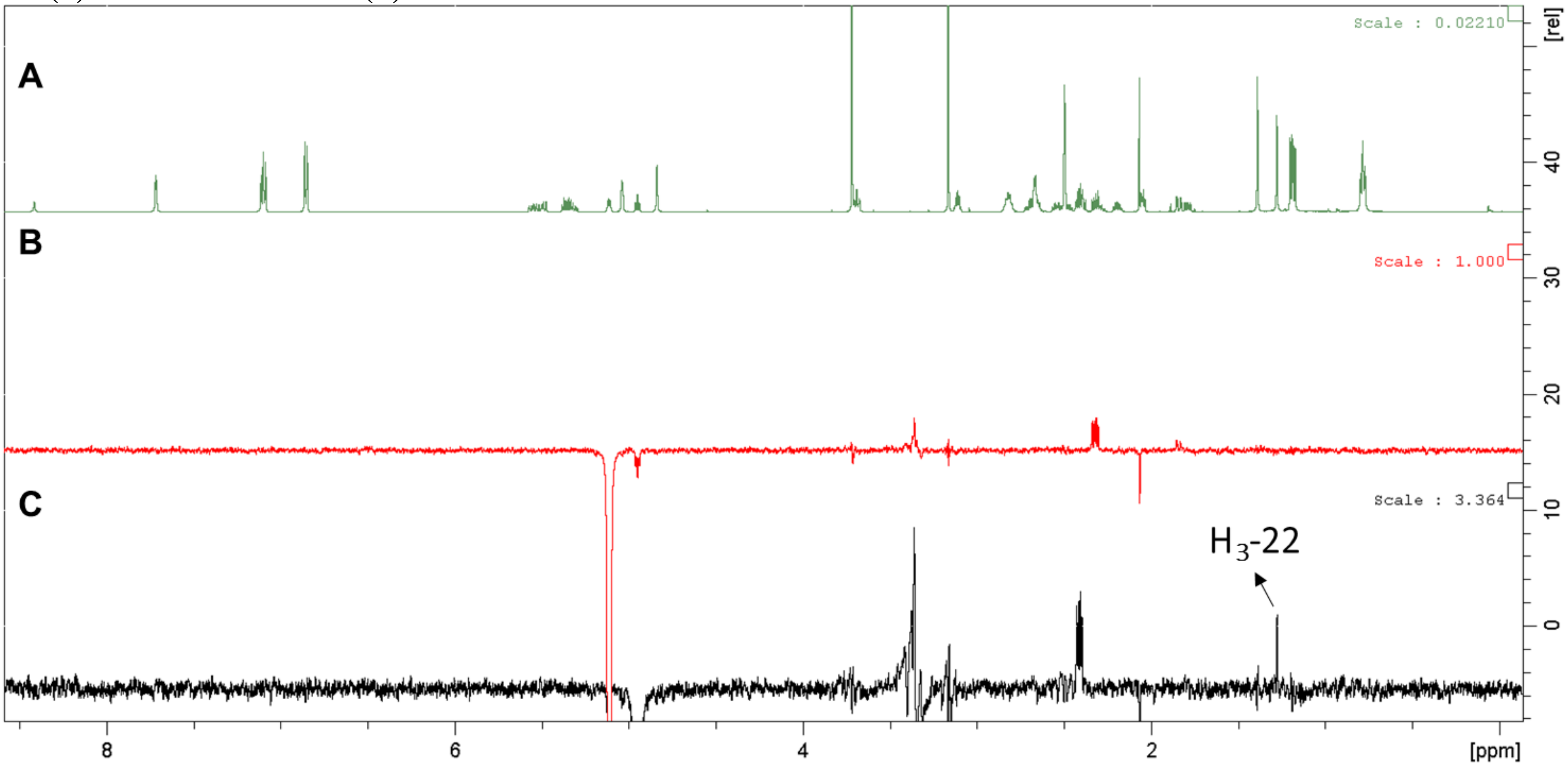

Figure S48. UV spectrum (75 µg/mL, MeOH) of perochalasin C (**3a**) and *epi*-perochalasin C (**3b**).

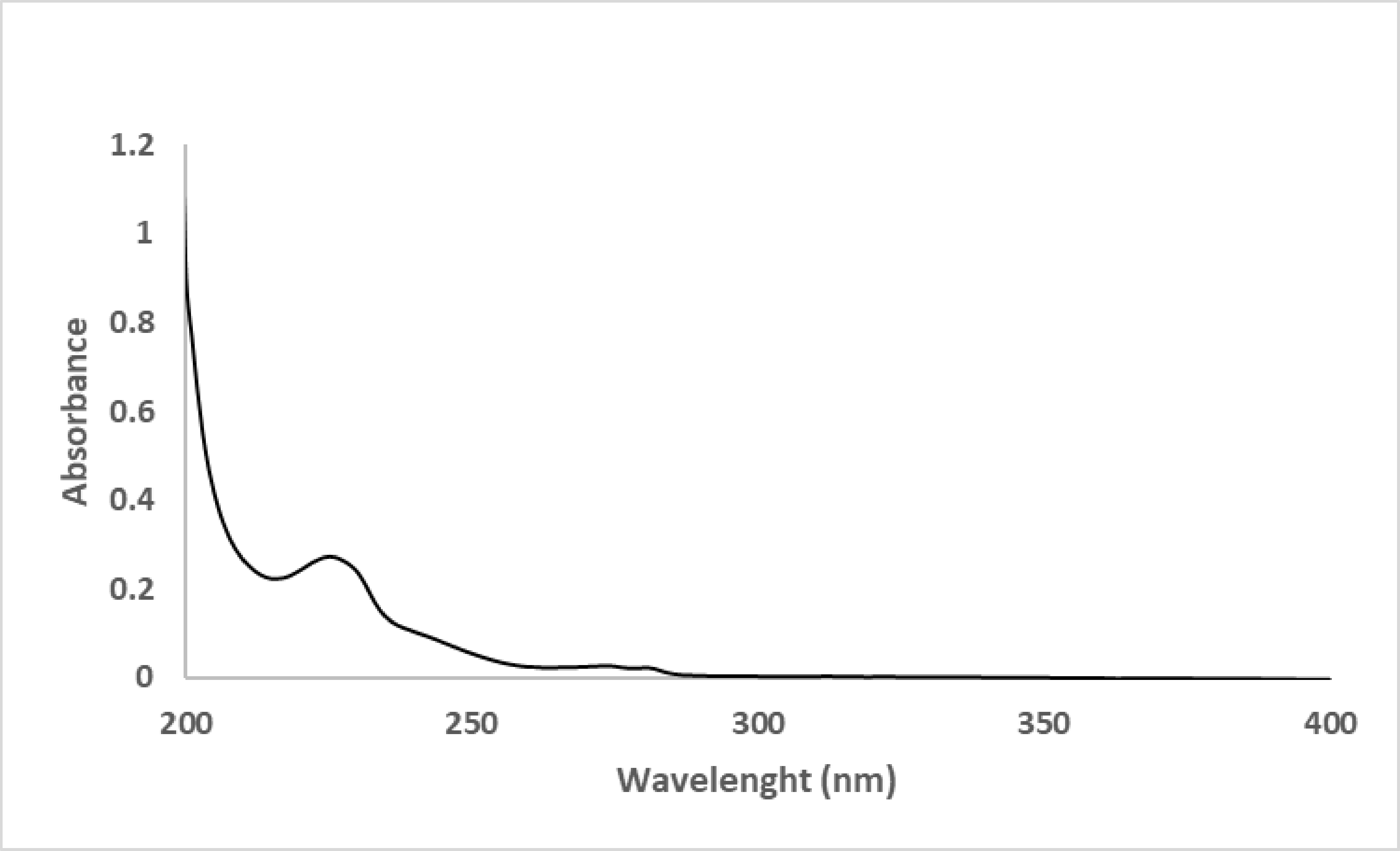

Figure S49. ECD spectrum (75 µg/mL, MeOH) of perochalasin C (**3a**) and *epi*-perochalasin C (**3b**).

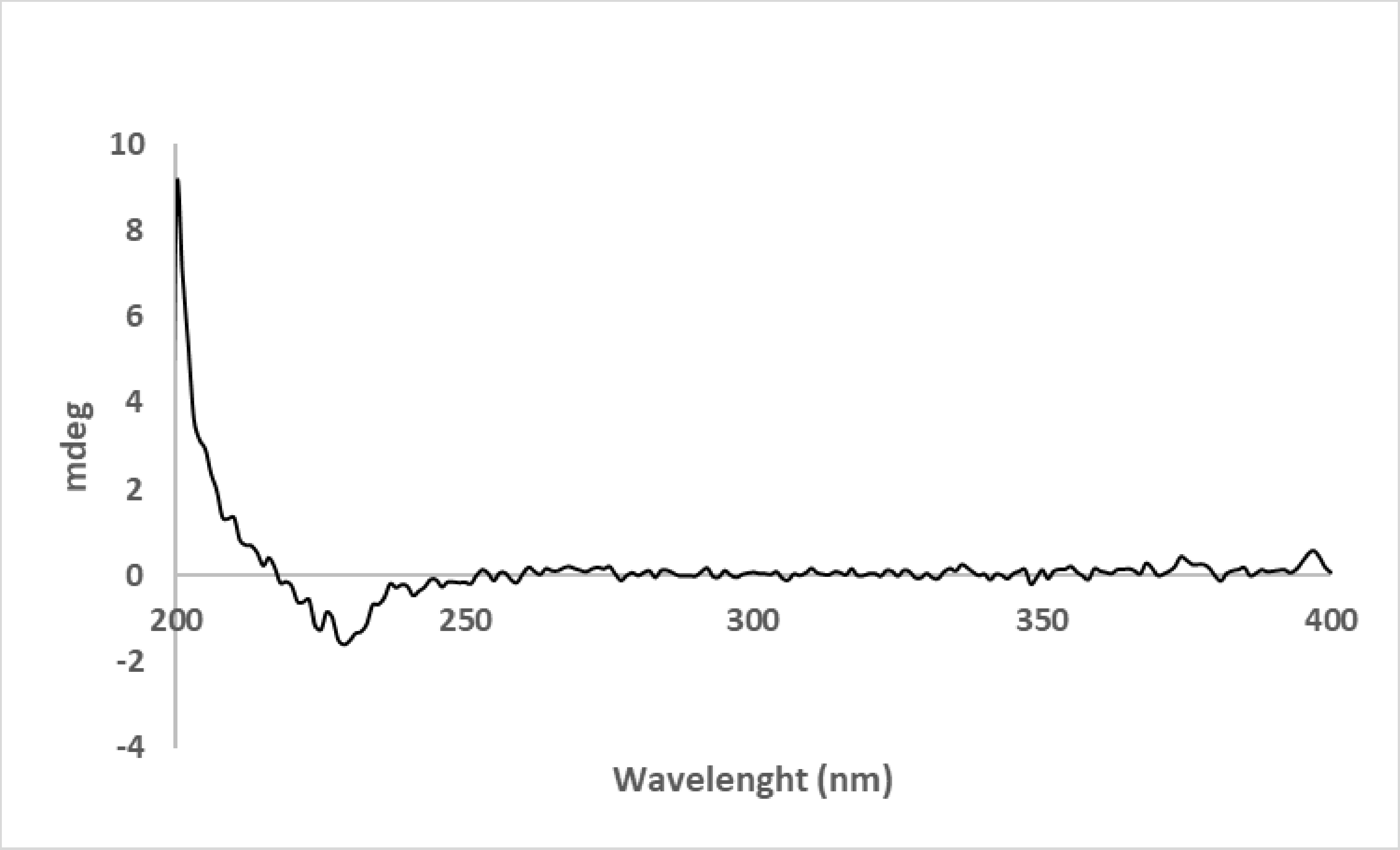

Figure S50. HPLC-MS analyses of samples from feeding studies of *Peroneutypa* sp. using [^{15}N]-hydroxylamine, as well as the standards **2a+2b** and **3a+3b** for comparison.

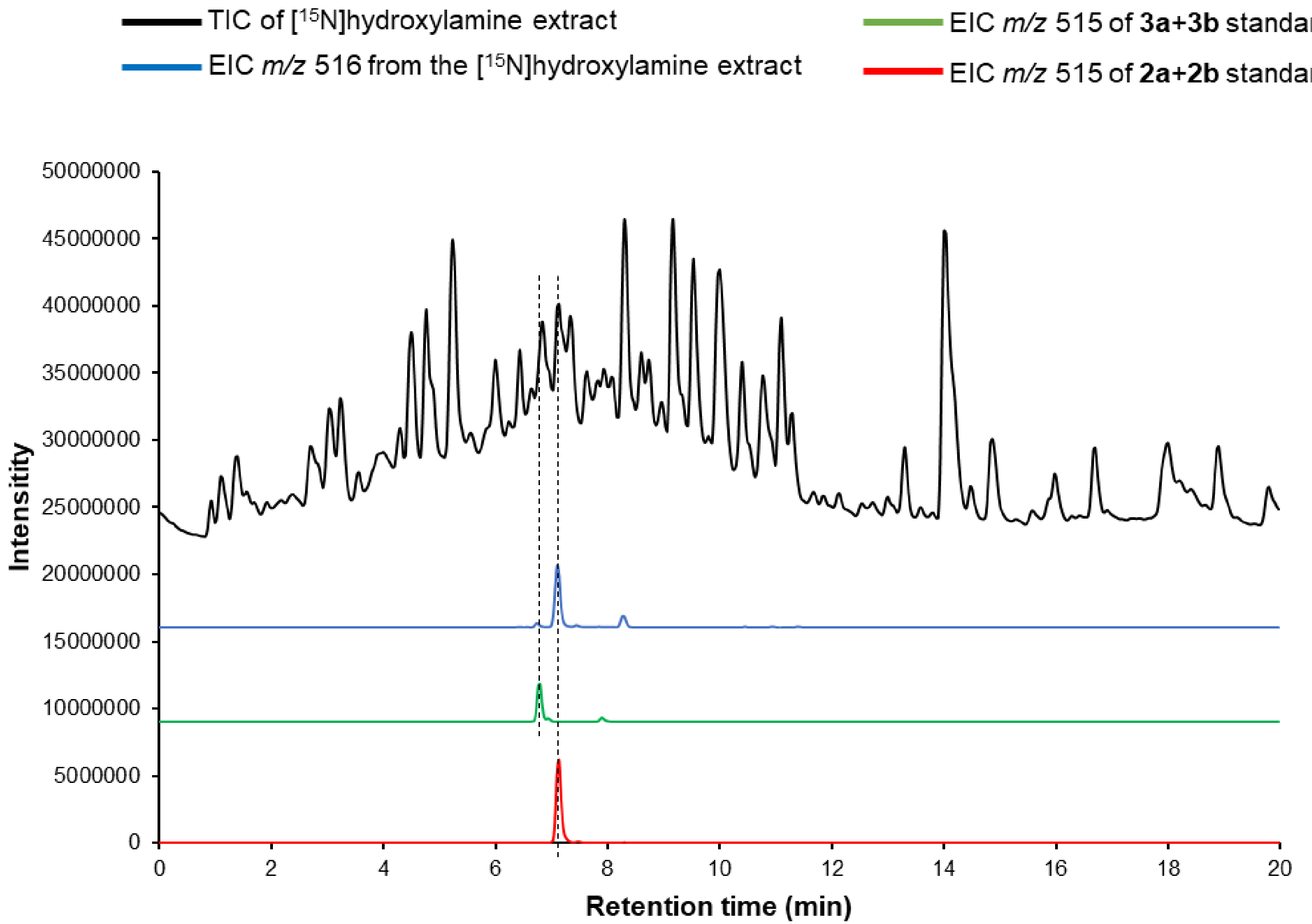

Figure S51. MS spectra of *Peroneutypa* sp. feeding studies using: [^{15}N]-glycine (**B**) and [^{15}N]-hydroxylamine (**C**), as well as the control (**A**). MS spectra from the extracts with the retention time of 7.1 min related to the standard **2a+2b**.

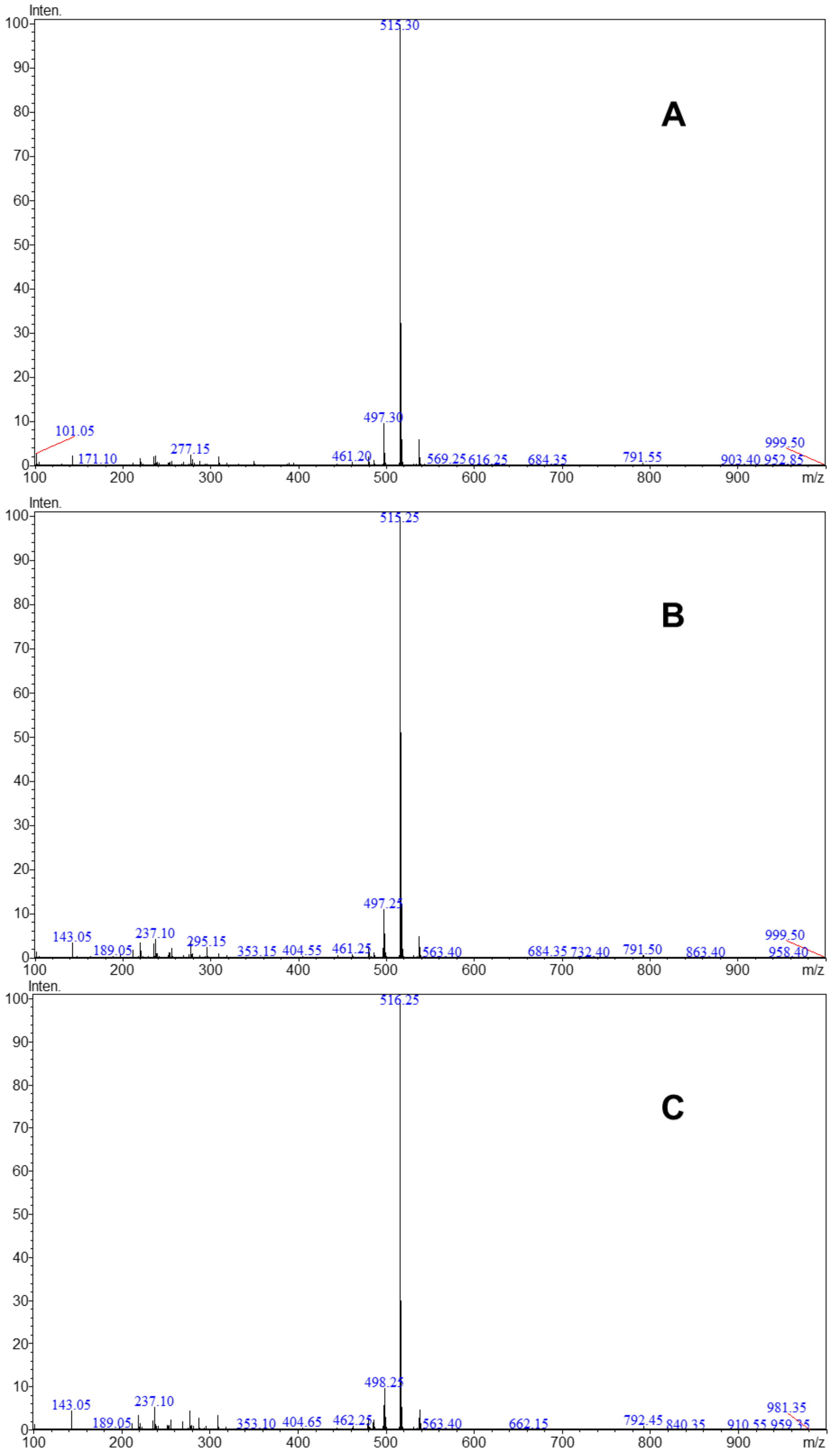

Figure S52. MS spectra of *Peroneutypa* sp. feeding studies using: ^{15}N-glycine (**B**) and ^{15}N-hydroxylamine (**C**), as well as the control (**A**). MS spectra from the extracts with the retention time of 6.8 min related to the standard **3a+3b**.

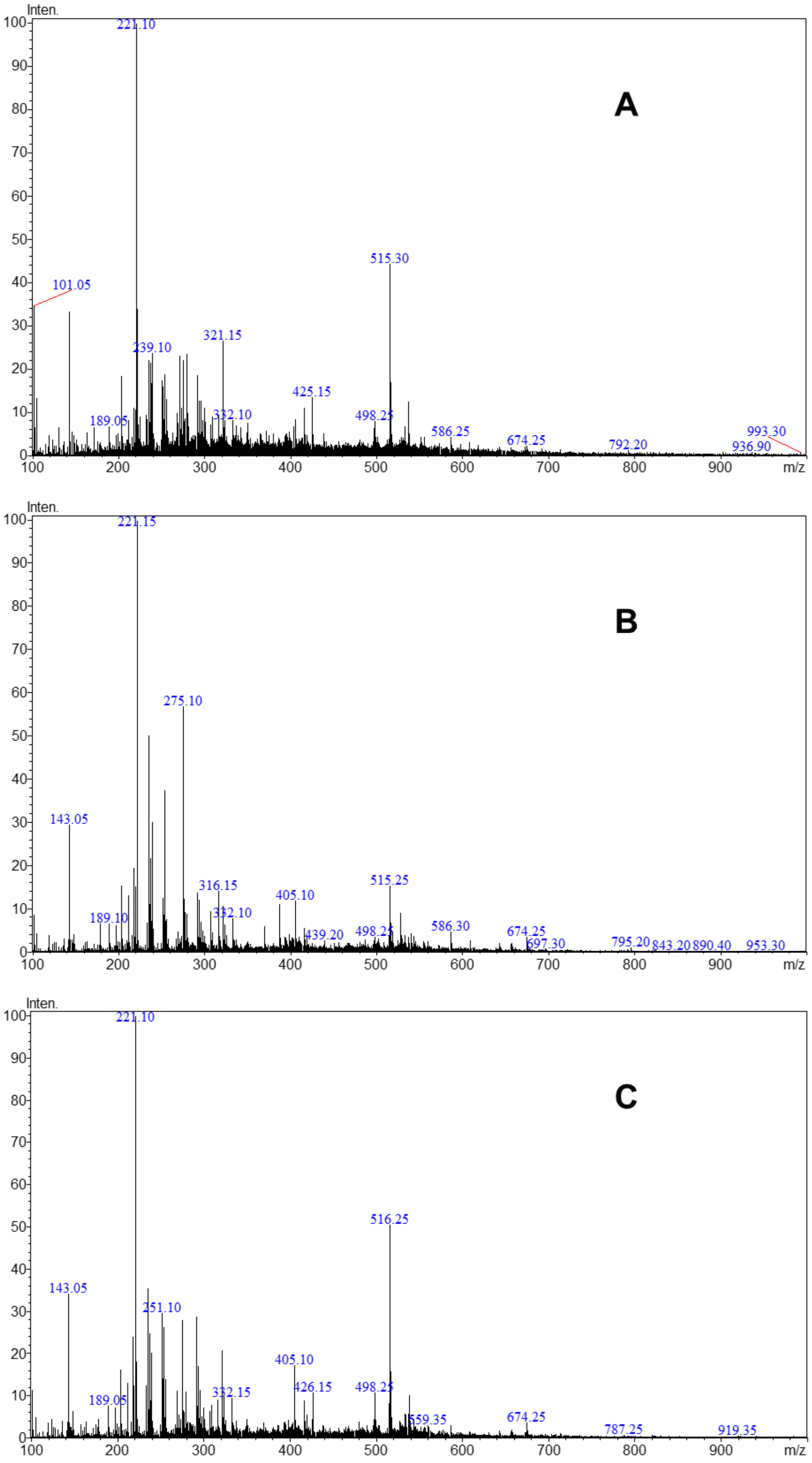

Figure S53. MS spectrum of the standard **1** (R_t = 7.2 min) by LC-MS. Positive ionization ESI mode.

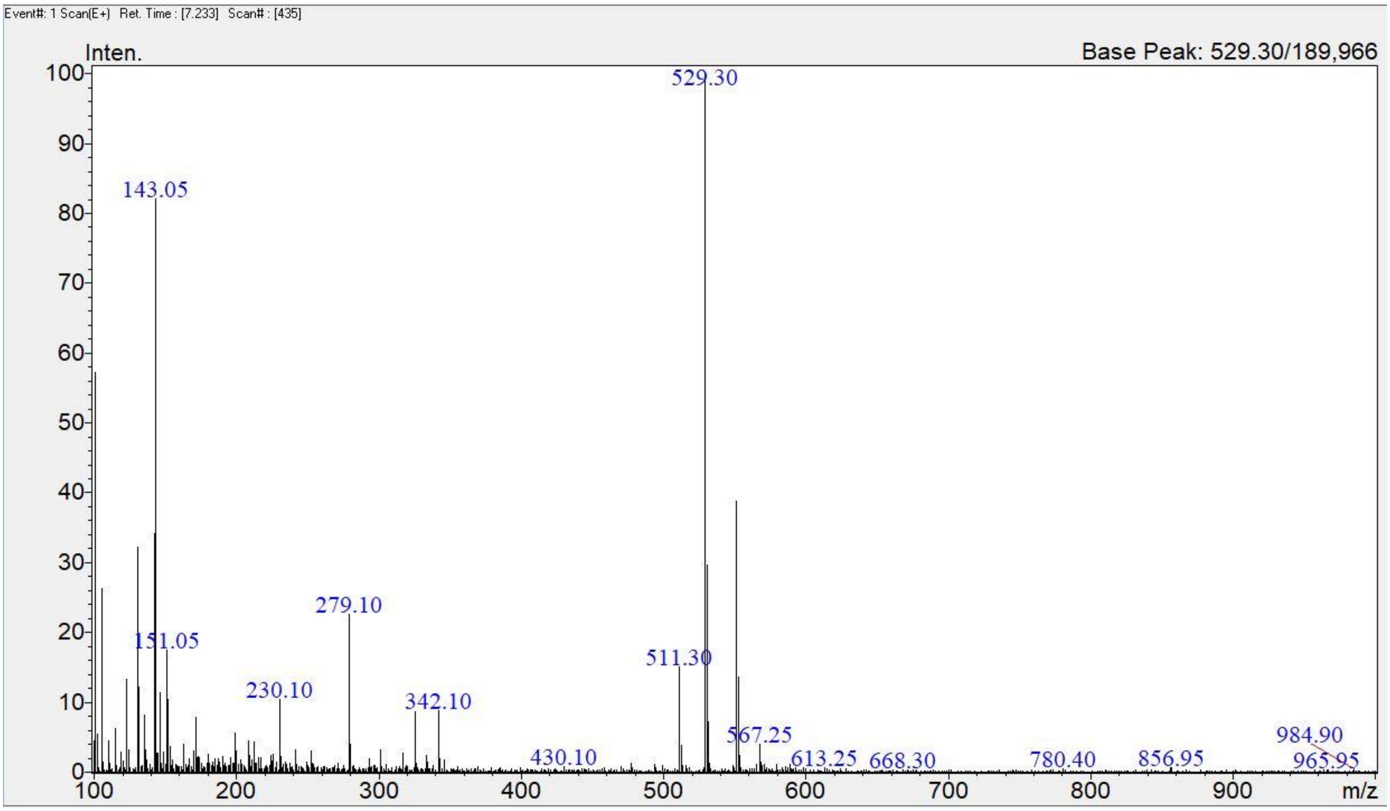

Figure S54. MS spectrum of the standard **2a+2b** (R_t = 7.1 min) by LC-MS. Positive ionization ESI mode.

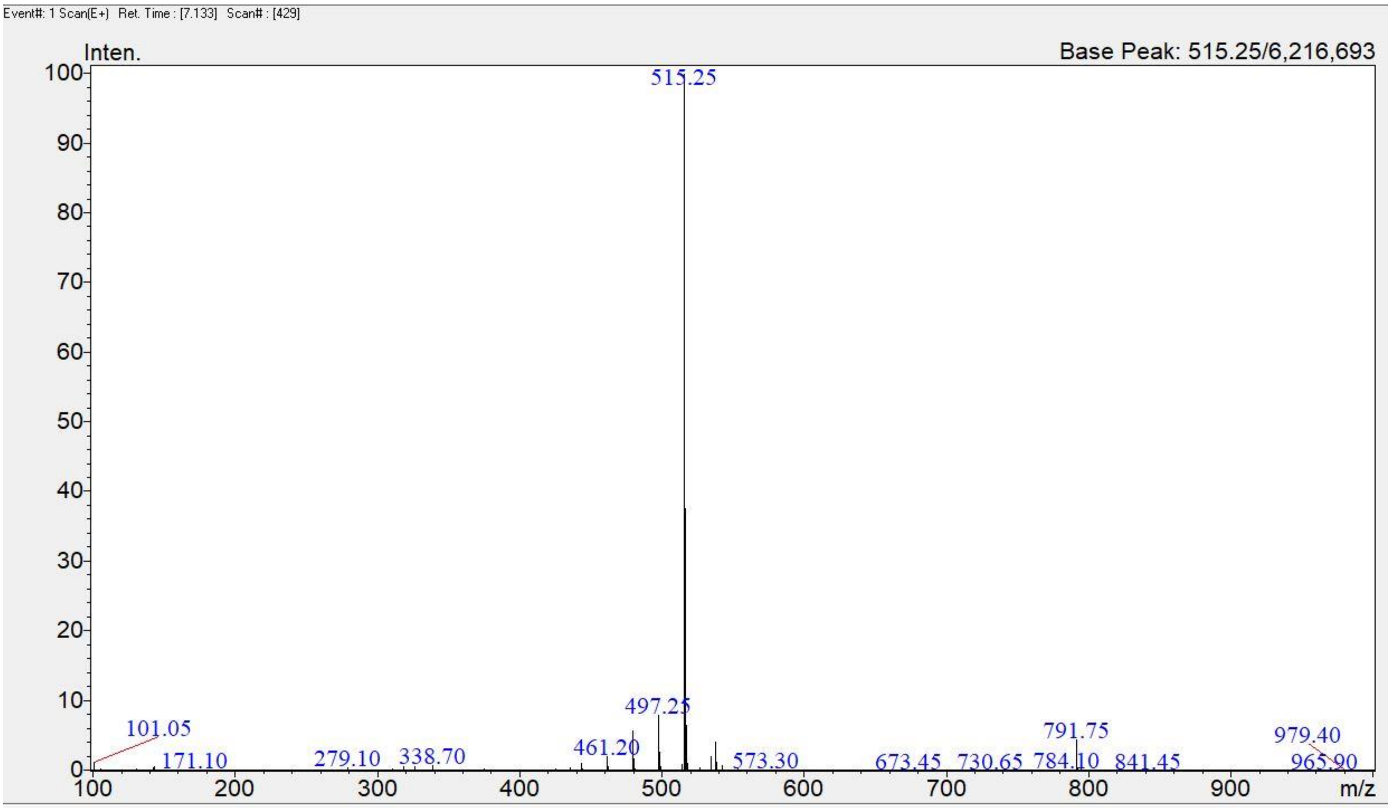

Figure S55. MS spectrum of the standard **3a+3b** (R_t = 6.8 min) by LC-MS. Positive ionization ESI mode.

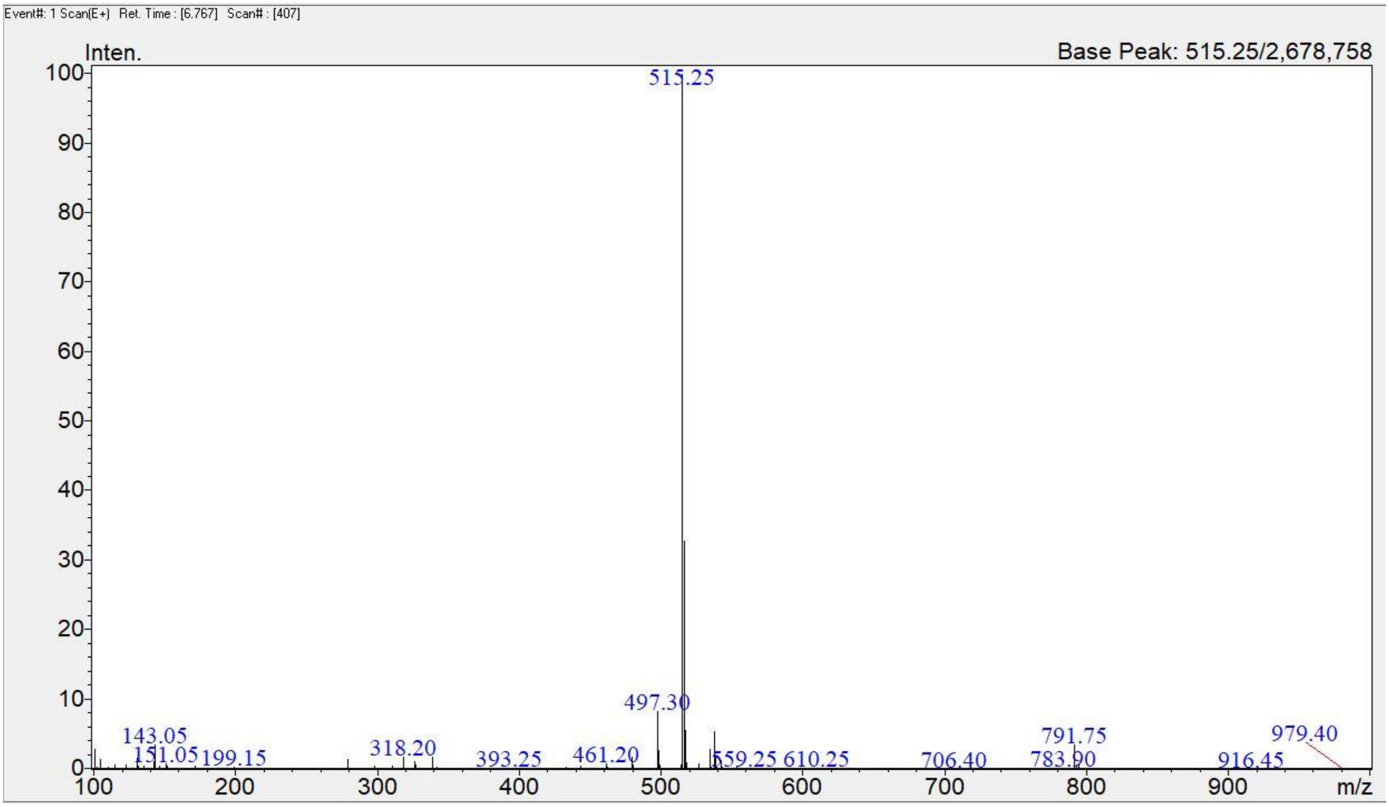

Figure S56. Chromatogram of the standards **1** (R_t = 7.2 min), **2a+2b** (R_t = 7.1 min) and **3a+3b** (R_t = 6.8 min) by HPLC-PDA monitored at 190 nm.

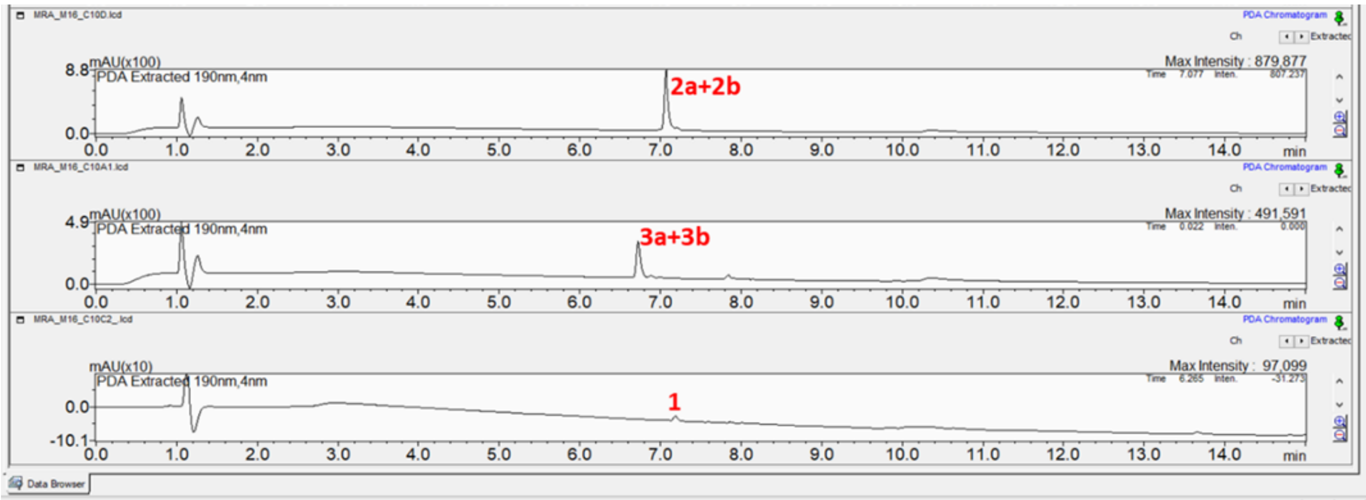

Figure S57. MS spectra of the investigation of phenochalasin B in the presence of [^{15}N]-hydroxylamine and [^{2}H]-hydroxylamine evaluated at 1 and 6 days. Comparison with the standard **3a+3b**.

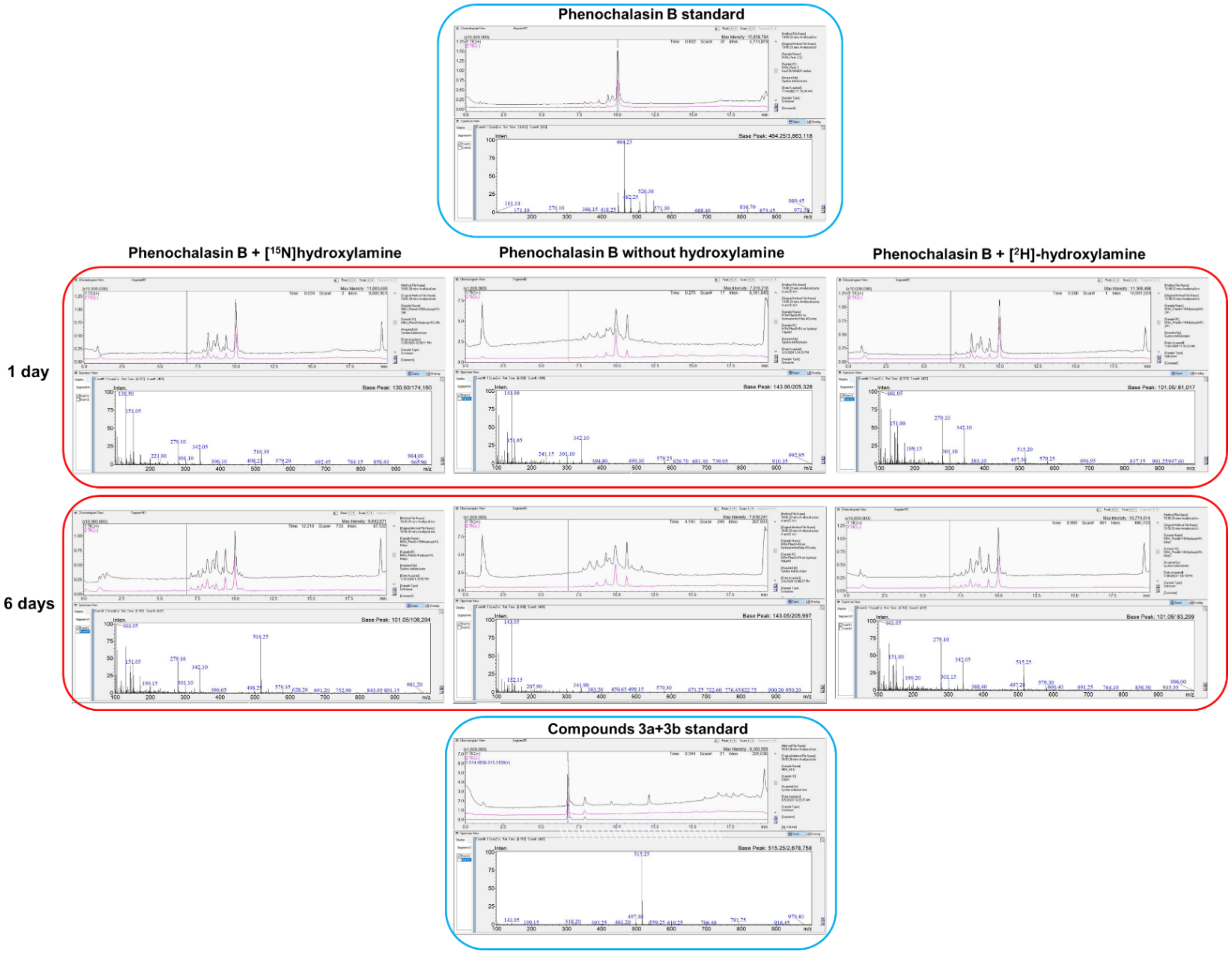

Figure 58. EIC of the investigation of phenochalasin B in the presence of [^{15}N]-hydroxylamine and [^{2}H]-hydroxylamine experiments at 1 and 6 days. MS spectra related of retention time of standard **3a+3b**.

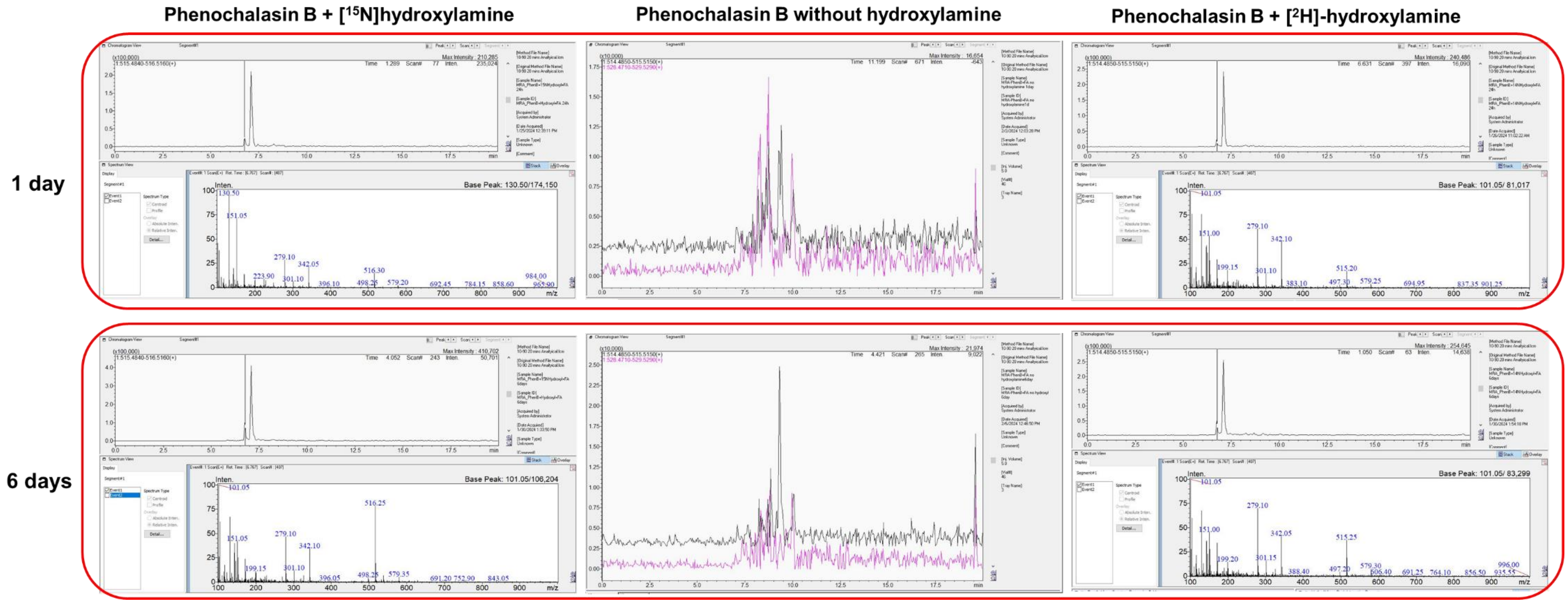

Figure S59. MS spectra of the investigation of phenochalasin B in the presence of [^{15}N]-hydroxylamine and [^{2}H]-hydroxylamine evaluated at 1 and 6 days. Comparison with the standard **2a+2b**.

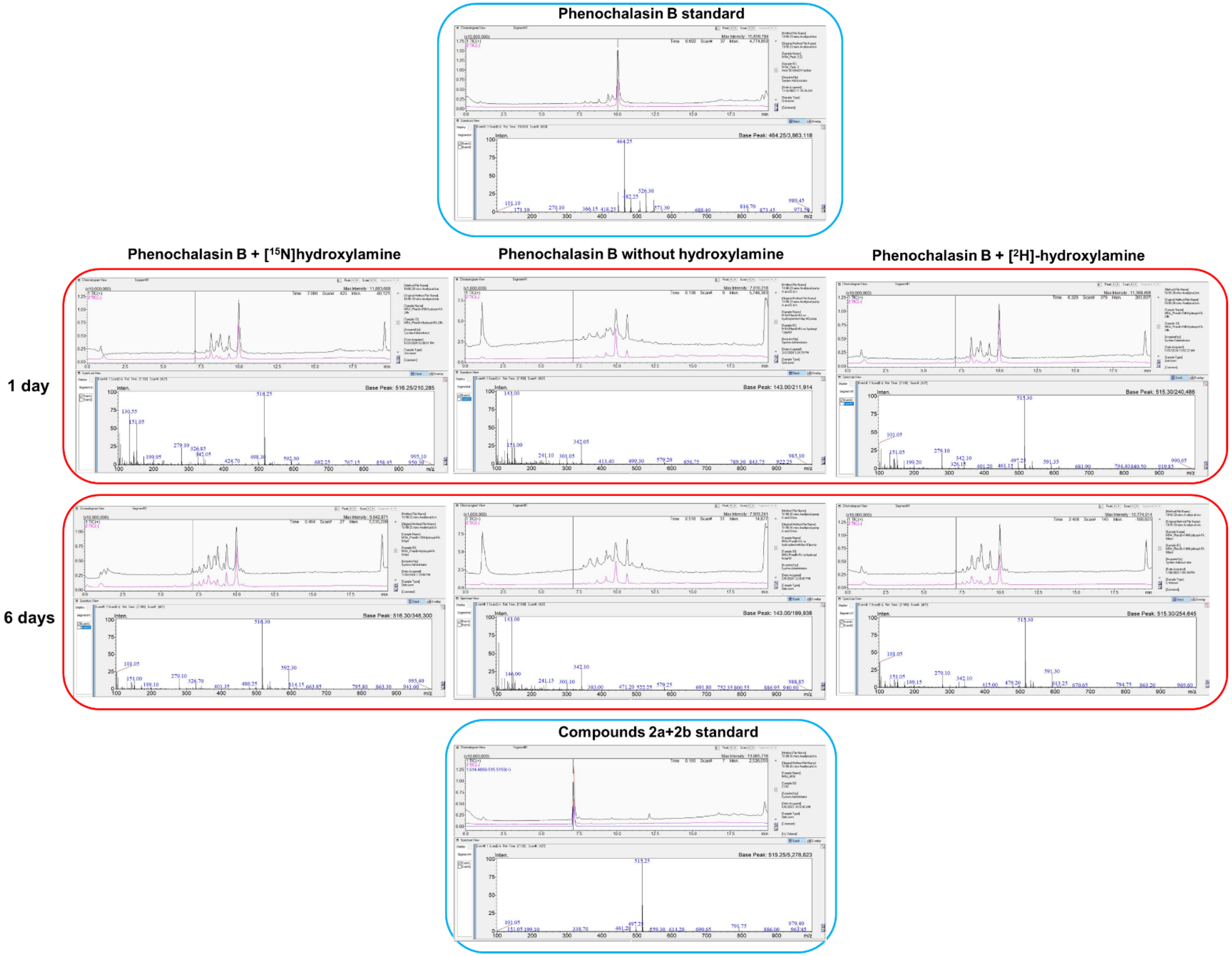

Figure S60. EIC of the investigation of phenochalasin B in the presence of [^{15}N]-hydroxylamine and [^{2}H]-hydroxylamine experiments at 1 and 6 days. MS spectra related of retention time of standard **2a+2b**.

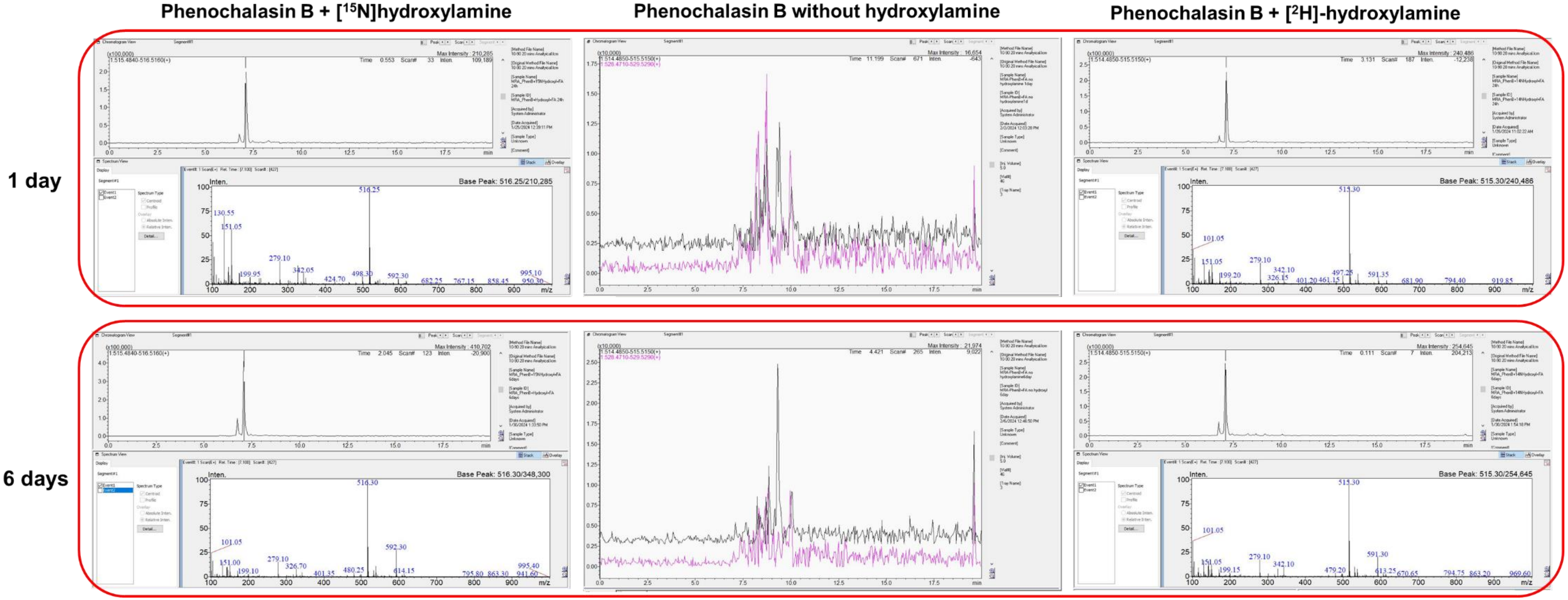

Figure S61. MS spectra of the investigation of phenochalasin B in the presence of [^{15}N]-hydroxylamine and [^{2}H]-hydroxylamine evaluated at 1 and 6 days. Comparison with the standard **1**.

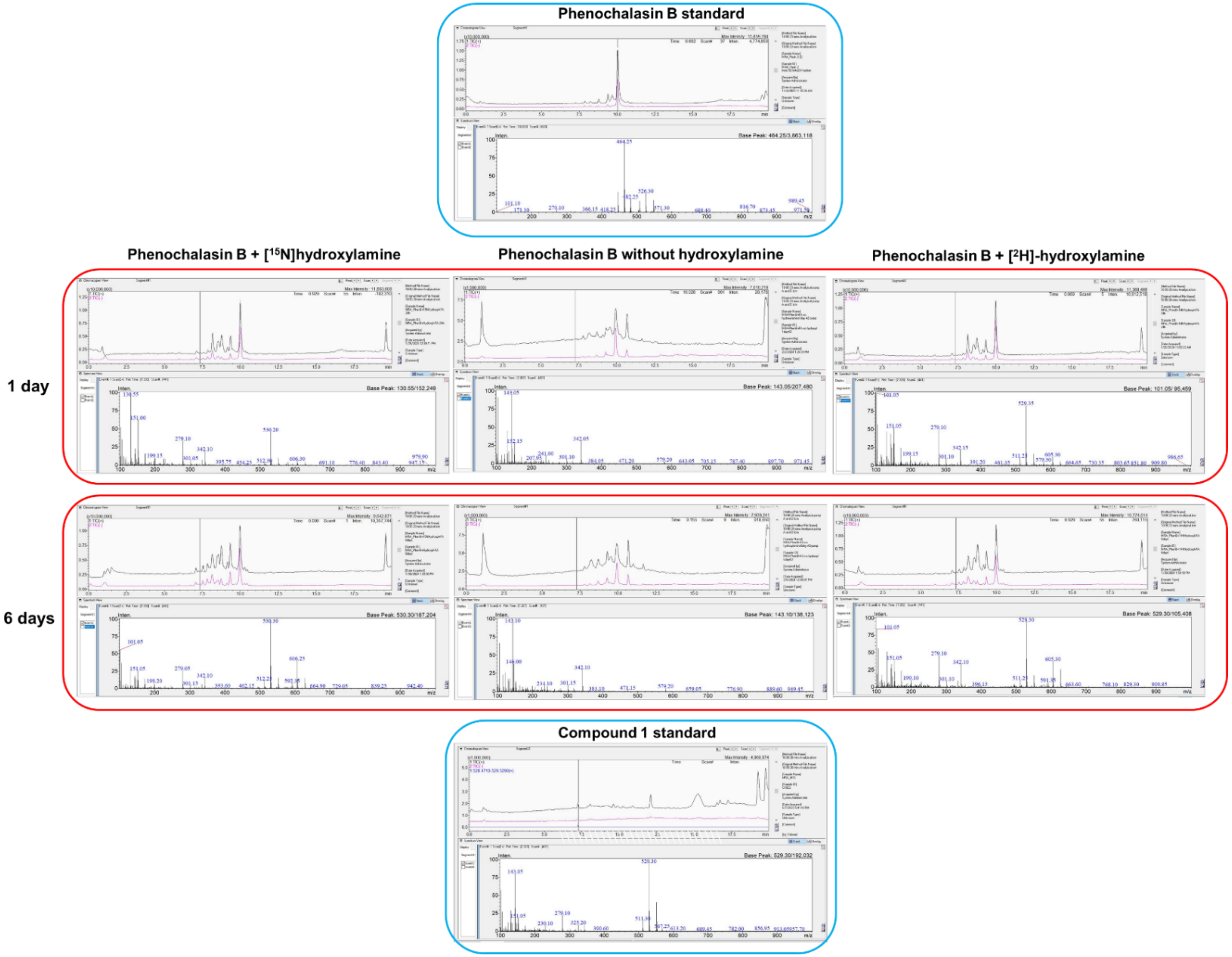

Figure S62. EIC of the investigation of phenochalasin B in the presence of [^{15}N]-hydroxylamine and [^{2}H]-hydroxylamine experiments at 1 and 6 days. MS spectra related of retention time of standard **1**.

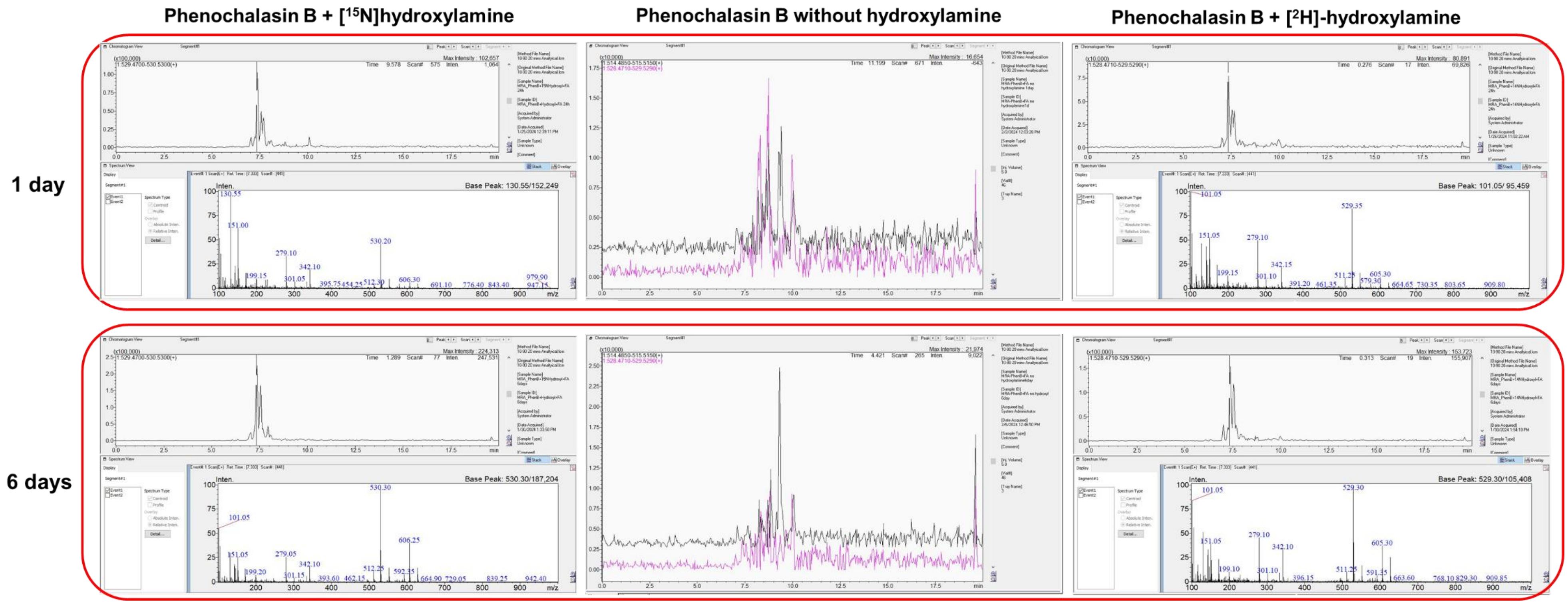

Table S4. ^1H (500 MHz) and ^{13}C (125 MHz) data of compound **7** in $CDCl_3$ and comparison with phenochalasin B NMR data.

	**7**		**Phenochalasin B[a]**	
**No.**	**δ_C, type**	**δ_H, mult. (*J* in Hz)**	**δ_C, type**	**δ_H, mult. (*J* in Hz)**
1	169.8, C	-	169.81, C	-
3	53.7, CH	3.66, ddd (7.6, 3.9, 2.5)	53.77, CH	3.65, ddd (8.0, 4.0, 3.0)
4	48.1, CH	2.99, dd (5.1, 2.8)	48.27, CH	2.99, dd (5.0, 3.0)
5	35.8, CH	2.29, dd (7.4, 5.1)	35.87, CH	2.30, dd (7.5, 5.0)
6	57.3, C	-	57.26, C	-
7	60.6, CH_2	2.63, s	60.61, CH_2	2.63, s
8	45.9, CH	2.64, overlapped	46.02, CH	2.64, d (10.0)
9	87.0, C	-	86.99, C	-
10	44.2, CH_2	2.59, dd (13.8, 7.8); 2.82, dd (13.8, 4.4)	44.31, CH_2	2.59, dd (14.0, 8.0); 2.82, dd (14.0, 4.0)
11	13.2, CH_3	1.12, d (7.3)	13.25, CH_3	1.12, d (7.5)
12	19.7, CH_3	1.24, s	19.69, CH_3	1.25, s
13	128.4, CH	5.89 ddd (14.9, 9.3, 1.5)	128.38, CH	5.89 ddd (15.0, 10.0, 1.5)
14	131.5, CH	5.23 ddd (14.9, 10.9, 3.8)	131.64, CH	5.23 ddd (150, 11.0, 4.0)
15	39.0, CH_2	2.14, m; 2.65, overlapped	39.07, CH_2	2.14, dddd (14.0, 4.0, 2.5, 1.5); 2.65, ddd (14.0, 11.5, 11.0)
16	40.8, CH	2.94, ddd (11.7, 6.8, 2.3)	40.85, CH	2.94, ddd (11.5, 7.0, 2.5)
17	211.7, C	-	211.72, C	-
18	76.7, C	-	76.74, C	-
19	120.4, CH	5.61, d (11.6)	120.42, CH	5.62, d (12.0)
20	142.11, CH	6.52, d (11.6)	142.17, CH	6.53, d (12.0)
22	149.4, C	-	149.36, C	-
24	20.1, CH_3	1.16, d (6.8)	20.08, CH_3	1.16, d (7.0)
25	24.3, CH_3	1.50, s	24.36, CH_3	1.50, s
1'	127.9, C	-	127.97, C	-
2', 6'	130.5, CH_2	7.06, dd (8.5, 2.0)	130.47, CH_2	7.05, dd (8.5, 2.0)
3', 5'	114.3, CH_2	6.86, dd (8.5, 2.0)	114.39, CH_2	6.86, dd (8.5, 2.0)
4'	158.8, C	-	158.92, C	-
7'	55.3, CH_3	3.79, s	55.29, CH_3	3.79, s
18-OH	-	3.49. s	-	4.43, s

[a]Data from Tomoda, H.; Namatame, I.; Tabata, N.; Kawaguchi, K.; Si, S.; Omur, S. *J. Antibiotics* **1999**, *52* (10), 857–861.

Figure S63. ^{1}H NMR spectrum (500 MHz, $CDCl_3$) of phenochalasin B (**7**).

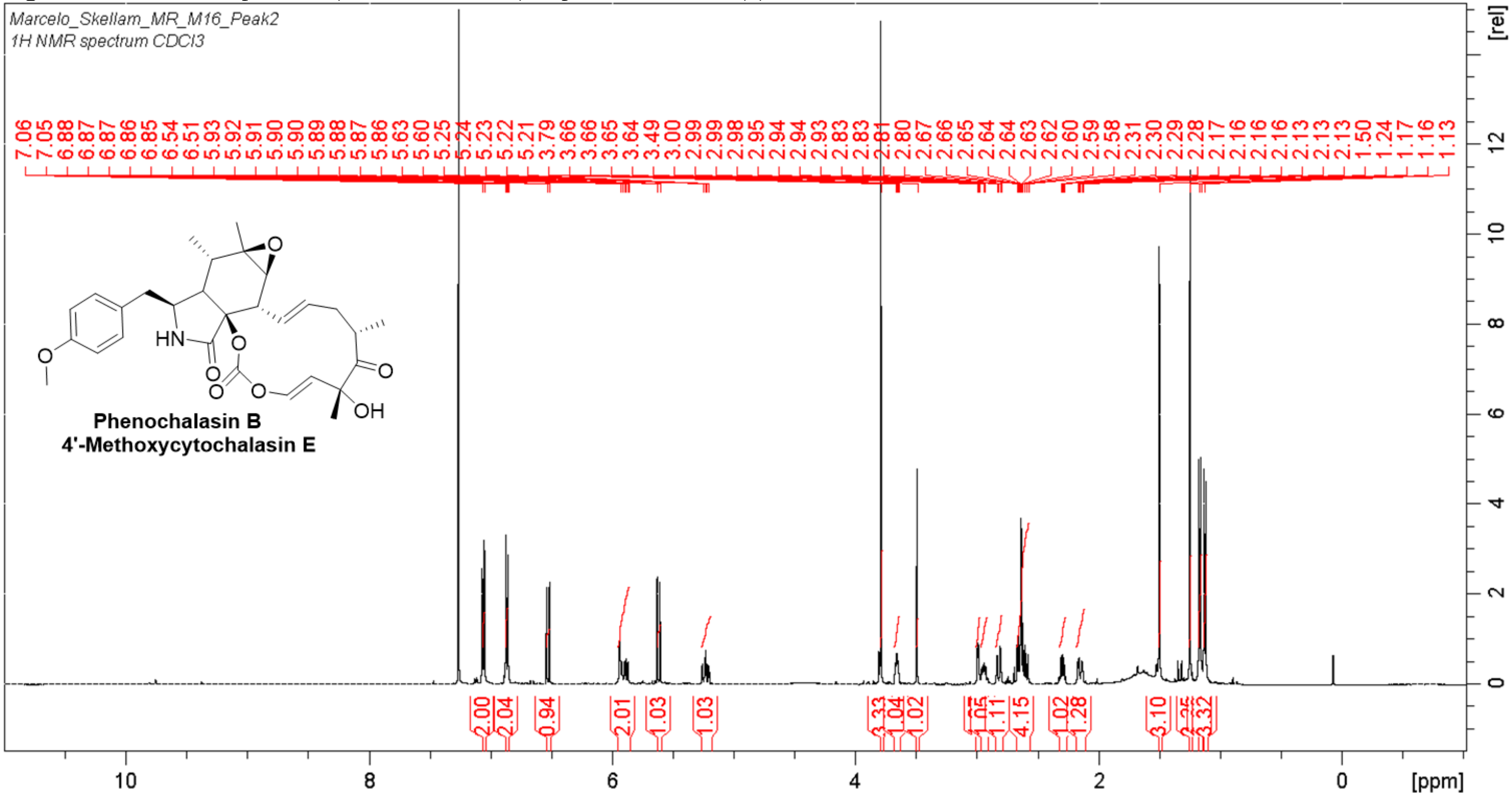

Figure S64. Expansion of ^{1}H NMR spectrum (500 MHz, $CDCl_3$) of phenochalasin B (**7**).

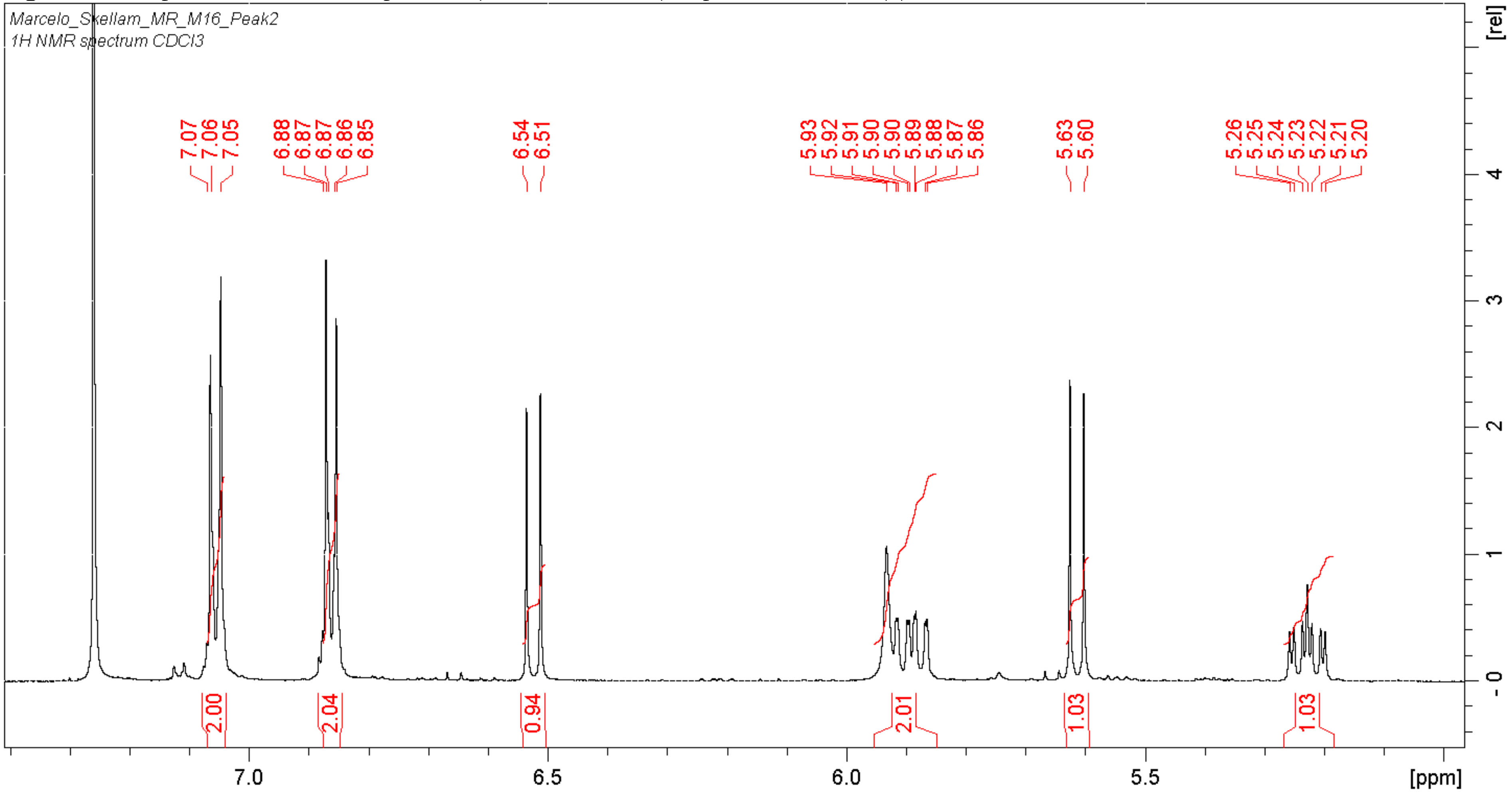

Figure S65. Expansion of ^{1}H NMR spectrum (500 MHz, $CDCl_3$) of phenochalasin B (**7**).

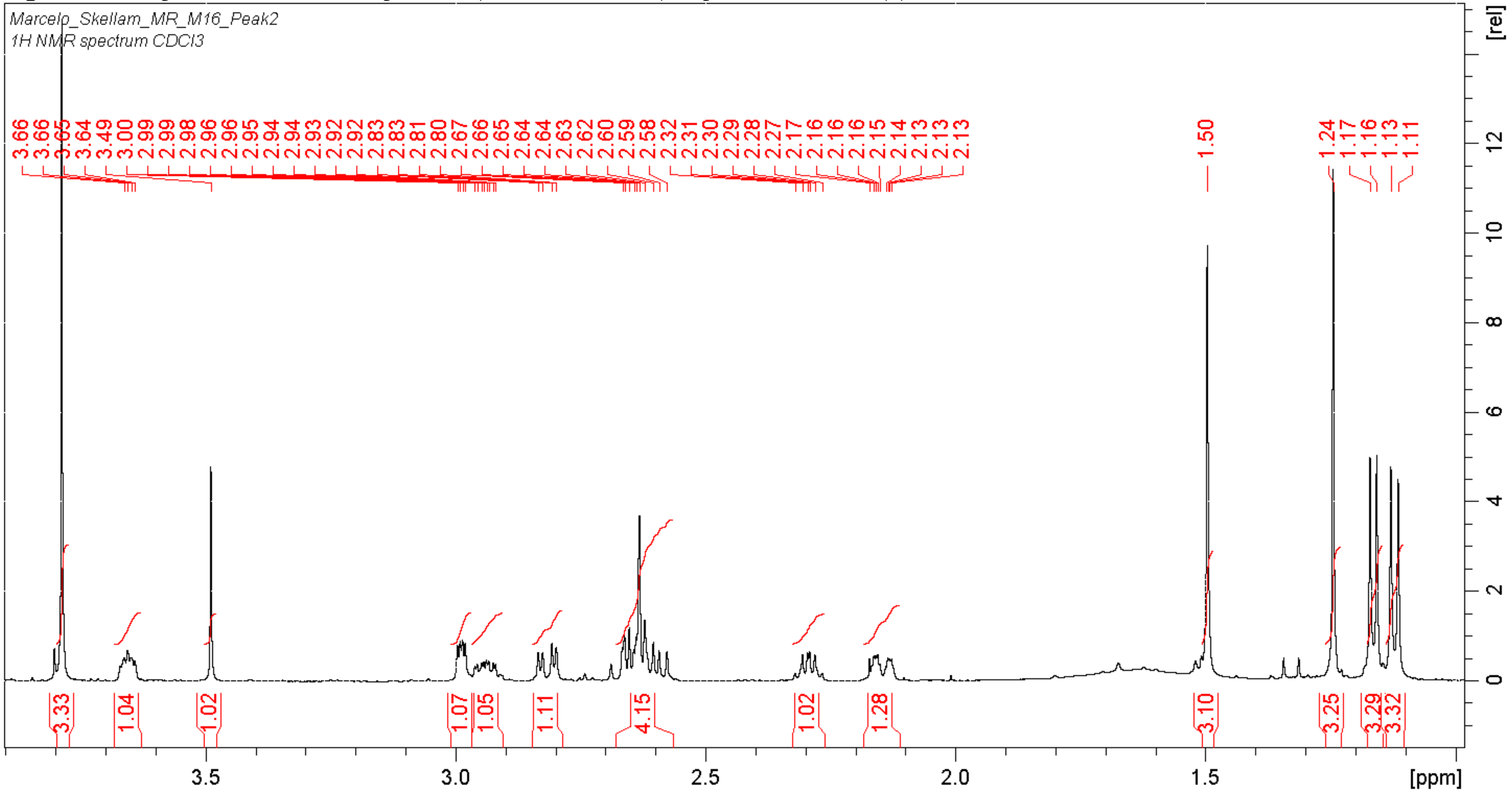

Figure S66. ^{13}C NMR spectrum (125 MHz, $CDCl_3$) of phenochalasin B (**7**).

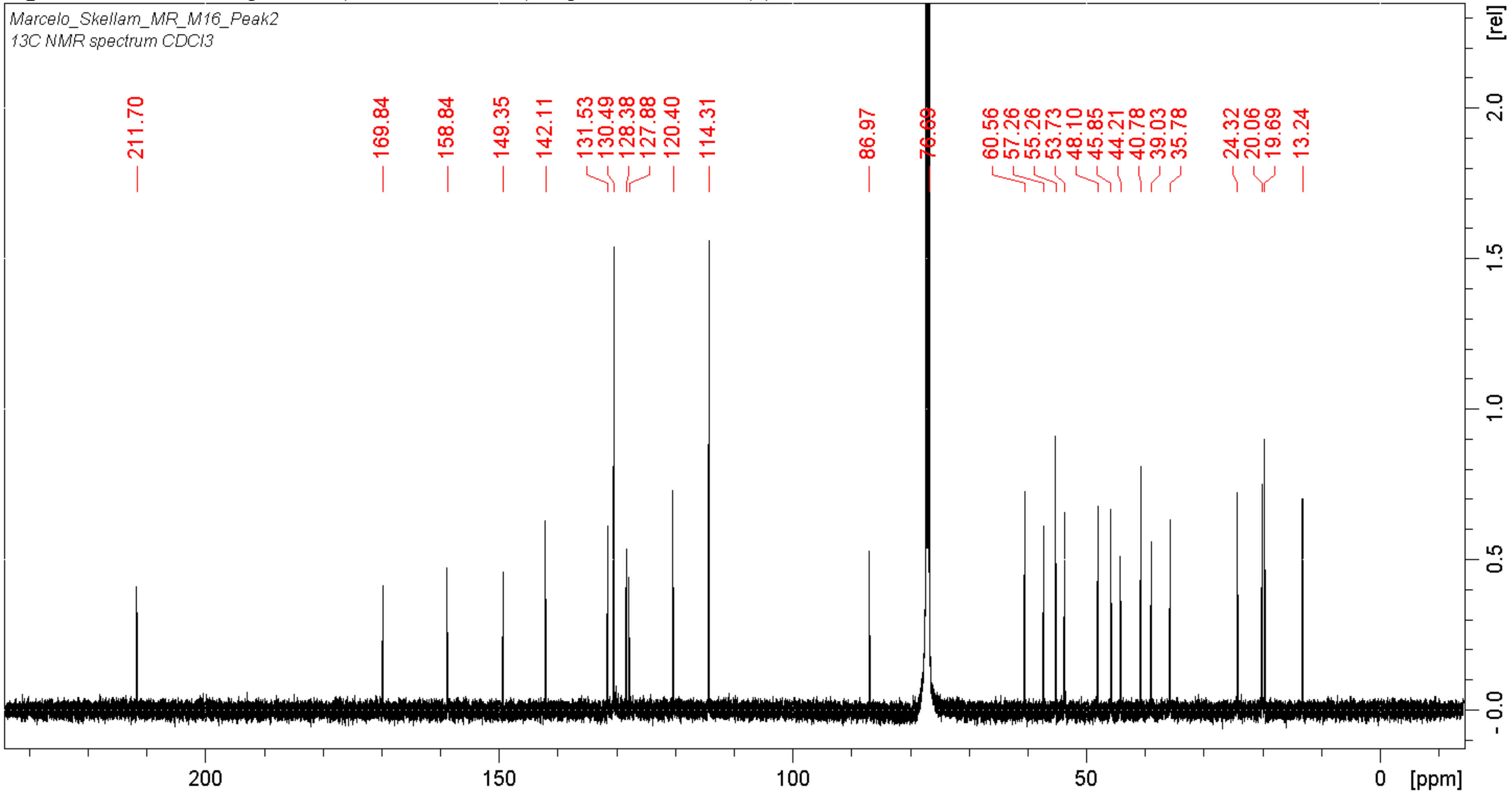

Scheme S1. Proposed mechanism for the non-enzymatic transformations resulting in the compounds **1**-**3**.

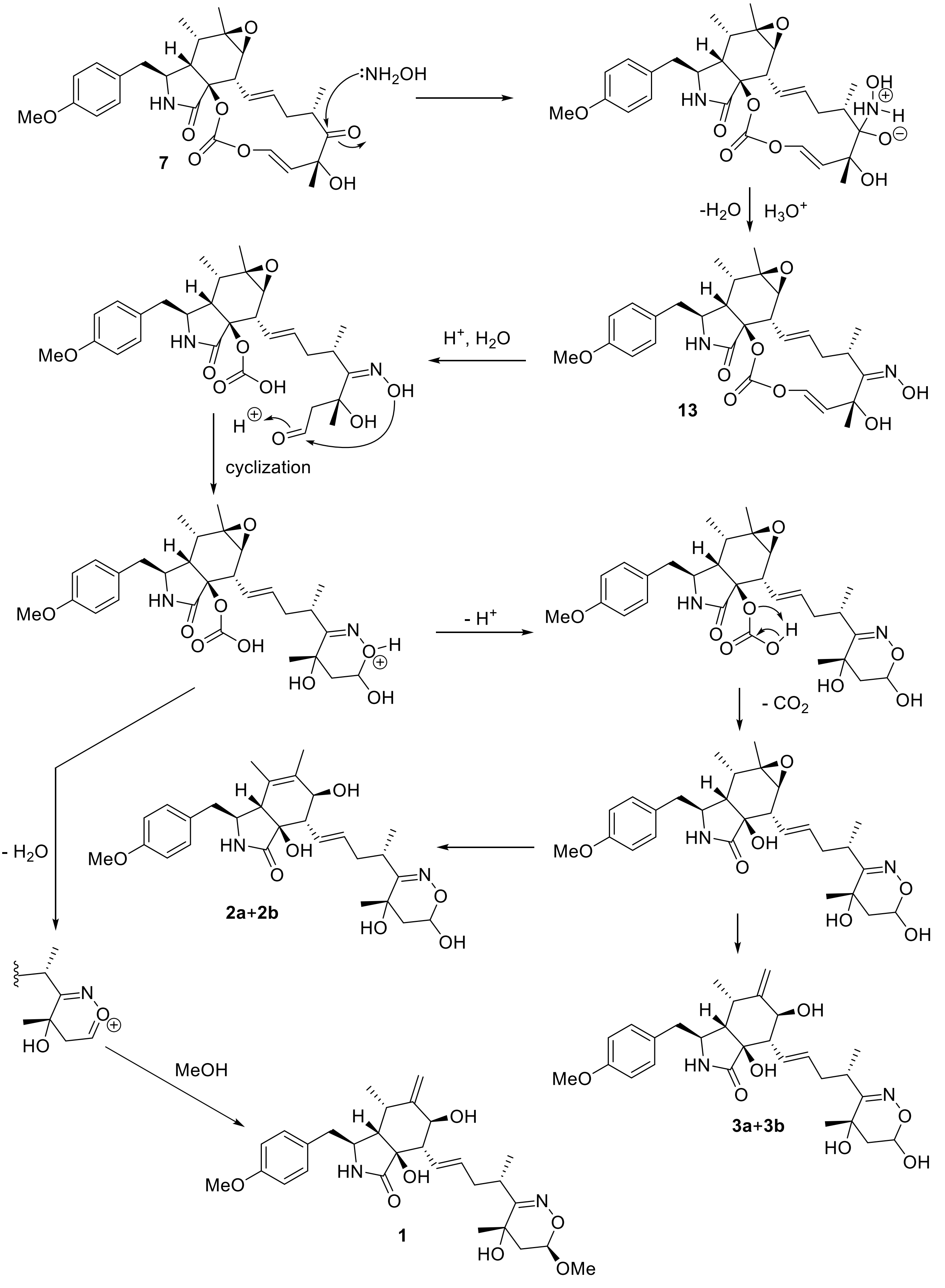

Table S5. Results of DP4+ analysis for the correlation between experimental and calculated NMR data of perochalasin A (**1**). Isomer 1: 3*S*,4*S*,5*S*,7*S*,8*S*,9*S*,13E16*S*,18*S*,20*R*; Isomer 2: 3*S*,4*S*,5*S*,7*S*,8*S*,9*S*,13*E*,16*R*,18*S*,20*R*; Isomer 3: 3*S*,4*S*,5*S*,7*S*,8*S*,9*S*,13*E*,16*S*,18*R*,20*S*; Isomer 4: 3*S*,4*S*,5*S*,7*S*,8*S*,9*S*,13*E*,16*R*,18*R*,20*S*.

	A	B	C	D	E	F	G	H
1	Functional		Solvent?		Basis Set		Type of Data	
2	mPW1PW91		PCM		6-31+G(d,p)		Unscaled Shifts	
3								
4			Isomer 1	Isomer 2	Isomer 3	Isomer 4	Isomer 5	Isomer 6
5	sDP4+ (H data)		99.97%	0.03%	0.00%	0.00%		
6	sDP4+ (C data)		98.78%	0.96%	0.00%	0.26%		
7	sDP4+ (all data)		100.00%	0.00%	0.00%	0.00%		
8	uDP4+ (H data)		99.87%	0.01%	0.10%	0.02%		
9	uDP4+ (C data)		99.84%	0.05%	0.00%	0.10%		
10	uDP4+ (all data)		100.00%	0.00%	0.00%	0.00%		
11	DP4+ (H data)		100.00%	0.00%	0.00%	0.00%		
12	DP4+ (C data)		100.00%	0.00%	0.00%	0.00%		
13	DP4+ (all data)		100.00%	0.00%	0.00%	0.00%		

Cont.

A	B	C	D	E	F	G	H
Functional		Solvent?		Basis Set		Type of Data	
mPW1PW91		PCM		6-31+G(d,p)		Unscaled Shifts	
		DP4+	100.00%	0.00%	0.00%	0.00%	
Nuclei	sp2?	Experimental	Isomer 1	Isomer 2	Isomer 3	Isomer 4	Isomer 5
C	x	174.8	174.506546	173.594138	173.776058	174.276485	
C		52.1	58.6387889	59.712587	56.9738968	57.6431861	
C		50.8	55.1040625	54.8128979	55.5414294	56.4791413	
C		30.1	37.8384377	42.051912	38.6803477	37.984422	
C	x	151.2	152.399687	153.408261	152.138088	151.979792	
C		71.4	72.616773	71.4791345	70.415582	70.7690555	
C		52.5	56.7981884	60.0946238	56.069552	57.3640136	
C		76.9	79.9769092	80.491281	81.2400608	82.3004935	
C		41.6	45.4352076	44.3272208	46.7863233	46.7455201	
C		14.4	16.5936116	18.6657062	18.530071	18.2116097	
C	x	110.7	110.646949	106.72569	111.755636	109.982234	
C	x	129.2	128.173844	132.848747	124.938169	129.326996	
C	x	131.3	136.433117	133.578855	141.375644	138.608456	
C		35.5	43.1659738	42.3513473	44.0716906	40.7193506	
C		30.5	39.2907328	40.8861381	48.2510382	43.1681638	
C	x	151.7	166.900821	167.267606	169.185115	165.415312	
C		77	70.6994021	73.1945325	67.4481776	70.9647301	
C		42.6	42.995549	43.4590125	39.2419029	41.4358391	
C		99.2	99.8660609	101.141218	100.019621	100.022555	
C		15	23.8396521	23.0383286	21.2570651	23.6563484	
C		27.4	26.6118058	26.9731098	30.5928194	26.9284585	
C		54.9	54.8430256	54.8226754	55.1546438	54.9050166	
C		59	56.6799921	57.5980695	55.5527011	57.3302546	
C	x	129.2	127.881168	130.432213	128.820607	127.704994	
C	x	130.8	128.78753	129.873994	128.350566	127.84237	
C	x	113.6	112.616489	112.854606	108.34096	112.534079	
C	x	157.8	156.140731	155.952788	156.347757	156.209562	
C	x	113.6	111.1217	109.944162	117.366602	111.065765	
C	x	130.8	128.462552	129.484188	125.751774	128.607988	
H		3.11	3.31941912	3.28388274	3.14007509	3.28208419	
H		2.06	2.26428362	2.23685112	2.22770857	2.12426971	
H		2.82	2.85107729	2.9043491	3.08387383	2.79436646	
H		3.69	4.07989524	4.21795993	4.11774676	4.02900209	
H		2.38	2.51118304	2.36486811	2.42173417	2.33746287	
H		2.7	2.80154763	3.35383454	2.43946083	2.29744561	
H		2.65	2.84554244	2.8052079	2.97941398	3.04213838	
H		0.78	1.04076845	1.17890288	1.32395577	1.30603674	
H	x	4.84	5.45116388	5.43372387	5.62056792	5.81132351	
H	x	5.05	5.4938869	5.61172498	5.91929895	5.9857867	
H	x	5.53	6.42952983	6.33198812	5.74722854	5.79860759	
H	x	5.35	6.25082913	6.23876237	6.31097851	6.27947655	
H		2.49	2.47718214	2.6350263	3.03240661	2.23429832	
H		2.22	2.35374205	2.58155654	2.2674167	2.28654908	
H		2.81	2.8721978	3.05819014	2.82253516	2.57006052	
H		1.89	1.93067011	1.84118793	1.83251226	1.60078516	
H		2.35	1.75576185	2.13385301	2.56077381	1.94090045	
H		4.95	5.00524346	4.63830375	4.9556081	4.75317592	
H		1.19	1.23780076	1.23015749	1.26976334	1.18591306	
H		1.37	1.24727038	1.45040063	1.48617565	1.2761988	
H		3.71	3.89868681	3.8761273	3.95298074	3.89038906	
H		3.6	3.68769813	3.70690207	3.50716359	3.69316027	
H	x	7.1	7.67040654	7.54485977	7.547647	7.68968458	
H	x	6.85	7.18176981	7.2961926	7.09477853	7.21122861	
H	x	6.85	7.17570027	7.14033556	7.10813172	7.15219635	
H	x	7.1	7.60989234	7.78198664	7.66867433	7.53713049	

Table S6. Atomic coordinates of the lowest energy conformer of (3*S*,4*S*,5*S*,7*S*,8*S*,9*S*,13*E*,16*S*,18*S*,20*R*)-perochalasin A (**1**).

Conf 1

E_{ZPC}=-1764.463047 Hartree

B3LYP/6-31G(d)

Symbol	X	Y	Z
C	-5.45744	-2.31669	0.223644
C	-4.42873	-1.5668	0.804328
C	-4.5082	-0.17641	0.927348
C	-5.66883	0.453974	0.445317
C	-6.70068	-0.27187	-0.13325
C	-6.60215	-1.66724	-0.24946
O	-7.66949	-2.28986	-0.82867
C	-7.63175	-3.70197	-0.9614
C	-3.37246	0.627782	1.521859
C	-2.43661	1.254114	0.4627
N	-1.81188	0.239819	-0.37673
C	-1.22528	2.014525	1.08646
C	0.059591	1.277428	0.596129
C	-0.4646	0.148223	-0.31745
O	0.224996	-0.7092	-0.86509
C	-1.22549	3.534977	0.774639
C	-0.94304	3.739975	-0.6988
C	0.341567	3.091545	-1.20885
C	1.050104	2.238121	-0.12245
C	-2.47246	4.247184	1.308243
C	-1.72446	4.418094	-1.5425
O	1.2038	4.13709	-1.65717
O	0.665452	0.686005	1.737111
C	2.261423	1.568993	-0.72499
C	3.527826	1.910308	-0.44267
C	4.756233	1.309438	-1.07801
C	5.475412	0.257884	-0.18526
C	6.891897	-0.03499	-0.71345
C	4.649883	-1.03057	-0.07638
N	4.453592	-1.62203	-1.20086
C	4.165615	-1.51758	1.292448
C	3.458061	-2.87018	1.152822
C	2.713286	-2.90359	-0.16078
O	3.680509	-2.77269	-1.24174
C	5.318116	-1.62173	2.303595
O	3.169181	-0.6049	1.813337
O	2.074695	-4.11767	-0.32064
C	1.172348	-4.15664	-1.42931
H	-5.35622	-3.39384	0.155418
H	-3.55015	-2.08688	1.179951

H	-5.76792	1.534041	0.53316
H	-7.59887	0.216255	-0.4987
H	-8.57061	-3.98251	-1.44217
H	-6.79185	-4.0266	-1.5899
H	-7.56284	-4.1979	0.015924
H	-2.76502	-0.00332	2.181946
H	-3.7706	1.445581	2.136491
H	-3.02456	1.923988	-0.17666
H	-2.35533	-0.46437	-0.86124
H	-1.24998	1.889057	2.173428
H	-0.37094	3.953787	1.327298
H	0.091664	2.431981	-2.05714
H	1.399455	2.931668	0.653006
H	-3.3916	3.909711	0.816099
H	-2.5823	4.068277	2.383903
H	-2.39543	5.328546	1.1544
H	-2.63308	4.919381	-1.22329
H	-1.45858	4.518979	-2.5913
H	2.056809	3.71614	-1.86485
H	1.352261	0.047882	1.454802
H	2.070228	0.799897	-1.47153
H	3.706291	2.674652	0.319244
H	4.502734	0.828028	-2.0277
H	5.472691	2.113048	-1.29698
H	5.576523	0.690136	0.819904
H	7.414134	-0.75217	-0.07078
H	6.837514	-0.46342	-1.71867
H	7.489295	0.883251	-0.75409
H	2.773096	-3.00441	1.995645
H	4.184854	-3.68937	1.158723
H	2.018074	-2.05853	-0.25854
H	4.926194	-2.00951	3.249456
H	6.099591	-2.29998	1.944029
H	5.773683	-0.64719	2.505542
H	3.478748	0.306977	1.679914
H	0.606382	-5.08622	-1.33019
H	0.489868	-3.29774	-1.40522
H	1.715415	-4.15984	-2.38073

Conf 2

E_{ZPC}=-1764.462492 Hartree

B3LYP/6-31G(d)

Symbol	X	Y	Z
C	7.197451	-1.34749	-0.24633
C	5.962985	-0.97755	-0.79079
C	5.553805	0.35823	-0.85032

C	6.433493	1.33078	-0.34404
C	7.663411	0.984474	0.198787
C	8.05501	-0.36203	0.253549
O	9.281249	-0.60032	0.803938
C	9.73324	-1.9431	0.879249
C	4.196086	0.743614	-1.39449
C	3.116244	0.887178	-0.29371
N	2.908241	-0.35495	0.437614
C	1.703833	1.204748	-0.87327
C	0.931455	-0.14138	-0.80636
C	1.745682	-1.00895	0.185944
O	1.420886	-2.10556	0.623318
C	0.939036	2.357119	-0.1463
C	0.372969	1.816416	1.148423
C	-0.68603	0.740514	0.950439
C	-0.55426	0.01981	-0.40824
C	1.740238	3.65761	-0.03326
C	0.733445	2.19552	2.378453
O	-2.00251	1.347659	0.940815
O	1.035784	-0.75147	-2.09388
C	-1.33041	-1.27552	-0.43394
C	-2.40643	-1.46278	-1.20636
C	-3.2872	-2.68595	-1.19231
C	-4.39374	-2.63948	-0.09904
C	-5.37782	-3.80757	-0.29134
C	-5.11593	-1.29289	-0.05149
N	-5.50162	-0.83912	-1.19021
C	-5.28712	-0.60925	1.313912
C	-6.04106	0.726253	1.144955
C	-5.70645	1.334036	-0.19626
O	-6.11597	0.424108	-1.23728
C	-6.05032	-1.51141	2.293534
O	-4.00593	-0.41498	1.916366
O	-6.40261	2.518647	-0.37096
C	-6.06863	3.22569	-1.56212
H	7.475871	-2.39497	-0.22566
H	5.311091	-1.75362	-1.18552
H	6.150912	2.38097	-0.38406
H	8.343958	1.7381	0.582946
H	10.71936	-1.90123	1.345289
H	9.066774	-2.55992	1.496704
H	9.822214	-2.39594	-0.11714
H	3.842942	-0.00071	-2.11769
H	4.262961	1.703202	-1.92427
H	3.441174	1.660059	0.411322
H	3.654961	-0.81251	0.945637
H	1.7995	1.480817	-1.92815
H	0.076053	2.582201	-0.78956
H	-0.63281	0.012129	1.770262

H	-0.9949	0.685961	-1.16094
H	2.605976	3.569816	0.630905
H	2.10712	3.967713	-1.01843
H	1.107634	4.461727	0.357646
H	1.445937	2.994723	2.559818
H	0.323703	1.709516	3.261947
H	-2.0801	1.865987	1.759164
H	0.511651	-1.57168	-2.05364
H	-0.98386	-2.06009	0.236437
H	-2.72948	-0.6523	-1.85907
H	-3.7659	-2.79946	-2.17135
H	-2.6868	-3.58723	-1.01408
H	-3.88941	-2.74797	0.867212
H	-6.11904	-3.85201	0.513382
H	-5.91465	-3.69753	-1.23957
H	-4.8413	-4.76396	-0.30705
H	-5.76886	1.396703	1.966186
H	-7.12546	0.570791	1.168352
H	-4.61738	1.491483	-0.31276
H	-6.1571	-0.98233	3.24663
H	-7.04728	-1.75637	1.914312
H	-5.50099	-2.43677	2.484654
H	-3.42011	0.0975	1.321621
H	-6.59539	4.181331	-1.51077
H	-4.98574	3.411592	-1.6191
H	-6.38477	2.678155	-2.45581

Conf 3

E_{ZPC}= -1764.462357 Hartree

B3LYP/6-31G(d)

Symbol	X	Y	Z
C	6.364496	-1.80562	0.731085
C	5.065192	-1.865	1.217959
C	3.956728	-1.68415	0.373671
C	4.205149	-1.44491	-0.98124
C	5.505675	-1.38321	-1.49132
C	6.59435	-1.56324	-0.6315
O	7.905213	-1.52788	-1.01395
C	8.197407	-1.30794	-2.38429
C	2.543881	-1.72557	0.912529
C	2.113925	-0.40826	1.605322
N	0.75379	-0.49587	2.146334
C	1.984512	0.839101	0.690052
C	0.514172	0.780899	0.201075
C	-0.20113	0.117959	1.4063
O	-1.4141	0.06041	1.624372

C	2.383159	2.162776	1.426823
C	1.861701	3.345138	0.636269
C	0.356611	3.349476	0.456437
C	-0.05921	2.112204	-0.36464
C	1.967201	2.286297	2.9132
C	2.653906	4.2748	0.097852
O	-0.13144	4.488782	-0.23842
O	0.481499	-0.2025	-0.84439
C	-1.54924	2.082335	-0.60942
C	-2.09607	1.841906	-1.80384
C	-3.56681	1.809492	-2.11487
C	-4.0962	0.39474	-2.4901
C	-5.37513	0.493268	-3.34303
C	-4.34086	-0.47181	-1.24915
N	-5.28731	-0.03946	-0.49358
C	-3.50028	-1.7297	-0.99968
C	-4.11626	-2.53405	0.158495
C	-4.60991	-1.59211	1.231773
O	-5.62212	-0.72485	0.674884
C	-3.38508	-2.62044	-2.24007
O	-2.15445	-1.3426	-0.67134
O	-5.19665	-2.31376	2.258597
C	-5.56913	-1.52799	3.388328
H	7.220061	-1.9523	1.383179
H	4.907281	-2.06782	2.275694
H	3.364656	-1.30659	-1.65717
H	5.652893	-1.20094	-2.54979
H	9.285918	-1.32351	-2.46483
H	7.775739	-2.09841	-3.0194
H	7.822968	-0.33406	-2.72703
H	2.456309	-2.53159	1.655181
H	1.832495	-1.93745	0.111607
H	2.817703	-0.21096	2.421403
H	0.481968	-1.16096	2.86039
H	2.634744	0.745697	-0.18316
H	3.479858	2.1968	1.407462
H	-0.12985	3.289276	1.444753
H	0.444972	2.214978	-1.33404
H	0.886181	2.232673	3.067071
H	2.424767	1.508657	3.532036
H	2.311302	3.253282	3.295908
H	3.734044	4.238265	0.215643
H	2.248985	5.089254	-0.49498
H	0.074677	5.265665	0.305329
H	-0.45181	-0.42184	-1.05098
H	-2.19054	2.273444	0.249363
H	-1.43262	1.649036	-2.65024
H	-4.15514	2.195462	-1.27591
H	-3.75415	2.472689	-2.97183

H	-3.32257	-0.09303	-3.09253
H	-5.74218	-0.49966	-3.62682
H	-6.16956	0.994823	-2.78256
H	-5.18209	1.059373	-4.26177
H	-3.36506	-3.22122	0.559865
H	-4.97517	-3.11892	-0.19003
H	-3.80323	-0.94555	1.618015
H	-2.8448	-3.53452	-1.97244
H	-4.37414	-2.89769	-2.61845
H	-2.82677	-2.12406	-3.03747
H	-2.16746	-0.78821	0.138057
H	-5.90084	-2.23092	4.155951
H	-4.71158	-0.95346	3.767837
H	-6.38355	-0.83773	3.145263

Conf 4

E_{ZPC}= -1764.462239 Hartree

B3LYP/6-31G(d)

Symbol	X	Y	Z
C	4.963009	2.446838	0.444374
C	4.076401	1.502658	0.973436
C	4.340058	0.130768	0.917609
C	5.53929	-0.2769	0.307345
C	6.432405	0.64398	-0.22271
C	6.149728	2.017393	-0.15857
O	7.090665	2.841639	-0.70363
C	6.864666	4.2418	-0.65687
C	3.354549	-0.88287	1.456938
C	2.444334	-1.49732	0.368015
N	1.657673	-0.47765	-0.31553
C	1.367227	-2.46526	0.943411
C	-9.5E-05	-1.7273	0.782632
C	0.330662	-0.49682	-0.09401
O	-0.46611	0.356339	-0.49848
C	1.384302	-3.87355	0.278972
C	0.822588	-3.74816	-1.11982
C	-0.62479	-3.27021	-1.1776
C	-1.08651	-2.65227	0.158596
C	2.739402	-4.57847	0.394256
C	1.490902	-4.00804	-2.24548
O	-1.42874	-4.41279	-1.49488
O	-0.37954	-1.2718	2.078717
C	-2.46644	-2.03141	0.118059
C	-3.22524	-1.77574	-0.95398
C	-4.63209	-1.21333	-0.93438
C	-4.98632	-0.26279	0.232482

C	-6.51241	-0.05905	0.31092
C	-4.27285	1.089269	0.107489
N	-4.46149	1.678998	-1.01888
C	-3.44664	1.632732	1.281094
C	-3.02939	3.084775	0.986372
C	-2.69376	3.229357	-0.47933
O	-3.86616	2.91693	-1.26529
C	-4.20717	1.572711	2.609693
O	-2.28336	0.810084	1.459221
O	-2.3424	4.540109	-0.75724
C	-1.87496	4.752654	-2.08688
H	4.721141	3.501119	0.515999
H	3.163842	1.85239	1.451222
H	5.780692	-1.33671	0.255279
H	7.361236	0.32728	-0.68694
H	7.730339	4.700791	-1.13765
H	5.955051	4.522744	-1.20418
H	6.78927	4.604273	0.376878
H	2.710365	-0.42465	2.216887
H	3.889694	-1.7085	1.944102
H	3.07056	-2.00322	-0.37469
H	2.090057	0.313206	-0.77815
H	1.53199	-2.59114	2.017973
H	0.674701	-4.48538	0.854052
H	-0.71414	-2.51859	-1.97714
H	-1.16397	-3.48531	0.872448
H	3.5357	-4.06897	-0.15901
H	3.049533	-4.64008	1.443872
H	2.671044	-5.59882	0.003032
H	2.506408	-4.39305	-2.2479
H	1.025713	-3.86718	-3.21781
H	-2.35318	-4.14416	-1.35775
H	-1.12935	-0.64574	2.004944
H	-2.86487	-1.80487	1.103682
H	-2.838	-1.99293	-1.94986
H	-4.81497	-0.67737	-1.87168
H	-5.3452	-2.05261	-0.92421
H	-4.65991	-0.72472	1.168927
H	-6.78526	0.603103	1.139909
H	-6.8822	0.393836	-0.61456
H	-7.02549	-1.01638	0.460737
H	-2.16997	3.344991	1.611497
H	-3.84814	3.778821	1.208144
H	-1.8984	2.530705	-0.79163
H	-3.5731	1.991382	3.398304
H	-5.13313	2.154065	2.561742
H	-4.44879	0.543168	2.886204
H	-1.78364	0.743431	0.611072
H	-1.5248	5.786657	-2.12816

H	-1.04028	4.076652	-2.32341
H	-2.6737	4.605459	-2.82115

Conf 5

E_{ZPC}= -1764.462076 Hartree

B3LYP/6-31G(d)

Symbol	X	Y	Z
C	7.125799	-1.63589	-0.22294
C	5.910032	-1.25372	-0.77765
C	5.548819	0.099937	-0.8895
C	6.461048	1.053069	-0.42568
C	7.688925	0.689771	0.133718
C	8.025839	-0.66485	0.238729
O	9.193866	-1.13862	0.763158
C	10.14726	-0.2006	1.2354
C	4.204199	0.510155	-1.44831
C	3.124591	0.715183	-0.35752
N	2.876889	-0.49737	0.409757
C	1.724421	1.057426	-0.95354
C	0.905999	-0.25793	-0.83891
C	1.691348	-1.11819	0.182492
O	1.329067	-2.18634	0.658759
C	0.999165	2.261835	-0.27231
C	0.418185	1.789629	1.042323
C	-0.67606	0.742411	0.886855
C	-0.57291	-0.0315	-0.44495
C	1.844003	3.537783	-0.20828
C	0.793922	2.203017	2.256728
O	-1.97216	1.391662	0.859668
O	0.985387	-0.91587	-2.10431
C	-1.39178	-1.30014	-0.42221
C	-2.47383	-1.48214	-1.1875
C	-3.39089	-2.67779	-1.12703
C	-4.46793	-2.57457	-0.00977
C	-5.48392	-3.72358	-0.14372
C	-5.15795	-1.21064	0.015701
N	-5.55689	-0.77867	-1.12692
C	-5.28591	-0.48706	1.3651
C	-6.02237	0.855043	1.175907
C	-5.70306	1.423557	-0.18619
O	-6.14633	0.495269	-1.19653
C	-6.03951	-1.34855	2.387611
O	-3.98776	-0.29645	1.931498
O	-6.38315	2.614935	-0.3776
C	-6.06055	3.286893	-1.59198
H	7.404828	-2.68197	-0.14302

H	5.229241	-2.01989	-1.14142
H	6.21738	2.110739	-0.50663
H	8.368287	1.462871	0.474508
H	10.99042	-0.78919	1.601504
H	10.49158	0.464968	0.43264
H	9.745287	0.405893	2.058076
H	3.832096	-0.24274	-2.15306
H	4.300237	1.452865	-2.00312
H	3.468338	1.49909	0.326309
H	3.605271	-0.95978	0.939571
H	1.831412	1.290088	-2.01776
H	0.143505	2.491276	-0.92369
H	-0.64345	0.042928	1.732494
H	-0.99215	0.621002	-1.2214
H	2.707679	3.444444	0.457808
H	2.218852	3.799021	-1.20457
H	1.240024	4.376685	0.153761
H	1.532662	2.984711	2.40684
H	0.370642	1.764453	3.158476
H	-2.02719	1.943721	1.657538
H	0.43355	-1.71564	-2.03368
H	-1.06963	-2.07002	0.276707
H	-2.77266	-0.68663	-1.86958
H	-3.8957	-2.79754	-2.09191
H	-2.81333	-3.59287	-0.94323
H	-3.9422	-2.66797	0.946594
H	-6.20605	-3.72625	0.679391
H	-6.04115	-3.62818	-1.08174
H	-4.9707	-4.69278	-0.14376
H	-5.72496	1.54183	1.974607
H	-7.10841	0.717214	1.222883
H	-4.614	1.560083	-0.3265
H	-6.11691	-0.79075	3.327079
H	-7.04824	-1.58935	2.038054
H	-5.49931	-2.27614	2.593259
H	-3.40856	0.189549	1.308637
H	-6.57014	4.252438	-1.55422
H	-4.976	3.452668	-1.67413
H	-6.40313	2.723393	-2.4658

Conf 6

E_{ZPC}= -1764.461973 Hartree

B3LYP/6-31G(d)

Symbol	X	Y	Z
C	-4.86999	-2.67256	0.4978
C	-4.01729	-1.71739	1.038728

C	-4.32133	-0.34651	0.980916
C	-5.5187	0.025062	0.360744
C	-6.38977	-0.91931	-0.18803
C	-6.06476	-2.27968	-0.12204
O	-6.83566	-3.28965	-0.61952
C	-8.06176	-2.95216	-1.2487
C	-3.36759	0.691825	1.531303
C	-2.43943	1.30541	0.457568
N	-1.61526	0.292103	-0.18946
C	-1.39794	2.308811	1.038835
C	0.005844	1.655824	0.82908
C	-0.28741	0.374025	0.015054
O	0.536486	-0.46432	-0.36412
C	-1.51377	3.730075	0.418333
C	-1.03595	3.66704	-1.01643
C	0.409582	3.208831	-1.18877
C	0.994274	2.627101	0.114136
C	-2.8919	4.364148	0.631403
C	-1.77565	3.967501	-2.08623
O	1.163532	4.355483	-1.59838
O	0.489716	1.283616	2.115832
C	2.397654	2.075805	-0.01933
C	3.075536	1.804603	-1.14072
C	4.507068	1.314695	-1.21852
C	5.020022	0.467317	-0.03101
C	6.558379	0.371267	-0.06864
C	4.398339	-0.93471	-0.01553
N	4.543195	-1.5828	-1.11596
C	3.705346	-1.45339	1.251492
C	3.362165	-2.94393	1.076639
C	2.931644	-3.20435	-0.34768
O	4.020527	-2.87075	-1.23896
C	4.56507	-1.2648	2.505517
O	2.509337	-0.69281	1.478869
O	2.650392	-4.54959	-0.51815
C	2.099184	-4.87752	-1.79099
H	-4.63596	-3.73133	0.549905
H	-3.10159	-2.0421	1.527961
H	-5.78969	1.077701	0.307178
H	-7.31035	-0.58652	-0.65402
H	-8.50517	-3.89759	-1.56614
H	-8.746	-2.44606	-0.55482
H	-7.90271	-2.31358	-2.1278
H	-2.73933	0.254949	2.316906
H	-3.92933	1.515454	1.990564
H	-3.05702	1.787843	-0.30832
H	-2.01741	-0.52848	-0.62712
H	-1.54223	2.392327	2.120489
H	-0.80033	4.359893	0.96905

H	0.442353	2.442096	-1.97901
H	1.080349	3.47066	0.813931
H	-3.69321	3.824256	0.114897
H	-3.13928	4.389036	1.698868
H	-2.89932	5.394331	0.260498
H	-2.79295	4.3394	-2.00811
H	-1.36942	3.878001	-3.09045
H	2.100679	4.103368	-1.53553
H	1.278193	0.710278	2.022984
H	2.888514	1.916149	0.937132
H	2.594354	1.953453	-2.10804
H	4.632856	0.723806	-2.13192
H	5.169563	2.18709	-1.33169
H	4.737586	0.966304	0.900762
H	6.942814	-0.21861	0.770722
H	6.885846	-0.11199	-0.99472
H	7.012064	1.368074	-0.01557
H	2.568804	-3.21356	1.78018
H	4.237313	-3.57256	1.277429
H	2.070826	-2.57837	-0.64027
H	4.027281	-1.6746	3.36699
H	5.521804	-1.78771	2.410871
H	4.756903	-0.20709	2.703716
H	1.940664	-0.71022	0.672791
H	1.82042	-5.93268	-1.74277
H	1.203274	-4.27454	-1.99928
H	2.82746	-4.72595	-2.59445

Conf 7

E_{ZPC}= -1764.461957 Hartree

B3LYP/6-31G(d)

Symbol	X	Y	Z
C	-6.70939	-1.33093	-0.46777
C	-5.40499	-1.49938	-0.93891
C	-4.31294	-1.59871	-0.07114
C	-4.57047	-1.53737	1.307991
C	-5.86053	-1.37012	1.797435
C	-6.94169	-1.26195	0.911141
O	-8.16851	-1.10193	1.487389
C	-9.3004	-0.99147	0.639476
C	-2.9055	-1.74955	-0.60382
C	-2.26904	-0.4294	-1.10953
N	-0.97601	-0.70557	-1.7348
C	-1.91329	0.58393	0.022119
C	-0.35374	0.571658	0.13353
C	0.110225	-0.3069	-1.04615

O	1.276355	-0.61324	-1.31816
C	-2.49862	2.010527	-0.19282
C	-1.77128	2.670491	-1.3432
C	-0.26803	2.813381	-1.14219
C	0.25301	2.004153	0.070268
C	-4.02766	2.037378	-0.2749
C	-2.34311	3.099882	-2.47045
O	0.012371	4.202759	-0.951
O	-0.02675	-0.07212	1.355935
C	1.761033	2.050223	0.107333
C	2.469735	2.404427	1.186179
C	3.971439	2.455627	1.274611
C	4.588724	1.163222	1.883417
C	5.986432	1.446445	2.467132
C	4.658623	0.025947	0.856434
N	5.323456	0.324314	-0.20259
C	4.001776	-1.32825	1.143435
C	4.544918	-2.35915	0.137704
C	4.611522	-1.7528	-1.25369
O	5.483411	-0.59767	-1.23354
C	4.259942	-1.82191	2.570882
O	2.576544	-1.21144	1.032592
O	5.069942	-2.59102	-2.26461
C	6.366242	-3.15871	-2.09042
H	-7.52658	-1.26286	-1.17677
H	-5.24373	-1.56063	-2.01365
H	-3.74584	-1.63146	2.010842
H	-6.05761	-1.32753	2.864111
H	-10.1596	-0.87001	1.30162
H	-9.22676	-0.11786	-0.02186
H	-9.43835	-1.89422	0.029567
H	-2.90499	-2.46476	-1.4386
H	-2.24688	-2.16502	0.168205
H	-2.92961	0.029976	-1.85264
H	-0.87096	-1.36526	-2.49707
H	-2.29531	0.205987	0.973838
H	-2.23364	2.573136	0.714622
H	0.247611	2.456991	-2.04979
H	-0.11655	2.502035	0.975024
H	-4.46785	1.571848	0.612694
H	-4.3826	3.072077	-0.33012
H	-4.41855	1.505906	-1.1486
H	-3.41504	3.050476	-2.63657
H	-1.75011	3.549354	-3.2629
H	0.940286	4.256646	-0.6612
H	0.942074	-0.20895	1.410525
H	2.282837	1.785788	-0.81223
H	1.929682	2.638708	2.106338
H	4.419445	2.620251	0.2899

H	4.259802	3.303063	1.910609
H	3.935179	0.837964	2.699831
H	6.426381	0.544894	2.908278
H	6.661348	1.797758	1.680242
H	5.931719	2.212327	3.249649
H	3.894445	-3.24014	0.136434
H	5.549216	-2.66901	0.448097
H	3.622225	-1.42355	-1.5921
H	3.83598	-2.82544	2.680251
H	5.331955	-1.86897	2.787721
H	3.777359	-1.1757	3.308219
H	2.321531	-0.99492	0.106845
H	6.626259	-3.60554	-3.05261
H	7.107544	-2.395	-1.83385
H	6.371043	-3.94649	-1.32486

Conf 8

E_{ZPC}= -1764.461943 Hartree

B3LYP/6-31G(d)

Symbol	X	Y	Z
C	6.381476	-1.76644	0.357185
C	5.093611	-1.86314	0.892165
C	3.952855	-1.63884	0.116003
C	4.139261	-1.31351	-1.23853
C	5.410817	-1.21328	-1.78915
C	6.543517	-1.43799	-0.99352
O	7.747884	-1.31798	-1.62699
C	8.927344	-1.5393	-0.87194
C	2.564781	-1.72212	0.711752
C	2.145581	-0.43674	1.468904
N	0.804971	-0.5588	2.051046
C	1.973853	0.84431	0.608846
C	0.48963	0.787396	0.164713
C	-0.17964	0.073433	1.367003
O	-1.38392	-0.00322	1.62361
C	2.381228	2.14262	1.384443
C	1.822118	3.348783	0.657977
C	0.312098	3.341584	0.523904
C	-0.11313	2.132592	-0.33377
C	2.013055	2.203778	2.88729
C	2.587258	4.30771	0.132009
O	-0.20989	4.501409	-0.10954
O	0.43402	-0.15795	-0.91408
C	-1.60854	2.093467	-0.54146
C	-2.18149	1.889349	-1.73042
C	-3.65851	1.847223	-2.00863

C	-4.17836	0.43674	-2.41258
C	-5.47382	0.542529	-3.23926
C	-4.38995	-0.4656	-1.19148
N	-5.32145	-0.06094	-0.40286
C	-3.53537	-1.72392	-0.99676
C	-4.11569	-2.5632	0.154785
C	-4.59246	-1.65402	1.263426
O	-5.62477	-0.7818	0.753208
C	-3.44631	-2.57991	-2.26344
O	-2.18419	-1.33515	-0.69333
O	-5.14873	-2.40823	2.283791
C	-5.50139	-1.65709	3.442945
H	7.238336	-1.95513	0.994132
H	4.984507	-2.13228	1.941362
H	3.268675	-1.1413	-1.86676
H	5.553585	-0.96686	-2.83679
H	9.758595	-1.39077	-1.5637
H	9.016016	-0.8262	-0.04128
H	8.967018	-2.56183	-0.473
H	2.520156	-2.55475	1.428124
H	1.82374	-1.91667	-0.06632
H	2.875055	-0.2649	2.267983
H	0.56276	-1.2535	2.747338
H	2.596051	0.795666	-0.28804
H	3.476278	2.190703	1.330998
H	-0.14323	3.235527	1.523181
H	0.364089	2.278359	-1.31123
H	0.938198	2.132563	3.073439
H	2.49779	1.40756	3.460258
H	2.360284	3.158879	3.296155
H	3.670895	4.278996	0.214399
H	2.155623	5.139142	-0.41657
H	0.003566	5.25867	0.458514
H	-0.50283	-0.38123	-1.09893
H	-2.22981	2.243327	0.339952
H	-1.53604	1.736848	-2.59865
H	-4.23259	2.201448	-1.14618
H	-3.8732	2.532104	-2.84172
H	-3.4101	-0.02577	-3.04117
H	-5.83526	-0.44676	-3.54222
H	-6.26317	1.020543	-2.65159
H	-5.30385	1.1344	-4.14621
H	-3.34856	-3.25287	0.520104
H	-4.97725	-3.14765	-0.18781
H	-3.78251	-1.01045	1.648146
H	-2.88805	-3.49408	-2.03609
H	-4.44283	-2.85872	-2.62043
H	-2.91719	-2.05583	-3.06312
H	-2.17922	-0.80725	0.133628

H	-5.80733	-2.38393	4.198944
H	-4.64062	-1.084	3.817294
H	-6.3284	-0.96923	3.238561

Conf 9

E_{ZPC}= -1764.461884 Hartree

B3LYP/6-31G(d)

Symbol	X	Y	Z
C	-6.80891	-1.32445	-0.91676
C	-5.51926	-1.30175	-1.43051
C	-4.40012	-1.54578	-0.61571
C	-4.63039	-1.81959	0.73531
C	-5.92159	-1.84852	1.272373
C	-7.01989	-1.59778	0.443546
O	-8.32277	-1.60051	0.851789
C	-8.59699	-1.8774	2.215822
C	-2.99751	-1.49445	-1.17957
C	-2.47316	-0.05503	-1.41754
N	-1.16438	-0.07568	-2.06645
C	-2.19318	0.737141	-0.1072
C	-0.66258	0.575211	0.129309
C	-0.11251	0.211156	-1.2717
O	1.072151	0.140559	-1.60759
C	-2.62855	2.238613	-0.1437
C	-1.57634	3.026074	-0.89266
C	-0.2218	3.059121	-0.19103
C	-0.01934	1.85301	0.741569
C	-4.07668	2.457628	-0.59159
C	-1.75643	3.661731	-2.05464
O	-0.13772	4.223538	0.645493
O	-0.49564	-0.5689	0.970003
C	1.395687	1.636342	1.221831
C	2.475897	2.376512	0.963823
C	3.864443	2.13489	1.517677
C	4.250433	0.672036	1.844434
C	5.477773	0.642022	2.777452
C	4.531474	-0.15697	0.583523
N	5.443102	0.32915	-0.17485
C	3.74381	-1.45422	0.347698
C	4.066448	-1.99645	-1.05805
C	5.51422	-1.74942	-1.43481
O	5.832367	-0.36081	-1.34306
C	4.026892	-2.48287	1.446216
O	2.341053	-1.17063	0.422692
O	6.34509	-2.53939	-0.6259

C	7.730527	-2.41704	-0.92592
H	-7.67245	-1.14096	-1.54865
H	-5.3781	-1.09925	-2.49055
H	-3.78482	-2.02283	1.388445
H	-6.05393	-2.07048	2.325174
H	-9.68259	-1.82938	2.318805
H	-8.24971	-2.87888	2.502678
H	-8.13683	-1.13348	2.879755
H	-2.96494	-2.02748	-2.14042
H	-2.29587	-1.9992	-0.5073
H	-3.17984	0.476716	-2.06199
H	-1.01025	-0.46274	-2.98949
H	-2.71539	0.259006	0.72543
H	-2.57593	2.577709	0.900637
H	0.575667	3.078634	-0.94782
H	-0.6166	2.055708	1.643374
H	-4.75822	1.816459	-0.02236
H	-4.36871	3.499951	-0.42382
H	-4.23396	2.242497	-1.65335
H	-2.72159	3.718917	-2.5493
H	-0.92888	4.156861	-2.55975
H	-0.34585	4.98654	0.082773
H	0.442434	-0.84908	0.959255
H	1.493652	0.77843	1.880288
H	2.376829	3.267714	0.346829
H	4.606717	2.527026	0.81328
H	3.980407	2.733255	2.435073
H	3.417761	0.193237	2.367506
H	5.778969	-0.38528	3.010654
H	6.329071	1.140153	2.302403
H	5.256606	1.153259	3.721899
H	3.445466	-1.46804	-1.78987
H	3.828468	-3.06284	-1.12305
H	5.705515	-1.96353	-2.49711
H	5.076453	-2.78241	1.429798
H	3.774842	-2.08003	2.43178
H	3.401785	-3.3657	1.271783
H	2.10574	-0.56215	-0.31545
H	8.249615	-3.14459	-0.29779
H	7.926455	-2.6482	-1.98407
H	8.100394	-1.40936	-0.70924

Conf 10

E_{ZPC}= -1764.461749 Hartree

B3LYP/6-31G(d)

Symbol	X	Y	Z

C	-2.31605	-3.14562	-1.72031
C	-0.92903	-3.09764	-1.67565
C	-0.23759	-2.88688	-0.47161
C	-1.00019	-2.72426	0.690751
C	-2.40059	-2.76042	0.666863
C	-3.06635	-2.97669	-0.54827
O	-4.42025	-3.03244	-0.69804
C	-5.23956	-2.94057	0.462625
C	1.277093	-2.82771	-0.44134
C	1.842898	-1.4089	-0.21076
N	1.380779	-0.49193	-1.25886
C	3.394725	-1.29756	-0.33969
C	3.600688	0.12088	-0.91766
C	2.329259	0.348158	-1.73487
O	2.240978	1.172443	-2.63884
C	4.291109	-1.61927	0.890368
C	4.338294	-0.45679	1.863437
C	4.775229	0.850042	1.216906
C	3.736158	1.271891	0.151799
C	4.010155	-2.98121	1.532213
C	4.042433	-0.52566	3.164238
O	6.078985	0.732006	0.647397
O	4.744419	0.151295	-1.77547
C	2.420744	1.691487	0.757106
C	1.730699	2.771283	0.377441
C	0.322939	3.067421	0.811556
C	-0.63186	3.073481	-0.42723
C	-0.77331	4.494198	-1.00621
C	-1.99353	2.463173	-0.1113
N	-2.58585	2.950313	0.919813
C	-2.52162	1.34775	-1.0281
C	-3.9186	0.897869	-0.56507
C	-4.00719	1.027132	0.939322
O	-3.82567	2.40201	1.302254
C	-2.56305	1.794373	-2.49308
O	-1.59309	0.242649	-0.99869
O	-5.25655	0.62079	1.383717
C	-5.42915	0.691686	2.800261
H	-2.84413	-3.31226	-2.65389
H	-0.36591	-3.22828	-2.59648
H	-0.5001	-2.57689	1.646117
H	-2.95336	-2.64565	1.592819
H	-6.26166	-3.09889	0.114247
H	-4.98264	-3.71686	1.19484
H	-5.16899	-1.94909	0.92586
H	1.675638	-3.2058	-1.39034
H	1.656087	-3.48418	0.348432
H	1.513515	-1.05468	0.776595
H	0.393861	-0.34747	-1.46321

H	3.68702	-1.98303	-1.14633
H	5.306631	-1.67158	0.471889
H	4.832451	1.6312	1.984124
H	4.145583	2.127172	-0.40017
H	4.028623	-3.77768	0.780015
H	4.775046	-3.21153	2.28111
H	3.037395	-3.01343	2.0357
H	3.722121	-1.44338	3.648959
H	4.127289	0.349843	3.803798
H	5.96768	0.554292	-0.3055
H	4.515505	0.795869	-2.47439
H	1.994896	1.034174	1.514799
H	2.157964	3.433501	-0.37773
H	-0.00293	2.311793	1.533552
H	0.246663	4.036924	1.321521
H	-0.17175	2.436411	-1.18897
H	-1.35675	4.501326	-1.93321
H	-1.27234	5.148197	-0.28352
H	0.213064	4.915465	-1.23294
H	-4.1061	-0.12785	-0.89852
H	-4.6923	1.545171	-0.9916
H	-3.2063	0.452146	1.447918
H	-3.18968	2.683477	-2.61283
H	-1.56027	2.012062	-2.86944
H	-2.98209	0.983634	-3.09779
H	-1.74384	-0.28654	-0.19702
H	-6.40045	0.240116	3.012282
H	-4.6423	0.12628	3.321075
H	-5.41667	1.727481	3.150422

Conf 11

E_{ZPC}= -1764.461649 Hartree

B3LYP/6-31G(d)

Symbol	X	Y	Z
C	4.822434	-2.15007	-0.27542
C	3.790521	-1.3191	-0.69519
C	4.022156	0.026007	-1.03211
C	5.334367	0.499212	-0.93466
C	6.386828	-0.32053	-0.51715
C	6.13116	-1.65525	-0.18236
O	7.075786	-2.5488	0.236002
C	8.418176	-2.10479	0.343875
C	2.881962	0.933188	-1.44079
C	2.27799	1.709414	-0.24521
N	1.741355	0.839214	0.8005
C	1.03381	2.578127	-0.5822

C	-0.15651	1.62682	-0.30468
C	0.386063	0.759722	0.86126
O	-0.25783	0.056501	1.636093
C	0.995868	3.916118	0.228327
C	-0.38717	4.530633	0.141611
C	-1.48674	3.645541	0.677852
C	-1.53046	2.329362	-0.15531
C	1.451235	3.835492	1.706261
C	-0.62227	5.734213	-0.38388
O	-2.73061	4.319349	0.602626
O	-0.22226	0.738169	-1.42991
C	-2.63892	1.436292	0.350395
C	-3.80795	1.264532	-0.28545
C	-4.95629	0.422121	0.206931
C	-5.09732	-0.93707	-0.53777
C	-6.46521	-1.58211	-0.24957
C	-3.94815	-1.8847	-0.17118
N	-3.92437	-2.22074	1.069388
C	-2.97757	-2.36926	-1.2535
C	-1.9524	-3.33988	-0.65553
C	-1.66096	-2.93923	0.771765
O	-2.89652	-3.02927	1.534205
C	-3.72102	-3.03175	-2.42398
O	-2.20991	-1.25078	-1.76505
O	-0.755	-3.81296	1.338257
C	-0.27742	-3.40275	2.623632
H	4.641327	-3.19003	-0.0217
H	2.783533	-1.72217	-0.77436
H	5.551175	1.532769	-1.19813
H	7.39008	0.086831	-0.46349
H	8.992003	-2.96672	0.689341
H	8.813661	-1.77157	-0.62501
H	8.517229	-1.28866	1.072187
H	2.08319	0.363064	-1.92152
H	3.236307	1.677594	-2.16618
H	3.07418	2.329403	0.183325
H	2.317046	0.192643	1.325065
H	1.017562	2.833535	-1.64652
H	1.695564	4.595338	-0.27568
H	-1.26878	3.387441	1.728093
H	-1.77977	2.649204	-1.17589
H	0.840355	3.158403	2.309239
H	2.49068	3.506477	1.797313
H	1.379997	4.833318	2.151846
H	0.18702	6.340723	-0.78434
H	-1.62403	6.14706	-0.42007
H	-3.41933	3.646294	0.735775
H	-0.83985	0.002106	-1.23675
H	-2.47284	0.952725	1.311121

H	-3.95143	1.749656	-1.25595
H	-4.85007	0.211736	1.275821
H	-5.89369	0.979219	0.071181
H	-5.04646	-0.73118	-1.61637
H	-6.58419	-2.52175	-0.80003
H	-6.55884	-1.80448	0.817277
H	-7.28008	-0.91019	-0.54258
H	-1.04355	-3.32086	-1.26486
H	-2.34606	-4.3616	-0.6493
H	-1.32582	-1.89466	0.845971
H	-2.98805	-3.38911	-3.15446
H	-4.31758	-3.88359	-2.0802
H	-4.3878	-2.32938	-2.93438
H	-2.82252	-0.52568	-1.97372
H	0.549444	-4.07329	2.869651
H	0.07624	-2.36462	2.592739
H	-1.06136	-3.49477	3.38284

Conf 12

E_{ZPC}= -1764.461626 Hartree

B3LYP/6-31G(d)

Symbol	X	Y	Z
C	4.98777	-1.89557	-0.34663
C	3.910413	-1.10501	-0.7606
C	4.058933	0.256526	-1.04289
C	5.341553	0.813187	-0.89779
C	6.424305	0.045727	-0.4892
C	6.254467	-1.31861	-0.20856
O	7.378533	-1.98801	0.183507
C	7.268919	-3.37398	0.464568
C	2.873716	1.106117	-1.44667
C	2.241021	1.854131	-0.24838
N	1.745319	0.958263	0.796014
C	0.960443	2.670391	-0.57827
C	-0.18834	1.67019	-0.29868
C	0.395092	0.819932	0.859886
O	-0.2155	0.083788	1.631351
C	0.871034	4.004266	0.235826
C	-0.53723	4.560345	0.159795
C	-1.59532	3.627928	0.699627
C	-1.58873	2.315409	-0.13962
C	1.338739	3.940862	1.710776
C	-0.82602	5.754131	-0.36121
O	-2.86666	4.249738	0.635107
O	-0.22492	0.783936	-1.42748
C	-2.65692	1.37414	0.364981

C	-3.81848	1.156574	-0.27001
C	-4.92999	0.26419	0.217705
C	-5.01534	-1.09292	-0.53825
C	-6.35598	-1.79583	-0.25712
C	-3.82918	-1.99651	-0.17864
N	-3.78758	-2.3335	1.061202
C	-2.84507	-2.44255	-1.26592
C	-1.78099	-3.37383	-0.673
C	-1.50018	-2.96353	0.753661
O	-2.72794	-3.10409	1.520125
C	-3.56762	-3.13151	-2.43409
O	-2.12146	-1.29594	-1.77876
O	-0.55718	-3.8004	1.316139
C	-0.09598	-3.37311	2.602004
H	4.826892	-2.94868	-0.14545
H	2.933267	-1.56892	-0.87459
H	5.495855	1.867603	-1.11901
H	7.415427	0.476616	-0.38542
H	8.270153	-3.70196	0.749967
H	6.575209	-3.56524	1.294219
H	6.939499	-3.94016	-0.41681
H	2.100911	0.497146	-1.9219
H	3.187313	1.865329	-2.17571
H	3.011269	2.506128	0.179724
H	2.349856	0.333787	1.314809
H	0.929165	2.927305	-1.64186
H	1.538273	4.713291	-0.27114
H	-1.36021	3.37451	1.747341
H	-1.85529	2.629747	-1.15758
H	0.76168	3.236923	2.316244
H	2.392173	3.657566	1.795322
H	1.226861	4.933892	2.158653
H	-0.04535	6.39476	-0.76529
H	-1.84442	6.124657	-0.39
H	-3.52613	3.547701	0.766522
H	-0.81363	0.024871	-1.23401
H	-2.46814	0.893056	1.322725
H	-3.98302	1.639929	-1.23803
H	-4.81339	0.050404	1.284813
H	-5.88979	0.783344	0.087622
H	-4.97187	-0.8759	-1.61507
H	-6.43509	-2.73602	-0.81372
H	-6.44203	-2.02861	0.808154
H	-7.19741	-1.15621	-0.54733
H	-0.87608	-3.31876	-1.28595
H	-2.13513	-4.40992	-0.66645
H	-1.20691	-1.90651	0.82794
H	-2.82473	-3.4609	-3.16769
H	-4.1311	-4.00492	-2.08884

H	-4.26208	-2.45388	-2.94118
H	-2.76328	-0.60451	-2.01222
H	0.760887	-4.00645	2.844347
H	0.210147	-2.3199	2.57401
H	-0.87323	-3.50324	3.362461

Conf 13

E_{ZPC}= -1764.461617 Hartree

B3LYP/6-31G(d)

Symbol	X	Y	Z
C	4.399919	-2.48528	-0.54947
C	3.571977	-1.45036	-0.99756
C	4.008363	-0.12238	-1.03938
C	5.322231	0.144189	-0.61635
C	6.15988	-0.86886	-0.16953
C	5.702523	-2.19513	-0.13047
O	6.599656	-3.11785	0.324551
C	6.193505	-4.47603	0.383753
C	3.085237	0.99331	-1.47757
C	2.405486	1.720526	-0.29063
N	1.631162	0.80265	0.537519
C	1.336251	2.75562	-0.74007
C	-0.01902	1.987053	-0.639
C	0.295323	0.860251	0.383186
O	-0.50614	0.080509	0.912166
C	1.320961	4.086298	0.086551
C	0.52531	3.865595	1.35615
C	-0.95381	3.603039	1.09496
C	-1.18315	2.950385	-0.28416
C	2.699567	4.729585	0.266876
C	1.009294	3.895629	2.60127
O	-1.66775	4.846819	1.047111
O	-0.22981	1.352819	-1.89867
C	-2.59736	2.431834	-0.48667
C	-3.2224	1.464956	0.192155
C	-4.62333	1.006714	-0.11861
C	-4.66646	-0.2514	-1.02873
C	-6.11852	-0.62043	-1.38206
C	-3.93188	-1.4419	-0.40851
N	-4.38168	-1.81142	0.737509
C	-2.75769	-2.0709	-1.1685
C	-2.26072	-3.32128	-0.41955
C	-2.37644	-3.10364	1.071222
O	-3.76127	-2.87257	1.407984
C	-3.13588	-2.44579	-2.60543

O	-1.70158	-1.10351	-1.28531
O	-1.9642	-4.23999	1.748006
C	-1.90984	-4.08989	3.164599
H	4.021887	-3.50124	-0.53943
H	2.564661	-1.69137	-1.3299
H	5.697858	1.165209	-0.64598
H	7.176418	-0.66176	0.150501
H	7.050446	-5.0279	0.774182
H	5.337084	-4.61266	1.057385
H	5.934897	-4.86445	-0.61022
H	2.295599	0.609465	-2.13203
H	3.64545	1.745218	-2.04898
H	3.182645	2.178497	0.328516
H	2.055107	0.017868	1.017525
H	1.496529	3.010579	-1.79238
H	0.733279	4.792798	-0.5156
H	-1.36371	2.960586	1.88705
H	-1.07038	3.765376	-1.00851
H	3.35613	4.160525	0.932886
H	3.209863	4.829347	-0.69838
H	2.593238	5.732417	0.694071
H	2.044074	4.138645	2.82478
H	0.37433	3.672421	3.456627
H	-1.52513	5.287147	1.900188
H	-0.97916	0.726924	-1.81938
H	-3.1538	2.940486	-1.27444
H	-2.69652	0.93064	0.979372
H	-5.16156	0.778856	0.809234
H	-5.17124	1.808046	-0.63119
H	-4.13494	0.014484	-1.94827
H	-6.15888	-1.46682	-2.07722
H	-6.66935	-0.90147	-0.47893
H	-6.62968	0.227607	-1.85302
H	-1.22517	-3.52574	-0.70868
H	-2.87258	-4.19476	-0.67187
H	-1.80756	-2.21701	1.403546
H	-2.26993	-2.90979	-3.08933
H	-3.96741	-3.15727	-2.61947
H	-3.41298	-1.56416	-3.1885
H	-1.41949	-0.81926	-0.38829
H	-1.47199	-5.01146	3.554996
H	-1.27364	-3.23784	3.44564
H	-2.90808	-3.94701	3.590732

Conf 14

E_{ZPC}= -1764.461516 Hartree

B3LYP/6-31G(d)

Symbol	X	Y	Z
C	-6.80039	-1.42745	-0.57833
C	-5.5184	-1.42747	-1.13452
C	-4.37322	-1.60547	-0.35188
C	-4.55194	-1.79355	1.028764
C	-5.81822	-1.79809	1.601436
C	-6.95412	-1.61272	0.800773
O	-8.1528	-1.63482	1.45448
C	-9.33589	-1.46028	0.692194
C	-2.99126	-1.5766	-0.96688
C	-2.48402	-0.14829	-1.29356
N	-1.19342	-0.19485	-1.97589
C	-2.17521	0.713931	-0.03485
C	-0.63812	0.570789	0.169262
C	-0.1227	0.138851	-1.22558
O	1.052844	0.058098	-1.58971
C	-2.61915	2.209645	-0.14027
C	-1.58963	2.95994	-0.95576
C	-0.21869	3.035998	-0.29008
C	0.01352	1.881075	0.698572
C	-4.07916	2.399259	-0.56268
C	-1.80072	3.530034	-2.14611
O	-0.12111	4.242908	0.482139
O	-0.44329	-0.52833	1.062132
C	1.441277	1.694552	1.152992
C	2.512215	2.421956	0.828773
C	3.914605	2.212389	1.36086
C	4.312699	0.769259	1.753478
C	5.559444	0.788082	2.660689
C	4.569286	-0.12195	0.530385
N	5.462305	0.327527	-0.27148
C	3.780408	-1.43092	0.377309
C	4.0741	-2.04273	-1.00592
C	5.512559	-1.81162	-1.42614
O	5.82761	-0.41938	-1.41212
C	4.088283	-2.40277	1.520237
O	2.378717	-1.14668	0.468418
O	6.363381	-2.55686	-0.59599
C	7.741862	-2.44429	-0.92993
H	-7.66075	-1.29083	-1.2237
H	-5.41825	-1.29408	-2.21019
H	-3.68142	-1.94621	1.662519
H	-5.95386	-1.94893	2.667912
H	-10.1616	-1.51317	1.404167
H	-9.35327	-0.48376	0.189865
H	-9.45395	-2.25297	-0.05871
H	-2.98921	-2.15628	-1.9008
H	-2.26355	-2.04462	-0.29574
H	-3.21257	0.343339	-1.94554

H	-1.06114	-0.63012	-2.88065
H	-2.67342	0.278917	0.835119
H	-2.54293	2.604562	0.882788
H	0.559891	3.019601	-1.06631
H	-0.56199	2.127469	1.603559
H	-4.74297	1.790513	0.060653
H	-4.37041	3.448913	-0.44734
H	-4.26315	2.122896	-1.60598
H	-2.77782	3.555797	-2.6195
H	-0.98845	4.000244	-2.69768
H	-0.34713	4.974456	-0.11441
H	0.496193	-0.8035	1.042791
H	1.558981	0.873105	1.853464
H	2.395573	3.278888	0.167504
H	4.638635	2.569371	0.619778
H	4.050071	2.857771	2.243044
H	3.492943	0.315589	2.317601
H	5.866008	-0.22593	2.94073
H	6.400314	1.260421	2.142706
H	5.357697	1.347889	3.581657
H	3.435651	-1.55326	-1.74974
H	3.838292	-3.11155	-1.01183
H	5.682083	-2.07907	-2.48004
H	5.137749	-2.70216	1.498246
H	3.854877	-1.95121	2.489171
H	3.461325	-3.29364	1.403009
H	2.124576	-0.57589	-0.29318
H	8.276616	-3.14052	-0.27982
H	7.916905	-2.7233	-1.98018
H	8.111884	-1.42654	-0.76753

Conf 15

E_{ZPC}= -1764.461511 Hartree

B3LYP/6-31G(d)

Symbol	X	Y	Z
C	5.461658	-1.51993	-1.42515
C	4.601271	-0.45826	-1.68092
C	4.429537	0.585487	-0.75562
C	5.160943	0.521558	0.434941
C	6.031273	-0.53572	0.710352
C	6.183766	-1.56731	-0.22425
O	7.003813	-2.64609	-0.06558
C	7.770198	-2.73844	1.125138
C	3.458119	1.716599	-1.01835
C	2.079993	1.508297	-0.35242
N	1.425075	0.29993	-0.84014

C	1.049676	2.634599	-0.66808
C	-0.20336	1.944817	-1.2823
C	0.189391	0.442707	-1.36152
O	-0.48232	-0.47265	-1.84655
C	0.707902	3.526928	0.55062
C	-0.08758	2.752135	1.594606
C	-1.16323	1.810261	1.045042
C	-1.48233	2.178366	-0.44244
C	1.934935	4.267892	1.094595
C	0.085273	2.872909	2.913057
O	-2.29608	1.841734	1.887424
O	-0.36049	2.432642	-2.61393
C	-2.66676	1.429742	-0.99333
C	-3.94857	1.670517	-0.70162
C	-5.03527	0.702728	-1.08673
C	-5.29986	-0.36297	0.033007
C	-5.83834	0.291868	1.315577
C	-4.07691	-1.24911	0.273178
N	-3.35504	-0.91767	1.285594
C	-3.75149	-2.41575	-0.6796
C	-2.78693	-3.37568	0.033159
C	-1.66951	-2.60723	0.696004
O	-2.21051	-1.64123	1.633724
C	-5.00053	-3.18516	-1.11962
O	-3.18317	-1.91511	-1.89847
O	-0.87169	-3.48112	1.418383
C	0.300569	-2.89233	1.972665
H	5.597686	-2.32125	-2.14478
H	4.061056	-0.43105	-2.62485
H	5.05971	1.318193	1.169332
H	6.583304	-0.54055	1.64336
H	8.345926	-3.66164	1.039235
H	8.459273	-1.88983	1.22857
H	7.129393	-2.7928	2.015281
H	3.302543	1.840527	-2.09706
H	3.868885	2.661819	-0.64266
H	2.224438	1.419391	0.732477
H	1.892929	-0.59872	-0.85377
H	1.462525	3.274392	-1.45528
H	0.034391	4.302826	0.155393
H	-0.75427	0.786305	1.044158
H	-1.69906	3.253371	-0.47552
H	2.650709	3.589238	1.573055
H	2.452439	4.796022	0.285554
H	1.641794	5.013421	1.840329
H	0.840758	3.520837	3.346497
H	-0.5585	2.335413	3.601374
H	-2.76019	0.98612	1.781858
H	-1.14623	1.994895	-2.98449

H	-2.4473	0.561348	-1.60471
H	-4.21493	2.514987	-0.06772
H	-5.98333	1.224033	-1.27271
H	-4.75807	0.176891	-2.00544
H	-6.09111	-1.0156	-0.35355
H	-6.04871	-0.45899	2.083824
H	-5.13258	1.009476	1.740419
H	-6.77047	0.82368	1.090797
H	-2.37404	-4.0815	-0.69415
H	-3.31353	-3.9444	0.80842
H	-1.06487	-2.04444	-0.03297
H	-4.6906	-4.06803	-1.68754
H	-5.593	-3.51243	-0.2588
H	-5.62714	-2.57286	-1.77303
H	-2.30521	-1.51843	-1.73895
H	0.899757	-3.71387	2.371667
H	0.879807	-2.36397	1.200575
H	0.055893	-2.19135	2.77763

Conf 16

E_{ZPC}= -1764.461361 Hartree

B3LYP/6-31G(d)

Symbol	X	Y	Z
C	4.307213	-2.62376	-0.58642
C	3.49091	-1.58739	-1.02425
C	3.957055	-0.26266	-1.08371
C	5.277761	-0.02333	-0.69059
C	6.114959	-1.05142	-0.24918
C	5.627599	-2.36264	-0.1933
O	6.349703	-3.44525	0.219244
C	7.694984	-3.24225	0.620118
C	3.047676	0.868479	-1.51154
C	2.388234	1.602259	-0.31761
N	1.596197	0.69845	0.508915
C	1.343003	2.666515	-0.75577
C	-0.03254	1.936819	-0.64177
C	0.260746	0.796125	0.371169
O	-0.55648	0.037179	0.90581
C	1.375603	3.9965	0.071547
C	0.591872	3.794175	1.351329
C	-0.89738	3.573537	1.109428
C	-1.16331	2.932583	-0.26855
C	2.77377	4.601532	0.232714
C	1.094145	3.805588	2.589487
O	-1.57673	4.836997	1.075406
O	-0.27632	1.315224	-1.90161

C	-2.59465	2.456588	-0.45477
C	-3.23762	1.502572	0.225501
C	-4.65504	1.086069	-0.0688
C	-4.74632	-0.16422	-0.98608
C	-6.21308	-0.49049	-1.32013
C	-4.03668	-1.37895	-0.38394
N	-4.48041	-1.74248	0.766398
C	-2.89219	-2.03701	-1.16416
C	-2.42541	-3.3087	-0.4322
C	-2.51155	-3.09999	1.061844
O	-3.88283	-2.82515	1.422114
C	-3.29884	-2.3875	-2.59949
O	-1.80781	-1.10152	-1.28575
O	-2.12802	-4.2552	1.722767
C	-2.04228	-4.119	3.139134
H	3.946693	-3.64699	-0.54667
H	2.473044	-1.80956	-1.33661
H	5.674084	0.989413	-0.73471
H	7.134426	-0.82111	0.038914
H	8.074093	-4.22533	0.905062
H	8.305577	-2.84257	-0.20049
H	7.762564	-2.56467	1.481843
H	2.247888	0.499885	-2.16262
H	3.615806	1.613796	-2.08358
H	3.178909	2.037462	0.300948
H	2.002454	-0.0981	0.984887
H	1.499276	2.917592	-1.80963
H	0.799009	4.719606	-0.52154
H	-1.31431	2.939807	1.904836
H	-1.0358	3.74649	-0.99169
H	3.424003	4.014463	0.889284
H	3.272607	4.687376	-0.73981
H	2.701167	5.606827	0.661235
H	2.138361	4.019148	2.798792
H	0.46554	3.596582	3.453106
H	-1.41266	5.268795	1.928974
H	-1.04081	0.708625	-1.81586
H	-3.14677	2.986883	-1.23122
H	-2.71668	0.947911	1.001782
H	-5.18628	0.866757	0.865113
H	-5.18749	1.905978	-0.56825
H	-4.22163	0.092819	-1.91207
H	-6.28688	-1.33138	-2.01916
H	-6.75842	-0.76159	-0.41066
H	-6.70716	0.374188	-1.7789
H	-1.40149	-3.54479	-0.73808
H	-3.06926	-4.15953	-0.68225
H	-1.90827	-2.23584	1.392795
H	-2.45385	-2.8749	-3.09726

H	-4.15276	-3.07206	-2.60948
H	-3.55449	-1.49273	-3.1723
H	-1.50691	-0.83405	-0.38985
H	-1.62862	-5.05802	3.513878
H	-1.37233	-3.29142	3.414728
H	-3.02707	-3.94613	3.585169

Conf 17

E_{ZPC}= -1764.461014 Hartree

B3LYP/6-31G(d)

Symbol	X	Y	Z
C	7.125747	0.5651	0.479864
C	6.272262	-0.5296	0.521911
C	5.284086	-0.7285	-0.45784
C	5.193728	0.214875	-1.48538
C	6.044364	1.323286	-1.54696
C	7.016835	1.503346	-0.55808
O	7.901886	2.542273	-0.51452
C	7.846799	3.512329	-1.54814
C	4.341301	-1.90936	-0.39249
C	3.206179	-1.75591	0.654506
N	2.377896	-2.95598	0.711153
C	2.177821	-0.64293	0.29636
C	1.013673	-1.40195	-0.39105
C	1.164403	-2.86292	0.105074
O	0.361569	-3.77304	-0.0533
C	1.677535	0.226262	1.498845
C	0.596015	-0.52546	2.245114
C	-0.65732	-0.7803	1.419313
C	-0.37611	-0.79436	-0.09813
C	2.806458	0.797245	2.362401
C	0.669112	-0.95215	3.509803
O	-1.61004	0.293135	1.627792
O	1.274562	-1.39289	-1.79687
C	-1.49754	-1.45187	-0.86661
C	-2.29785	-0.77906	-1.70111
C	-3.51052	-1.34594	-2.39366
C	-4.79854	-1.28867	-1.52282
C	-6.02518	-1.68514	-2.36451
C	-4.99078	0.069169	-0.84717
N	-4.82519	1.088629	-1.61193
C	-5.3259	0.092985	0.652371
C	-5.48866	1.550146	1.132368
C	-4.56821	2.451706	0.345475
O	-4.91913	2.373902	-1.05131
C	-6.6106	-0.69287	0.949143

O	-4.30293	-0.58773	1.382232
O	-4.72774	3.766832	0.749494
C	-3.81631	4.681285	0.146721
H	7.892141	0.713303	1.234444
H	6.382342	-1.25194	1.328613
H	4.446878	0.082447	-2.26492
H	5.941141	2.02608	-2.36588
H	8.632401	4.235685	-1.32224
H	8.038042	3.065121	-2.53267
H	6.876511	4.026294	-1.56922
H	4.903932	-2.82039	-0.14423
H	3.873553	-2.07797	-1.36833
H	3.654876	-1.58035	1.637358
H	2.740764	-3.86293	0.977289
H	2.62243	0.037746	-0.4339
H	1.17907	1.089267	1.03338
H	-1.11443	-1.73015	1.725413
H	-0.32024	0.25404	-0.41821
H	3.553922	1.296176	1.736499
H	2.408258	1.532118	3.070404
H	3.326655	0.029791	2.944666
H	1.522298	-0.74552	4.148901
H	-0.13376	-1.53629	3.95569
H	-1.74113	0.369912	2.587693
H	0.500825	-1.80238	-2.22449
H	-1.65115	-2.51389	-0.68149
H	-2.11965	0.285364	-1.85105
H	-3.69012	-0.79401	-3.32292
H	-3.3411	-2.39646	-2.66252
H	-4.6657	-2.01375	-0.71269
H	-6.93579	-1.73673	-1.75884
H	-6.19031	-0.95164	-3.16096
H	-5.87306	-2.66804	-2.82662
H	-5.27415	1.59886	2.204574
H	-6.51141	1.906272	0.965716
H	-3.51209	2.13358	0.433573
H	-7.46578	-0.27667	0.407703
H	-6.49659	-1.74737	0.685159
H	-6.81473	-0.63711	2.023831
H	-3.42487	-0.19817	1.190268
H	-3.9785	5.644706	0.635436
H	-2.77533	4.363925	0.308562
H	-3.99741	4.778274	-0.92859

Conf 18

E_{ZPC}= -1764.460671 Hartree

B3LYP/6-31G(d)

Symbol	X	Y	Z
C	6.661819	-0.92605	0.616349
C	5.428293	-0.96144	1.252078
C	4.259544	-1.33799	0.567785
C	4.383189	-1.68728	-0.77954
C	5.616604	-1.65832	-1.43906
C	6.764778	-1.27311	-0.73958
O	8.019429	-1.20884	-1.27436
C	8.18407	-1.55727	-2.63948
C	2.918077	-1.34492	1.265869
C	2.294898	0.062555	1.455201
N	1.078524	-0.01903	2.258311
C	1.804537	0.713285	0.125313
C	0.252537	0.558625	0.140639
C	-0.08628	0.14537	1.589467
O	-1.21299	-0.03836	2.044571
C	2.269441	2.184747	-0.09111
C	1.472511	3.094751	0.815781
C	-0.02982	3.074188	0.56346
C	-0.47373	1.857245	-0.29894
C	3.78996	2.362033	-0.04005
C	1.98354	3.866191	1.777592
O	-0.37309	4.290898	-0.1033
O	-0.05416	-0.53652	-0.71501
C	-1.98116	1.788236	-0.30264
C	-2.74845	2.044019	-1.37312
C	-4.25751	2.066362	-1.37321
C	-4.91047	0.753881	-1.89242
C	-6.40276	0.968954	-2.20561
C	-4.71706	-0.39156	-0.8904
N	-5.22955	-0.16817	0.267175
C	-3.98833	-1.66786	-1.31997
C	-4.0078	-2.69712	-0.18443
C	-3.91382	-1.97451	1.139025
O	-5.07733	-1.10976	1.274697
C	-4.59364	-2.26938	-2.59762
O	-2.59042	-1.37396	-1.55897
O	-3.94353	-2.87777	2.182405
C	-3.64622	-2.30218	3.457887
H	7.563774	-0.63922	1.148148
H	5.371491	-0.69815	2.306602
H	3.500606	-1.99959	-1.33321
H	5.666557	-1.9421	-2.48415
H	9.247603	-1.4365	-2.85338
H	7.892322	-2.59891	-2.82872
H	7.604745	-0.89631	-3.29791
H	3.022947	-1.8042	2.259136
H	2.198451	-1.95441	0.707237
H	3.014729	0.709606	1.96731

H	1.069497	-0.40648	3.194227
H	2.174848	0.123886	-0.71725
H	1.970846	2.433178	-1.12059
H	-0.55246	3.024048	1.532087
H	-0.1412	2.052567	-1.32649
H	4.210912	2.162925	0.950769
H	4.279697	1.68258	-0.74532
H	4.057728	3.388837	-0.31121
H	3.051337	3.93782	1.96162
H	1.341669	4.479774	2.404665
H	-1.30939	4.205315	-0.35652
H	-0.99153	-0.79691	-0.60622
H	-2.45462	1.575627	0.654364
H	-2.2621	2.241762	-2.3326
H	-4.63798	2.252522	-0.36415
H	-4.60127	2.891439	-2.01162
H	-4.40699	0.481819	-2.83045
H	-6.86265	0.055206	-2.59768
H	-6.94029	1.25085	-1.29527
H	-6.53313	1.762226	-2.95086
H	-3.17038	-3.38966	-0.31387
H	-4.94071	-3.27059	-0.19771
H	-3.03081	-1.32132	1.188208
H	-4.06103	-3.19423	-2.84172
H	-5.65513	-2.50256	-2.46038
H	-4.49758	-1.5925	-3.45259
H	-2.52773	-0.55716	-2.08263
H	-3.52396	-3.13947	4.149258
H	-2.72065	-1.71462	3.412666
H	-4.46502	-1.66095	3.801861

Table S7. Atomic coordinates of the lowest energy conformer of (3*S*,4*S*,5*S*,7*S*,8*S*,9*S*,13*E*,16*R*,18*S*,20*R*)-**1**.

Conf 1

E_{ZPC}= -1764.45944 Hartree

B3LYP/6-31G(d)

Symbol	X	Y	Z
C	4.607417	-0.43446	0.102035
C	3.40839	0.066801	-0.4157
C	3.30138	1.383036	-0.88085
C	4.449718	2.189919	-0.82038
C	5.65143	1.706817	-0.31669
C	5.7377	0.388278	0.153272
O	6.959762	0.007242	0.634199
C	7.100868	-1.31509	1.124775
C	1.976164	1.925562	-1.37093
C	1.118104	2.508479	-0.21918

N	0.858951	1.536134	0.841624
C	-0.33012	2.917369	-0.61233
C	-1.14574	1.628232	-0.35414
C	-0.38246	0.981878	0.836667
O	-0.76748	0.090556	1.588846
C	-0.84389	4.176452	0.164187
C	-2.35259	4.284137	0.032921
C	-3.10205	3.08585	0.565725
C	-2.6806	1.827577	-0.22629
C	-0.42846	4.281744	1.652037
C	-2.96678	5.327027	-0.52823
O	-4.50367	3.289803	0.45469
O	-0.90335	0.774197	-1.47754
C	-3.34471	0.583883	0.304393
C	-3.95799	-0.33829	-0.44642
C	-4.43589	-1.65484	0.096717
C	-3.64837	-2.89805	-0.43361
C	-3.96999	-3.17834	-1.91232
C	-2.13758	-2.83902	-0.19318
N	-1.45247	-2.20433	-1.07746
C	-1.55241	-3.55411	1.030254
C	-0.03496	-3.30558	1.116841
C	0.517292	-3.3301	-0.28517
O	-0.0544	-2.22104	-1.01392
C	-2.22759	-3.09007	2.328831
O	-1.8204	-4.94969	0.793835
O	1.893172	-3.16582	-0.297
C	2.492592	-3.41249	-1.57071
H	4.638591	-1.45861	0.45707
H	2.544468	-0.59221	-0.45382
H	4.40272	3.215768	-1.1808
H	6.539023	2.33095	-0.27691
H	8.137521	-1.40378	1.455465
H	6.431932	-1.50566	1.974808
H	6.905593	-2.06055	0.342052
H	1.396482	1.143421	-1.86714
H	2.143364	2.729249	-2.1002
H	1.660338	3.36206	0.204352
H	1.60576	1.098104	1.366365
H	-0.39055	3.141493	-1.68202
H	-0.40358	5.044892	-0.34258
H	-2.83864	2.937169	1.627352
H	-3.01274	2.000304	-1.2583
H	0.658526	4.31547	1.771202
H	-0.83683	5.209412	2.067097
H	-0.79784	3.453526	2.262667
H	-2.40134	6.16624	-0.92718
H	-4.04782	5.370921	-0.59814
H	-4.91787	2.412869	0.515335

H	-1.29335	-0.10649	-1.30354
H	-3.21877	0.397466	1.369426
H	-4.06128	-0.17095	-1.51903
H	-4.38401	-1.62711	1.190446
H	-5.49093	-1.82065	-0.16564
H	-4.00588	-3.75868	0.14349
H	-5.05176	-3.29522	-2.04709
H	-3.62216	-2.37072	-2.56139
H	-3.48273	-4.10102	-2.24331
H	0.431799	-4.08061	1.735743
H	0.169359	-2.32379	1.556312
H	0.218523	-4.25369	-0.80986
H	-1.75594	-3.59854	3.180589
H	-2.09857	-2.01314	2.469211
H	-3.29253	-3.33833	2.336347
H	-1.65296	-5.4191	1.628126
H	2.142178	-2.69866	-2.32264
H	3.569364	-3.29687	-1.43328
H	2.274801	-4.43521	-1.90979

Conf 2

E_{ZPC}= -1764.459067 Hartree

B3LYP/6-31G(d)

Symbol	X	Y	Z
C	4.657733	-0.60079	0.124827
C	3.464796	-0.07598	-0.38411
C	3.387083	1.233511	-0.87348
C	4.558503	2.008563	-0.84584
C	5.75397	1.50197	-0.35104
C	5.811214	0.190627	0.142888
O	7.029578	-0.21522	0.611678
C	7.142428	-1.5308	1.127522
C	2.072694	1.806169	-1.35843
C	1.241883	2.430166	-0.20883
N	0.934003	1.471045	0.851003
C	-0.18374	2.907705	-0.60624
C	-1.0616	1.660135	-0.34981
C	-0.33193	0.97441	0.84001
O	-0.76013	0.09715	1.584804
C	-0.63855	4.190072	0.168215
C	-2.139	4.372728	0.027058
C	-2.95028	3.215305	0.558981
C	-2.58567	1.933624	-0.22475
C	-0.22728	4.272962	1.658509
C	-2.69688	5.442527	-0.54218
O	-4.33905	3.487363	0.434761

O	-0.85954	0.794631	-1.47353
C	-3.31181	0.727438	0.311583
C	-3.97757	-0.16206	-0.43408
C	-4.53048	-1.44629	0.115776
C	-3.82699	-2.73603	-0.42144
C	-4.18437	-2.99515	-1.89589
C	-2.3132	-2.75943	-0.19227
N	-1.60492	-2.17722	-1.09623
C	-1.76116	-3.49959	1.034888
C	-0.23188	-3.33104	1.109999
C	0.329444	-3.35835	-0.28929
O	-0.204	-2.24306	-1.02699
C	-2.39684	-3.00379	2.335971
O	-2.13064	-4.88873	0.93875
O	1.708439	-3.23904	-0.28702
C	2.315517	-3.47187	-1.56011
H	4.668021	-1.61857	0.498996
H	2.582015	-0.71076	-0.39645
H	4.534414	3.028118	-1.22575
H	6.658952	2.101718	-0.33682
H	8.181232	-1.64086	1.444603
H	6.481755	-1.68551	1.991122
H	6.915424	-2.28637	0.363255
H	1.464716	1.035578	-1.83859
H	2.256824	2.593992	-2.10076
H	1.82352	3.257118	0.215231
H	1.656992	1.001208	1.381553
H	-0.23056	3.134329	-1.67611
H	-0.15209	5.035563	-0.33517
H	-2.70346	3.059206	1.623464
H	-2.90713	2.117064	-1.2583
H	0.859389	4.255013	1.783746
H	-0.59377	5.217984	2.073414
H	-0.63864	3.461727	2.265028
H	-2.08786	6.251258	-0.93993
H	-3.77391	5.539626	-0.6199
H	-4.79741	2.633679	0.507804
H	-1.30097	-0.06085	-1.30082
H	-3.19405	0.539175	1.377237
H	-4.07442	0.007479	-1.50698
H	-4.46425	-1.42337	1.208472
H	-5.59653	-1.54666	-0.13497
H	-4.22741	-3.57218	0.163657
H	-5.27276	-3.04619	-2.01585
H	-3.79703	-2.21039	-2.55044
H	-3.76146	-3.94575	-2.23849
H	0.189191	-4.13406	1.722929
H	0.019375	-2.3624	1.554227
H	0.023156	-4.27559	-0.83356

H	-1.94503	-3.54631	3.173127
H	-2.21302	-1.93422	2.465636
H	-3.47158	-3.20281	2.35165
H	-1.74965	-5.247	0.120568
H	1.993326	-2.73163	-2.29862
H	3.393062	-3.38965	-1.40712
H	2.074528	-4.48077	-1.92491

Conf 3

E_{ZPC}= -1764.458791 Hartree

B3LYP/6-31G(d)

Symbol	X	Y	Z
C	-6.75602	0.249963	-1.71371
C	-5.84618	-0.65127	-1.17278
C	-5.33427	-0.48689	0.125585
C	-5.7767	0.618009	0.860334
C	-6.69121	1.534261	0.335484
C	-7.18539	1.352551	-0.96194
O	-8.08153	2.178283	-1.57676
C	-8.55799	3.306059	-0.85999
C	-4.31082	-1.44816	0.691774
C	-2.85391	-0.94563	0.563464
N	-2.4821	-0.73144	-0.83178
C	-1.79356	-1.97487	1.072435
C	-0.75999	-2.13965	-0.07354
C	-1.41714	-1.44318	-1.28099
O	-1.03791	-1.5518	-2.43958
C	-1.14413	-1.59582	2.423625
C	-0.32521	-0.31712	2.293184
C	0.461935	-0.16618	0.991234
C	0.633066	-1.53763	0.284495
C	-2.15137	-1.59166	3.57987
C	-0.25117	0.628099	3.233549
O	1.709813	0.459727	1.279516
O	-0.61699	-3.51972	-0.38876
C	1.564816	-1.43134	-0.89157
C	2.700032	-2.12484	-1.02273
C	3.71435	-1.87633	-2.10498
C	5.008608	-1.20307	-1.5631
C	5.997883	-0.90972	-2.70724
C	4.703045	0.069862	-0.77151
N	3.887133	0.868128	-1.35624
C	5.375159	0.284542	0.594576
C	4.990091	1.649441	1.192124
C	4.653291	2.62254	0.089346
O	3.561173	2.073278	-0.69222

C	5.003829	-0.82583	1.587952
O	6.802373	0.330017	0.391505
O	4.225934	3.821105	0.626419
C	4.033646	4.861219	-0.32994
H	-7.15375	0.117117	-2.71501
H	-5.53424	-1.50836	-1.76576
H	-5.40704	0.771408	1.872241
H	-7.01129	2.373184	0.942854
H	-9.25187	3.815517	-1.53105
H	-9.08999	3.009114	0.053664
H	-7.74194	3.991397	-0.59485
H	-4.38451	-2.41733	0.183185
H	-4.5106	-1.62872	1.755112
H	-2.76179	-0.00034	1.115396
H	-3.08944	-0.24374	-1.47938
H	-2.28121	-2.94967	1.184198
H	-0.43127	-2.40824	2.636351
H	-0.11179	0.4877	0.312791
H	1.075921	-2.23699	1.004877
H	-2.71055	-2.53389	3.602468
H	-1.64435	-1.48666	4.544285
H	-2.87094	-0.76898	3.494795
H	-0.80153	0.565408	4.166872
H	0.392641	1.490793	3.098227
H	2.036333	0.901576	0.475914
H	-0.18607	-3.53981	-1.26291
H	1.309145	-0.69803	-1.65476
H	2.966001	-2.86049	-0.26049
H	4.009669	-2.81728	-2.5905
H	3.282513	-1.23063	-2.87598
H	5.486604	-1.91629	-0.87818
H	6.265782	-1.8331	-3.23404
H	5.547135	-0.21809	-3.4262
H	6.915428	-0.44748	-2.32782
H	5.824599	2.013695	1.798188
H	4.105434	1.552369	1.829037
H	5.498353	2.76146	-0.60694
H	5.479916	-0.61073	2.550795
H	3.919277	-0.8629	1.728003
H	5.348611	-1.81099	1.253658
H	7.107625	-0.57401	0.213762
H	3.212878	4.628012	-1.01598
H	3.79295	5.761381	0.239592
H	4.95091	5.034459	-0.91129

Conf 4

E_{ZPC}= -1764.458748 Hartree

B3LYP/6-31G(d)

Symbol	X	Y	Z
C	-6.73711	0.701589	-1.35569
C	-5.86785	-0.33291	-0.99122
C	-5.26328	-0.37923	0.268415
C	-5.56173	0.656206	1.171727
C	-6.42127	1.6906	0.829628
C	-7.01588	1.722363	-0.44167
O	-7.84532	2.779416	-0.68097
C	-8.47617	2.8634	-1.94901
C	-4.28771	-1.47766	0.635457
C	-2.8089	-1.04224	0.52037
N	-2.47795	-0.64793	-0.84602
C	-1.78618	-2.18287	0.82849
C	-0.76931	-2.18045	-0.34524
C	-1.44482	-1.31194	-1.42353
O	-1.10239	-1.26877	-2.59789
C	-1.10824	-2.07067	2.212835
C	-0.25953	-0.80667	2.301164
C	0.466869	-0.41043	1.016666
C	0.63082	-1.6377	0.077903
C	-2.09663	-2.25134	3.371605
C	-0.11163	-0.0753	3.408693
O	1.713028	0.190741	1.355236
O	-0.63408	-3.49741	-0.86454
C	1.532416	-1.32159	-1.08275
C	2.672866	-1.96245	-1.35738
C	3.648002	-1.51555	-2.41222
C	4.940454	-0.89336	-1.8094
C	5.879991	-0.39399	-2.92419
C	4.629398	0.231764	-0.82094
N	3.758183	1.073358	-1.24214
C	5.360833	0.268833	0.531407
C	4.937957	1.49783	1.353327
C	4.517577	2.617345	0.433212
O	3.417994	2.149661	-0.39054
C	5.100847	-1.0032	1.350926
O	6.771401	0.429976	0.276128
O	4.062692	3.693456	1.169791
C	3.795756	4.864315	0.400958
H	-7.1874	0.694288	-2.3417
H	-5.66934	-1.12604	-1.7091
H	-5.11479	0.64758	2.163706
H	-6.65365	2.486648	1.530242
H	-9.07925	3.772779	-1.92364
H	-7.74134	2.938362	-2.76157
H	-9.12958	2.000221	-2.13297
H	-4.44091	-2.34946	-0.01232
H	-4.46337	-1.80896	1.666193

H	-2.64388	-0.19311	1.197943
H	-3.09066	-0.05823	-1.39623
H	-2.30982	-3.14384	0.772602
H	-0.41373	-2.92435	2.260061
H	-0.14929	0.333849	0.484038
H	1.093803	-2.44678	0.656808
H	-2.68626	-3.16445	3.232189
H	-1.57168	-2.34213	4.32773
H	-2.78807	-1.40497	3.454628
H	-0.6179	-0.3154	4.338228
H	0.549963	0.784163	3.415512
H	1.984683	0.78691	0.635253
H	-0.24244	-3.3838	-1.75014
H	1.248908	-0.47487	-1.70607
H	2.972329	-2.81036	-0.73733
H	3.954084	-2.35927	-3.04676
H	3.173919	-0.77329	-3.06182
H	5.460606	-1.68771	-1.25769
H	6.156885	-1.21554	-3.59538
H	5.382419	0.383085	-3.51332
H	6.795873	0.038258	-2.50733
H	5.777792	1.80161	1.984921
H	4.083646	1.254883	1.992704
H	5.327712	2.904929	-0.25871
H	5.610041	-0.91168	2.316423
H	4.028363	-1.13034	1.525354
H	5.483516	-1.90102	0.852164
H	7.110085	-0.41211	-0.06767
H	2.960952	4.708492	-0.29014
H	3.540003	5.649318	1.115748
H	4.684316	5.169616	-0.17053

Conf 5

E_{ZPC}= -1764.45873 Hartree

B3LYP/6-31G(d)

Symbol	X	Y	Z
C	4.64117	-0.55923	0.117074
C	3.447902	-0.03915	-0.39569
C	3.364333	1.273678	-0.87507
C	4.529682	2.057112	-0.83365
C	5.72554	1.555037	-0.33494
C	5.788768	0.240236	0.149
O	7.006938	-0.16074	0.622902
C	7.125732	-1.48044	1.12685
C	2.048	1.839758	-1.36245
C	1.209863	2.452815	-0.21229

N	0.914668	1.49001	0.848028
C	-0.22194	2.913482	-0.60815
C	-1.08415	1.65552	-0.34975
C	-0.34601	0.981117	0.840962
O	-0.76596	0.103361	1.590143
C	-0.69116	4.190895	0.166027
C	-2.19411	4.354761	0.027996
C	-2.98979	3.187512	0.562173
C	-2.61127	1.910421	-0.2221
C	-0.27787	4.280426	1.655404
C	-2.76672	5.417268	-0.54027
O	-4.38238	3.442526	0.441874
O	-0.8733	0.792453	-1.47308
C	-3.31895	0.694705	0.316881
C	-3.96948	-0.20683	-0.42737
C	-4.50229	-1.49774	0.126259
C	-3.78516	-2.77717	-0.4176
C	-4.14324	-3.04133	-1.89083
C	-2.26896	-2.79145	-0.19246
N	-1.57775	-2.19375	-1.09784
C	-1.69177	-3.52997	1.026774
C	-0.17675	-3.30435	1.104352
C	0.379424	-3.34512	-0.29664
O	-0.18135	-2.2417	-1.04702
C	-2.35419	-3.08287	2.335611
O	-1.86146	-4.94923	0.837623
O	1.755541	-3.18287	-0.30024
C	2.365545	-3.45372	-1.56352
H	4.655404	-1.57987	0.48315
H	2.570513	-0.68101	-0.41916
H	4.500751	3.079491	-1.20567
H	6.626178	2.160977	-0.31013
H	8.163371	-1.58646	1.44917
H	6.460951	-1.64799	1.984888
H	6.908344	-2.23016	0.354133
H	1.447508	1.066677	-1.84797
H	2.228814	2.632425	-2.10049
H	1.781621	3.286601	0.211868
H	1.644307	1.026804	1.375373
H	-0.27258	3.138686	-1.67814
H	-0.21648	5.041995	-0.3391
H	-2.73818	3.034672	1.625986
H	-2.93747	2.088735	-1.255
H	0.809166	4.275232	1.778293
H	-0.65444	5.221646	2.069982
H	-0.67839	3.465183	2.263841
H	-2.16877	6.233377	-0.93973
H	-3.84504	5.500902	-0.61564
H	-4.82998	2.583244	0.514995

H	-1.28914	-0.07465	-1.29475
H	-3.19737	0.509936	1.382642
H	-4.06939	-0.04195	-1.5005
H	-4.42693	-1.47397	1.218625
H	-5.56904	-1.61161	-0.11621
H	-4.19887	-3.61231	0.166475
H	-5.23123	-3.10148	-2.01125
H	-3.76192	-2.25093	-2.54146
H	-3.70684	-3.98538	-2.23324
H	0.267816	-4.08769	1.725086
H	0.036918	-2.32231	1.537406
H	0.084518	-4.2726	-0.8149
H	-3.42582	-3.31396	2.350058
H	-1.88574	-3.61951	3.167402
H	-2.22166	-2.00792	2.489848
H	-2.81016	-5.14882	0.885934
H	2.021102	-2.75483	-2.33228
H	3.441124	-3.33461	-1.41967
H	2.151414	-4.48309	-1.88429

Conf 6

E_{ZPC}= -1764.458563 Hartree

B3LYP/6-31G(d)

Symbol	X	Y	Z
C	4.516103	-1.14595	0.17456
C	3.391345	-0.51059	-0.3391
C	3.458892	0.803883	-0.83708
C	4.700153	1.445684	-0.80414
C	5.84366	0.819949	-0.29763
C	5.75268	-0.48511	0.198555
O	6.798007	-1.19694	0.718115
C	8.071669	-0.57694	0.766151
C	2.21631	1.507875	-1.3382
C	1.447039	2.230001	-0.20388
N	1.028598	1.324027	0.865121
C	0.084079	2.851757	-0.62234
C	-0.92335	1.706282	-0.36186
C	-0.28238	0.963349	0.844836
O	-0.8105	0.148812	1.596911
C	-0.23968	4.186014	0.129221
C	-1.71226	4.52107	-0.02638
C	-2.64227	3.462077	0.516041
C	-2.41101	2.140396	-0.2513
C	0.168087	4.2502	1.621579
C	-2.1518	5.634901	-0.61456
O	-3.99465	3.876813	0.382412

O	-0.8043	0.815211	-1.47627
C	-3.26044	1.022357	0.294007
C	-4.01108	0.198589	-0.446
C	-4.69037	-1.01778	0.116397
C	-4.11673	-2.37535	-0.40782
C	-4.48751	-2.61327	-1.88232
C	-2.61434	-2.55912	-0.16626
N	-1.84992	-2.05263	-1.06842
C	-2.13861	-3.34996	1.063731
C	-0.60773	-3.31012	1.151845
C	-0.04968	-3.42385	-0.24435
O	-0.46904	-2.26418	-1.00309
C	-2.75	-2.81079	2.362886
O	-2.47923	-4.73974	0.886509
O	1.334946	-3.42674	-0.23585
C	1.922457	-3.77913	-1.48955
H	4.458301	-2.15874	0.561944
H	2.446961	-1.04917	-0.35637
H	4.787981	2.459982	-1.18913
H	6.787346	1.353918	-0.29904
H	8.746276	-1.31075	1.211458
H	8.433548	-0.31524	-0.2374
H	8.05916	0.327525	1.389377
H	1.533756	0.80097	-1.81595
H	2.488392	2.264172	-2.08632
H	2.110476	2.995226	0.215466
H	1.691787	0.78993	1.412882
H	0.07195	3.066928	-1.69556
H	0.334576	4.969003	-0.38277
H	-2.41652	3.29488	1.583611
H	-2.70653	2.344632	-1.28859
H	1.248612	4.140178	1.754466
H	-0.11718	5.228355	2.023257
H	-0.31548	3.484358	2.233762
H	-1.45864	6.369163	-1.01888
H	-3.21212	5.843882	-0.70143
H	-4.54036	3.07685	0.462119
H	-1.3185	0.002638	-1.29681
H	-3.16521	0.83327	1.361584
H	-4.08739	0.365185	-1.52087
H	-4.62055	-0.9907	1.209126
H	-5.76132	-1.01417	-0.13373
H	-4.62788	-3.15218	0.179562
H	-5.57378	-2.54919	-2.01551
H	-4.0103	-1.87965	-2.53611
H	-4.15954	-3.60524	-2.21011
H	-0.26663	-4.1392	1.778571
H	-0.27842	-2.36013	1.583761
H	-0.4498	-4.31149	-0.76184

H	-3.84229	-2.90438	2.369113
H	-2.35951	-3.39432	3.203322
H	-2.48381	-1.75979	2.509002
H	-3.44464	-4.82383	0.940709
H	1.672321	-3.05049	-2.26725
H	3.002593	-3.78858	-1.33264
H	1.5902	-4.77813	-1.80586

Conf 7

E_{ZPC}= -1764.458142 Hartree

B3LYP/6-31G(d)

Symbol	X	Y	Z
C	6.146227	-1.74295	-0.62661
C	5.145759	-0.83374	-0.98678
C	5.069998	0.442417	-0.4204
C	6.043018	0.78875	0.533038
C	7.044646	-0.09855	0.903781
C	7.103112	-1.37555	0.325182
O	8.126018	-2.17335	0.752086
C	8.245969	-3.46894	0.18724
C	3.956896	1.399396	-0.78847
C	2.776218	1.349189	0.21045
N	2.138699	0.033059	0.283627
C	1.561736	2.249397	-0.14972
C	0.654861	1.315021	-0.97674
C	0.918818	-0.067	-0.3003
O	0.233974	-1.09211	-0.32962
C	0.85522	2.859752	1.110194
C	-0.54267	3.343953	0.763398
C	-1.45126	2.281728	0.179365
C	-0.79672	1.789397	-1.15377
C	0.786624	1.956232	2.367028
C	-0.93929	4.604626	0.945416
O	-2.73959	2.803941	-0.05918
O	1.254498	1.200277	-2.28495
C	-1.62281	0.73644	-1.84811
C	-2.77168	0.927185	-2.50306
C	-3.63961	-0.22834	-2.91825
C	-4.73568	-0.55241	-1.85423
C	-5.70634	-1.60563	-2.41569
C	-4.16802	-0.85778	-0.45631
N	-4.2843	0.161896	0.322749
C	-3.48339	-2.19101	-0.07158
C	-2.73836	-2.00665	1.26548
C	-3.51668	-1.14775	2.23833
O	-3.80617	0.124876	1.65196

C	-4.48157	-3.35492	0.017826
O	-2.54345	-2.56352	-1.08796
O	-4.67669	-1.81525	2.64502
C	-5.43594	-1.11479	3.625068
H	6.171153	-2.71957	-1.09665
H	4.414185	-1.12779	-1.73597
H	6.019752	1.778031	0.986115
H	7.799752	0.174634	1.634447
H	9.118675	-3.92257	0.6606
H	7.360532	-4.0846	0.394911
H	8.405213	-3.42323	-0.89834
H	3.576605	1.186255	-1.79032
H	4.335792	2.430196	-0.79386
H	3.16973	1.606711	1.200153
H	2.614268	-0.79106	0.628704
H	1.870979	3.07825	-0.79416
H	1.452223	3.736051	1.393593
H	-1.52066	1.433685	0.875892
H	-0.7387	2.689637	-1.78039
H	1.779645	1.670146	2.727286
H	0.292284	2.513875	3.169035
H	0.217039	1.037162	2.204194
H	-0.26059	5.359367	1.336918
H	-1.95459	4.909111	0.717443
H	-3.37275	2.061446	-0.02679
H	0.601973	0.74972	-2.84921
H	-1.29525	-0.28627	-1.69896
H	-3.16452	1.934411	-2.64119
H	-4.16217	-0.00431	-3.85804
H	-3.03415	-1.12457	-3.08021
H	-5.30967	0.369205	-1.70151
H	-6.22032	-1.18654	-3.28842
H	-5.18205	-2.50753	-2.74588
H	-6.46999	-1.89201	-1.68551
H	-1.7843	-1.50073	1.080233
H	-2.5183	-2.98244	1.708513
H	-2.91233	-0.86771	3.114115
H	-3.95151	-4.23466	0.397878
H	-5.30066	-3.11945	0.699947
H	-4.87961	-3.60514	-0.96525
H	-1.69444	-2.1204	-0.90426
H	-5.84388	-0.18075	3.224522
H	-6.25212	-1.77897	3.917391
H	-4.82128	-0.88601	4.5087

Conf 8

E_{ZPC}= -1764.45805 Hartree

B3LYP/6-31G(d)

Symbol	X	Y	Z
C	4.888726	-2.59618	-0.62425
C	3.995394	-1.62142	-1.08177
C	4.29147	-0.25693	-1.00866
C	5.531397	0.111116	-0.45738
C	6.431797	-0.84049	0.001177
C	6.115585	-2.20579	-0.07768
O	7.066137	-3.06168	0.397301
C	6.805259	-4.45531	0.336858
C	3.298061	0.789749	-1.46431
C	2.463771	1.38855	-0.30843
N	1.694432	0.364679	0.389006
C	1.379544	2.400353	-0.79021
C	0.001142	1.721997	-0.50898
C	0.354368	0.444436	0.284154
O	-0.44278	-0.38975	0.726614
C	1.516151	3.808491	-0.14081
C	1.103839	3.674243	1.305438
C	-0.36631	3.322299	1.490014
C	-0.99513	2.652673	0.249372
C	2.875692	4.465249	-0.39792
C	1.911309	3.814366	2.361863
O	-1.13419	4.516453	1.697583
O	-0.52684	1.345167	-1.77279
C	-2.30352	1.987049	0.599728
C	-3.39715	2.013001	-0.16699
C	-4.71118	1.378829	0.194556
C	-5.22466	0.217648	-0.71856
C	-5.28164	0.651712	-2.19004
C	-4.53402	-1.11986	-0.38241
N	-5.05112	-1.62241	0.6885
C	-3.3748	-1.74531	-1.18448
C	-2.91091	-3.05469	-0.5076
C	-3.09455	-2.97078	0.985504
O	-4.49697	-2.77433	1.254928
C	-3.75717	-2.08265	-2.63447
O	-2.27988	-0.82657	-1.26911
O	-2.71031	-4.16069	1.580579
C	-2.73068	-4.1354	3.005374
H	4.620025	-3.64317	-0.70628
H	3.050248	-1.94033	-1.51575
H	5.798633	1.164165	-0.39459
H	7.391576	-0.55449	0.420239
H	7.683763	-4.94279	0.763252
H	5.918931	-4.72628	0.925741
H	6.668142	-4.79605	-0.69793
H	2.604232	0.364576	-2.19942
H	3.821772	1.618764	-1.95811

H	3.143794	1.857594	0.411708
H	2.132566	-0.4579	0.786387
H	1.453028	2.516646	-1.87566
H	0.76311	4.442092	-0.63039
H	-0.46927	2.657048	2.362041
H	-1.20695	3.457265	-0.46468
H	3.067066	4.537317	-1.47466
H	2.894765	5.47949	0.015075
H	3.708362	3.912975	0.051312
H	2.947675	4.126529	2.271289
H	1.55507	3.62601	3.373283
H	-0.7271	4.986527	2.442834
H	-1.32391	0.788611	-1.64985
H	-2.32972	1.443556	1.543729
H	-3.36183	2.560205	-1.11003
H	-4.68084	1.009612	1.224893
H	-5.48694	2.159216	0.160761
H	-6.25817	0.057193	-0.39013
H	-5.84533	1.589953	-2.26152
H	-4.28963	0.830724	-2.6136
H	-5.79677	-0.08511	-2.81373
H	-1.86425	-3.23187	-0.77383
H	-3.50757	-3.90431	-0.85761
H	-2.55283	-2.11256	1.423057
H	-2.96211	-2.69844	-3.06839
H	-4.69376	-2.64845	-2.67229
H	-3.85636	-1.18797	-3.24701
H	-1.83136	-0.75242	-0.39663
H	-3.74972	-4.02265	3.389635
H	-2.31705	-5.0909	3.335968
H	-2.10665	-3.31684	3.393004

Conf 9

E_{ZPC}= -1764.458009 Hartree

B3LYP/6-31G(d)

Symbol	X	Y	Z
C	4.373189	-1.42591	0.210779
C	3.318838	-0.71175	-0.36818
C	3.504413	0.569481	-0.90219
C	4.796618	1.117526	-0.84464
C	5.857203	0.420128	-0.27952
C	5.651632	-0.85957	0.256368
O	6.754382	-1.46402	0.791882
C	6.6016	-2.75839	1.350096
C	2.340705	1.350092	-1.47232
C	1.571508	2.176563	-0.40692

N	1.11886	1.343112	0.700116
C	0.242401	2.769117	-0.96718
C	-0.8452	1.752264	-0.51557
C	-0.18883	0.990204	0.665337
O	-0.73852	0.191959	1.419195
C	-0.08843	4.23158	-0.53664
C	-0.66636	4.212455	0.859474
C	-2.01477	3.510575	0.947418
C	-2.20039	2.406405	-0.116
C	1.055046	5.222401	-0.77404
C	-0.10588	4.749931	1.947111
O	-3.07499	4.450431	0.71064
O	-1.00989	0.856035	-1.60422
C	-3.19462	1.362582	0.327058
C	-3.98002	0.636786	-0.47474
C	-4.81333	-0.50806	0.03234
C	-4.38268	-1.92086	-0.48447
C	-4.7477	-2.10202	-1.96869
C	-2.91479	-2.26393	-0.21562
N	-2.08293	-1.87797	-1.11911
C	-2.55079	-3.06755	1.04031
C	-1.02066	-3.20609	1.153683
C	-0.44221	-3.39618	-0.22574
O	-0.72861	-2.23099	-1.01918
C	-3.10141	-2.42492	2.315327
O	-3.18983	-4.35715	0.962188
O	0.934594	-3.54411	-0.18158
C	1.519006	-3.93311	-1.42568
H	4.180415	-2.41283	0.616578
H	2.334516	-1.17341	-0.39788
H	4.976569	2.108316	-1.25735
H	6.85621	0.843822	-0.24275
H	7.588571	-3.04476	1.718378
H	5.889116	-2.75757	2.185818
H	6.270464	-3.48722	0.598039
H	1.621148	0.673013	-1.94554
H	2.693355	2.044365	-2.2464
H	2.234225	2.954728	-0.01433
H	1.768327	0.822636	1.2769
H	0.276674	2.746026	-2.06055
H	-0.91248	4.541152	-1.19643
H	-2.1208	3.074092	1.952125
H	-2.56931	2.892939	-1.02712
H	1.40974	5.159216	-1.80941
H	0.713172	6.24803	-0.59806
H	1.913665	5.046612	-0.11776
H	0.823249	5.311117	1.912964
H	-0.56432	4.641863	2.928448
H	-2.95067	5.176643	1.342768

H	-1.5649	0.097563	-1.33159
H	-3.18863	1.132757	1.390849
H	-3.99643	0.83995	-1.5456
H	-4.7923	-0.50036	1.127082
H	-5.8654	-0.38037	-0.26164
H	-4.96622	-2.6518	0.088359
H	-5.82161	-1.9366	-2.11343
H	-4.19888	-1.40516	-2.60709
H	-4.51149	-3.11557	-2.31072
H	-0.78416	-4.05516	1.802559
H	-0.5892	-2.29141	1.573197
H	-0.90352	-4.26045	-0.7469
H	-4.19389	-2.3995	2.304574
H	-2.7893	-3.03003	3.17316
H	-2.70617	-1.41267	2.432645
H	-2.88556	-4.79534	0.151034
H	1.379426	-3.16481	-2.19237
H	2.585358	-4.06998	-1.23589
H	1.087836	-4.88151	-1.77809

Conf 10

E_{ZPC}= -1764.457948 Hartree

B3LYP/6-31G(d)

Symbol	X	Y	Z
C	4.245718	-1.86914	0.271909
C	3.245902	-1.09878	-0.31062
C	3.528139	0.161552	-0.87009
C	4.851752	0.608655	-0.82394
C	5.87121	-0.15462	-0.24684
C	5.567794	-1.40293	0.307594
O	6.476863	-2.23632	0.89685
C	7.828185	-1.81306	0.963096
C	2.426913	1.017765	-1.45561
C	1.730397	1.925942	-0.40838
N	1.188935	1.153412	0.703066
C	0.471483	2.640089	-0.98898
C	-0.71733	1.749649	-0.52752
C	-0.14916	0.941947	0.668654
O	-0.78296	0.220283	1.433238
C	0.29079	4.137297	-0.59009
C	-0.28952	4.206539	0.803225
C	-1.70206	3.646393	0.899142
C	-1.99797	2.548816	-0.14661
C	1.531931	4.99887	-0.84086
C	0.321212	4.706787	1.881588
O	-2.66145	4.684505	0.643235

O	-0.96949	0.859453	-1.60417
C	-3.10041	1.624141	0.303847
C	-3.95321	0.976787	-0.49617
C	-4.90915	-0.06608	0.014406
C	-4.62542	-1.52334	-0.48076
C	-4.99169	-1.68444	-1.96683
C	-3.20484	-2.01544	-0.18943
N	-2.32717	-1.73283	-1.08791
C	-2.94032	-2.83179	1.083453
C	-1.43468	-3.12564	1.217809
C	-0.86257	-3.39769	-0.15055
O	-1.01849	-2.22283	-0.96675
C	-3.43493	-2.1144	2.34128
O	-3.70899	-4.04926	1.017109
O	0.490316	-3.68386	-0.0832
C	1.04883	-4.16252	-1.30758
H	4.024831	-2.83995	0.705301
H	2.229155	-1.48472	-0.33048
H	5.104665	1.575706	-1.25456
H	6.885093	0.229537	-0.23975
H	8.369465	-2.61506	1.468634
H	8.25552	-1.663	-0.03755
H	7.934029	-0.88495	1.54091
H	1.654119	0.390216	-1.91335
H	2.83177	1.664612	-2.24494
H	2.461945	2.641503	-0.01835
H	1.777821	0.579577	1.29416
H	0.508499	2.592225	-2.08154
H	-0.49429	4.516922	-1.26069
H	-1.8529	3.239877	1.910693
H	-2.30884	3.05591	-1.06811
H	1.881574	4.878016	-1.87285
H	1.298612	6.057882	-0.68656
H	2.365157	4.746943	-0.17664
H	1.302526	5.170097	1.838395
H	-0.14681	4.666702	2.863522
H	-2.46648	5.404862	1.264064
H	-1.60122	0.166369	-1.32459
H	-3.12732	1.410583	1.370651
H	-3.93835	1.166837	-1.56941
H	-4.89962	-0.04643	1.109082
H	-5.93832	0.169426	-0.29407
H	-5.28902	-2.18104	0.093712
H	-6.04007	-1.40656	-2.1265
H	-4.36436	-1.05952	-2.60738
H	-4.86196	-2.72216	-2.29269
H	-1.29285	-3.9832	1.882902
H	-0.91518	-2.25349	1.627467
H	-1.40445	-4.21837	-0.66501

H	-4.51895	-1.97691	2.318071
H	-3.19469	-2.73325	3.212274
H	-2.93862	-1.14627	2.446463
H	-3.42955	-4.53927	0.226773
H	0.998279	-3.40421	-2.09519
H	2.092657	-4.40275	-1.09666
H	0.527471	-5.07077	-1.64346

Conf 11

E_{ZPC}= -1764.457757 Hartree

B3LYP/6-31G(d)

Symbol	X	Y	Z
C	4.362663	-1.38816	0.202355
C	3.306469	-0.67885	-0.379
C	3.485741	0.607108	-0.90366
C	4.773055	1.165146	-0.83451
C	5.835466	0.472413	-0.26687
C	5.636308	-0.81218	0.259683
O	6.740354	-1.41152	0.798641
C	6.594167	-2.71179	1.344808
C	2.318595	1.381466	-1.47522
C	1.543369	2.20193	-0.40952
N	1.100203	1.365974	0.699736
C	0.207603	2.781812	-0.96765
C	-0.86786	1.752643	-0.51558
C	-0.2046	1.003729	0.669672
O	-0.7492	0.208723	1.430893
C	-0.13943	4.240094	-0.53524
C	-0.72029	4.213584	0.859616
C	-2.06066	3.496063	0.945211
C	-2.23164	2.390224	-0.11871
C	0.993735	5.243562	-0.76868
C	-0.169	4.757783	1.948644
O	-3.13185	4.423465	0.708238
O	-1.01911	0.852272	-1.6024
C	-3.21135	1.333037	0.324277
C	-3.97898	0.589383	-0.47812
C	-4.79573	-0.56545	0.032336
C	-4.34501	-1.97184	-0.48481
C	-4.70876	-2.16367	-1.96786
C	-2.87244	-2.30345	-0.2165
N	-2.05231	-1.90108	-1.12185
C	-2.48548	-3.10344	1.03759
C	-0.95821	-3.18998	1.151776
C	-0.38781	-3.39557	-0.22876
O	-0.69847	-2.23673	-1.03596

C	-3.06784	-2.48752	2.315701
O	-2.94026	-4.46346	0.884723
O	0.994228	-3.50575	-0.19176
C	1.571767	-3.93932	-1.42349
H	4.174111	-2.37898	0.600518
H	2.326628	-1.14912	-0.41854
H	4.94793	2.159874	-1.24
H	6.830962	0.903491	-0.22121
H	7.580991	-2.99361	1.717048
H	5.876311	-2.72379	2.175836
H	6.273469	-3.43668	0.584501
H	1.604201	0.700137	-1.94993
H	2.667878	2.079075	-2.24789
H	2.199817	2.986272	-0.01858
H	1.75535	0.852704	1.276557
H	0.241087	2.760249	-2.06106
H	-0.9655	4.5418	-1.19625
H	-2.16247	3.057718	1.94954
H	-2.60592	2.871878	-1.0302
H	1.351564	5.185556	-1.80325
H	0.64046	6.265236	-0.59216
H	1.852521	5.075903	-0.11054
H	0.753606	5.329747	1.916925
H	-0.62852	4.644166	2.928857
H	-3.01399	5.152575	1.33828
H	-1.55274	0.079293	-1.32757
H	-3.20729	1.10882	1.389205
H	-3.99321	0.785067	-1.55017
H	-4.77111	-0.55597	1.127245
H	-5.85037	-0.45286	-0.25908
H	-4.94073	-2.69903	0.087488
H	-5.78388	-2.00863	-2.11561
H	-4.16424	-1.46215	-2.60418
H	-4.45707	-3.17462	-2.30504
H	-0.69895	-4.02459	1.809825
H	-0.55654	-2.25677	1.558306
H	-0.84405	-4.26943	-0.72289
H	-4.16381	-2.488	2.305448
H	-2.7406	-3.08456	3.173553
H	-2.71577	-1.45988	2.445509
H	-3.91093	-4.45861	0.893197
H	1.399236	-3.21388	-2.22503
H	2.644717	-4.03611	-1.24536
H	1.162891	-4.91513	-1.72255

Conf 12

E_{ZPC}= -1764.457656 Hartree

B3LYP/6-31G(d)

Symbol	X	Y	Z
C	4.233639	-1.85568	0.269767
C	3.233046	-1.08735	-0.31417
C	3.514175	0.173605	-0.87288
C	4.836823	0.623303	-0.82504
C	5.856915	-0.13819	-0.24661
C	5.554771	-1.38699	0.307399
O	6.464722	-2.21868	0.898147
C	7.815247	-1.79352	0.964709
C	2.411818	1.028152	-1.45872
C	1.71723	1.93759	-0.41136
N	1.180478	1.16675	0.703773
C	0.455266	2.649163	-0.98863
C	-0.72922	1.754752	-0.52466
C	-0.1574	0.955098	0.675256
O	-0.79006	0.241007	1.448069
C	0.270165	4.145656	-0.58878
C	-0.31116	4.21342	0.804281
C	-1.72168	3.648116	0.900746
C	-2.01338	2.548653	-0.14406
C	1.508914	5.010927	-0.83863
C	0.296912	4.717095	1.882566
O	-2.68538	4.682391	0.644813
O	-0.97748	0.861732	-1.59925
C	-3.10885	1.616842	0.307919
C	-3.95392	0.959714	-0.49209
C	-4.89987	-0.09103	0.020167
C	-4.60636	-1.54369	-0.48293
C	-4.97151	-1.70431	-1.96921
C	-3.18448	-2.03841	-0.19234
N	-2.31208	-1.73977	-1.08898
C	-2.90593	-2.86436	1.073704
C	-1.39935	-3.11295	1.213034
C	-0.83126	-3.39596	-0.1548
O	-1.00592	-2.22235	-0.98279
C	-3.43813	-2.17851	2.33789
O	-3.50232	-4.16882	0.923854
O	0.530257	-3.64708	-0.0896
C	1.082372	-4.1684	-1.29855
H	4.013412	-2.82696	0.702391
H	2.21752	-1.47642	-0.33614
H	5.088587	1.590841	-1.25534
H	6.870153	0.247677	-0.23825
H	8.35757	-2.5945	1.470802
H	8.242846	-1.64321	-0.03582
H	7.919736	-0.86495	1.542119
H	1.63866	0.399308	-1.9139
H	2.815032	1.674084	-2.2497

H	2.449097	2.654845	-0.02501
H	1.772127	0.595441	1.294565
H	0.489939	2.601856	-2.08127
H	-0.51578	4.523192	-1.25963
H	-1.87046	3.241678	1.912627
H	-2.32796	3.053271	-1.06562
H	1.859705	4.890912	-1.87031
H	1.2725	6.069309	-0.68462
H	2.342309	4.761273	-0.17379
H	1.276509	5.183996	1.839564
H	-0.17149	4.675928	2.864281
H	-2.48983	5.405643	1.262099
H	-1.59371	0.155953	-1.3164
H	-3.13426	1.404638	1.374945
H	-3.94	1.146949	-1.56561
H	-4.88323	-0.07563	1.115098
H	-5.93268	0.137622	-0.2817
H	-5.28831	-2.19346	0.086263
H	-6.01964	-1.42702	-2.13202
H	-4.34166	-1.07601	-2.60328
H	-4.83425	-2.74046	-2.29559
H	-1.23997	-3.96236	1.883716
H	-0.90506	-2.2237	1.615619
H	-1.36793	-4.22411	-0.64687
H	-4.52795	-2.06369	2.312107
H	-3.18722	-2.79821	3.205259
H	-2.98185	-1.19229	2.464865
H	-4.4666	-4.05798	0.91004
H	1.002435	-3.44826	-2.11946
H	2.135242	-4.37357	-1.09454
H	0.578298	-5.10257	-1.58491

Table S8. Atomic coordinates of the lowest energy conformer of (3*S*,4*S*,5*S*,7*S*,8*S*,9*S*,13*E*,16*S*,18*R*,20*S*)-**1**.

Conf 1

E_{ZPC}= -1764.465306 Hartree

B3LYP/6-31G(d)

Symbol	X	Y	Z
C	-6.68702	-0.67574	-0.3137
C	-5.69138	-0.02363	0.400056
C	-4.52956	-0.69554	0.819113
C	-4.41153	-2.04981	0.492251
C	-5.40328	-2.725	-0.22764
C	-6.54967	-2.03544	-0.6352
O	-7.58271	-2.58508	-1.33608
C	-7.50164	-3.958	-1.68644
C	-3.42933	0.032747	1.561571

C	-2.49557	0.841722	0.632839
N	-1.83407	-0.02635	-0.33714
C	-1.30601	1.525664	1.370695
C	-0.01372	1.126901	0.600692
C	-0.48538	0.016464	-0.36832
O	0.231176	-0.72194	-1.04589
C	-1.45874	3.055992	1.537453
C	-1.46116	3.738922	0.178087
C	-0.42133	3.230363	-0.81749
C	0.639493	2.331256	-0.14604
C	-2.63463	3.435427	2.44436
C	-2.28738	4.727693	-0.17193
O	0.189698	4.355895	-1.44004
O	0.898917	0.579783	1.542134
C	1.680988	1.878315	-1.13617
C	3.000479	2.062542	-1.00564
C	4.023169	1.499278	-1.95004
C	5.072781	0.593054	-1.24317
C	6.084374	0.072893	-2.27666
C	4.439292	-0.46894	-0.33089
N	4.502068	-0.10238	0.902034
C	3.744277	-1.74066	-0.85928
C	2.882071	-2.33969	0.270798
C	3.558596	-2.2426	1.622085
O	3.90149	-0.88471	1.914968
C	4.757111	-2.77495	-1.37465
O	2.91896	-1.40838	-1.97709
O	4.674999	-3.08762	1.662343
C	5.33468	-3.11643	2.922583
H	-7.58611	-0.15685	-0.6313
H	-5.8201	1.028778	0.644594
H	-3.53178	-2.60295	0.814677
H	-5.2725	-3.77698	-0.45394
H	-8.4202	-4.18224	-2.23147
H	-6.63783	-4.159	-2.33386
H	-7.44223	-4.59873	-0.79671
H	-2.81707	-0.68283	2.124007
H	-3.8619	0.727278	2.291977
H	-3.0943	1.591254	0.09824
H	-2.34439	-0.71359	-0.87937
H	-1.20809	1.076554	2.365185
H	-0.5447	3.380872	2.059114
H	-0.93787	2.625175	-1.5857
H	1.133124	2.927377	0.632327
H	-2.56999	2.89942	3.397812
H	-2.62858	4.507294	2.666389
H	-3.60142	3.199637	1.984653
H	-3.0422	5.123038	0.500468
H	-2.21046	5.195941	-1.14829

H	0.940549	4.014158	-1.95579
H	1.784823	0.541222	1.140086
H	1.314625	1.319265	-1.99634
H	3.381878	2.605819	-0.13896
H	3.528387	0.934348	-2.74493
H	4.583928	2.319344	-2.42399
H	5.61957	1.234758	-0.54191
H	6.836168	-0.57764	-1.81807
H	5.593418	-0.47715	-3.08496
H	6.61006	0.923186	-2.7274
H	2.632782	-3.3802	0.042061
H	1.944292	-1.78073	0.3454
H	2.867921	-2.48901	2.442316
H	5.49033	-3.0245	-0.6055
H	4.211107	-3.68451	-1.64744
H	5.267155	-2.41243	-2.26741
H	2.059901	-1.07688	-1.64615
H	4.635317	-3.3901	3.72727
H	6.112728	-3.8796	2.84848
H	5.787527	-2.14821	3.161478

Conf 2

E_{ZPC}= -1764.460682 Hartree

B3LYP/6-31G(d)

Symbol	X	Y	Z
C	4.685807	-1.6837	-0.54693
C	3.620699	-0.88142	-0.94002
C	3.742348	0.516778	-1.0038
C	4.977412	1.075633	-0.65981
C	6.059925	0.286776	-0.26121
C	5.915139	-1.10437	-0.20164
O	6.899211	-1.97497	0.170495
C	8.167263	-1.44404	0.519705
C	2.56416	1.381896	-1.39455
C	1.83771	1.989485	-0.17216
N	1.298294	0.98202	0.744083
C	0.560749	2.807136	-0.50495
C	-0.57594	1.764413	-0.39583
C	-0.04577	0.821023	0.71333
O	-0.67672	0.005926	1.389077
C	0.380809	4.054101	0.426205
C	-1.03362	4.590499	0.309661
C	-2.10272	3.587646	0.674392
C	-1.99713	2.374064	-0.27896
C	0.751526	3.857626	1.917303
C	-1.31509	5.825439	-0.10895

O	-3.38674	4.183076	0.570988
O	-0.52312	0.97443	-1.59487
C	-3.04614	1.331858	0.012087
C	-3.85133	0.824624	-0.92893
C	-4.78052	-0.34182	-0.74957
C	-4.1646	-1.62099	-1.39386
C	-5.24612	-2.64856	-1.78892
C	-3.00935	-2.19898	-0.57439
N	-1.88847	-2.1449	-1.2119
C	-3.13298	-2.70216	0.872445
C	-2.01294	-3.72491	1.109791
C	-0.66812	-3.18463	0.688355
O	-0.70727	-2.64085	-0.6602
C	-4.47126	-3.35021	1.232835
O	-3.01845	-1.58411	1.762341
O	0.245283	-4.23332	0.699842
C	1.596054	-3.85417	0.474067
H	4.594536	-2.76506	-0.50979
H	2.675824	-1.34481	-1.2139
H	5.110011	2.154871	-0.71032
H	7.001299	0.762236	-0.00978
H	8.79027	-2.30197	0.778902
H	8.624931	-0.9046	-0.32023
H	8.099419	-0.77113	1.385026
H	1.840618	0.810419	-1.98072
H	2.903534	2.220199	-2.01781
H	2.562111	2.607041	0.370919
H	1.883796	0.327762	1.247903
H	0.589845	3.165833	-1.53868
H	1.06214	4.820964	0.035749
H	-1.94226	3.240461	1.709806
H	-2.18382	2.777044	-1.28319
H	1.802271	3.581317	2.048056
H	0.591574	4.803523	2.445286
H	0.147304	3.095162	2.416668
H	-0.52562	6.517804	-0.39266
H	-2.33874	6.176448	-0.1735
H	-4.02972	3.456555	0.514292
H	-1.10944	0.20015	-1.48633
H	-3.09685	0.941775	1.026401
H	-3.76944	1.210552	-1.94766
H	-4.99065	-0.50816	0.310705
H	-5.73702	-0.13847	-1.25046
H	-3.68858	-1.31063	-2.33201
H	-4.79289	-3.5703	-2.17001
H	-5.91349	-2.91005	-0.96438
H	-5.86481	-2.22656	-2.5895
H	-2.2075	-4.63753	0.533888
H	-1.97836	-3.98945	2.17104

H	-0.326	-2.36527	1.336094
H	-4.68224	-4.2217	0.606941
H	-4.41953	-3.67557	2.276974
H	-5.29586	-2.6394	1.149876
H	-2.21023	-1.06975	1.559048
H	1.893216	-3.02148	1.12846
H	2.207797	-4.72893	0.708209
H	1.760311	-3.56488	-0.57018

Table S9. Atomic coordinates of the lowest energy conformer of (3*S*,4*S*,5*S*,7*S*,8*S*,9*S*,13*E*,16*R*,18*R*,20*S*)-**1**.

Conf 1

E_{ZPC}= -1764.468841 Hartree

B3LYP/6-31G(d)

Symbol	X	Y	Z
C	5.802105	-2.51462	-0.49516
C	4.750805	-1.79022	-1.06821
C	4.719654	-0.39286	-1.04654
C	5.791524	0.272665	-0.42535
C	6.843234	-0.42744	0.14854
C	6.856056	-1.83109	0.119185
O	7.933417	-2.42543	0.708155
C	8.004637	-3.8427	0.702999
C	3.557098	0.378134	-1.63587
C	2.554833	0.881095	-0.57266
N	1.977242	-0.23621	0.172337
C	1.302058	1.593016	-1.16337
C	0.065884	0.982394	-0.44024
C	0.630646	-0.30161	0.209652
O	-0.01948	-1.23489	0.691245
C	1.335796	3.13557	-1.09933
C	1.389258	3.608028	0.345252
C	0.531015	2.816453	1.326392
C	-0.54854	1.962331	0.62126
C	2.409554	3.747711	-2.00652
C	2.112887	4.644026	0.776945
O	-0.06194	3.731852	2.241905
O	-0.89225	0.666227	-1.4293
C	-1.40326	1.26499	1.648599
C	-2.739	1.312428	1.706975
C	-3.56359	0.527794	2.698934
C	-3.73114	-0.96121	2.296187
C	-4.55716	-1.71858	3.352381
C	-4.33916	-1.1204	0.901431
N	-5.38496	-0.40407	0.691771
C	-3.67813	-2.07577	-0.10296
C	-4.57554	-2.21878	-1.33821

C	-5.19592	-0.88142	-1.66448
O	-6.01628	-0.453	-0.55371
C	-3.39374	-3.4583	0.500894
O	-2.44796	-1.49704	-0.56899
O	-6.01916	-0.99789	-2.77045
C	-6.53841	0.239489	-3.25054
H	5.788436	-3.59755	-0.54177
H	3.944521	-2.33557	-1.55439
H	5.803935	1.360299	-0.39724
H	7.672453	0.088239	0.622837
H	8.93114	-4.09751	1.220506
H	7.156258	-4.29153	1.236486
H	8.039093	-4.24149	-0.31951
H	3.012957	-0.2512	-2.35061
H	3.924691	1.249268	-2.19115
H	3.085735	1.547343	0.120575
H	2.544992	-0.99552	0.530245
H	1.197098	1.29125	-2.21131
H	0.362498	3.454581	-1.50528
H	1.189609	2.122958	1.883955
H	-1.18508	2.639964	0.039318
H	2.333045	3.339368	-3.02035
H	2.290773	4.83356	-2.07797
H	3.421276	3.551993	-1.63296
H	2.742181	5.231323	0.11582
H	2.072078	4.953728	1.816216
H	-0.74397	3.237487	2.728576
H	-1.5981	0.100326	-1.04973
H	-0.86804	0.659709	2.383543
H	-3.28242	1.891677	0.961196
H	-3.08432	0.549979	3.686568
H	-4.55541	0.977807	2.804266
H	-2.72244	-1.39011	2.264751
H	-4.09966	-1.6225	4.344275
H	-5.57279	-1.31196	3.397757
H	-4.63231	-2.78602	3.119003
H	-5.38783	-2.92883	-1.14791
H	-3.97521	-2.58346	-2.17646
H	-4.42397	-0.1071	-1.81637
H	-4.31397	-3.93636	0.852901
H	-2.94393	-4.09368	-0.26922
H	-2.69302	-3.39987	1.339972
H	-1.72184	-1.58597	0.089801
H	-7.0663	0.011882	-4.17931
H	-7.23284	0.688561	-2.53294
H	-5.72678	0.951484	-3.46078

Conf 2

E_{ZPC}= -1764.468688 Hartree

B3LYP/6-31G(d)

Symbol	X	Y	Z
C	5.656246	-2.74429	-0.52626
C	4.624524	-2.00305	-1.09107
C	4.658454	-0.59849	-1.11465
C	5.773037	0.03167	-0.55096
C	6.820108	-0.69487	0.020498
C	6.76363	-2.09408	0.036059
O	7.724865	-2.9063	0.562897
C	8.871413	-2.30344	1.142352
C	3.511798	0.204426	-1.69273
C	2.540949	0.745912	-0.6192
N	1.938289	-0.3457	0.143594
C	1.303634	1.493507	-1.19845
C	0.057606	0.926234	-0.45664
C	0.590882	-0.36762	0.200084
O	-0.08148	-1.27436	0.7012
C	1.387415	3.034725	-1.14343
C	1.471697	3.512332	0.297832
C	0.592971	2.759292	1.291257
C	-0.5172	1.933381	0.600614
C	2.469857	3.607929	-2.06566
C	2.237048	4.52297	0.717647
O	0.033445	3.703017	2.199304
O	-0.91979	0.628943	-1.43282
C	-1.38572	1.271465	1.639887
C	-2.71957	1.353819	1.702719
C	-3.55959	0.602778	2.707599
C	-3.76931	-0.88569	2.323034
C	-4.60668	-1.60918	3.39398
C	-4.39233	-1.04448	0.934906
N	-5.42071	-0.30348	0.724977
C	-3.76452	-2.02853	-0.06301
C	-4.6735	-2.1608	-1.29105
C	-5.26201	-0.81149	-1.62698
O	-6.06305	-0.3507	-0.51507
C	-3.51331	-3.41153	0.554284
O	-2.52227	-1.48814	-0.54288
O	-6.09601	-0.91842	-2.72583
C	-6.58724	0.326719	-3.21521
H	5.630403	-3.82947	-0.51522
H	3.779927	-2.52557	-1.53532
H	5.835972	1.117996	-0.55941
H	7.667397	-0.16532	0.441104
H	9.495208	-3.12625	1.495972
H	9.433321	-1.71337	0.40623

H	8.604298	-1.66049	1.991496
H	2.937966	-0.41182	-2.39569
H	3.897775	1.060353	-2.25873
H	3.103088	1.399989	0.060857
H	2.485839	-1.11845	0.504249
H	1.176108	1.189534	-2.24317
H	0.420458	3.382952	-1.54044
H	1.231407	2.051323	1.854094
H	-1.13634	2.625906	0.017477
H	2.36834	3.198075	-3.07665
H	2.385054	4.696754	-2.14068
H	3.479383	3.381515	-1.70337
H	2.881092	5.084061	0.048021
H	2.216986	4.839373	1.755591
H	-0.66378	3.2359	2.691448
H	-1.63571	0.083442	-1.04218
H	-0.86308	0.660835	2.379444
H	-3.25091	1.938032	0.952094
H	-3.07437	0.623425	3.69235
H	-4.53842	1.080409	2.812778
H	-2.7726	-1.34154	2.288983
H	-4.13841	-1.51516	4.381028
H	-5.61058	-1.17483	3.443261
H	-4.71257	-2.67656	3.172709
H	-5.50222	-2.8481	-1.08851
H	-4.08806	-2.54901	-2.12925
H	-4.47188	-0.05861	-1.79258
H	-4.44347	-3.86036	0.918477
H	-3.08697	-4.06746	-0.21194
H	-2.80458	-3.36317	1.387303
H	-1.79504	-1.58982	0.112747
H	-7.12605	0.103283	-4.13871
H	-7.26612	0.799881	-2.49819
H	-5.75944	1.016262	-3.43696

Conf 3

E_{ZPC}= -1764.466063 Hartree

B3LYP/6-31G(d)

Symbol	X	Y	Z
C	-5.02222	-2.8056	-0.3646
C	-4.00611	-2.09283	0.281263
C	-4.24194	-0.85176	0.880303
C	-5.54828	-0.33593	0.815239
C	-6.57022	-1.0277	0.179879
C	-6.31368	-2.27151	-0.41794
O	-7.38299	-2.87353	-1.01511

C	-7.18513	-4.13871	-1.62629
C	-3.12582	-0.06998	1.538286
C	-2.52626	1.030804	0.632992
N	-1.96888	0.476931	-0.59517
C	-1.3187	1.770351	1.286698
C	-0.06384	1.371731	0.445594
C	-0.63208	0.566713	-0.74401
O	0.016078	0.058057	-1.66383
C	-1.52392	3.308793	1.404874
C	-1.43973	3.87832	0.009997
C	-0.06645	3.726847	-0.62931
C	0.785624	2.60268	-0.00152
C	-2.76348	3.694048	2.217703
C	-2.44609	4.449544	-0.66005
O	0.699421	4.925533	-0.43987
O	0.707683	0.500746	1.25941
C	1.94637	2.254128	-0.90011
C	3.17806	1.940934	-0.4886
C	4.300311	1.571446	-1.43137
C	4.223812	0.114206	-1.95508
C	5.337711	-0.15498	-2.98452
C	4.264123	-0.91419	-0.82356
N	5.231643	-0.75547	0.006909
C	3.193423	-2.01273	-0.76737
C	3.548188	-3.02219	0.331859
C	4.143317	-2.28989	1.510676
O	5.356864	-1.62827	1.092402
C	3.026771	-2.73772	-2.10999
O	1.937814	-1.42893	-0.37775
O	4.479252	-3.19024	2.507086
C	4.925386	-2.58584	3.718275
H	-4.79542	-3.76778	-0.80957
H	-3.00929	-2.52624	0.324735
H	-5.76961	0.623103	1.279449
H	-7.57972	-0.6306	0.137086
H	-8.15492	-4.42886	-2.03444
H	-6.4509	-4.08338	-2.44094
H	-6.85822	-4.89232	-0.89762
H	-2.31279	-0.74208	1.837453
H	-3.49272	0.416857	2.45151
H	-3.31898	1.738097	0.363698
H	-2.5172	-0.10324	-1.21897
H	-1.15547	1.371153	2.2922
H	-0.65335	3.688484	1.958461
H	-0.1945	3.53369	-1.70653
H	1.191224	3.002412	0.935443
H	-2.71538	3.252715	3.219698
H	-2.82348	4.78169	2.332613
H	-3.69811	3.361757	1.75262

H	-3.41964	4.631505	-0.21421
H	-2.32679	4.767538	-1.6945
H	0.159996	5.659885	-0.77429
H	1.341553	-0.0036	0.705522
H	1.734686	2.25898	-1.96997
H	3.396955	1.922259	0.57833
H	4.277665	2.23496	-2.30609
H	5.267566	1.715009	-0.93961
H	3.254506	0.014531	-2.45821
H	5.282324	0.563751	-3.81071
H	6.319785	-0.06273	-2.50945
H	5.263076	-1.16219	-3.40938
H	4.293086	-3.7408	-0.02704
H	2.644973	-3.56472	0.625138
H	3.459357	-1.50913	1.886734
H	2.695285	-2.05831	-2.90175
H	3.965127	-3.20389	-2.42799
H	2.269297	-3.52086	-1.99882
H	1.494465	-0.97252	-1.12621
H	4.185014	-1.8642	4.09361
H	5.03822	-3.39553	4.442792
H	5.885044	-2.07658	3.582458

Conf 4

E_{ZPC}= -1764.46597 Hartree

B3LYP/6-31G(d)

Symbol	X	Y	Z
C	-5.09979	-2.22487	-0.01828
C	-4.10383	-1.48876	0.632774
C	-4.20308	-0.1051	0.806941
C	-5.35109	0.532752	0.304787
C	-6.35087	-0.17941	-0.34287
C	-6.23179	-1.56793	-0.51147
O	-7.26682	-2.17655	-1.159
C	-7.20566	-3.58124	-1.35115
C	-3.09712	0.685971	1.472035
C	-2.13448	1.358665	0.467519
N	-1.47962	0.375719	-0.38871
C	-0.94498	2.104389	1.145866
C	0.360443	1.443047	0.607488
C	-0.13375	0.334112	-0.3468
O	0.567339	-0.48223	-0.95031
C	-0.98388	3.642467	0.943188
C	-0.72475	3.971948	-0.51435
C	0.536008	3.34931	-1.10922
C	1.298005	2.471251	-0.0951

C	-2.24182	4.280902	1.540496
C	-1.50705	4.742933	-1.27361
O	1.372017	4.410748	-1.57279
O	1.010344	0.837583	1.71445
C	2.494565	1.811769	-0.72807
C	3.722974	1.817928	-0.19694
C	4.882732	1.031097	-0.7379
C	5.254977	-0.19433	0.15994
C	5.690263	0.237641	1.568665
C	4.152549	-1.25178	0.167222
N	3.455814	-1.30896	1.247173
C	3.89955	-2.13113	-1.0652
C	3.212865	-3.42235	-0.59465
C	2.040972	-3.11472	0.306549
O	2.432711	-2.24368	1.402375
C	5.174138	-2.48599	-1.83446
O	3.082629	-1.41443	-2.00043
O	1.578006	-4.30065	0.866063
C	0.374057	-4.16645	1.612547
H	-4.98384	-3.29735	-0.1255
H	-3.23513	-2.01498	1.023102
H	-5.46564	1.60745	0.430844
H	-7.23919	0.314334	-0.72444
H	-8.12045	-3.85068	-1.88191
H	-6.33668	-3.86832	-1.95795
H	-7.17022	-4.11829	-0.39404
H	-2.50655	0.03489	2.127962
H	-3.52384	1.475867	2.103185
H	-2.70823	2.046552	-0.16609
H	-2.00405	-0.31189	-0.91658
H	-0.96602	1.899361	2.22084
H	-0.13289	4.038084	1.518037
H	0.244389	2.714385	-1.9645
H	1.656084	3.126718	0.708457
H	-2.34202	4.01038	2.59767
H	-2.18916	5.37274	1.478781
H	-3.15544	3.96588	1.023429
H	-2.40139	5.225738	-0.89166
H	-1.2541	4.942537	-2.31125
H	2.224463	4.005508	-1.81031
H	1.787935	0.336529	1.402157
H	2.318294	1.248875	-1.64305
H	3.891194	2.372058	0.727296
H	4.650271	0.67937	-1.7474
H	5.775941	1.668555	-0.80273
H	6.127791	-0.65612	-0.31829
H	6.506048	0.967151	1.496962
H	4.87007	0.6851	2.134128
H	6.047001	-0.62152	2.144908

H	3.919272	-4.04655	-0.0345
H	2.864902	-3.98924	-1.4636
H	1.231026	-2.59948	-0.22793
H	5.90543	-2.98572	-1.19061
H	4.913561	-3.16163	-2.65541
H	5.632951	-1.59501	-2.27083
H	2.267609	-1.09893	-1.55948
H	0.063279	-5.17845	1.883082
H	0.528911	-3.57524	2.521412
H	-0.41583	-3.69499	1.007928

Conf 5

E_{ZPC}= -1764.465857 Hartree

B3LYP/6-31G(d)

Symbol	X	Y	Z
C	-4.65615	-2.31517	-0.32535
C	-3.66092	-1.50723	0.235045
C	-3.96369	-0.29758	0.869064
C	-5.31463	0.08578	0.927698
C	-6.3171	-0.70268	0.378641
C	-5.99393	-1.91234	-0.25485
O	-7.04881	-2.61655	-0.7609
C	-6.78285	-3.8533	-1.40245
C	-2.86931	0.58248	1.431678
C	-2.42091	1.689944	0.446967
N	-1.95621	1.153459	-0.83431
C	-1.18072	2.50551	0.905935
C	0.012594	1.733627	0.285585
C	-0.61222	1.111795	-0.98989
O	-0.02187	0.570186	-1.92976
C	-1.26656	4.01585	0.513813
C	0.095762	4.658501	0.683372
C	1.165165	4.046084	-0.18627
C	1.335034	2.542864	0.16041
C	-1.84306	4.324434	-0.88932
C	0.338245	5.652597	1.53922
O	2.397163	4.725886	-0.00446
O	0.255085	0.614464	1.152712
C	2.34404	1.919009	-0.7716
C	3.460949	1.29129	-0.38472
C	4.425839	0.627799	-1.33944
C	3.91371	-0.73836	-1.86774
C	4.935995	-1.36328	-2.8355
C	3.561731	-1.70268	-0.73351
N	4.46472	-1.81039	0.174181
C	2.213567	-2.43689	-0.76185

C	2.162723	-3.46563	0.37372
C	2.855805	-2.90469	1.592912
O	4.240871	-2.64463	1.272107
C	1.951703	-3.12534	-2.10869
O	1.152658	-1.49894	-0.49477
O	2.829973	-3.83122	2.618952
C	3.328884	-3.3464	3.863788
H	-4.37727	-3.24859	-0.80121
H	-2.62517	-1.83623	0.182328
H	-5.58628	1.016368	1.422274
H	-7.36075	-0.40829	0.431892
H	-7.75219	-4.23867	-1.72386
H	-6.13597	-3.72265	-2.28031
H	-6.31635	-4.57254	-0.71618
H	-1.9926	-0.0125	1.699138
H	-3.21844	1.081266	2.345599
H	-3.27677	2.349689	0.265251
H	-2.55039	0.590033	-1.43078
H	-1.07208	2.455482	1.993805
H	-1.94798	4.479088	1.239214
H	0.856769	4.128498	-1.24315
H	1.73974	2.526025	1.180272
H	-2.87693	3.980027	-0.99116
H	-1.83925	5.408607	-1.04358
H	-1.26765	3.871948	-1.70169
H	-0.45068	6.060202	2.167379
H	1.3275	6.08692	1.629368
H	3.093055	4.134995	-0.33903
H	0.787136	-0.0578	0.673774
H	2.121928	2.000406	-1.83596
H	3.667657	1.185189	0.679949
H	4.596543	1.27368	-2.21069
H	5.39278	0.475329	-0.85033
H	2.990291	-0.53178	-2.4228
H	5.160426	-0.67414	-3.65819
H	5.867927	-1.58648	-2.30591
H	4.564695	-2.29685	-3.27163
H	2.683419	-4.38522	0.086054
H	1.11876	-3.70548	0.594931
H	2.409513	-1.94391	1.904327
H	1.904133	-2.40476	-2.93147
H	2.732201	-3.85807	-2.33839
H	0.989276	-3.6455	-2.06001
H	0.981214	-0.91982	-1.26815
H	2.798413	-2.43316	4.170242
H	3.142541	-4.13424	4.59687
H	4.402469	-3.1383	3.811522

Conf 6

E_{ZPC}= -1764.465825 Hartree

B3LYP/6-31G(d)

Symbol	X	Y	Z
C	-4.94188	-2.91775	-0.55244
C	-3.93671	-2.22941	0.117109
C	-4.20005	-1.03093	0.802238
C	-5.51525	-0.55523	0.791021
C	-6.53986	-1.23261	0.125474
C	-6.25365	-2.42275	-0.5542
O	-7.17044	-3.1701	-1.23525
C	-8.5159	-2.72115	-1.26263
C	-3.09449	-0.26103	1.491579
C	-2.50693	0.880686	0.630235
N	-1.9273	0.38224	-0.61139
C	-1.32067	1.621164	1.320974
C	-0.05131	1.28568	0.474469
C	-0.59244	0.513932	-0.74927
O	0.073136	0.059599	-1.6846
C	-1.5636	3.148412	1.49789
C	-1.48362	3.774217	0.126833
C	-0.10333	3.679089	-0.50908
C	0.771308	2.553407	0.082861
C	-2.81763	3.471598	2.315566
C	-2.49863	4.34908	-0.52692
O	0.631868	4.888196	-0.27149
O	0.735667	0.401859	1.258958
C	1.947661	2.269947	-0.81803
C	3.186166	1.983265	-0.40777
C	4.327386	1.686426	-1.35343
C	4.300218	0.250322	-1.93689
C	5.434211	0.055744	-2.96086
C	4.35629	-0.82149	-0.84705
N	5.309427	-0.66917	0.000999
C	3.315211	-1.94927	-0.84682
C	3.684908	-2.99116	0.21656
C	4.24684	-2.29042	1.430331
O	5.446286	-1.57984	1.053429
C	3.181318	-2.62536	-2.21841
O	2.040359	-1.41395	-0.4499
O	4.596647	-3.22054	2.394047
C	5.010984	-2.65358	3.634225
H	-4.73684	-3.84668	-1.07546
H	-2.9276	-2.63554	0.118984
H	-5.75667	0.364151	1.321006
H	-7.5465	-0.83087	0.14966
H	-9.06532	-3.458	-1.85124
H	-8.94572	-2.67033	-0.25336

H	-8.60398	-1.73667	-1.74129
H	-2.27394	-0.93368	1.768574
H	-3.46997	0.187023	2.420805
H	-3.31127	1.581476	0.378612
H	-2.45773	-0.18317	-1.26363
H	-1.15424	1.186441	2.311118
H	-0.7063	3.526582	2.072668
H	-0.22135	3.522046	-1.59328
H	1.158976	2.926163	1.038385
H	-2.76468	2.993743	3.300432
H	-2.90585	4.552231	2.471207
H	-3.74048	3.133733	1.831275
H	-3.47854	4.491813	-0.0807
H	-2.38037	4.710594	-1.54711
H	0.075663	5.620967	-0.5809
H	1.386307	-0.06275	0.689853
H	1.742892	2.304901	-1.88867
H	3.398148	1.934809	0.659623
H	4.29255	2.384393	-2.2005
H	5.285224	1.838743	-0.84614
H	3.340506	0.143264	-2.45667
H	5.368388	0.805969	-3.75774
H	6.407108	0.156168	-2.46891
H	5.394112	-0.93487	-3.4271
H	4.45292	-3.67511	-0.16091
H	2.793631	-3.56884	0.477451
H	3.537491	-1.54426	1.828933
H	2.843825	-1.92344	-2.9878
H	4.133504	-3.05812	-2.54255
H	2.440354	-3.42855	-2.14656
H	1.591633	-0.94271	-1.18555
H	5.140895	-3.48881	4.326152
H	5.955976	-2.11035	3.531007
H	4.244766	-1.9709	4.030028

Conf 7

E_{ZPC}= -1764.465808 Hartree

B3LYP/6-31G(d)

Symbol	X	Y	Z
C	4.912463	-2.47043	-0.02546
C	3.950256	-1.70097	-0.66911
C	4.12862	-0.3218	-0.87168
C	5.316628	0.251659	-0.40651
C	6.29656	-0.50412	0.241749
C	6.09474	-1.8758	0.437733
O	6.977702	-2.71262	1.055374

C	8.190655	-2.16539	1.547997
C	3.053049	0.520094	-1.52459
C	2.1351	1.236022	-0.508
N	1.453083	0.286219	0.363985
C	0.968081	2.029625	-1.17087
C	-0.35552	1.414794	-0.62158
C	0.106155	0.30122	0.343406
O	-0.61932	-0.47496	0.970443
C	1.066364	3.564741	-0.95991
C	0.824892	3.89141	0.500702
C	-0.47233	3.337116	1.083928
C	-1.25883	2.479383	0.07114
C	2.344511	4.1593	-1.55936
C	1.651302	4.602022	1.271961
O	-1.26605	4.444879	1.51406
O	-1.02552	0.816757	-1.72075
C	-2.48123	1.867134	0.70154
C	-3.7027	1.901017	0.155818
C	-4.89009	1.153888	0.693178
C	-5.27589	-0.081	-0.18575
C	-5.67184	0.331891	-1.61168
C	-4.20184	-1.16693	-0.14944
N	-3.48747	-1.2675	-1.21459
C	-3.99321	-2.02521	1.106214
C	-3.34325	-3.34825	0.674031
C	-2.14718	-3.09884	-0.2136
O	-2.48927	-2.23368	-1.33114
C	-5.28768	-2.31982	1.867209
O	-3.16439	-1.31301	2.034662
O	-1.71859	-4.31043	-0.74398
C	-0.49339	-4.23646	-1.46378
H	4.77425	-3.53659	0.125088
H	3.044341	-2.18143	-1.03266
H	5.49196	1.315341	-0.55514
H	7.204596	-0.01887	0.58138
H	8.731732	-2.99765	2.001614
H	8.797936	-1.73607	0.740112
H	8.006584	-1.3952	2.308753
H	2.424794	-0.10203	-2.17367
H	3.508616	1.289871	-2.16032
H	2.748394	1.900546	0.113246
H	1.956096	-0.41509	0.894698
H	0.970766	1.830486	-2.24711
H	0.22831	3.995841	-1.52796
H	-0.2254	2.705036	1.954902
H	-1.58894	3.1428	-0.73786
H	2.426904	3.895636	-2.61982
H	2.334706	5.251706	-1.48616
H	3.249097	3.803856	-1.05272

H	2.572385	5.037919	0.897052
H	1.41085	4.799037	2.313169
H	-2.14131	4.08401	1.740025
H	-1.81106	0.332038	-1.40182
H	-2.3319	1.31338	1.626705
H	-3.84386	2.445189	-0.77877
H	-4.68344	0.818371	1.713806
H	-5.76881	1.813323	0.72923
H	-6.16953	-0.51022	0.284432
H	-6.46925	1.083779	-1.5718
H	-4.82866	0.745779	-2.16875
H	-6.03943	-0.52971	-2.17731
H	-4.06162	-3.96009	0.115467
H	-3.02815	-3.90803	1.559948
H	-1.32816	-2.6029	0.324978
H	-6.02486	-2.8166	1.227792
H	-5.0581	-2.9784	2.711007
H	-5.72543	-1.40317	2.270648
H	-2.3368	-1.03006	1.595042
H	-0.21453	-5.26458	-1.70674
H	-0.60462	-3.65887	-2.38782
H	0.299321	-3.78278	-0.84943
